# What You Didn’t Learn in Residency: A Collective Curriculum for New Academic EM Faculty and Fellows

**DOI:** 10.21980/J8WP9Z

**Published:** 2024-01-31

**Authors:** Jessica Schmidt, Benjamin Schnapp, Sara Damewood, Mary Westergaard

**Affiliations:** * University of Wisconsin Madison, Department of Emergency Medicine, Madison, WI

## Abstract

**Audience and Type of Curriculum:**

This curriculum is designed for emergency medicine fellows and first-year junior faculty. The curriculum covers core topics related to academic and professional success for an early career faculty member.

**Length of Curriculum:**

The curriculum is designed as quarterly sessions over the course of one academic year.

**Introduction:**

An increasing number of emergency medicine graduates are pursuing fellowship after completion of residency.^1^ Fellowship can be challenging as newly minted graduates begin to explore their academic niche, refine their clinical practice, and define their personal and professional spheres. We propose a structured curriculum to help guide fellows and new faculty to mitigate these challenges.

**Educational Goals:**

The aim of this curriculum is to develop relevant skills to promote academic success for fellows and first-year faculty at the start of their academic career and which could be completed during a one-year training timeline. We included topics relevant to all fellow and new faculty’s expected personal and professional journey during this first year, including time management, academic productivity, resilience/wellness, and developing a national reputation.

**Educational Methods:**

The educational strategies used in this curriculum consist primarily of lecture seminars. There is one short individual activity associated with the lectures and one small group discussion.

**Research Methods:**

The course was assessed with pre- and post-test surveys following each lecture. Surveys assessed participants’ reaction, learning, and behavior for each session. Evaluations were completed based on a 5-point Likert scale (1=strongly disagree, 5=strongly agree).

**Results:**

Fifteen participants attended the seminar series encompassing fellows and first-year faculty/post-fellows from ten different fellowship subspecialities. Average pre-assessment scores were low for many of the self-reported skills and confidence throughout the seminar series. Overall, participants reported increased confidence on the post-test for each of the seminar topics. In addition, participants reported that they learned new skills and planned to use the new ideas presented. All participants reported they would recommend these seminars to someone else on their same career path.

**Discussion:**

Overall, participants reported increased confidence, new skills, and plans to use the ideas presented in the seminar series. The content appears applicable to this learner set since all reported they would recommend the series to others on their career path. In conclusion, we believe our seminar series will build skills for fellows and first-year faculty which will promote academic success.

**Topics:**

Academic success, professional development, early career development.

## USER GUIDE

[Table t3-jetem-9-1-c16]

**Table t3-jetem-9-1-c16:** 

List of Resources:	
Abstract	16
User Guide	18
Curriculum Chart	21
Appendix A: Time Management Slides.pptx	24
Appendix B: Time Management Worksheet	25
Appendix C: Time Management Instructor Guide	26
Appendix D: Resilience Slides.pptx	27
Appendix E: Resilience Activity Worksheet	28
Appendix F: Resilience Activity Instructor Guide	29
Appendix G: Academic Productivity Slides.pptx	31
Appendix H: How to Build a National Reputation Slides.pptx	32
Appendix I: Time Management Pre- and Post-Assessment Tool.docx	33
Appendix J: Resilience Pre- and Post-Assessment Tool.docx	35
Appendix K: Academic Productivity Pre-and Post-Assessment Tool.docx	37
Appendix L: Developing a National Reputation Pre-and Post-Assessment Tool.docx	39

**Learner Audience:**

Fellows and First-Year Junior Faculty

**Length of Curriculum:**

The curriculum is designed as quarterly sessions over the course of one academic year.

**Topics:**

Academic success, professional development, early career development.

**Objectives:**

By the end of this curriculum learners will be able to:

1. Describe skills to set priorities and manage time
2. Define strategies to promote resilience
3. State the importance of academic productivity
4. Identify steps needed to develop a national reputation
5. Stimulate connectedness among fellow and early faculty

### Brief introduction

An increasing number of trainees are pursuing fellowship after completion of residency.^1^ In emergency medicine, the number of Accreditation Council for Graduate Medical Education (ACGME) accredited and non-accredited fellowships has grown substantially over the past decade to include over 650 fellowship positions listed on the academic society page.^2^

Fellowships range from traditional disciplines such as emergency medical services (EMS) and toxicology to newer programs in medical informatics and health equity. Other specialties are also seeing increased growth in available programs and applicants.^3^

Fellowship, and the first years of academic practice, can be challenging as newly minted graduates begin to explore their academic niche, refine their clinical practice, and define their personal and professional spheres. Fellows/junior faculty are at risk of joining the increasing number of physicians suffering from burnout,^4^ struggling with work-life balance,^5^ and lacking mentorship.^6^ Previous authors have described the importance of building programs to guide physicians to help mitigate these challenges.^7^,^8^

### Problem identification, general and targeted needs assessment

To address these important topics, many individual fellowship program directors (PD) create their own ad hoc content. This leads to redundant work for PDs and variability in exposure for trainees.^9^ Although longer more specialty-focused resources have been developed for individual themes in personal and professional development, it would be difficult to implement all of this didactic content over a brief fellowship time-frame.^10^–^15^ In an effort to create a more standardized curriculum for fellows and first-year faculty that would still be feasible over the course of a one-year period, our educational group created a longitudinal seminar series with topics relevant to fellows’ expected personal and professional journey over the course of one year.

Our aim was to provide a longitudinal curriculum common to all fellows and early faculty that would build relevant skills over the course of the academic year. An initial list of themes was generated by education experts, and then four key topics were selected by consensus of a panel of expert faculty fellowship PDs. Topics that were unlikely to be covered extensively in the various fellowship curricula were prioritized. Ultimately the faculty PDs selected the following topics: time management, resilience, academic productivity, and developing a national reputation.

We were attentive to the timing of seminar topics to correspond with expected personal and professional development for individuals over the fellowship year or first year in academic medicine. In the beginning, as first-year attending physicians with increasing administrative responsibilities, skills in time management and prioritization are important. Approaching midyear and winter, we aimed to focus on building skills in resilience and maintaining wellness. Later in the year, fellows have more experience clinically and are better prepared to focus on scholarship and academic writing, and finally, as they gain a niche area of expertise, fellows benefit from skills to network and promote their work.

### Goals of the curriculum

The goal of our curriculum was to develop a relevant skill-set designed to create successful academic careers. A secondary goal was to promote relationship building between fellows and first-year faculty. As such, seminars were held at a time when there would be maximal attendance (immediately following monthly departmental Morbidity and Mortality conference), in person, and in a small conference room at a round table, to facilitate discussion and interaction.

### Objectives of the curriculum

By the end of this curriculum learners will be able to:

1. Describe skills to set priorities and manage time
2. Define strategies to promote resilience
3. State the importance of academic productivity
4. Identify steps needed to develop a national reputation
5. Stimulate connectedness among fellow and early faculty

### Educational strategies

Please see the below curriculum chart.

### Results and tips for successful implementation

We invited nine fellows and six first-year junior faculty to attend our seminar series. The average attendance for each seminar was seven participants. Ninety-seven percent of attendees completed the pre-surveys and 100% of attendees completed the post-surveys. Responses were based on a scale of 1 to 5 with 1=strongly disagree and 5=strongly agree.

Average pre-assessment scores were low for many of the self-reported skills and confidence throughout the seminar series (Table 1). The exception was resilience where a higher average, 4.13, was reported for those who have a resilience strategy, although with lower confidence in the ability to employ such strategies. Participants appeared to value academic productivity and resilience somewhat higher than time management and national reputation prior to the seminar series.

Post-assessment scores (Table 2) show that participants felt they developed new skills, reflected and thought about the topics, and planned to use the ideas presented in the future with scores ranging from 4.0–4.71. Overall, participants selected that they would recommend these seminars to peers, with a combined average score of 4.7 for the entire series.

### Associated content

Appendix A: Time Management Slides.pptx

Appendix B: Time Management Worksheet

Appendix C: Time Management Instructor Guide

Appendix D: Resilience Slides.pptx

Appendix E: Resilience Activity Worksheet

Appendix F: Resilience Activity Instructor Guide

Appendix G: Academic Productivity Slides.pptx

Appendix H: How to Build a National Reputation Slides.pptx

Appendix I: Time Management Pre- and Post-Assessment Tool.docx

Appendix J: Resilience Pre- and Post-Assessment Tool.docx

Appendix K: Academic Productivity Pre-and Post-Assessment Tool.docx

Appendix L: Developing a National Reputation Pre-and Post-Assessment Tool.docx

### Evaluation and feedback

The vast majority of attendees recommended the lectures to future learners. Comments made by participants included, “In all my training not once have I received a lecture on time management, much appreciated.”

Future interactions of this lecture series will be expanded based on participants’ interests and requests to include information on finances for early career physicians and also on growing leadership skills.

## Figures and Tables

**Table t4-jetem-9-1-c16:** 

Topic	Recommended Educational Strategy	Educational Content	Objectives	Learners	Timing, Resources Needed	Recommended Assessment, Milestones Addressed
Time Management	1. Lecture reframing time management concepts and introducing “calendaring”: *The Last “Time Management” Talk (Appendix A)* 2. Calendaring demonstration *(Appendix B,C)*	Five lessons in time management from the coaching industry. Introduce detailed framework for calendaring for academic physicians. Demonstration of calendaring.	Analyze how the 5 Lessons of Time Management apply to attendees. Describe the concept of calendaring. Outline a personalized strategy for a weekly Timeout session.	Fellows and junior faculty	35 minutes (lecture) 15 minutes (demonstration) Instructors: 1 Equipment: PowerPoint (and projector/screen) Pre-post assessment *(Appendix I)*	Milestone: PROF1, Level 2 Identifies and describes potential triggers and takes responsibility for professionalism lapses. PROF2, Level 3 Recognizes situations that might impact one’s own ability to complete tasks and responsibilities in a timely manner, and describes strategies for ensuring timely task completion in the future. Assessment: Pre and post-survey.
Burnout and Resilience	1. Lecture on burnout, wellness and tools to develop resilience: *Burnout and Resilience (Appendix D)* 2. Individual activity focus on resilience tools with focus on personal reflection (*Appendix E, F)*	What is burnout? What is wellness? How can you cultivate resilience?	Define the factors that lead to burnout and its effects on personal health and clinical care. Describe the importance of resilience and explain how it differs from wellness. Compare tools that can increase resilience including self-awareness and self-care, self-compassion, reframing and gratitude.	Fellows and junior faculty	50 minutes (lecture) 10 minute (individual activity) Pre-post assessment *(Appendix J)*	Milestone: PROF 3; Level 3 With assistance, proposes a plan to optimize personal and professional well-being. Assessment: Pre and post-survey.
Academic Productivity	1. Brief lecture on strategies to optimize efforts towards scholarly productivity: *How To Get Published in 15 Easy Rules (Appendix G)* 2. Small group discussion on difficulties encountered so far with scholarly work and strategies to overcome these.	Thinking about the work you already do from a scholarly perspective. Setting up scholarly projects for success. Avoiding problems that may delay or disrupt scholarly project completion.	List 15 rules to increase your academic productivity. Describe best practices and tips for keeping projects on schedule. Explain potential pitfalls that may prevent projects from getting completed.	Fellows and junior faculty	50 minutes (lecture) 20 minute (small group discussion) Pre-post assessment *(Appendix K)*	Milestone: SBP 2, Level 5: Creates, implements, and assesses quality improvement initiatives at the institutional or community level. ICS 3, Level 5: Facilitates dialogue regarding systems issues among larger community stakeholders (eg, institution, the health care system, and/or the field). Assessment: Pre and post-survey.
Developing a National Reputation	1. Lecture on general strategies of building national presence: *How to Build a National Reputation (Appendix H)* 2. Each fellow participant identifies tangible next action they will take to start getting a national reputation.	Description of strategies to build national reputation: research presentations and national organization involvement.	Understand the benefit of a national reputation. Optimize time at national EM conferences. Learn strategies to build national presence in EM.	Fellows and Junior Faculty	45 minutes (lecture) 10 minute (each participant says how they will build national presence) Pre-post assessment *(Appendix L)*	Milestones: PBLI 2, Level 3: Independently creates and implements a learning plan. ICS 3 Level 5 - Facilitates dialogue regarding systems issues among larger community stakeholders. Assessment: Pre and post-survey.

**Table 1:** Pre-survey results by seminar topic

Time Management		Resilience		Academic Productivity		National Reputation	
Transfer to-do list	3.0	Burnout current/past	4.00	Know definition	3.57	Previous interest	3.57
Written down priorities	3.29	Important to my career	3.88	Important to my career	4.00	Important to my career	3.14
Have a strategy	3.57	Have a strategy	4.13	Have a strategy	2.57	I have the skills	3.14
Confident in my ability	3.0	Confident in my ability	3.88	Confident in my ability	2.86	Confident in my ability	2.86
Consult my calendar daily	4.29	Have used strategies	3.75	Value the topic	4.00		

**Table 2:** Post-survey results by seminar topic

Time Management		Resilience		Academic Productivity		National Reputation	
Prepared me for time management	4.0	Better understanding of burnout	4.38	Better understanding of acad prod	4.71	Better understanding of nat’l rep	4.57
New skills	4.38	New skills	4.50	New skills	4.57	New skills	4.43
Think about the topic	4.38	Think about the topic	4.50	Think about the topic	4.71	Think about the topic	4.57
Plan to use new ideas	4.75	Plan to use new ideas	4.38	Plan to use new ideas	4.71	Plan to use new ideas	4.57
Recommend this lecture	4.88	Recommend this lecture	4.50	Recommend this lecture	4.71	Recommend this lecture	4.71

## DIDACTICS AND HANDS-ON CURRICULUM

### Curriculum Chart

[Table t4-jetem-9-1-c16]

## Appendix A Time Management Slides

Please see associated Power Point

## Appendix B Time Management Worksheet

Make a list of ALL the activities you have done today (put it all in, this means time spent commuting, eating, email, social media, working, social activities, sleeping)

**List your top 5 priorities (what you value most)**

1.

2.

3.

4.

5.

## Appendix C Time Management Instructor Guide

This exercise is meant to help participants evaluate how they are spending their time and where they want to spend time. Total time 10–15 minutes.

**Before the lecture begins:**

Allow participants 5 minutes to write down all activities they have completed today. This includes work, social, media, sleep, etc.
Next give another 2 minutes to have participants list their 5 top priorities or values (eg, family, sleep, clinical skills, exercise, career development, research, publications).

**Analysis of time spent and priorities:**

Take an additional 5 minutes to discuss the follow:
Describe the “stones and sand” metaphor and have participants re-examine their lists.
  Image you have the following: sand, pebbles, stones and big rocks. You want to fill a jar to the top.
  First, you put in the sand, then the small pebbles, and then stones. Your jar will be almost full, and you still have not fit in the big rocks.
  Now re-image filling the jar starting with the big rocks, then add the stones, and then pebbles and sand which will fill in the cracks.
Now use this metaphor to think about how you spend your time. Take a look back at your day’s activities. Where are you spending the most time? Are you able to prioritize the big rocks?
Time management allows you to be purposeful about how to spend your time. It will not give you more time, but will allow you to structure your day to make sure you are spending time on your priorities and not have those things left “outside the jar.”
Allow participants to reflect on how they spend their time and if this aligns with their priorities and values.

## Appendix D Resilience Slides

Please see associated Power Point

## Appendix E Resilience Activity Worksheet

First, think about this: You are working your first “real” job as an attending/fellow. Everyone has something on their mind that is bothering them, a nagging thought, a hard case, a potential error. Write it here, fold the paper in half, and turn in over. (We will come back to this later when we have some tools but you WILL NOT HAVE TO SHARE IT; this is just for you.)[Fig f1-jetem-9-1-c16][Table t5-jetem-9-1-c16]

**Table t5-jetem-9-1-c16:**

	**Self-Awareness and Self-care:** Identify yourself (introvert/extrovert/mix). Name 2–3 ways to “recharge” that are important to you. Name 2–3 things you have done today and what you would LIKE to do for self-care.
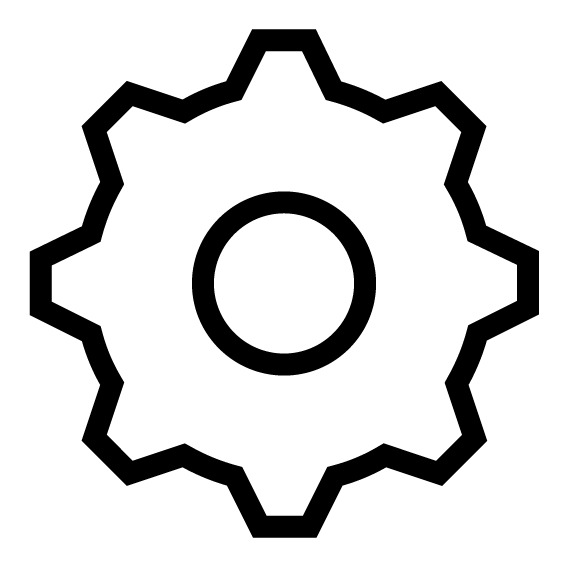
	**Self-Compassion:** Think of a life stress that causes a physical discomfort (shoulders tense/stomach churning). Acknowledge it: say to yourself, "This hurts," or "This is a moment of stress." (This is called mindfulness.) Recognize that element of the human experience and give yourself grace. (Examples: I will forgive myself, I accept myself as I am right now.)
	**Reframing:** Identify the “mind-ruts” you had created in the above example from Self-Compassion. (What did you say in your head/what was the “inner nag”?) Reframe that example in a more positive way.
	**Gratitude:** Write down 3 good things:

## Appendix F Resilience Activity Instructor Guide

This exercise is intended to frame the lecture topic in an individual and meaningful way for each participant.

**Materials:** Provided worksheet (Appendix B Exercise Worksheet) or use blank paper, pencil/pen.

**Before the lecture begins:**

Provide 2–5 minutes for reflection on the instructions below. The aim is to have participants think of the “first-thing” that comes to mind--something that may be bothering them--and will serve as a point on which they can apply the tools learned later in the lecture.

**Participant Instructions:**

*First, think about this: You are working your first “real” job as an attending/fellow. Everyone has something on their mind that is bothering them, a nagging thought, a hard case, a potential error. Write it here, fold the paper in half, and turn it over.*

*(We will come back to this later when we have some tools but you WILL NOT HAVE TO SHARE IT; this is just for you.)*

During the **second half of the lecture**, there will be several prompts on Slides 16, 18, 20, 22 with the below icon. PAUSE at these slides and ask the participants to write (or reflect) at each of these steps.[Fig f2-jetem-9-1-c16]

**Self-Awareness and Self-care:**

1. Identify yourself (introvert/extrovert/mix). Name 2–3 ways to “recharge” that are important to you.
2. Name 2–3 things you have done today and what you would LIKE to do for self-care.

**Self-Compassion:**

1. First, think about something happening in your life that is causing you stress in a way that you can actually feel physical discomfort. Perhaps it's your head hurting, your shoulders tensing up, a knot in your stomach or sternum, or tightness in your jaw.
2. Once you can feel the stress and discomfort, say to yourself, "This hurts," or "This is a moment of stress." This is mindfulness: witnessing the feeling, acknowledging it, and not fighting it off.
3. Then, it's time to recognize that element of human experience by saying something to yourself like, "We all struggle," or "other people feel this way, too," or "I'm not alone in this." You might even try putting your hands on your heart or somewhere else on your body that feels loving and warm. Think about a phrase that could be comforting; ask yourself, "What do I need to hear right now?" Examples of this self-compassion could be:
  *I will be patient with myself.*
  *I forgive myself.*
  *I will learn to accept myself as I am, right now.*
  *I will give myself the compassion that I need.*

**Reframing:**

1. Ask participants to identify the “mind-ruts” they had created in their heads in the above example from Self-Compassion. (What did you say in your head, what was the “inner nag,” what emotion arose?)
2. Ask them to reframe that example in a more positive way from a different perspective: imagine you are someone external hearing this same issue. What might you say?

**Gratitude:**

Ask each participant to write down 3 “good” things or things that brought them joy today. They do not have to be big or earth-shattering; it could be as simple as “I got a parking spot close to the entrance.”

## Appendix G Academic Productivity Slides

Please see associated Power Point

## Appendix H How to Build a National Reputation Slides

Please see associated Power Point

## Appendix I Time Management Pre- and Post- Assessment Tool

### TIME MANAGEMENT

#### PRE-LECTURE

1. I have a defined strategy for time management.[Table t6-jetem-9-1-c16]
  **Table t6-jetem-9-1-c16:**
  Strongly disagree	Disagree	Neutral	Agree	Strongly Agree
2. I am confident in my time management skills.[Table t7-jetem-9-1-c16]
  **Table t7-jetem-9-1-c16:**
  Strongly disagree	Disagree	Neutral	Agree	Strongly Agree
3. I transfer my To Do list items directly onto my calendar.[Table t8-jetem-9-1-c16]
  **Table t8-jetem-9-1-c16:**
  Strongly disagree	Disagree	Neutral	Agree	Strongly Agree
4. I consult my calendar every day.[Table t9-jetem-9-1-c16]
  **Table t9-jetem-9-1-c16:**
  Strongly disagree	Disagree	Neutral	Agree	Strongly Agree

#### POST-LECTURE

1. The content of this lecture has prepared me to manage my time better.[Table t10-jetem-9-1-c16]
  **Table t10-jetem-9-1-c16:**
  Strongly disagree	Disagree	Neutral	Agree	Strongly Agree
2. This lecture gave me skills I will use in my academic career.[Table t11-jetem-9-1-c16]
  **Table t11-jetem-9-1-c16:**
  Strongly disagree	Disagree	Neutral	Agree	Strongly Agree
3. This lecture made me think differently about how to manage my time.[Table t12-jetem-9-1-c16]
  **Table t12-jetem-9-1-c16:**
  Strongly disagree	Disagree	Neutral	Agree	Strongly Agree
4. I plan to use the ideas discussed in today’s lecture in the future.[Table t13-jetem-9-1-c16]
  **Table t13-jetem-9-1-c16:**
  Strongly disagree	Disagree	Neutral	Agree	Strongly Agree
5. I would recommend this lecture for future fellows and new faculty.[Table t14-jetem-9-1-c16]
  **Table t14-jetem-9-1-c16:**
  Strongly disagree	Disagree	Neutral	Agree	Strongly Agree

## Appendix J Resilience Pre- and Post- Assessment Tool

### RESILIENCE

#### PRE-LECTURE

1. I currently suffer or have previously suffered from burnout.[Table t15-jetem-9-1-c16]
  **Table t15-jetem-9-1-c16:**
  Strongly disagree	Disagree	Neutral	Agree	Strongly Agree
2. I am concerned burnout will affect my career.[Table t16-jetem-9-1-c16]
  **Table t16-jetem-9-1-c16:**
  Strongly disagree	Disagree	Neutral	Agree	Strongly Agree
3. I have strategies to manage burnout.[Table t17-jetem-9-1-c16]
  **Table t17-jetem-9-1-c16:**
  Strongly disagree	Disagree	Neutral	Agree	Strongly Agree
4. I have previously employed strategies to promote resilience.[Table t18-jetem-9-1-c16]
  **Table t18-jetem-9-1-c16:**
  Strongly disagree	Disagree	Neutral	Agree	Strongly Agree
5. I am confident in my ability to be resilient.[Table t19-jetem-9-1-c16]
  **Table t19-jetem-9-1-c16:**
  Strongly disagree	Disagree	Neutral	Agree	Strongly Agree

#### POST-LECTURE

1. I have a better understanding of burnout.[Table t20-jetem-9-1-c16]
  **Table t20-jetem-9-1-c16:**
  Strongly disagree	Disagree	Neutral	Agree	Strongly Agree
2. This lecture made me think about the idea of resilience.[Table t21-jetem-9-1-c16]
  **Table t21-jetem-9-1-c16:**
  Strongly disagree	Disagree	Neutral	Agree	Strongly Agree
3. This lecture gave me skills to promote resilience.[Table t22-jetem-9-1-c16]
  **Table t22-jetem-9-1-c16:**
  Strongly disagree	Disagree	Neutral	Agree	Strongly Agree
4. I plan to use the tools I learned today in the future.[Table t23-jetem-9-1-c16]
  **Table t23-jetem-9-1-c16:**
  Strongly disagree	Disagree	Neutral	Agree	Strongly Agree
5. I would recommend this lecture to someone else in my career.[Table t24-jetem-9-1-c16]
  **Table t24-jetem-9-1-c16:**
  Strongly disagree	Disagree	Neutral	Agree	Strongly Agree

## Appendix K Academic Productivity Pre- and Post-Assessment Tool

### ACADEMIC PRODUCTIVITY

#### PRE-LECTURE

1. I know what academic productivity means.[Table t25-jetem-9-1-c16]
  **Table t25-jetem-9-1-c16:**
  Strongly disagree	Disagree	Neutral	Agree	Strongly Agree
2. I value academic productivity.[Table t26-jetem-9-1-c16]
  **Table t26-jetem-9-1-c16:**
  Strongly disagree	Disagree	Neutral	Agree	Strongly Agree
3. Being academically productive is important in my career.[Table t27-jetem-9-1-c16]
  **Table t27-jetem-9-1-c16:**
  Strongly disagree	Disagree	Neutral	Agree	Strongly Agree
4. I have a strategy for academic productivity.[Table t28-jetem-9-1-c16]
  **Table t28-jetem-9-1-c16:**
  Strongly disagree	Disagree	Neutral	Agree	Strongly Agree
5. I am confident in my ability to be academically productive.[Table t29-jetem-9-1-c16]
  **Table t29-jetem-9-1-c16:**
  Strongly disagree	Disagree	Neutral	Agree	Strongly Agree

#### POST-LECTURE

1. I have a better understanding of academic productivity.[Table t30-jetem-9-1-c16]
  **Table t30-jetem-9-1-c16:**
  Strongly disagree	Disagree	Neutral	Agree	Strongly Agree
2. This lecture made me think about new or different types of academic productivity.[Table t31-jetem-9-1-c16]
  **Table t31-jetem-9-1-c16:**
  Strongly disagree	Disagree	Neutral	Agree	Strongly Agree
3. This lecture gave me skills I plan to use in the future.[Table t32-jetem-9-1-c16]
  **Table t32-jetem-9-1-c16:**
  Strongly disagree	Disagree	Neutral	Agree	Strongly Agree
4. I plan to use the ideas discussed in today’s lecture in the future.[Table t33-jetem-9-1-c16]
  **Table t33-jetem-9-1-c16:**
  Strongly disagree	Disagree	Neutral	Agree	Strongly Agree
5. I would recommend this lecture to someone else on my career path.[Table t34-jetem-9-1-c16]
  **Table t34-jetem-9-1-c16:**
  Strongly disagree	Disagree	Neutral	Agree	Strongly Agree

## Appendix L Developing a National Reputation Pre- and Post-Assessment Tool

### DEVELOPING A NATIONAL REPUTATION

#### PRE-LECTURE

1. I have previously been interested in involvement in work at a national level.[Table t35-jetem-9-1-c16]
  **Table t35-jetem-9-1-c16:**
  Strongly disagree	Disagree	Neutral	Agree	Strongly Agree
2. I believe having a national reputation is important to my career.[Table t36-jetem-9-1-c16]
  **Table t36-jetem-9-1-c16:**
  Strongly disagree	Disagree	Neutral	Agree	Strongly Agree
3. I believe I have skills to develop my own national reputation.[Table t37-jetem-9-1-c16]
  **Table t37-jetem-9-1-c16:**
  Strongly disagree	Disagree	Neutral	Agree	Strongly Agree
4. I am confident in my ability to develop my national reputation.[Table t38-jetem-9-1-c16]
  **Table t38-jetem-9-1-c16:**
  Strongly disagree	Disagree	Neutral	Agree	Strongly Agree

#### POST-LECTURE

1. I have a better understanding of how to develop a national reputation.[Table t39-jetem-9-1-c16]
  **Table t39-jetem-9-1-c16:**
  Strongly disagree	Disagree	Neutral	Agree	Strongly Agree
2. This lecture made me think about the benefits of building a national reputation.[Table t40-jetem-9-1-c16]
  **Table t40-jetem-9-1-c16:**
  Strongly disagree	Disagree	Neutral	Agree	Strongly Agree
3. In this lecture I learned new skills to develop a national reputation or use at the next national conference I attend.[Table t41-jetem-9-1-c16]
  **Table t41-jetem-9-1-c16:**
  Strongly disagree	Disagree	Neutral	Agree	Strongly Agree
4. I plan to use the skills I learned today in the future.[Table t42-jetem-9-1-c16]
  **Table t42-jetem-9-1-c16:**
  Strongly disagree	Disagree	Neutral	Agree	Strongly Agree
5. I would recommend this lecture to someone else in my career.[Table t43-jetem-9-1-c16]
  **Table t43-jetem-9-1-c16:**
  Strongly disagree	Disagree	Neutral	Agree	Strongly Agree

## References

[1] Nelson LS, Keim SM, Ankel FK (2020). American board of emergency medicine report on residency and fellowship training information (2019–2020). Ann Emerg Med.

[2] Society for Academic Emergency Medicine (2022). Fellowship Directory.

[3] National Resident Matching Program (2022). Results and Data: Specialties Matching Service 2022 Appointment Year. National Resident Matching Program.

[4] Zhang Q, Mu MC, He Y, Cai ZL, Li ZC (2020). Burnout in emergency medicine physicians: A meta-analysis and systematic review. Medicine (Baltimore).

[5] Shanafelt TD, Boone S, Tan L (2012). Burnout and satisfaction with work-life balance among US physicians relative to the general US population. Arch Intern Med.

[6] Lucas RH, Dandar V (2021). Importance of mentoring on workplace engagement of emergency medicine faculty: a multi-institutional study. West J Emerg Med.

[7] Thorndyke LE, Gusic ME, George JH, Quillen DA, Milner RJ (2006). Empowering junior faculty: Penn State's faculty development and mentoring program. Acad Med.

[8] Childs E, Remein CD, Bhasin RM (2021). How to launch and continually enhance an effective medical campus faculty development program: steps for implementation and lessons learned. J Healthc Leadersh.

[9] Petinaux B, Douglass K, Lee J (2012). Emergency medicine Joint Fellowship Curriculum. J Emerg Med.

[10] Li ST, Gusic ME, Vinci RJ, Szilagyi PG, Klein MD (2018). A structured framework and resources to use to get your medical education work published. MedEdPORTAL.

[11] Karpinski J (2015). Residents as leaders: A comprehensive guide to establishing a leadership development program for postgraduate trainees. MedEdPortal.

[12] Nagy C, Schwabe D, Jones W (2016). Time to talk about it: physician depression and suicide video/discussion session for interns, residents, and fellows. MedEDPortal.

[13] Uijtdehaage S, Kalishman S, O'Sullivan P, Robins L (2013). How to succeed as a medical education scholar: identifying your individual strategy and creating a roadmap for scholarship. MedEdPortal.

[14] Pitre C, Pettit K, Ladd L, Chisholm C, Welch JL (2018). Physician time management. MedEdPORTAL.

[15] Loyal J, Porto A, Camenga D (2018). Creating a program for junior faculty professional development: a tool kit. MedEdPORTAL.

