# Supplementary material for: What You Didn’t Learn in Residency: A Collective Curriculum for New Academic EM Faculty and Fellows
Source: J Educ Teach Emerg Med. 2024 Jan 31;9(1):C16–40. doi: 10.21980/J8WP9Z (PMC10854884; doi:10.21980/J8WP9Z)
Supplement: Supplementary file 1 — Please see associated Power Point [file jetem-9-1-C16-AppendixA.pptx]

## Slide 1
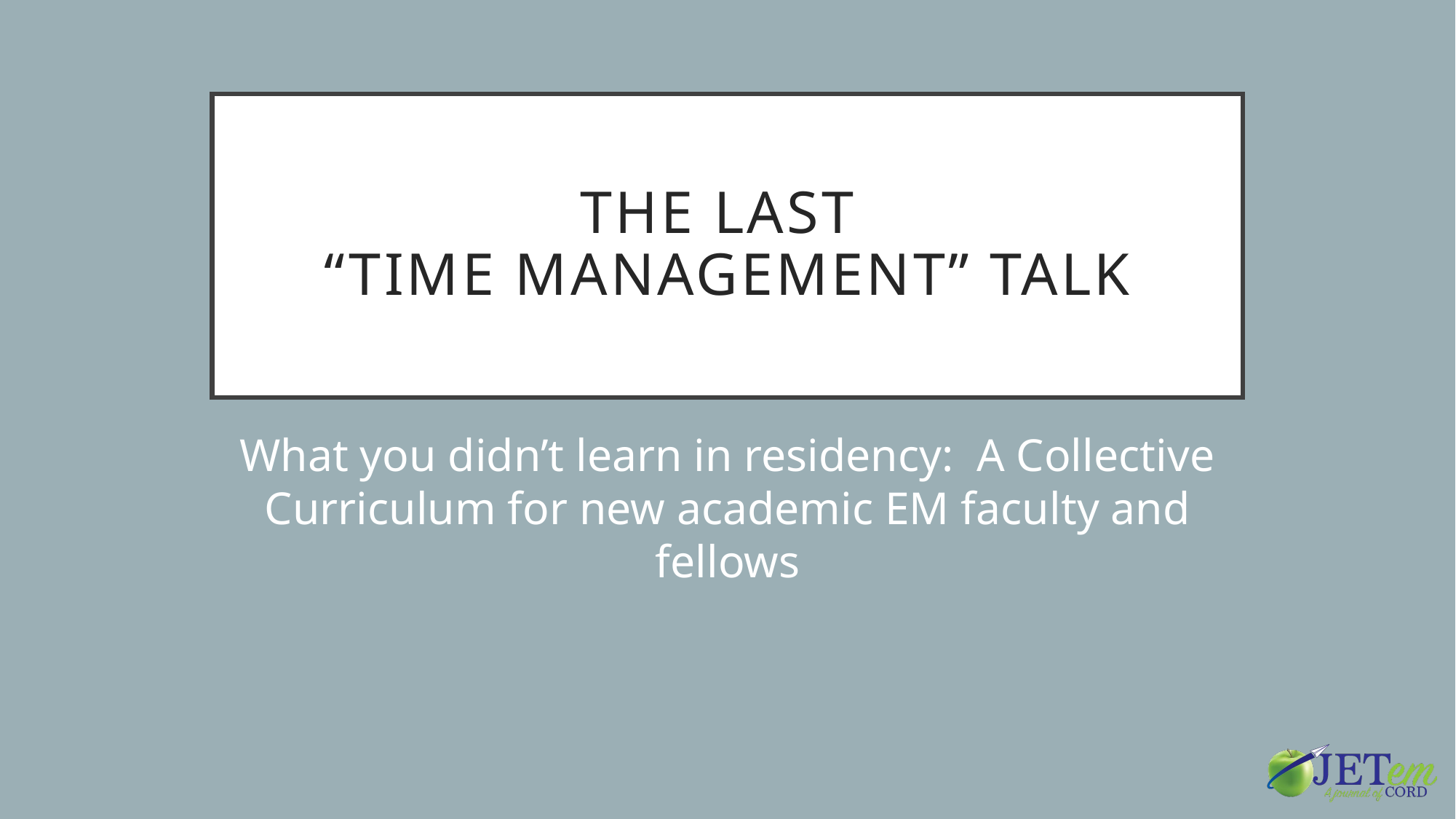

# The last “Time Management” talk
What you didn’t learn in residency: A Collective Curriculum for new academic EM faculty and fellows

## Slide 2
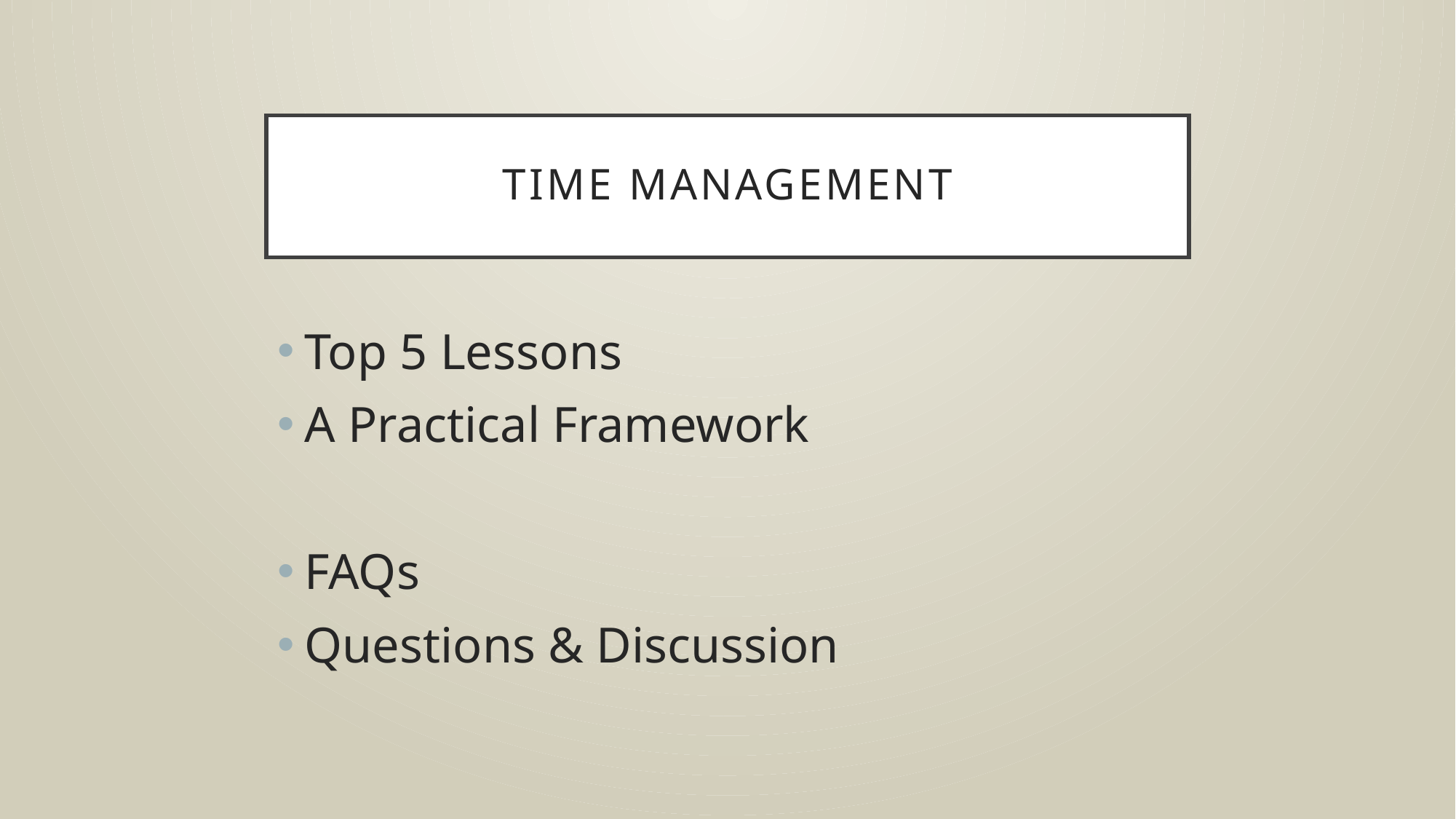

# Time management
Top 5 Lessons
A Practical Framework
FAQs
Questions & Discussion

## Slide 3
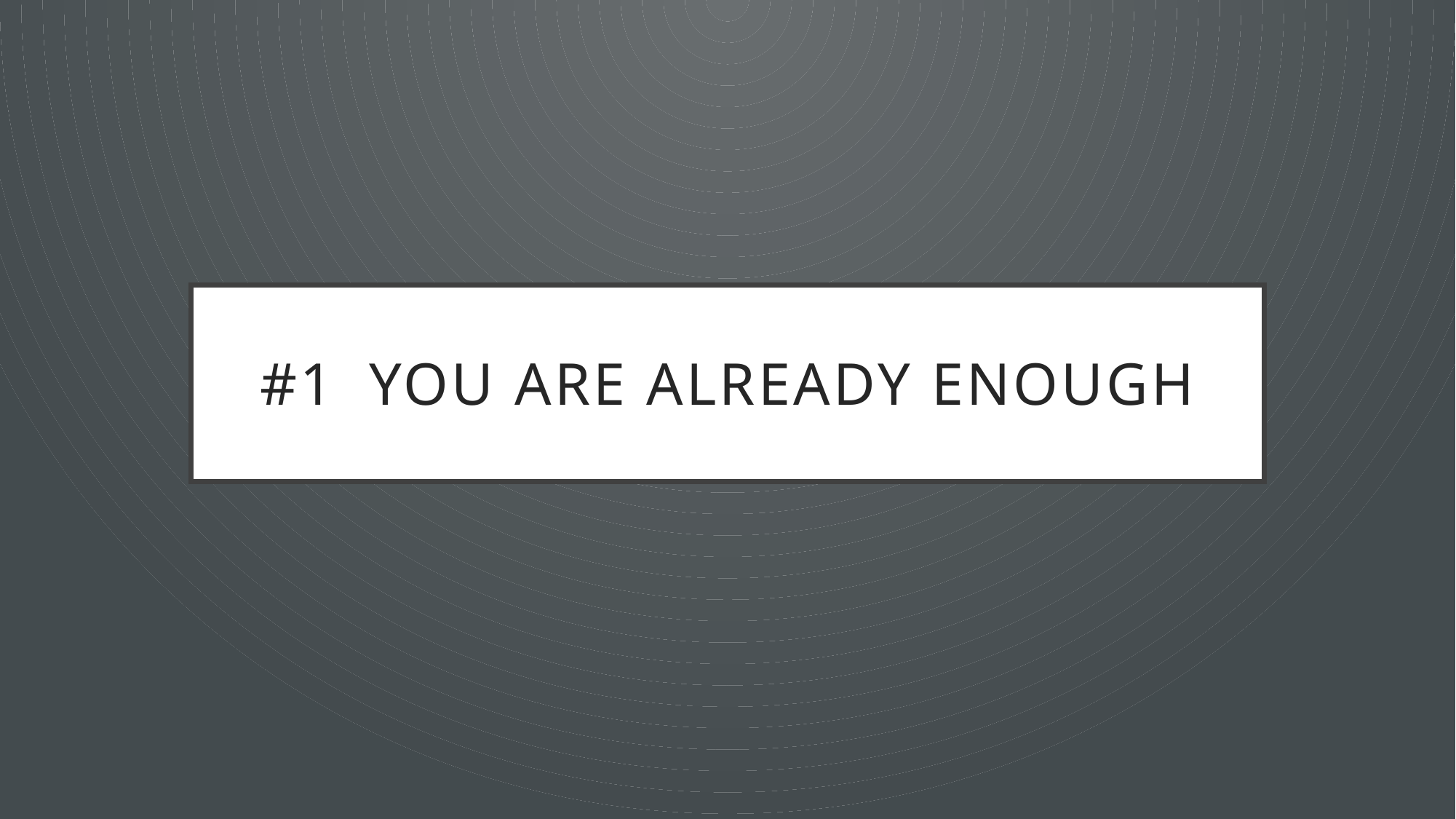

# #1 	you are already enough

## Slide 4
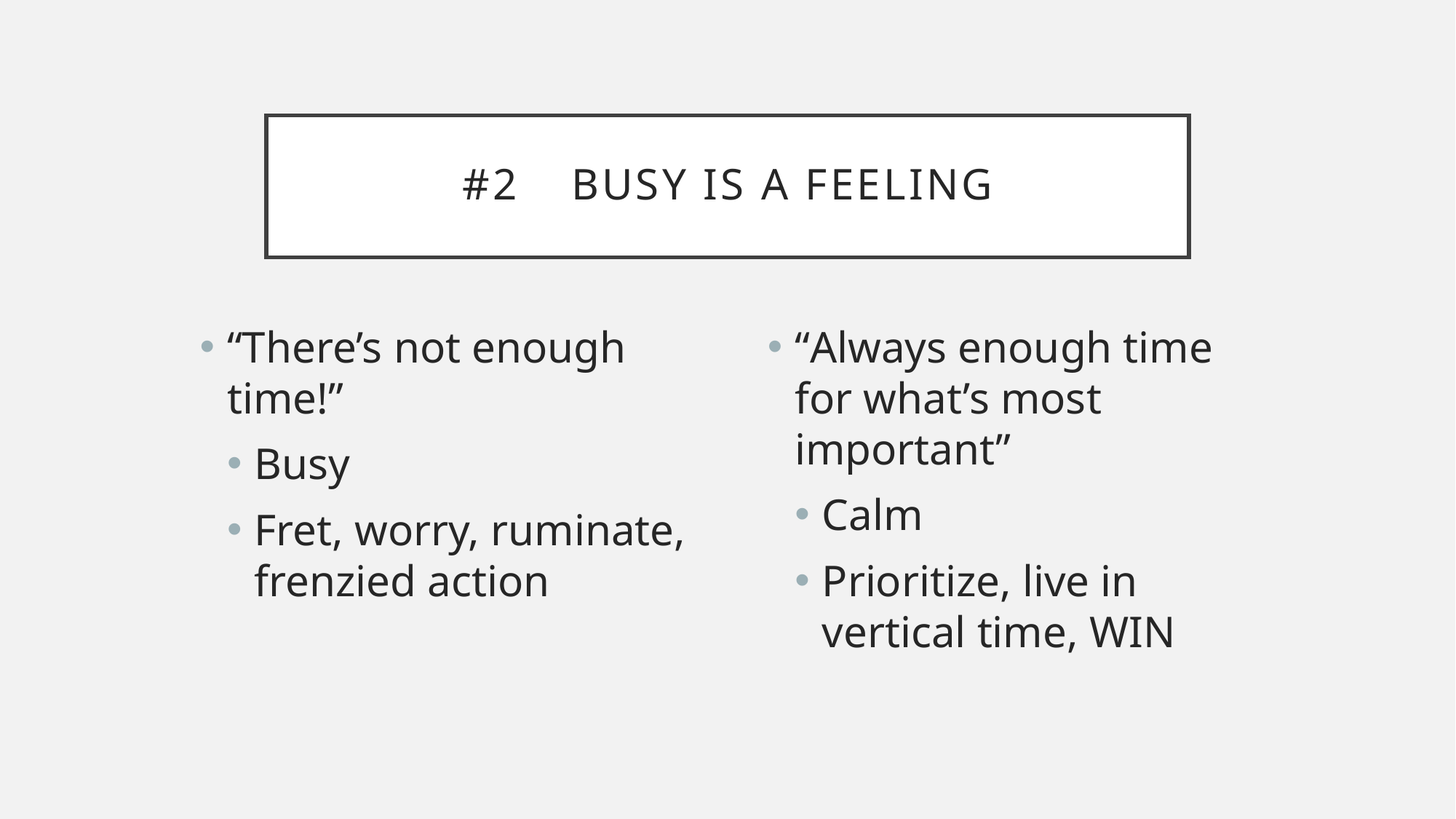

# #2 	busy is a feeling
“There’s not enough time!”
Busy
Fret, worry, ruminate, frenzied action
“Always enough time for what’s most important”
Calm
Prioritize, live in vertical time, WIN

## Slide 5
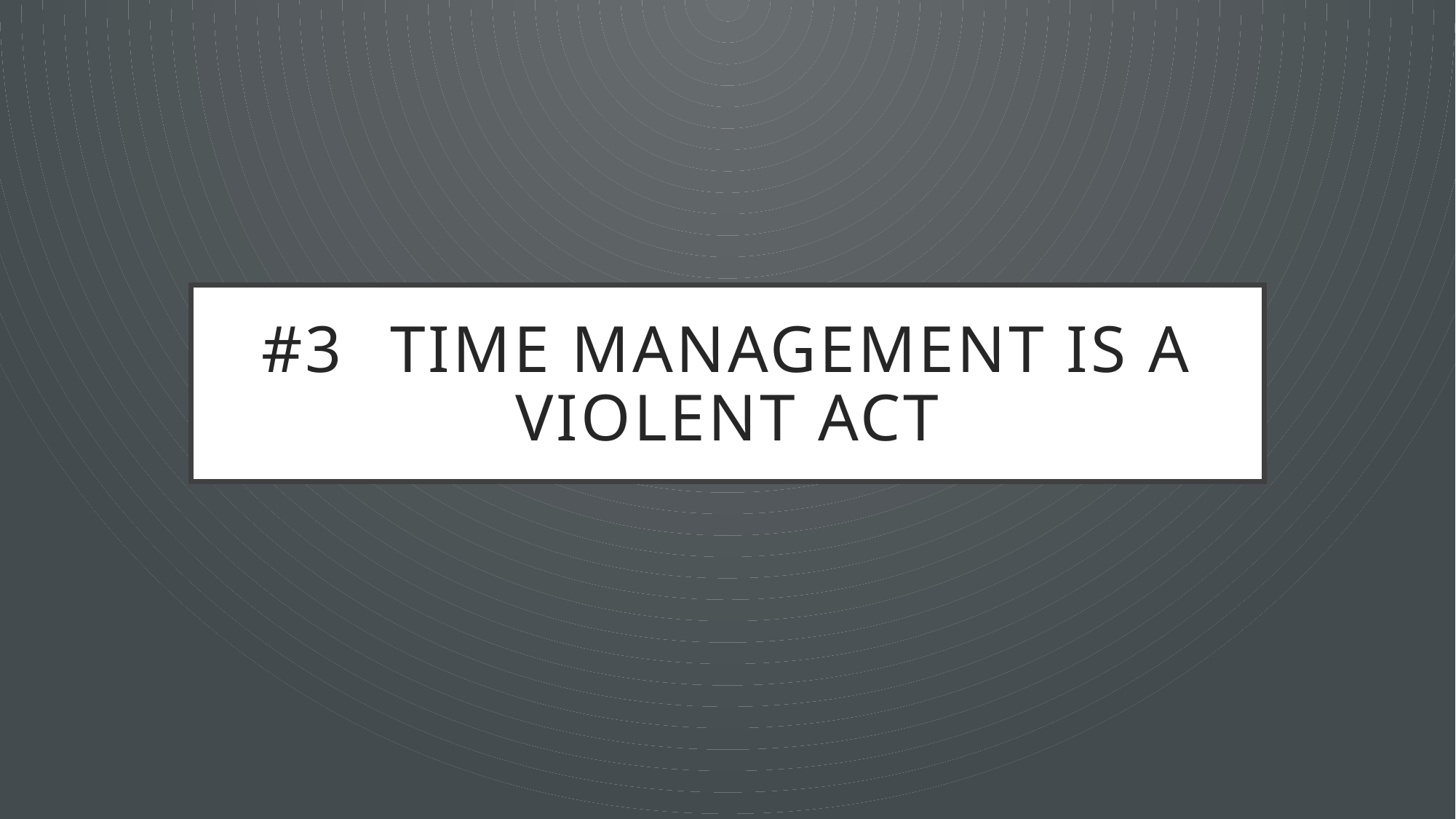

# #3	 Time management is a violent act

## Slide 6
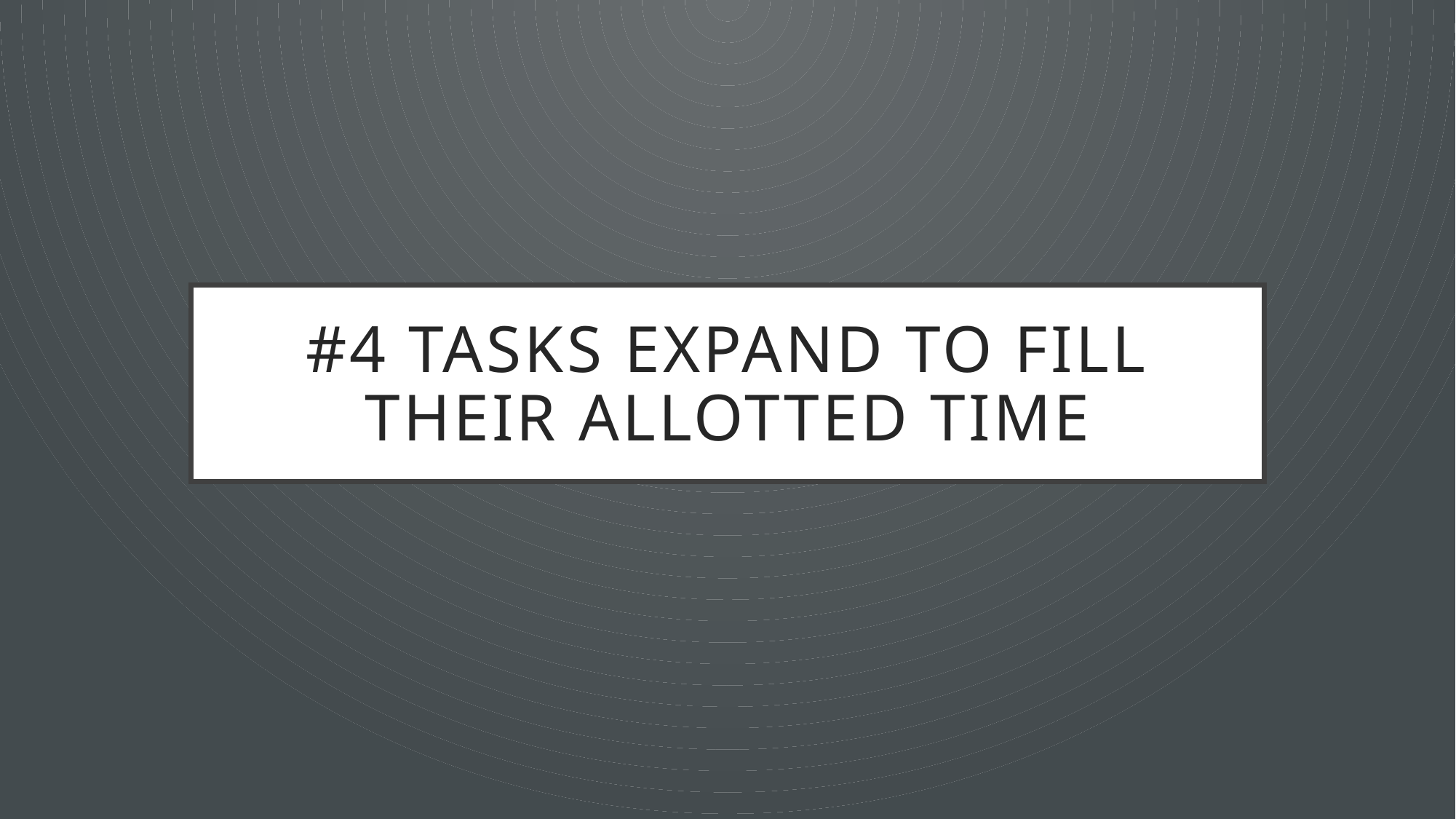

# #4 Tasks expand to fill their allotted time

## Slide 7
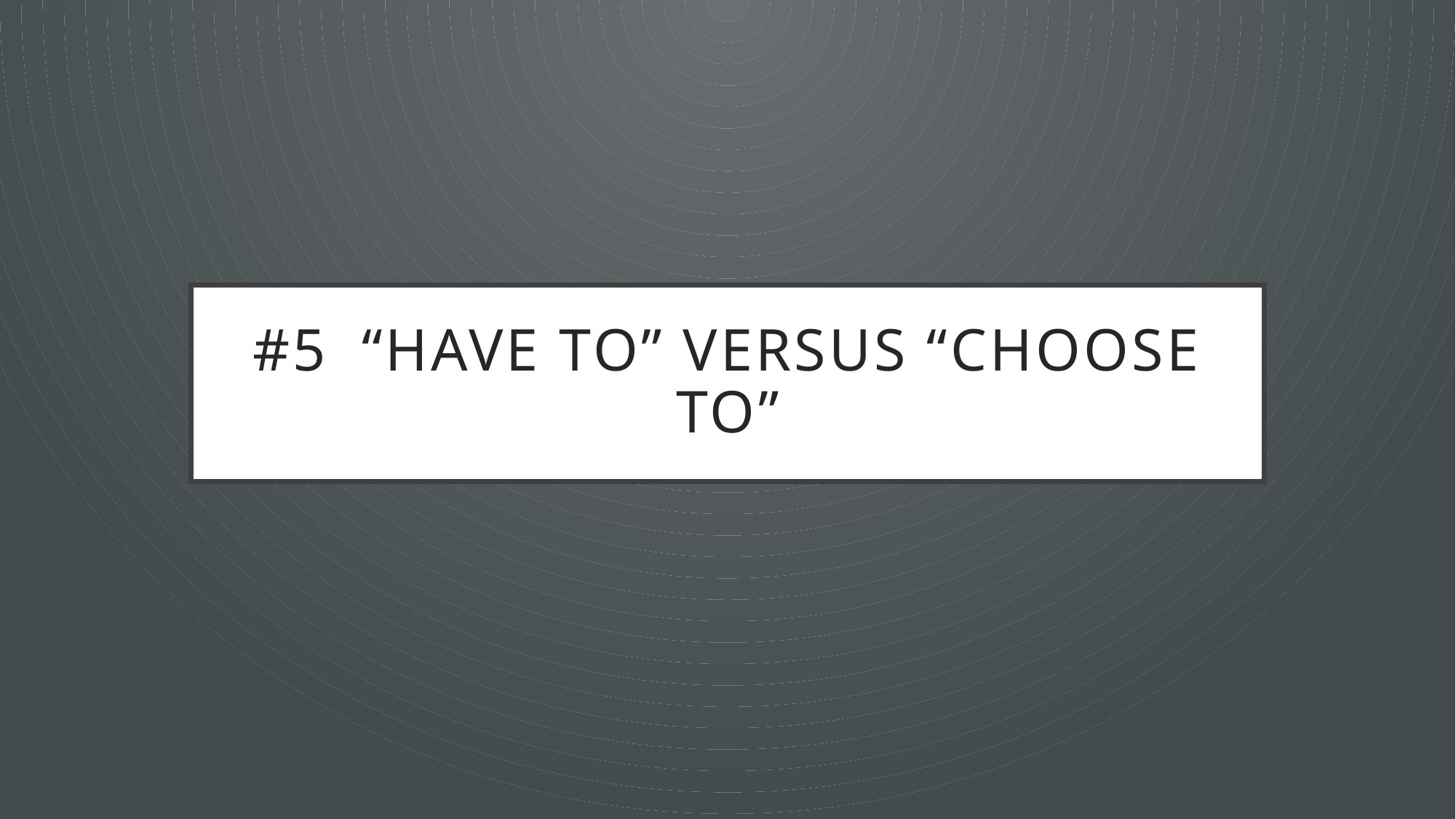

# #5 	“Have to” versus “choose to”

## Slide 8
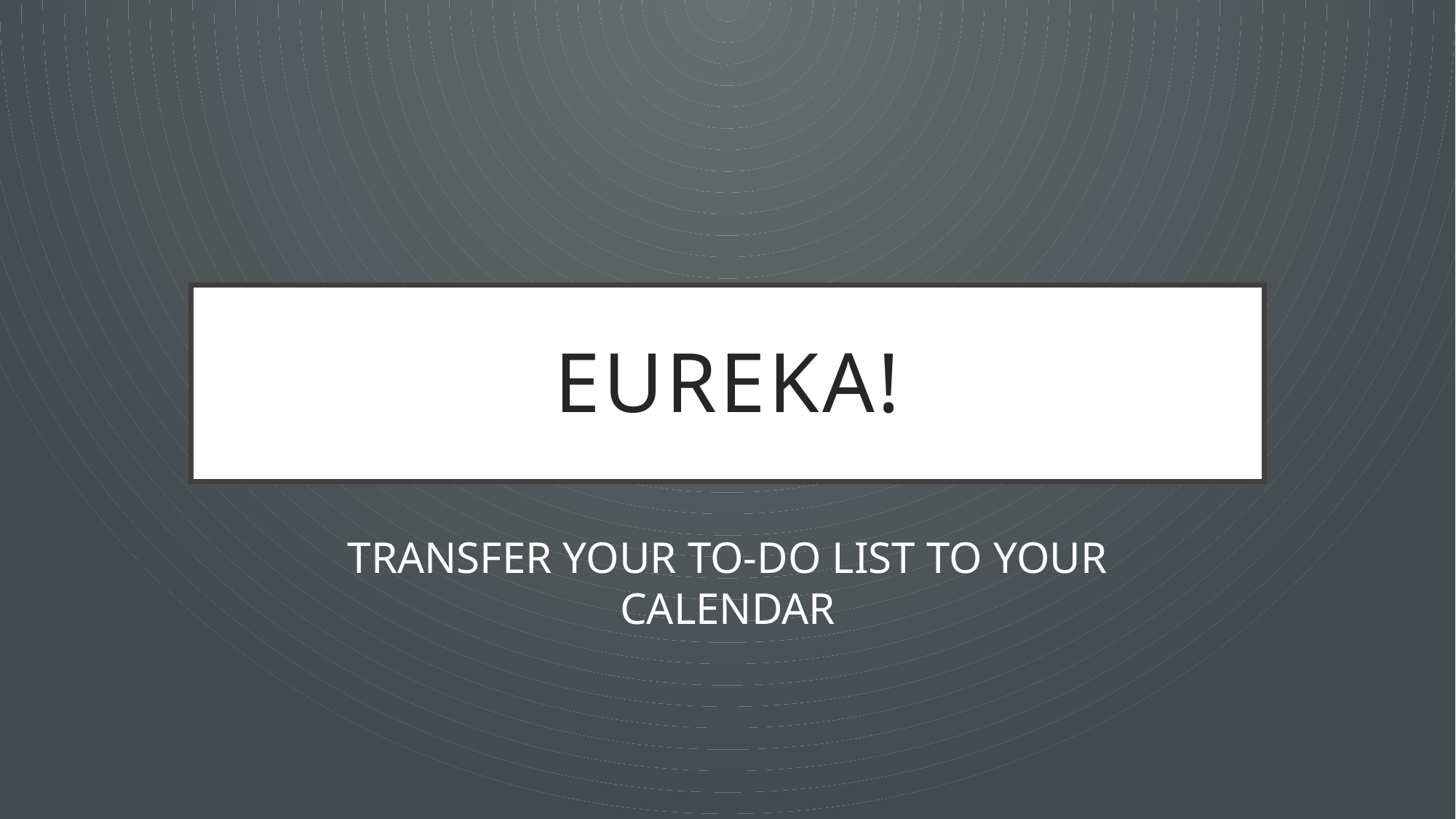

# Eureka!
TRANSFER YOUR TO-DO LIST TO YOUR CALENDAR

## Slide 9
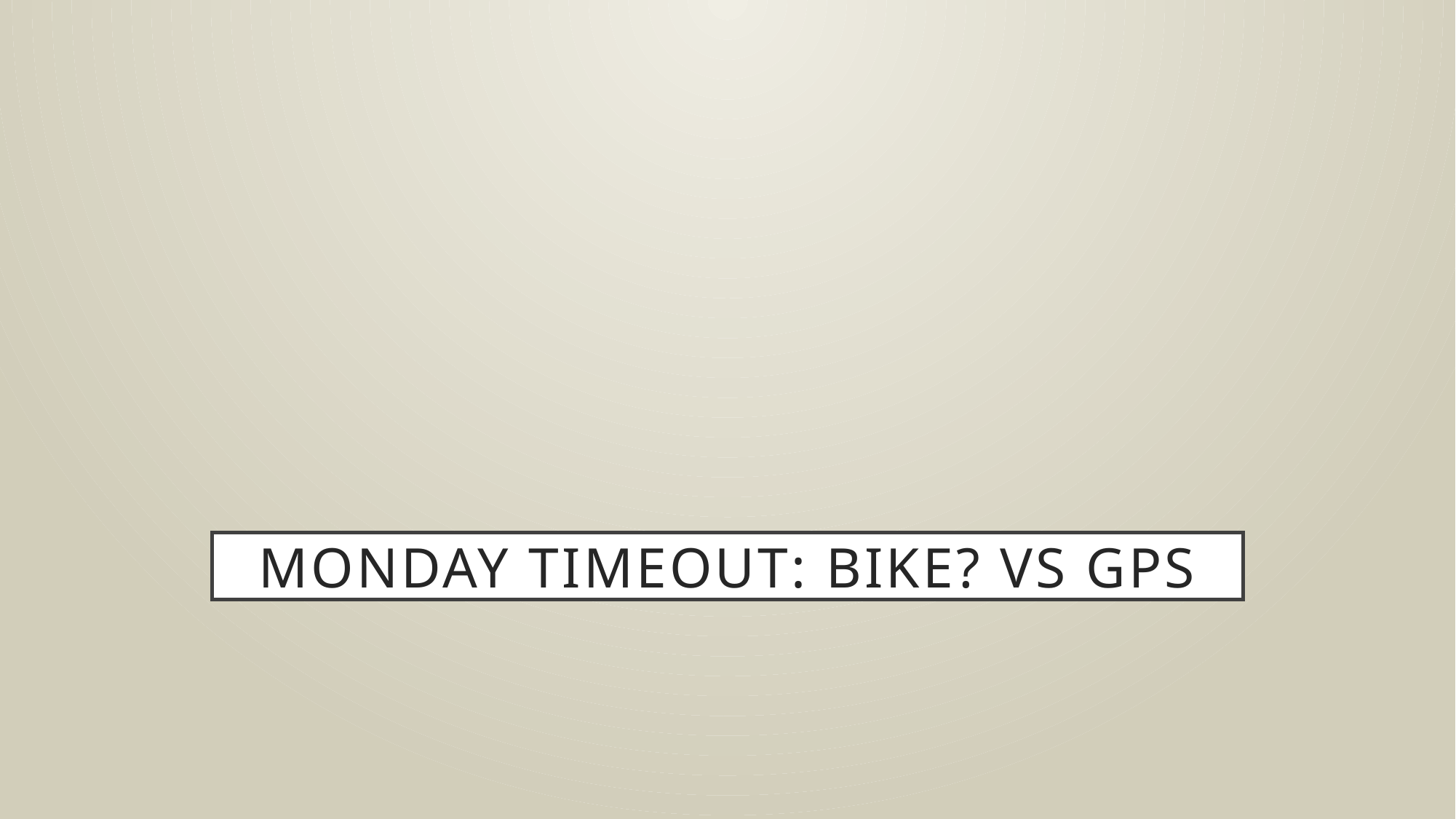

# Monday Timeout: Bike? vs GPS

## Slide 10
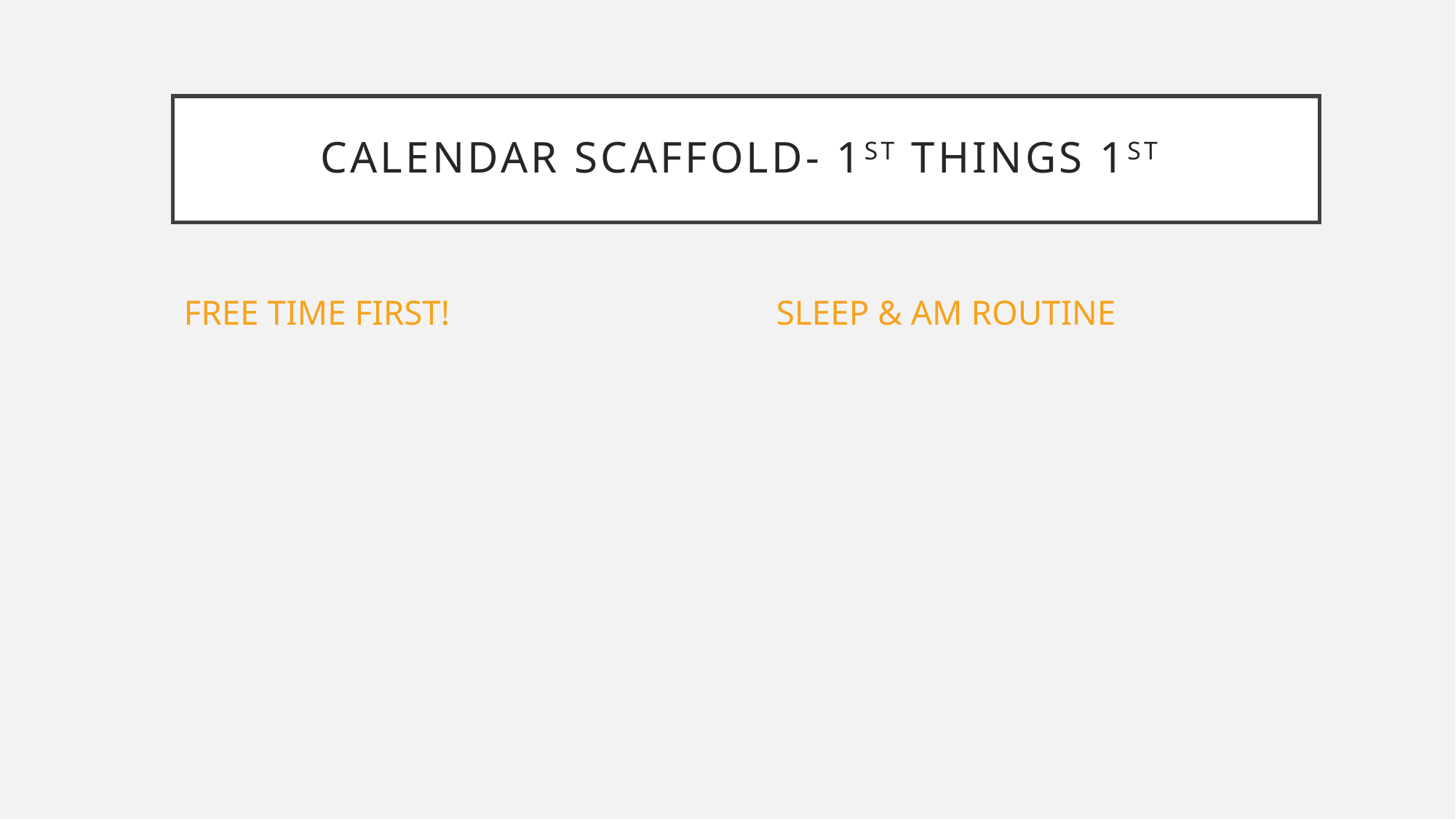

# Calendar scaffold- 1st things 1st
Free time first!
Sleep & Am routine

## Slide 11
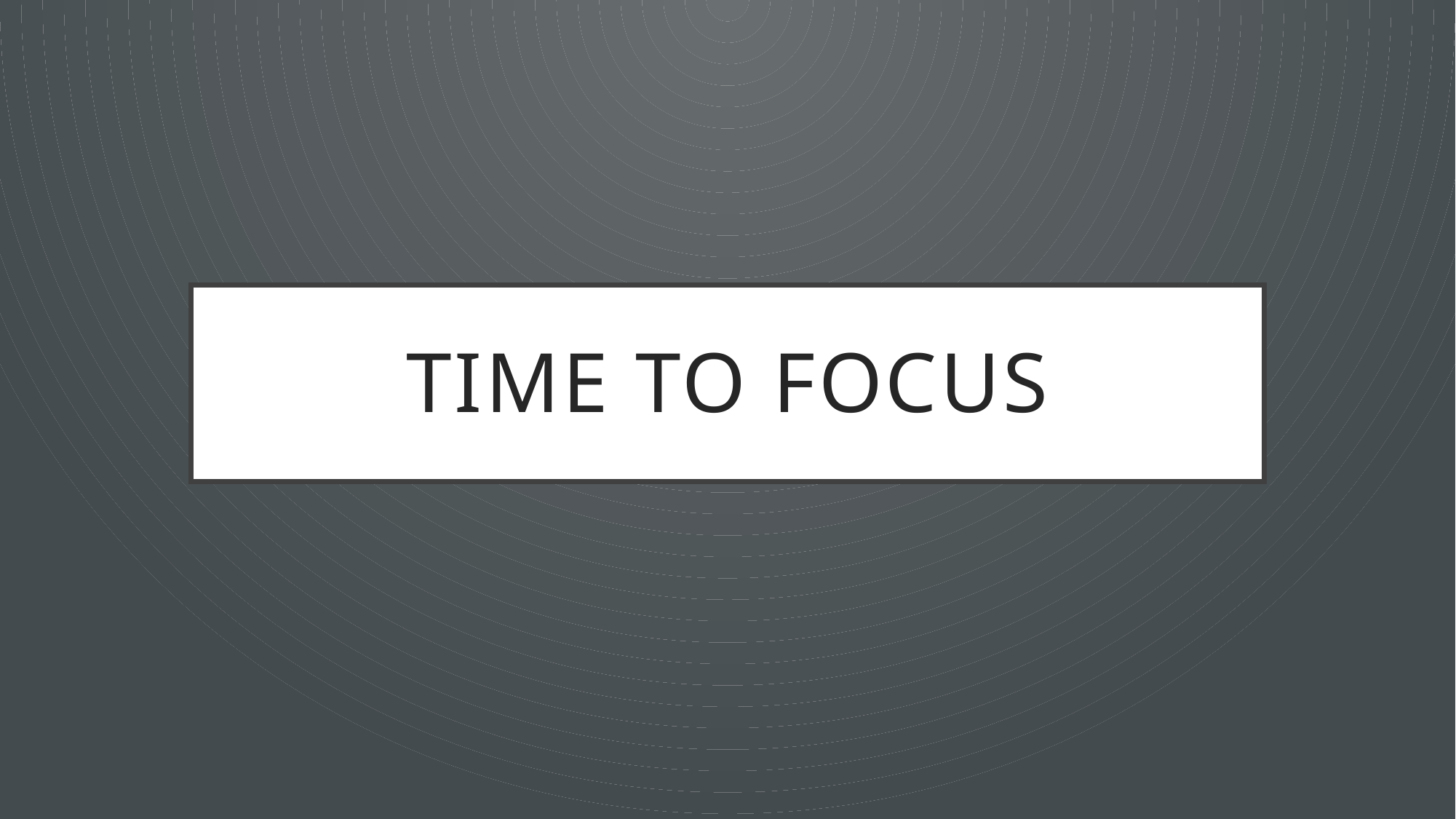

# Time to focus

## Slide 12
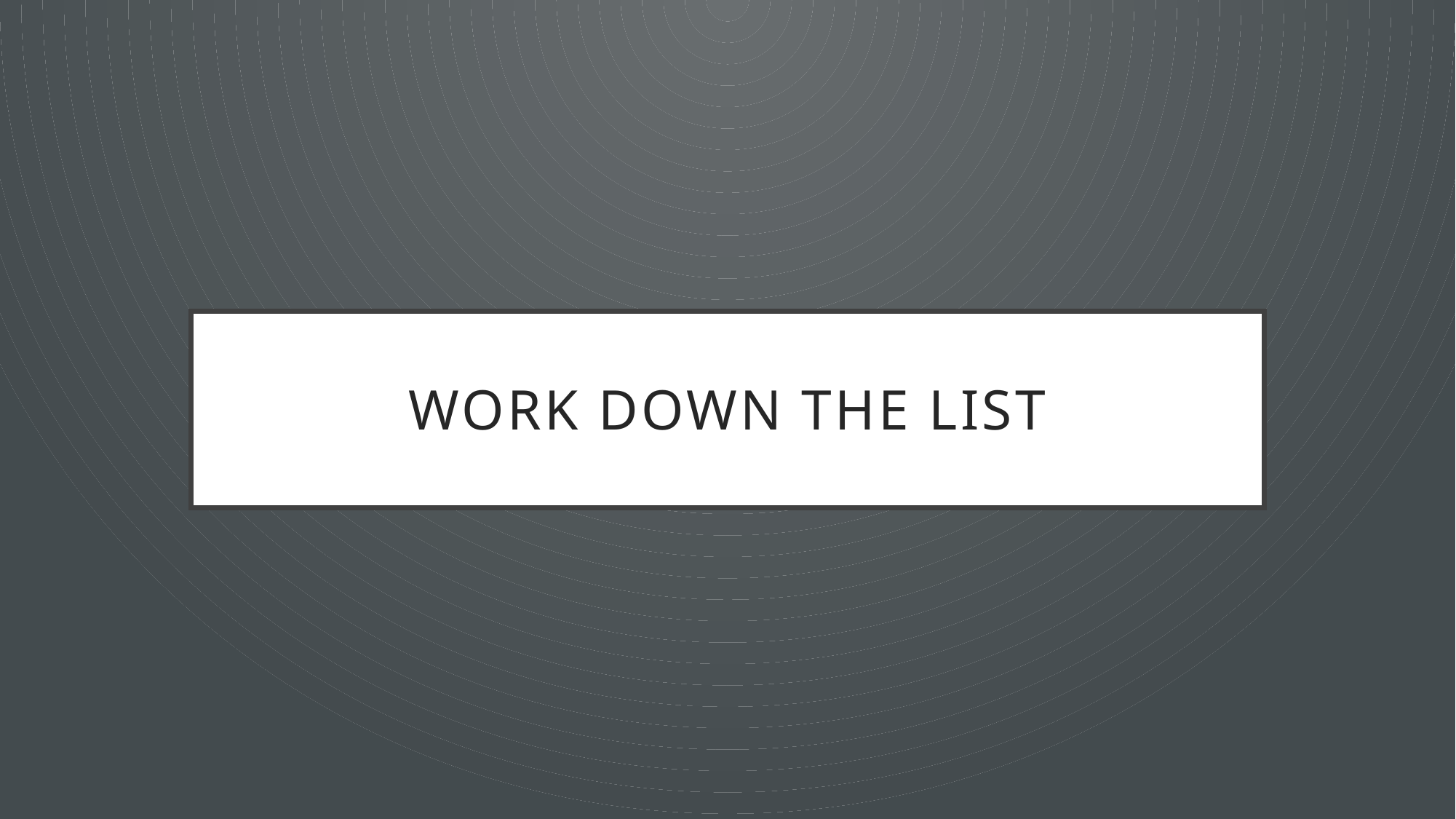

# Work down the list

## Slide 13
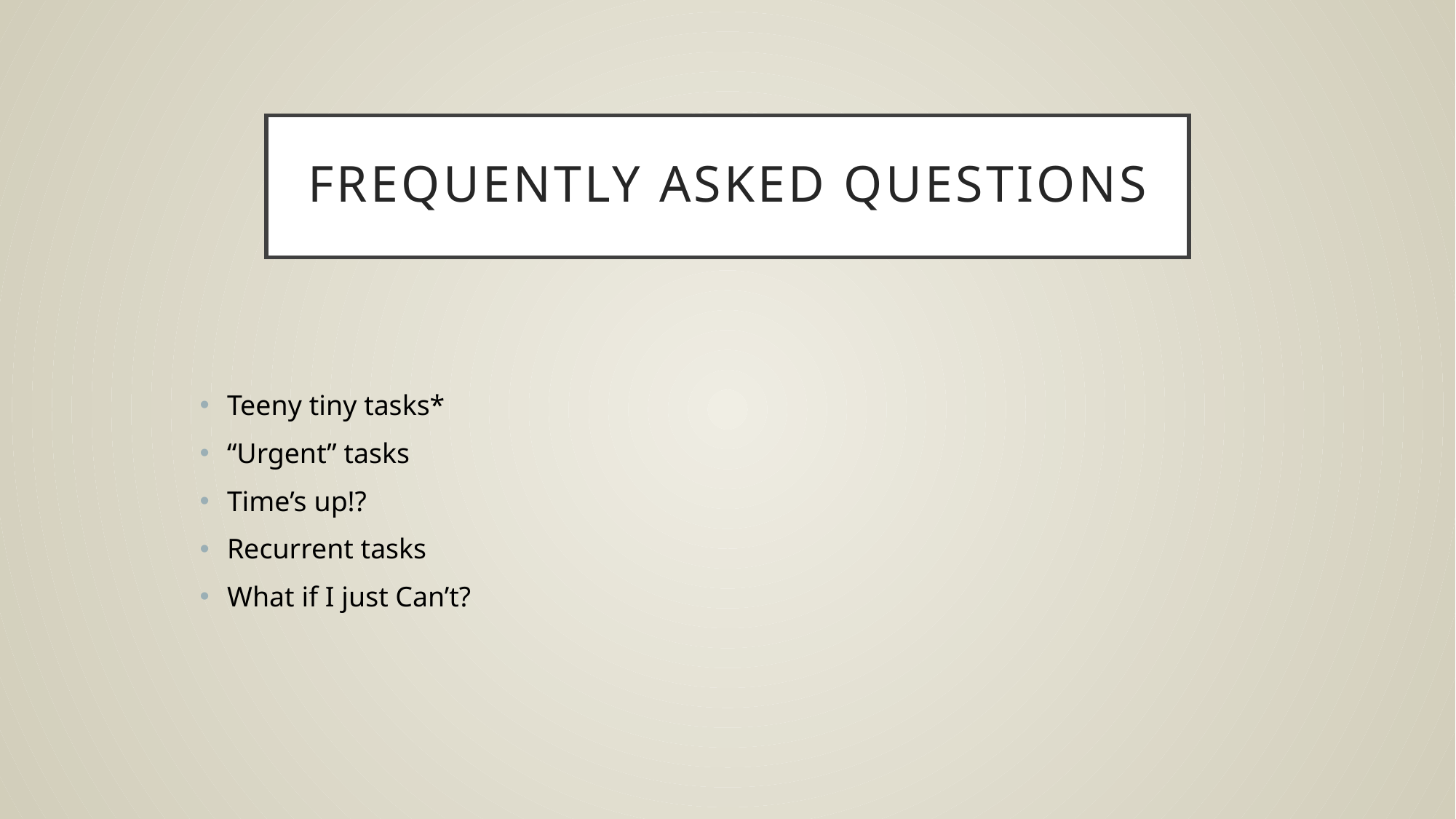

# Frequently asked questions
Teeny tiny tasks*
“Urgent” tasks
Time’s up!?
Recurrent tasks
What if I just Can’t?

## Slide 14
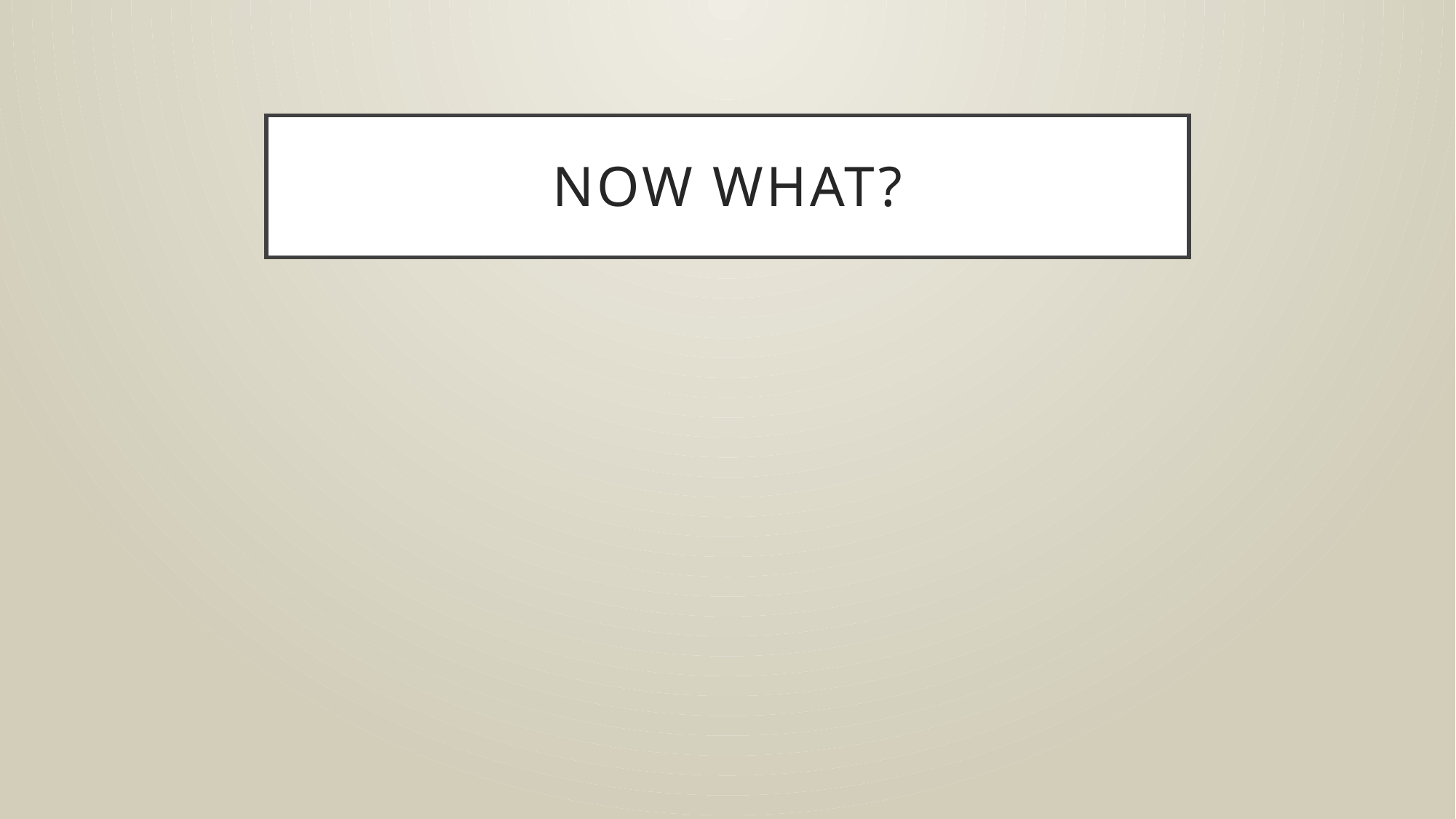

# Now what?

## Slide 15
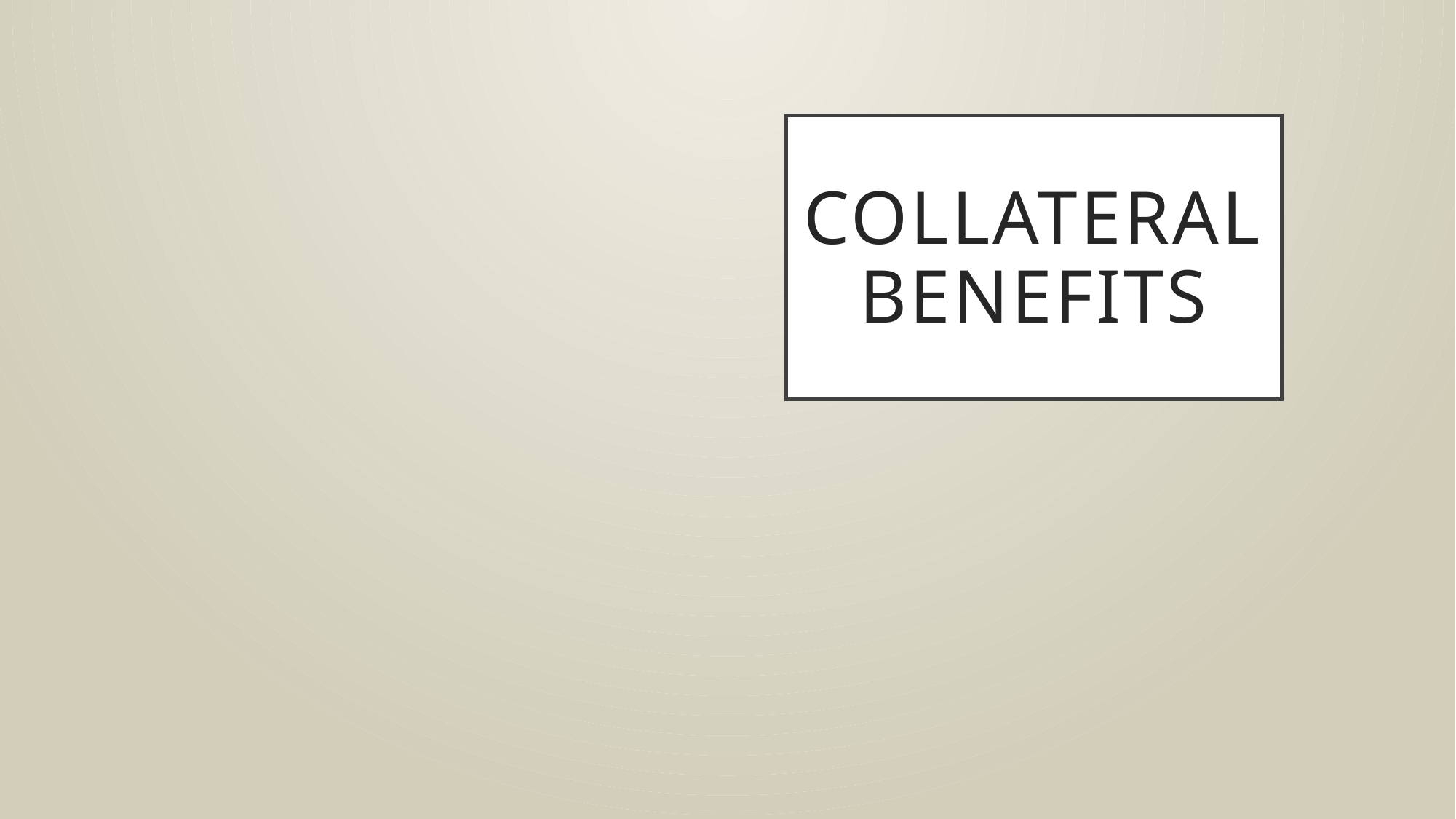

# Collateral benefits

## Slide 16
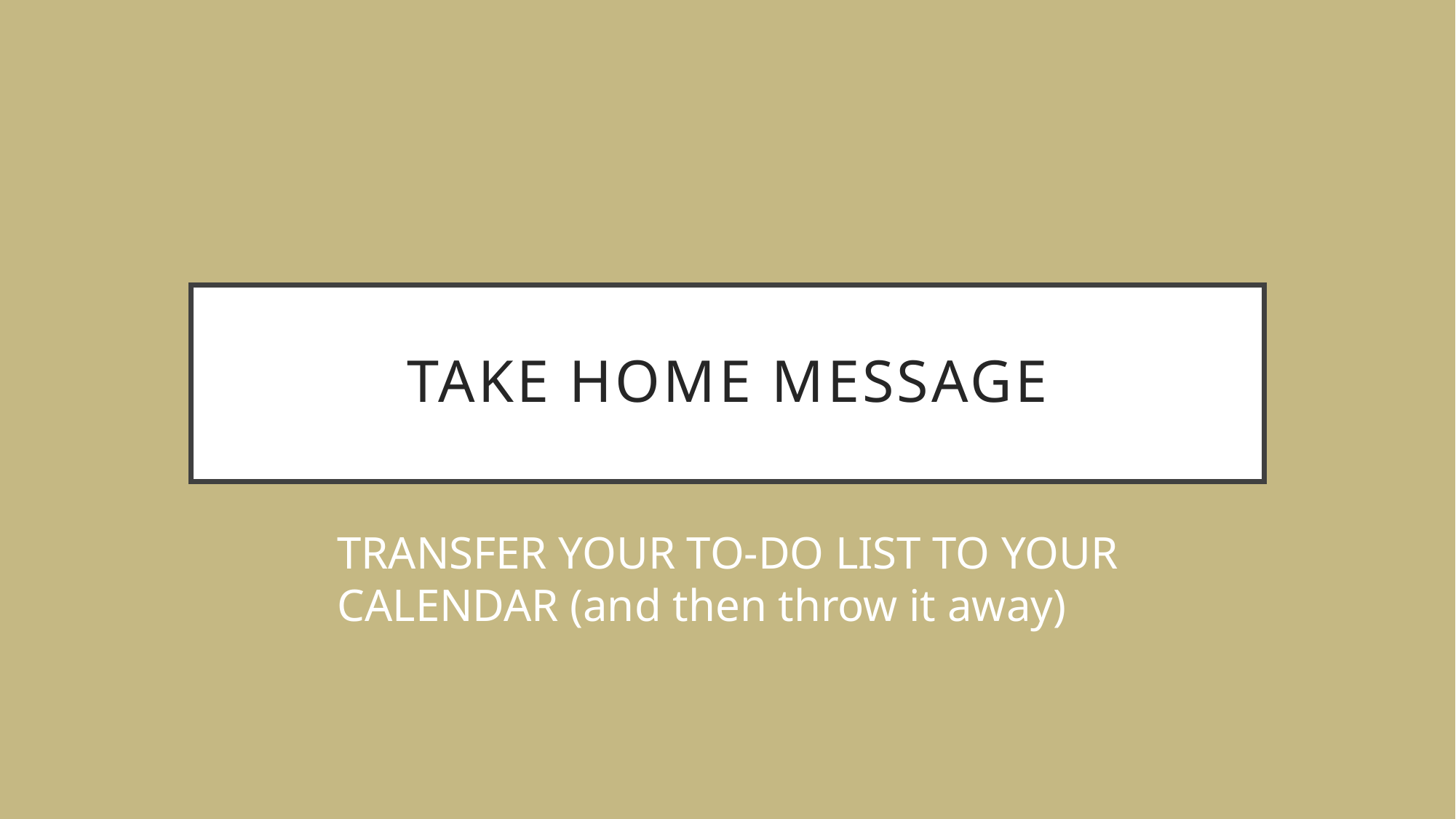

# Take home message
TRANSFER YOUR TO-DO LIST TO YOUR CALENDAR (and then throw it away)

## Slide 17
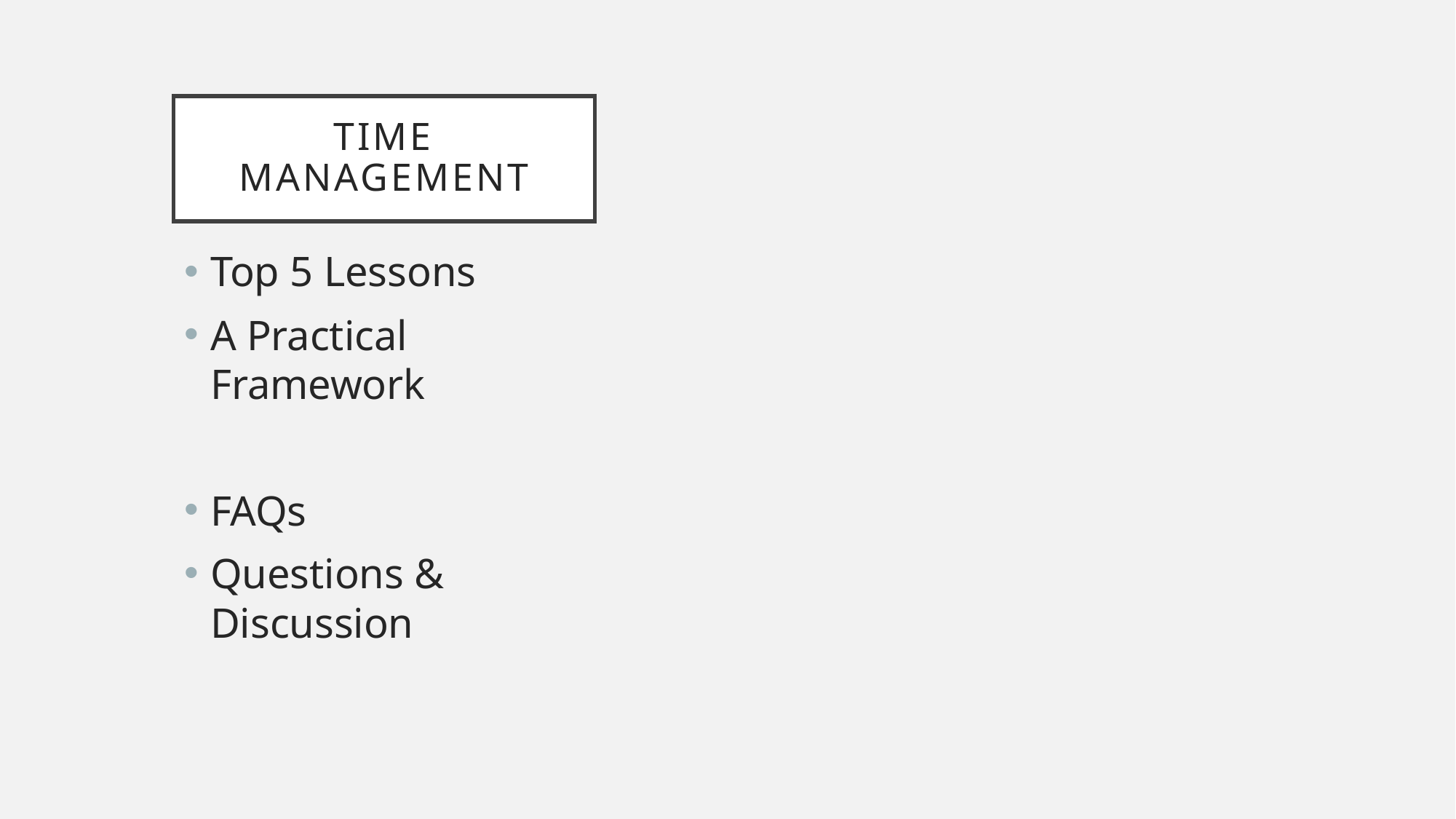

# Time management
Top 5 Lessons
A Practical Framework
FAQs
Questions & Discussion

## Slide 18
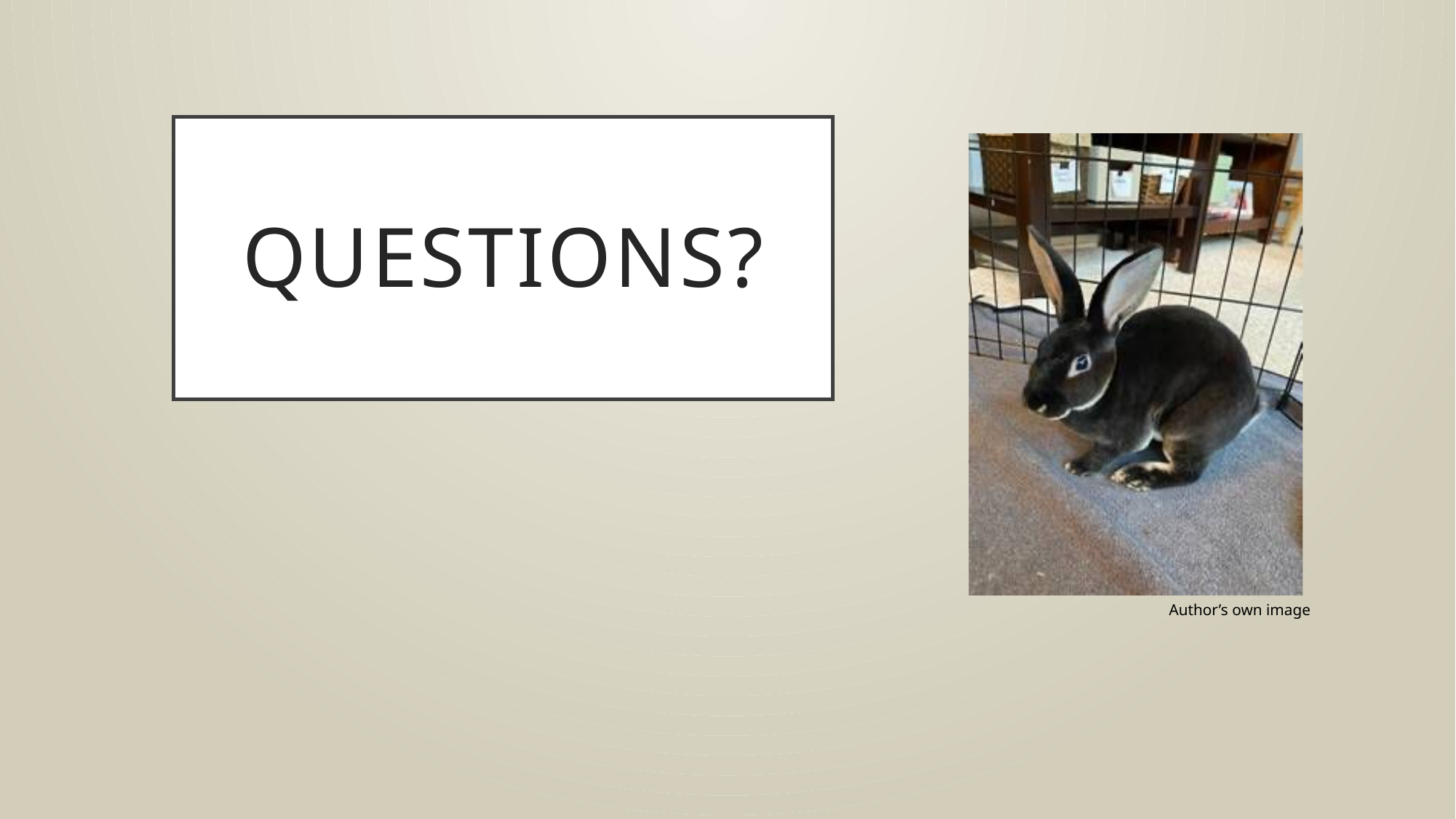

# Questions?
Author’s own image
