# Supplementary material for: What You Didn’t Learn in Residency: A Collective Curriculum for New Academic EM Faculty and Fellows
Source: J Educ Teach Emerg Med. 2024 Jan 31;9(1):C16–40. doi: 10.21980/J8WP9Z (PMC10854884; doi:10.21980/J8WP9Z)
Supplement: Supplementary file 2 — Please see associated Power Point [file jetem-9-1-C16-AppendixD.pptx]

## Slide 1
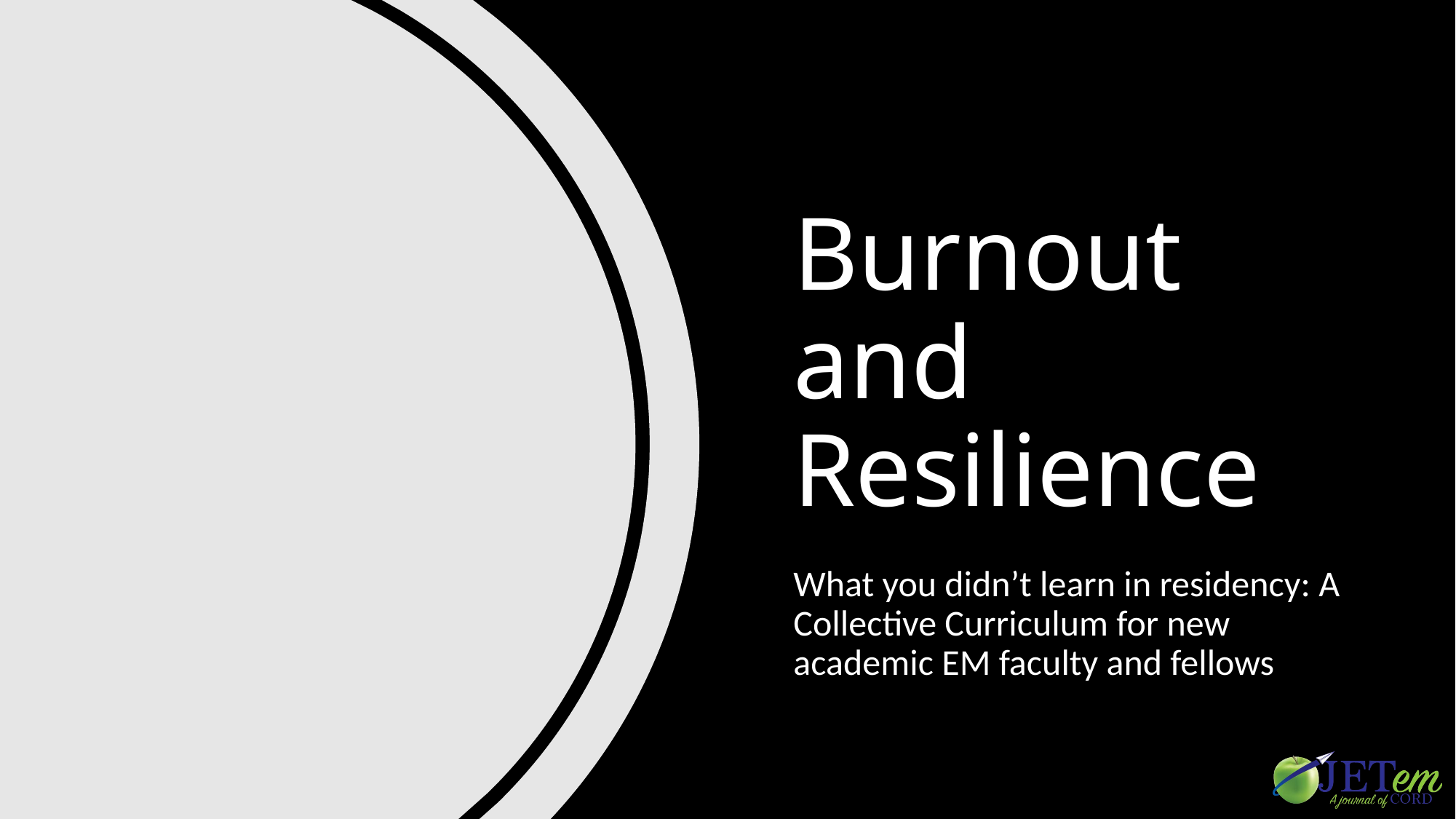

# Burnout and Resilience
What you didn’t learn in residency: A Collective Curriculum for new academic EM faculty and fellows

## Slide 2
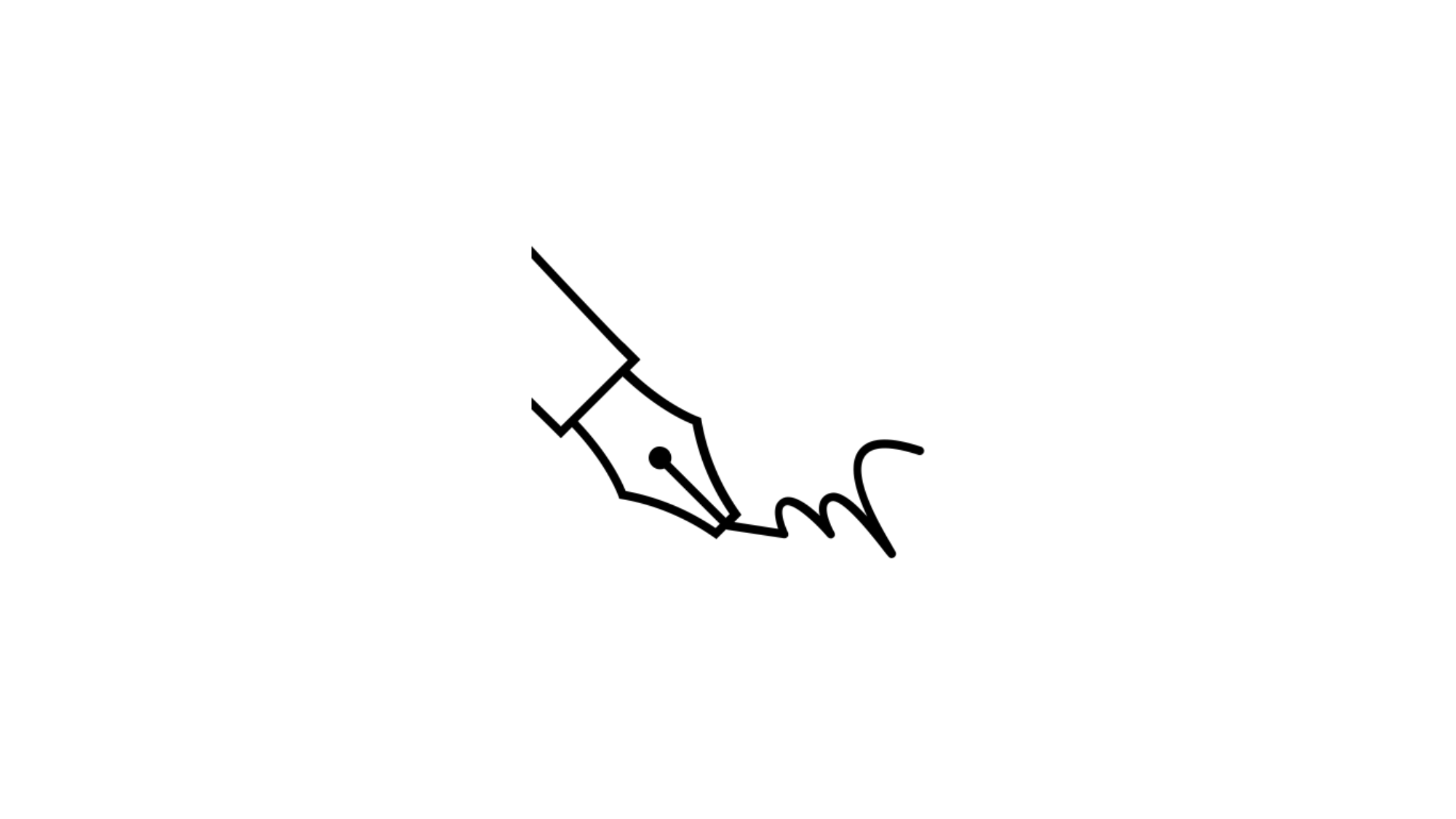

## Slide 3
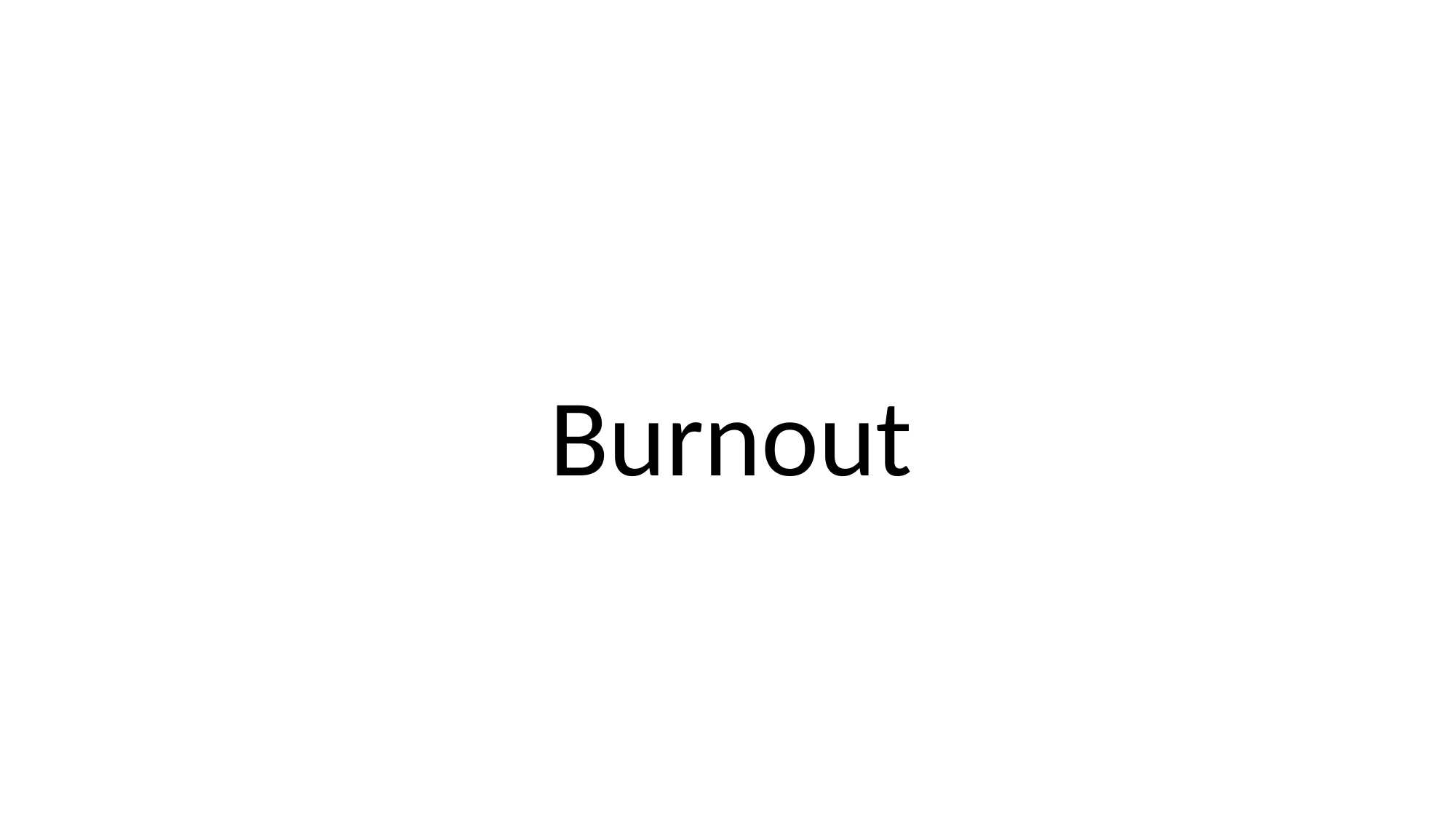

Burnout

## Slide 4
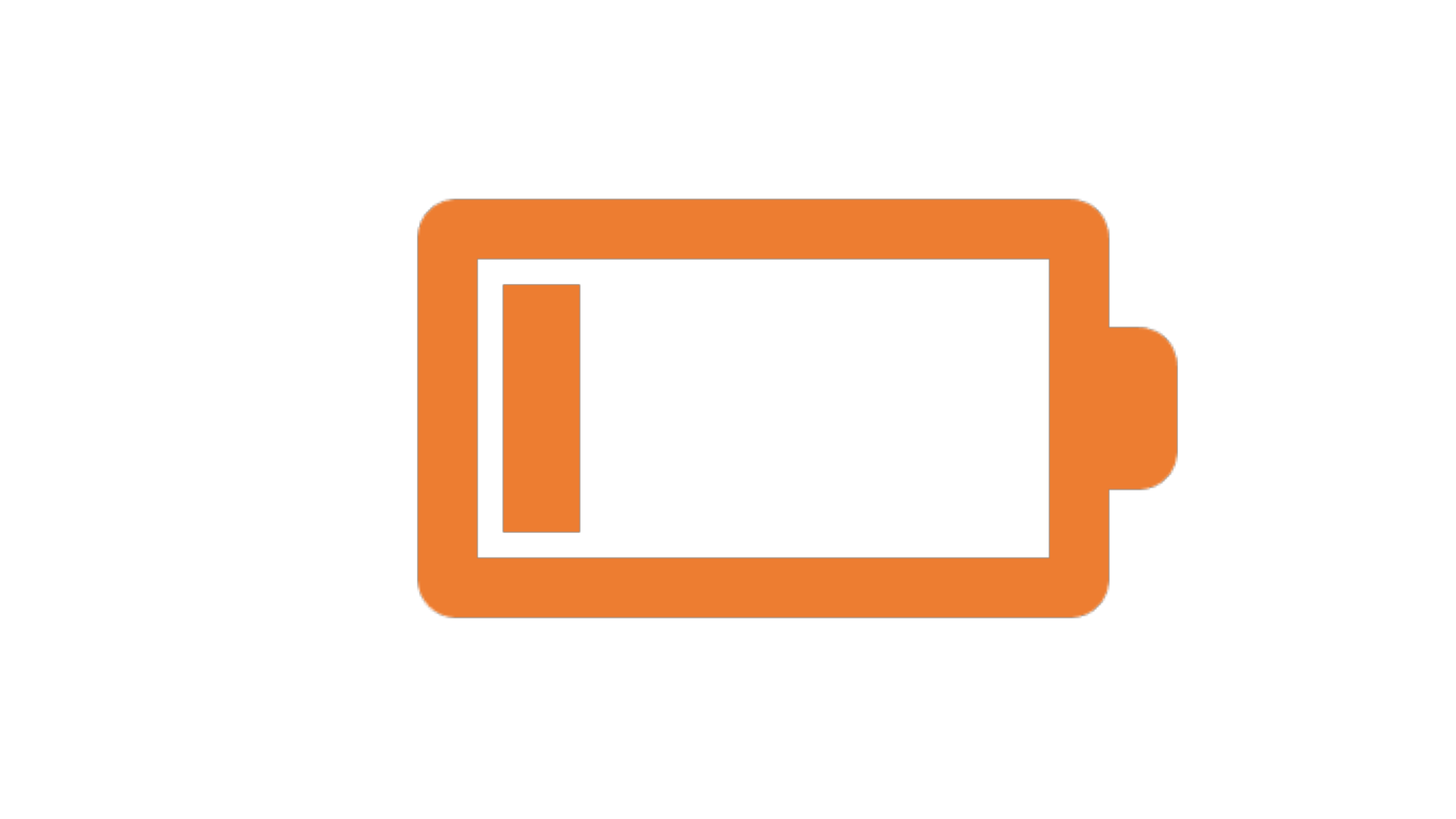

## Slide 5
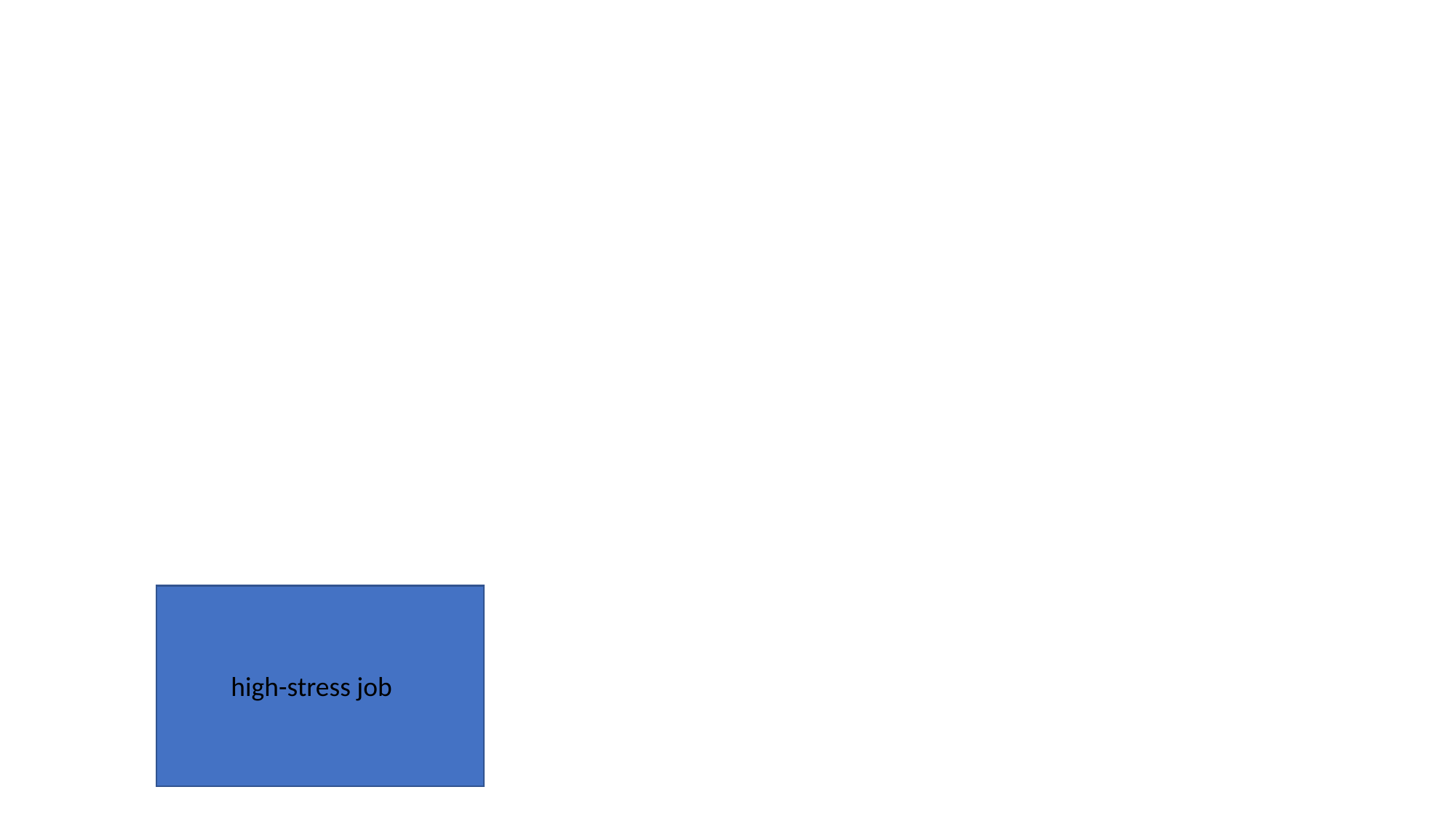

high-stress job

## Slide 6
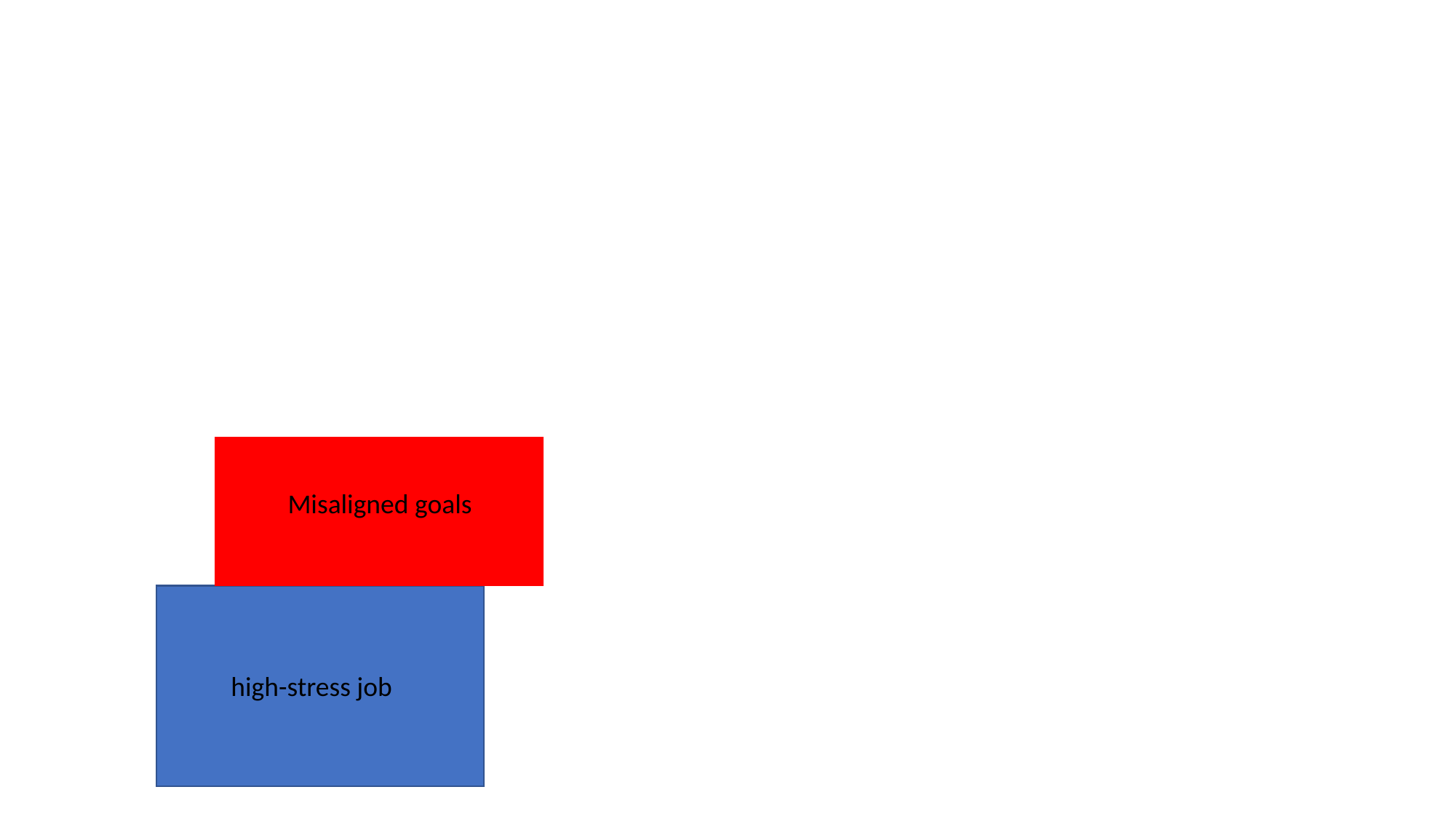

Misaligned goals
high-stress job

## Slide 7
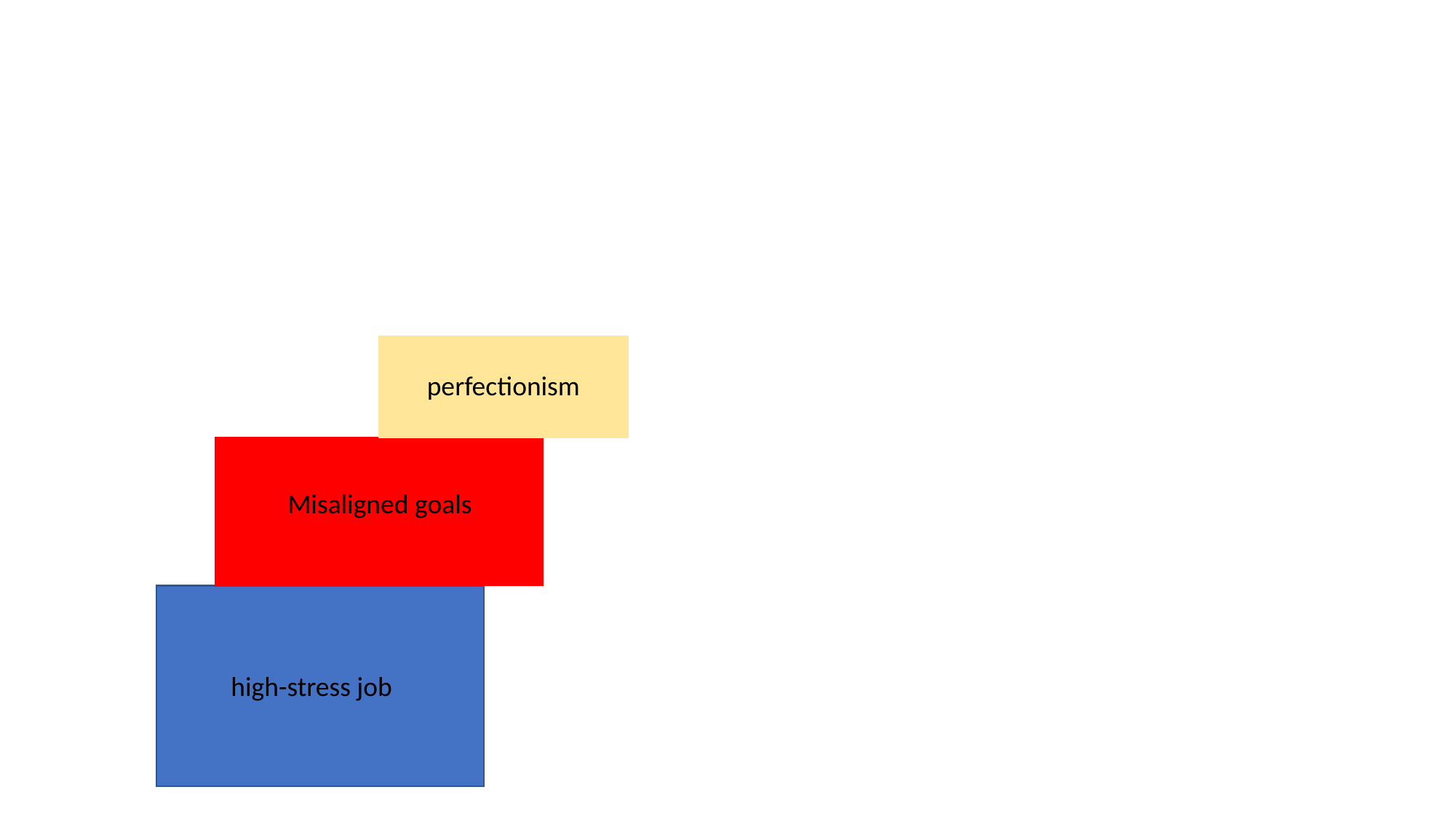

perfectionism
Misaligned goals
high-stress job

## Slide 8
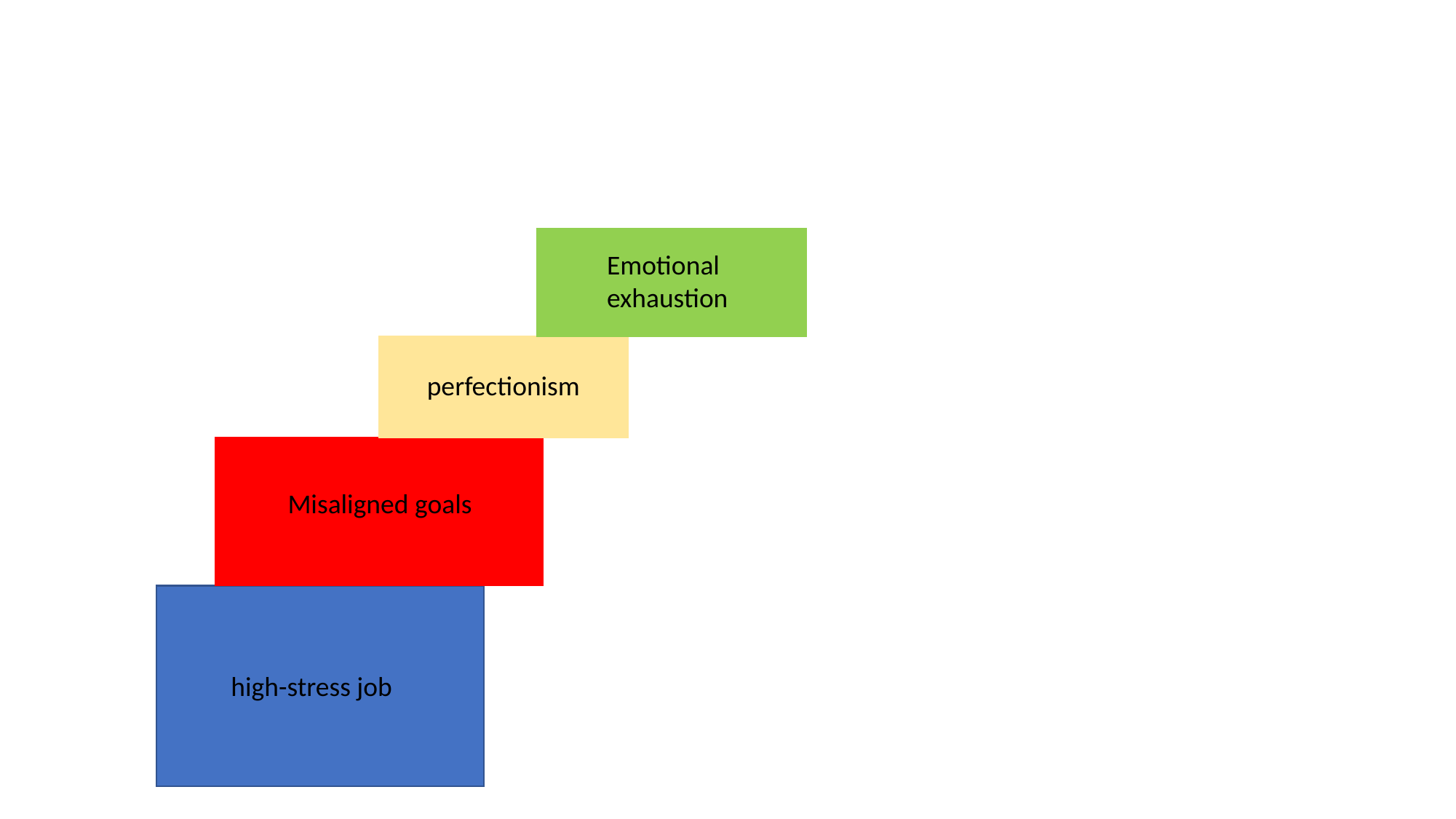

Emotional
exhaustion
perfectionism
Misaligned goals
high-stress job

## Slide 9
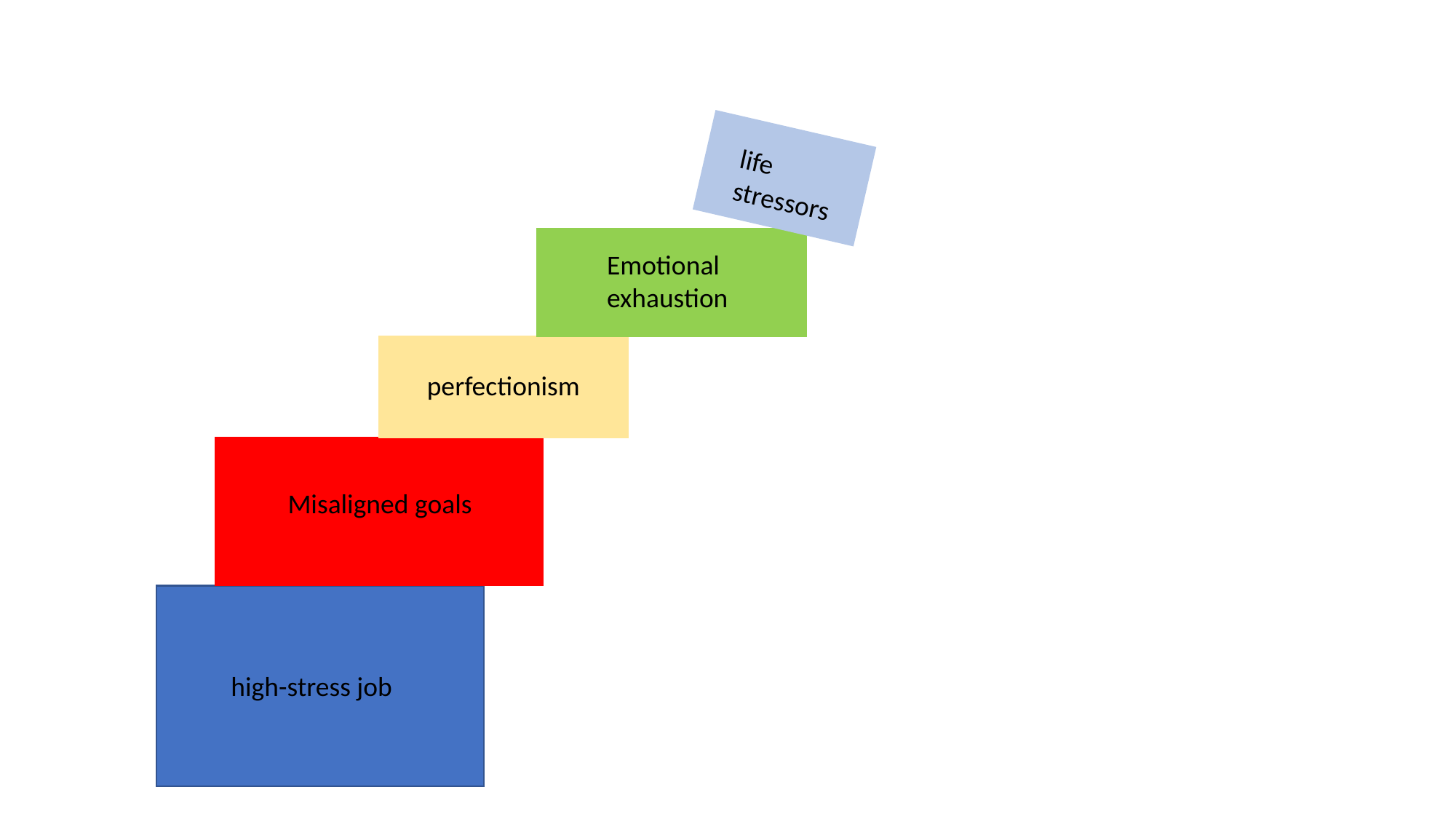

life
stressors
Emotional
exhaustion
perfectionism
Misaligned goals
high-stress job

## Slide 10
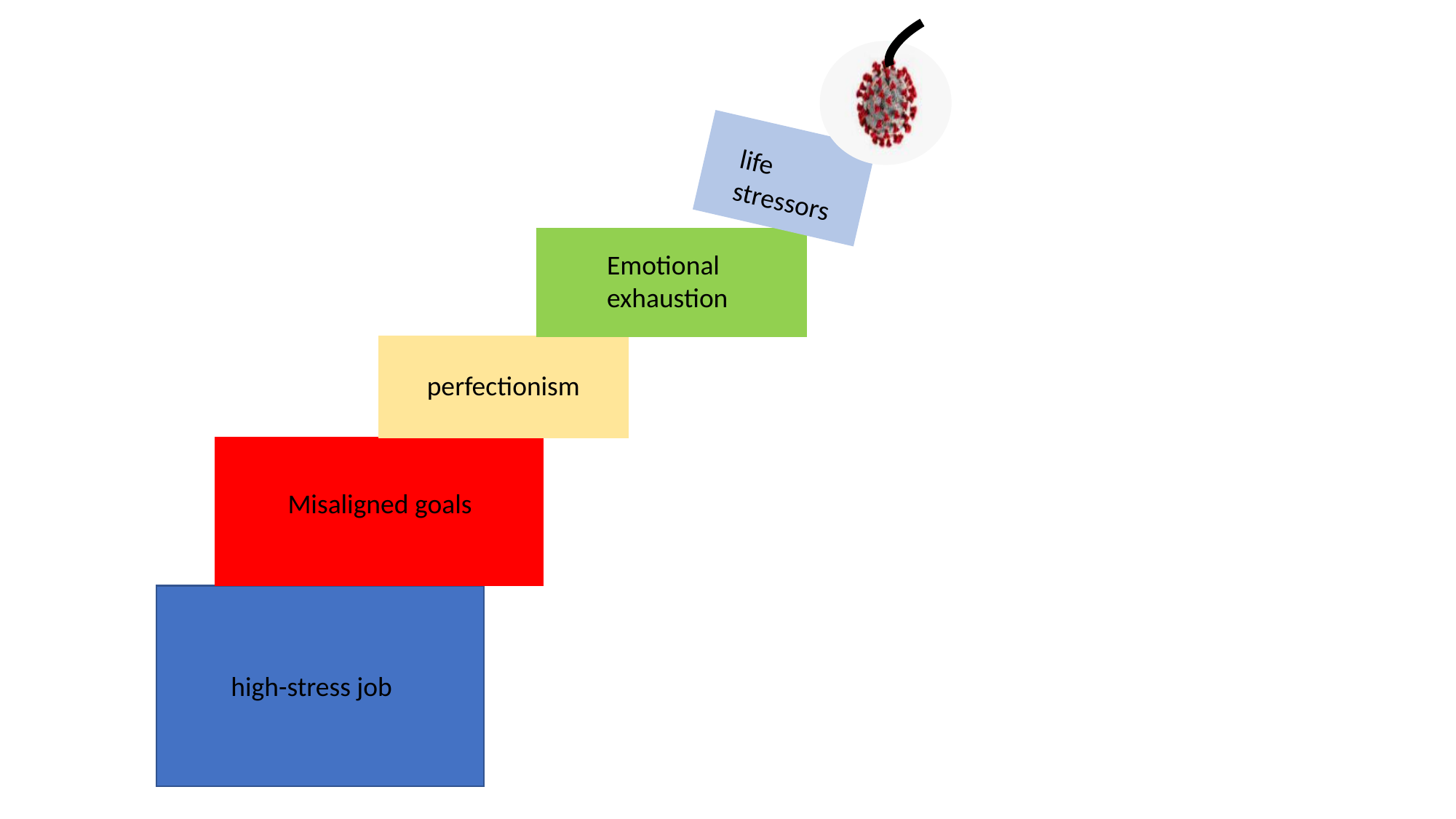

life
stressors
Emotional
exhaustion
perfectionism
Misaligned goals
high-stress job

## Slide 11
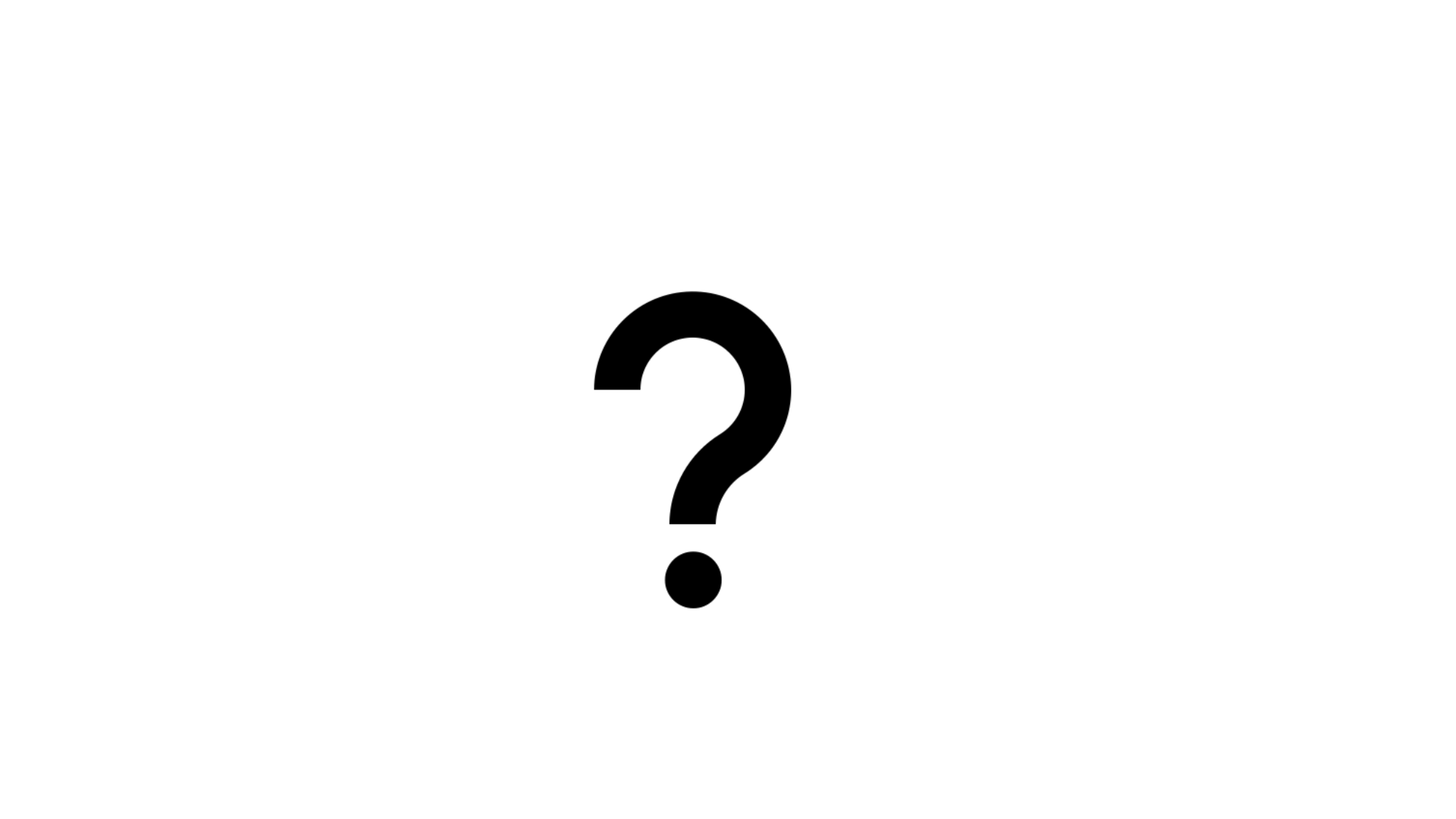

## Slide 12
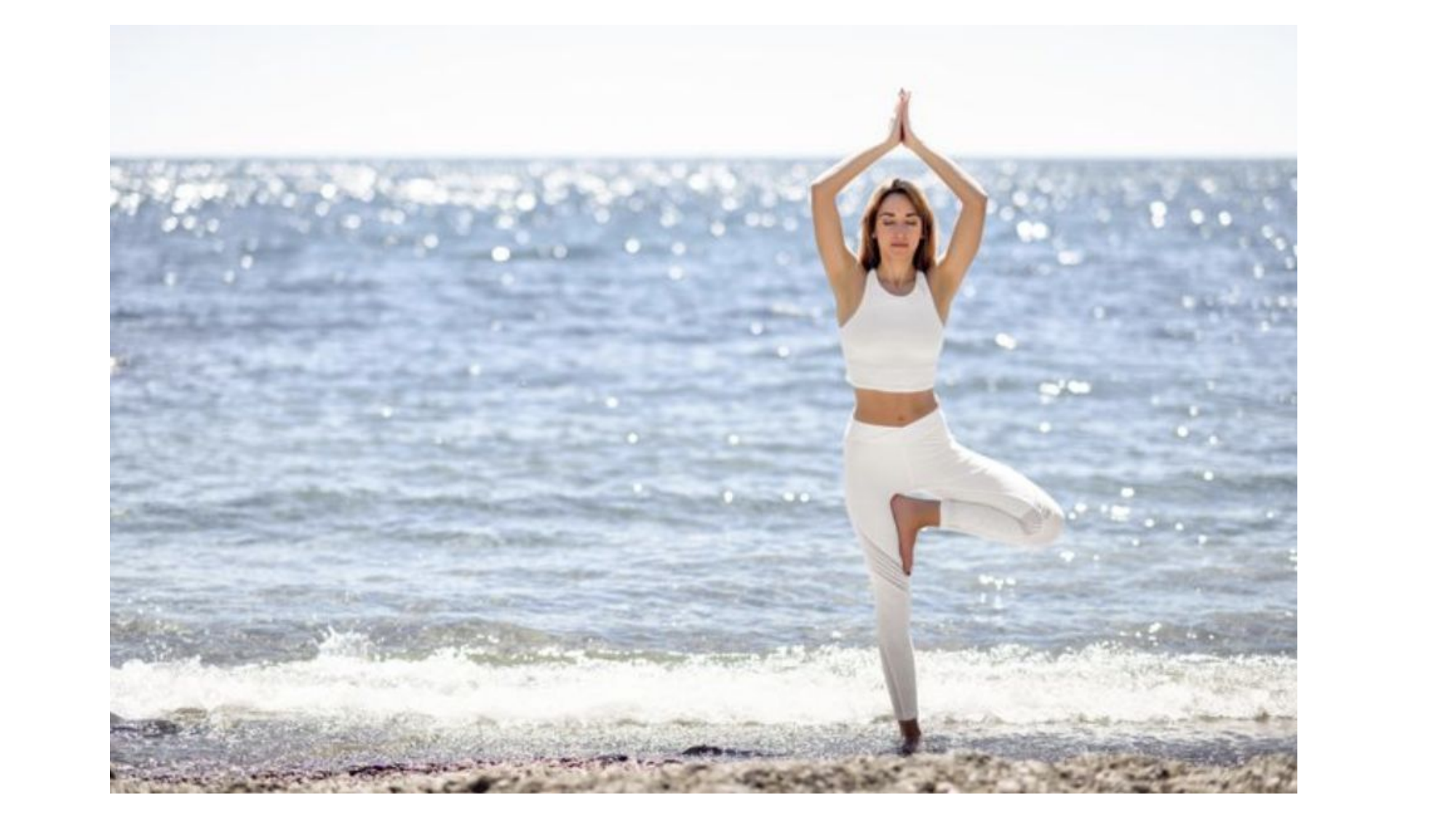

## Slide 13
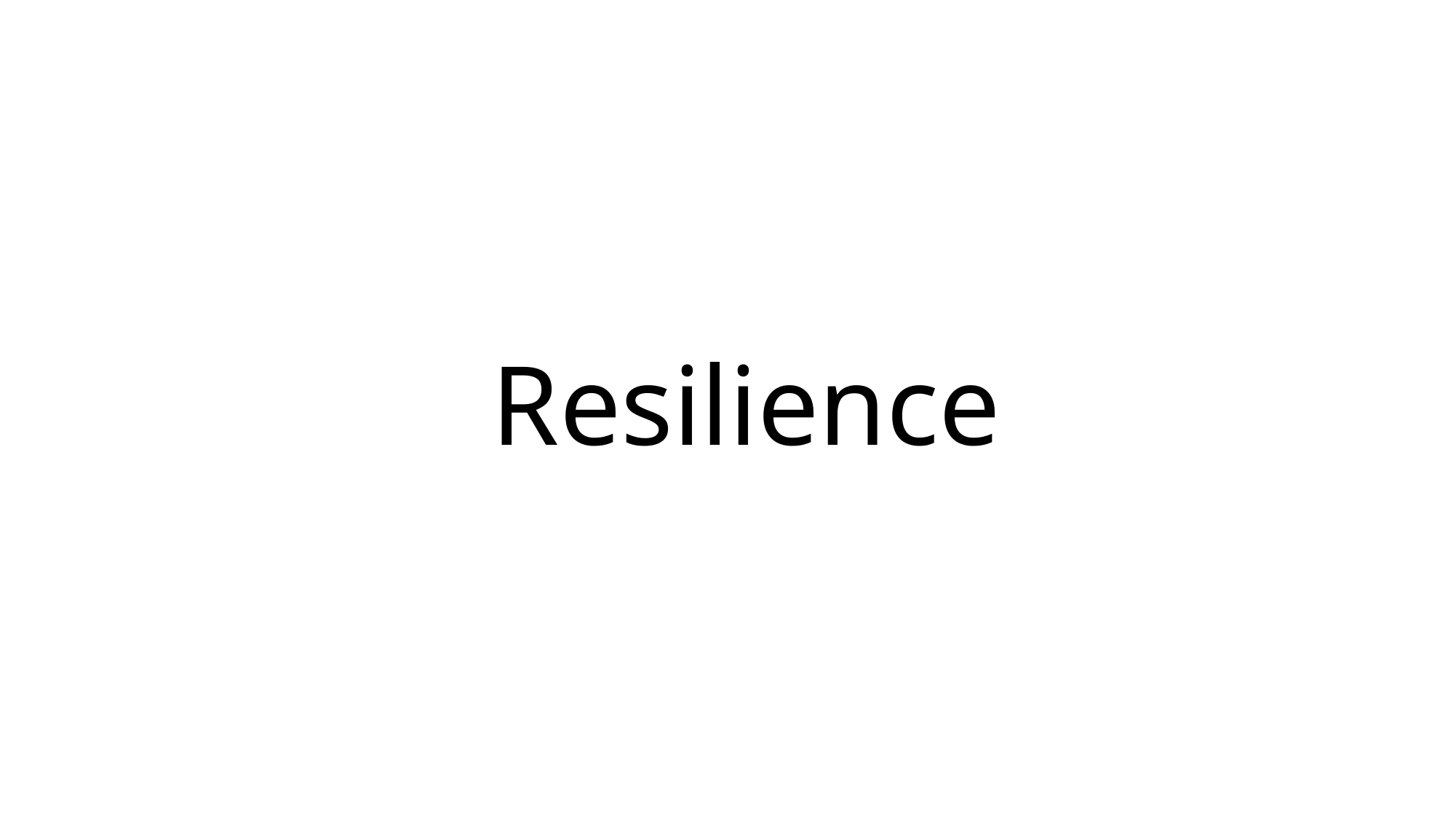

# Resilience

## Slide 14
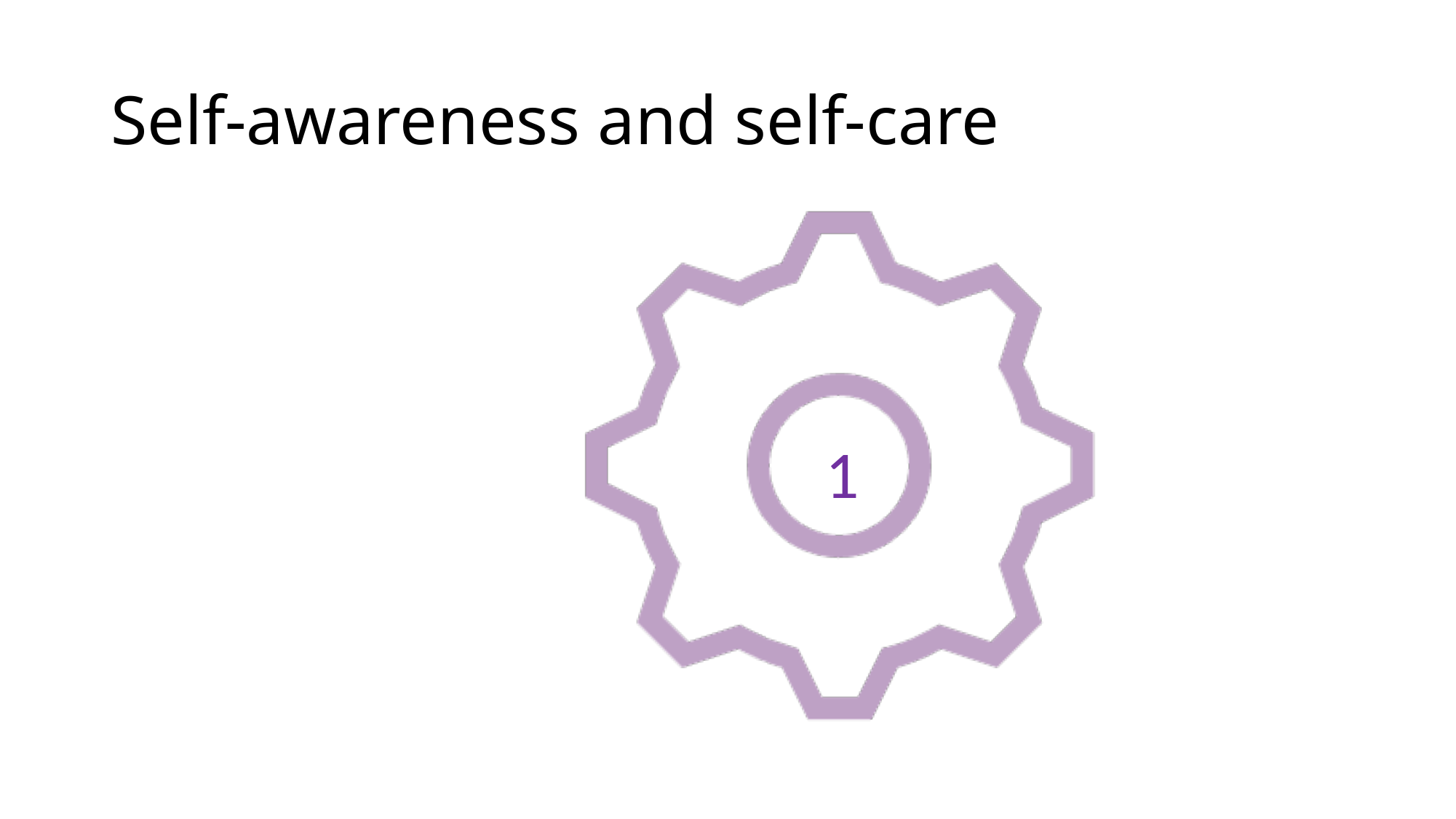

# Self-awareness and self-care
1

## Slide 15
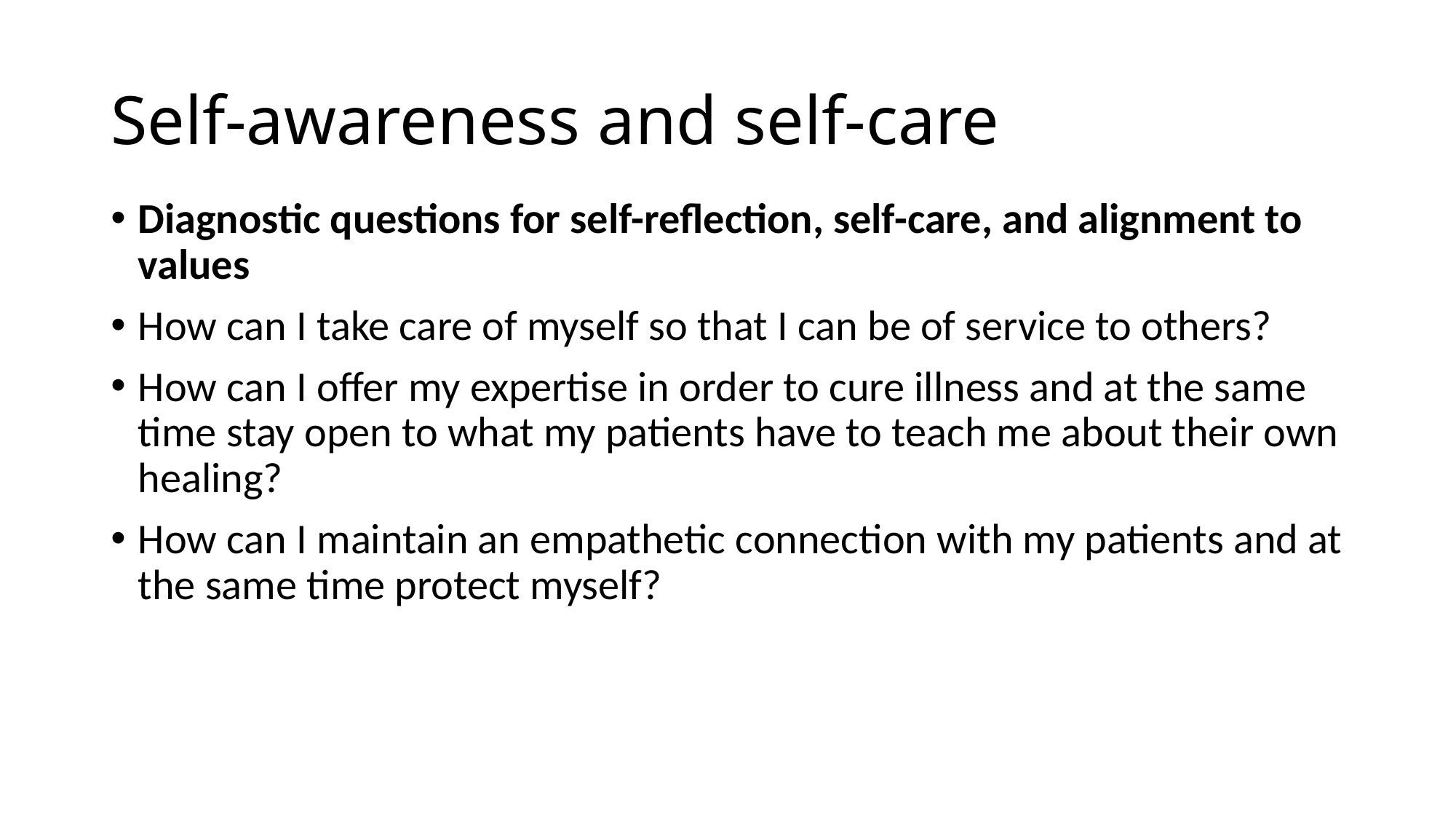

# Self-awareness and self-care
Diagnostic questions for self-reflection, self-care, and alignment to values
How can I take care of myself so that I can be of service to others?
How can I offer my expertise in order to cure illness and at the same time stay open to what my patients have to teach me about their own healing?
How can I maintain an empathetic connection with my patients and at the same time protect myself?

## Slide 16
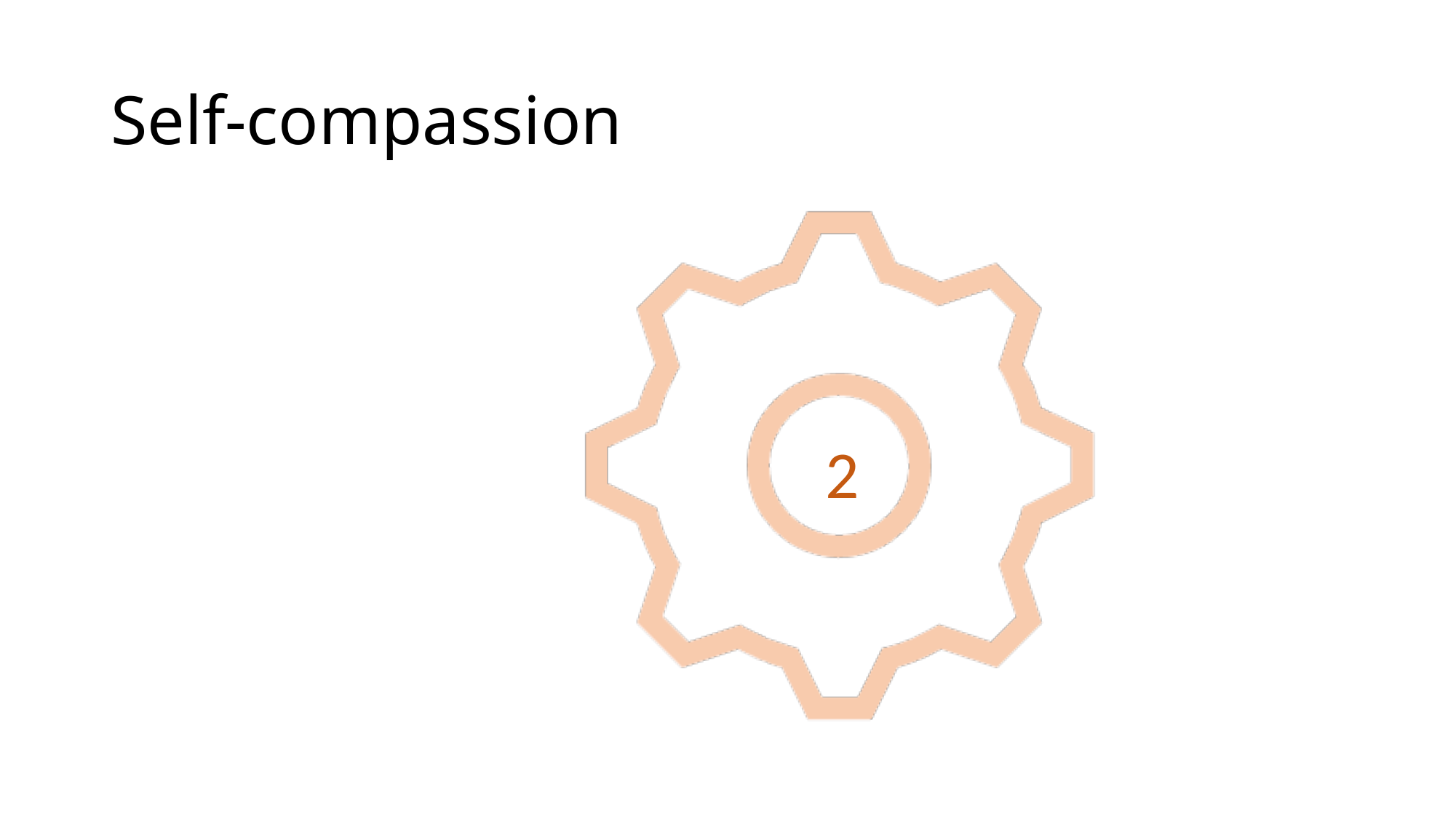

# Self-compassion
2

## Slide 17
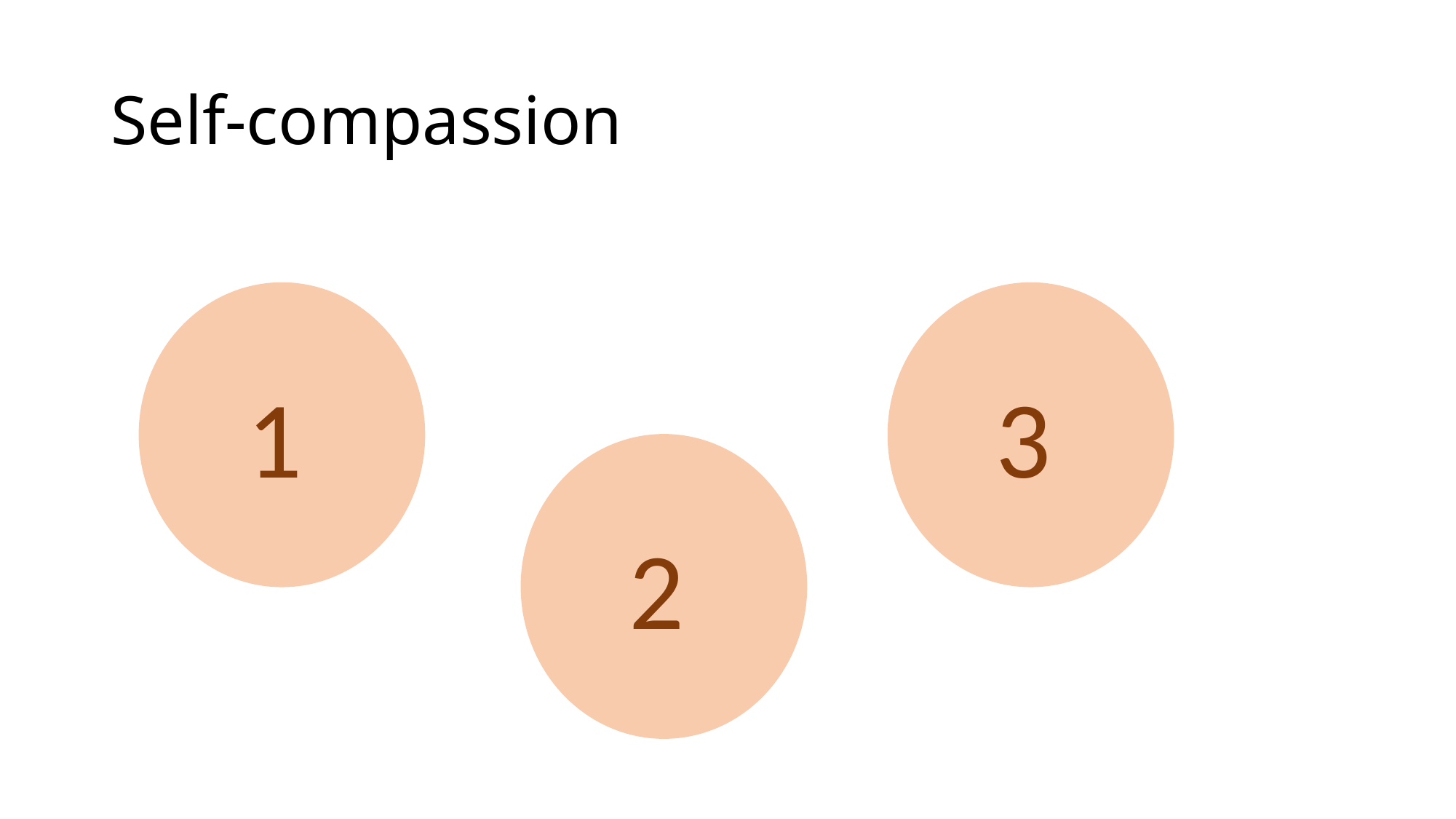

# Self-compassion
1
3
2

## Slide 18
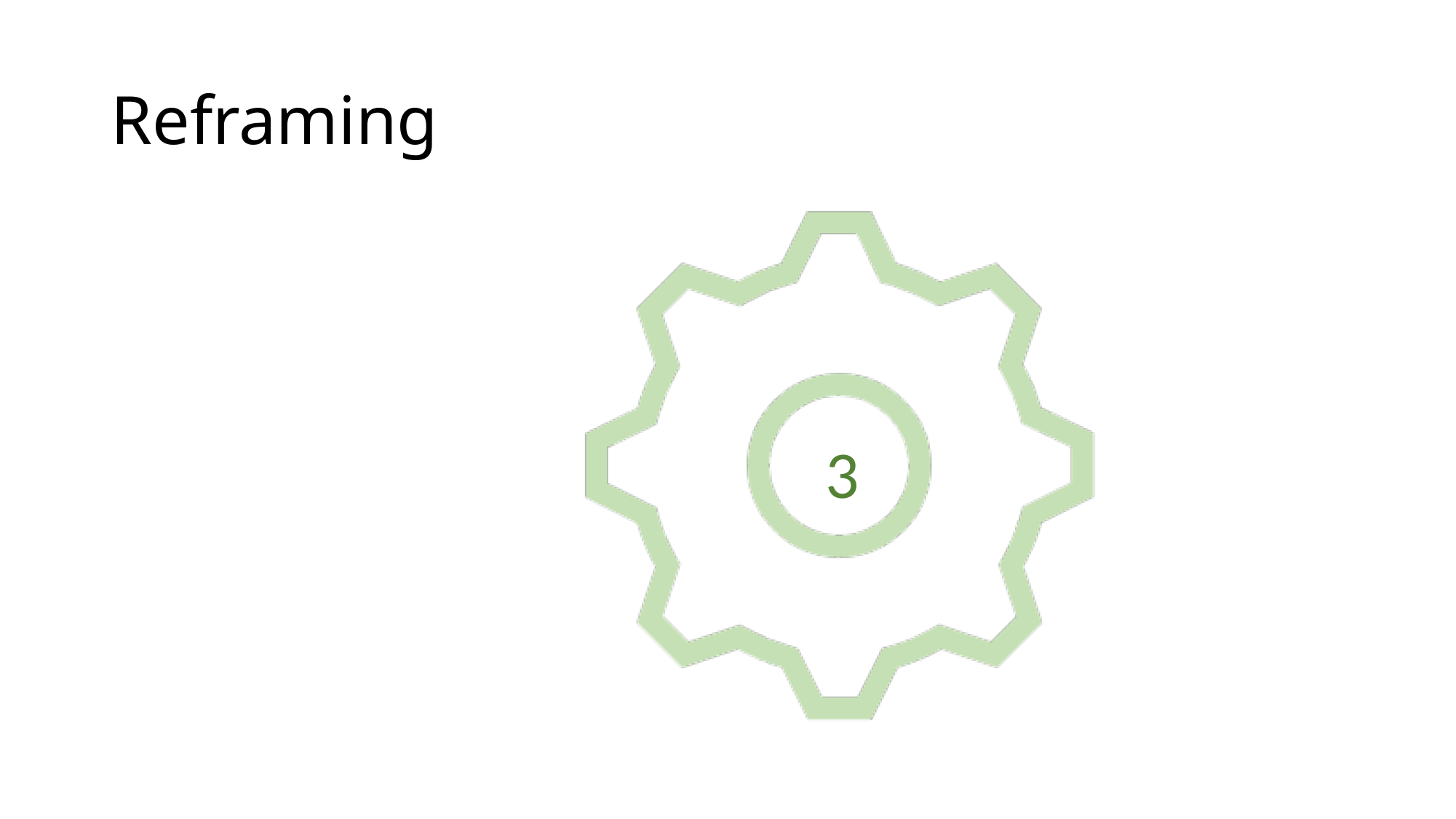

# Reframing
3

## Slide 19
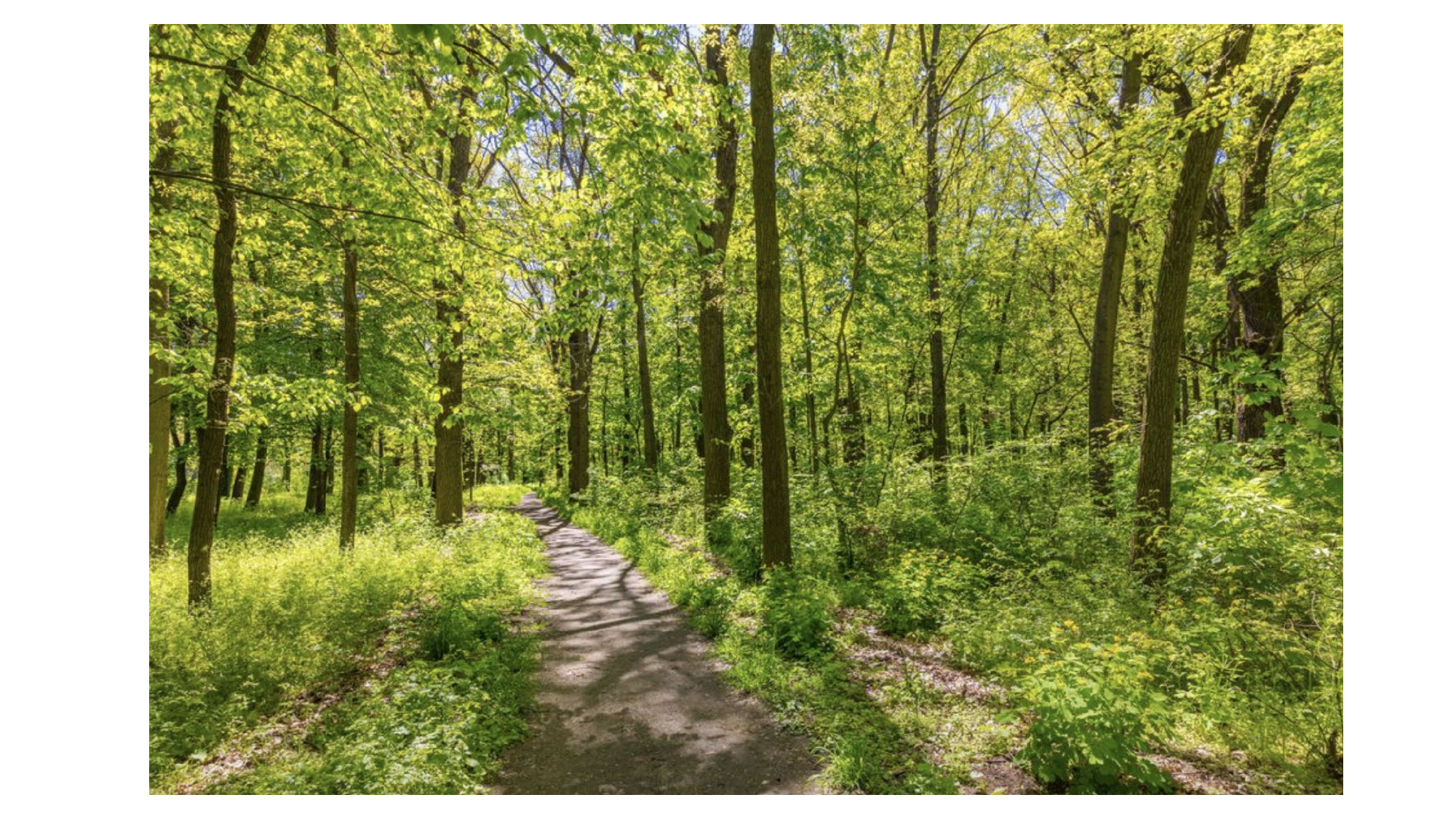

## Slide 20
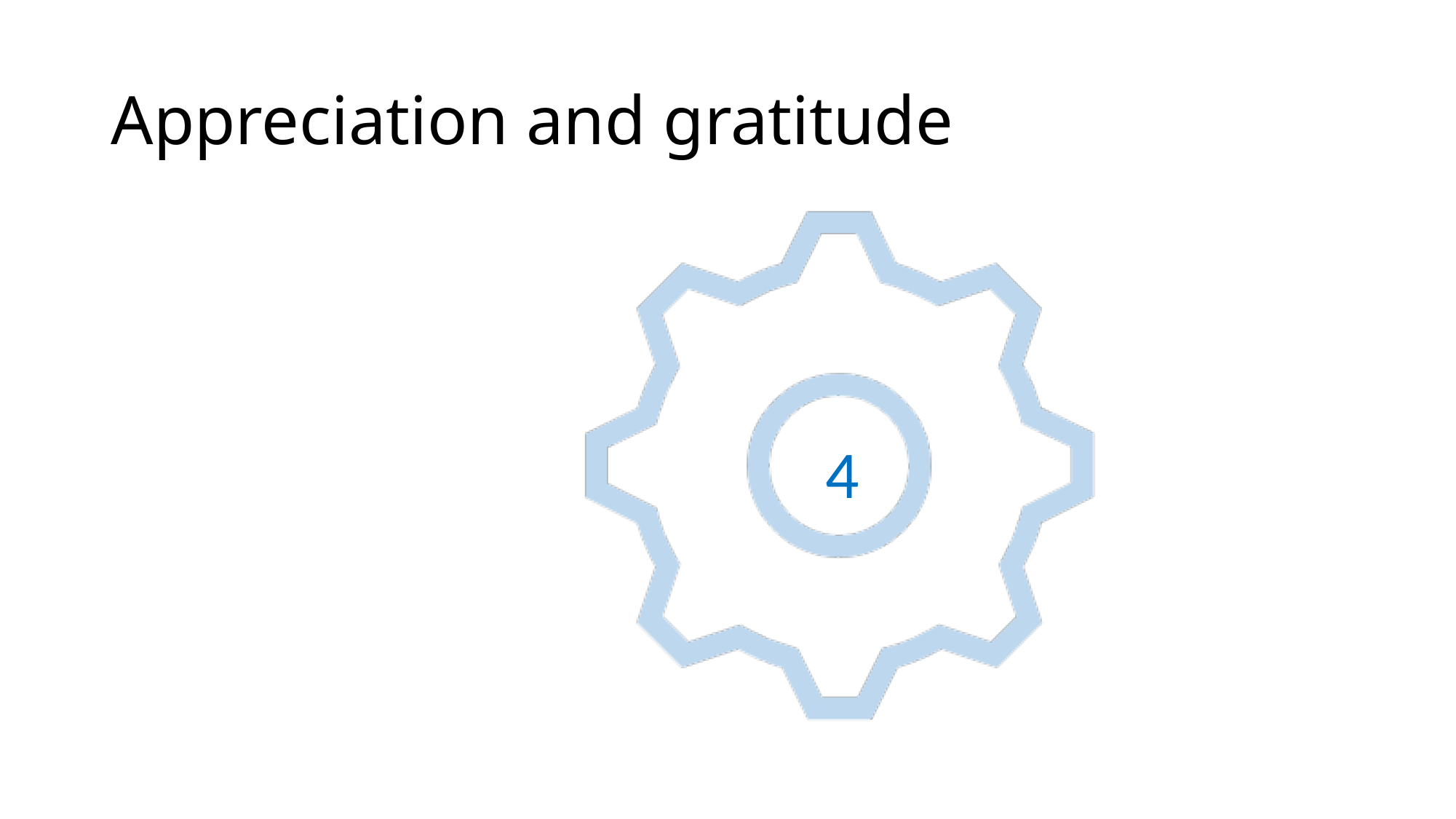

# Appreciation and gratitude
4

## Slide 21
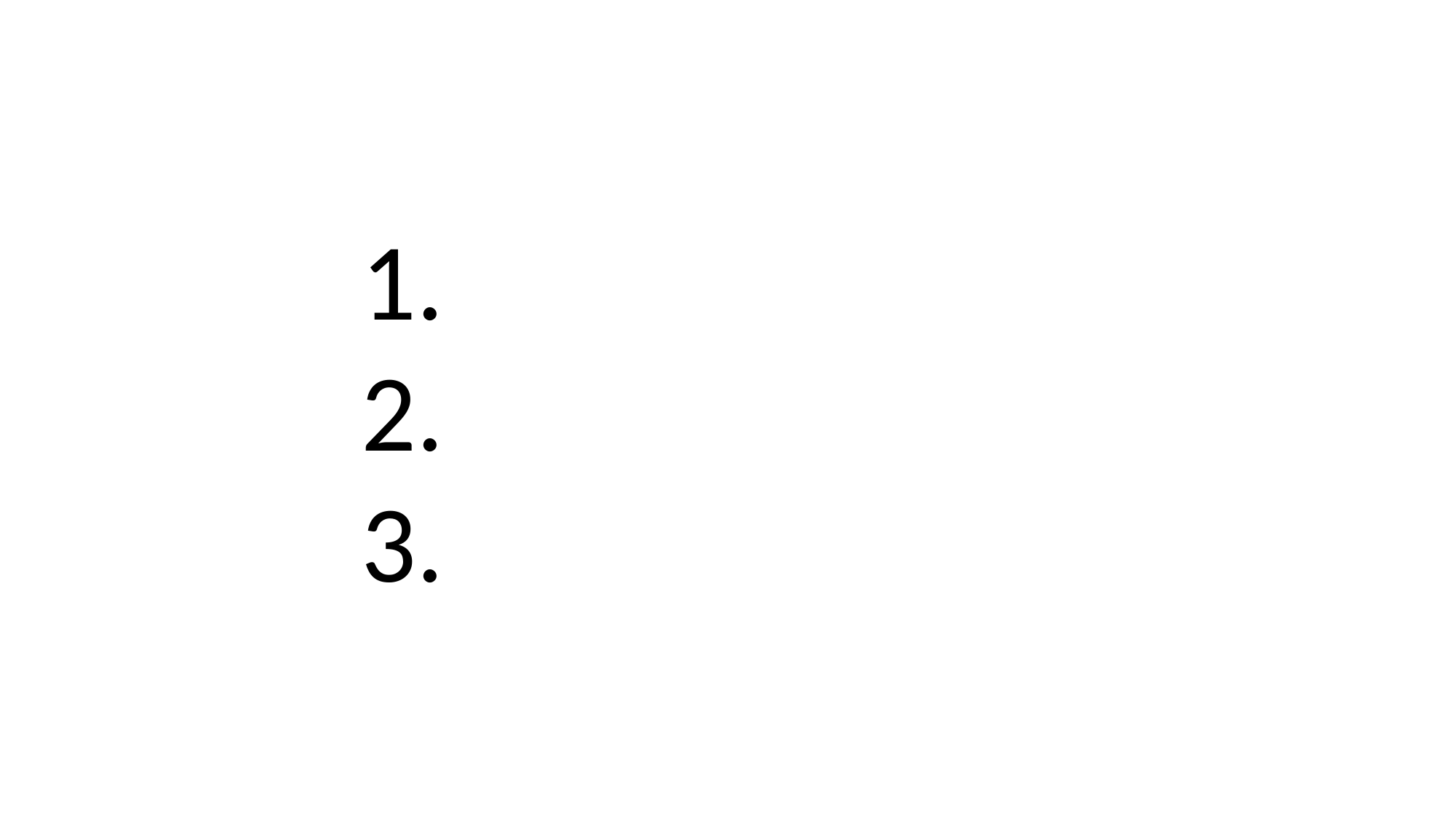

1.
2.
3.

## Slide 22
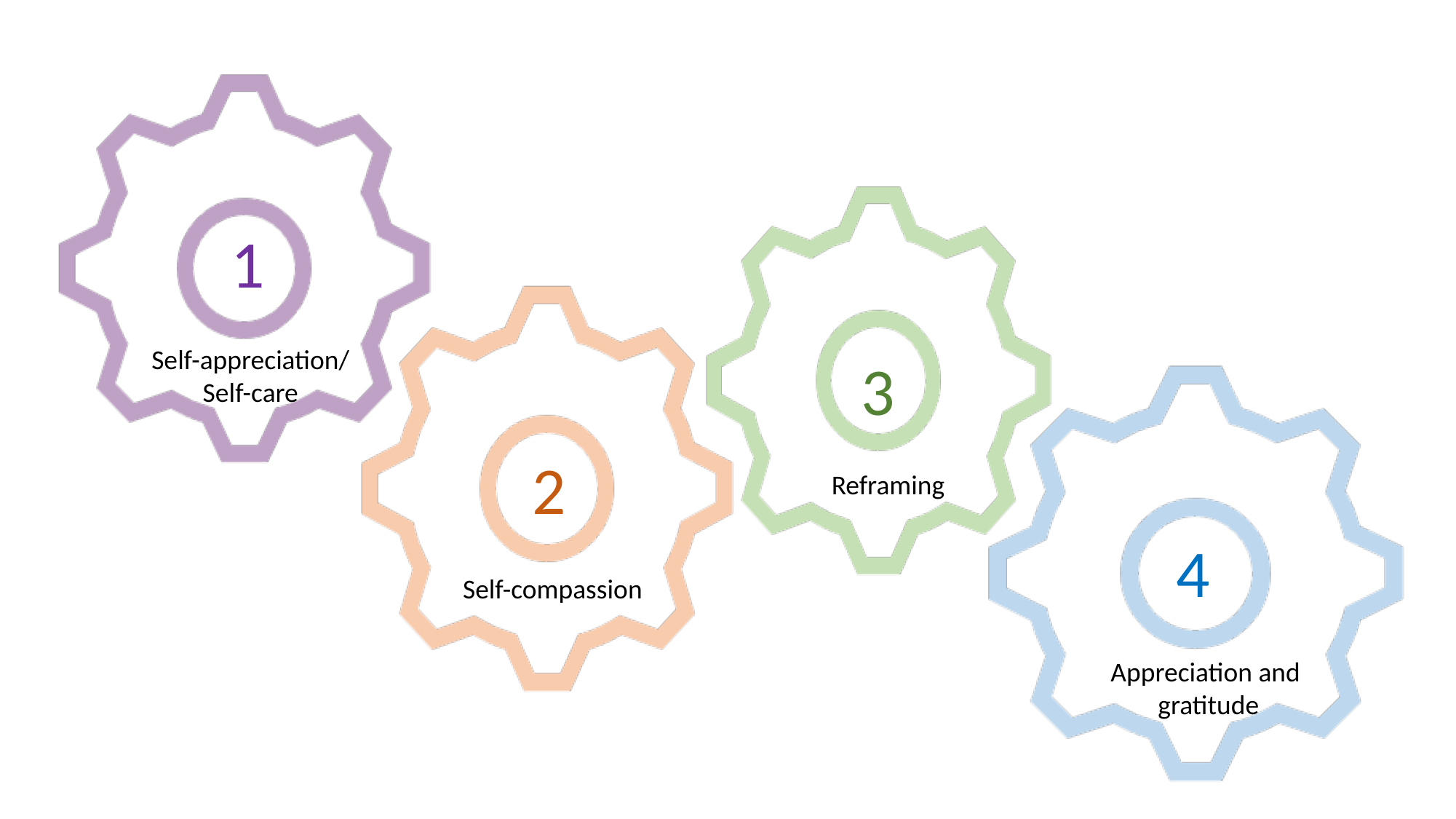

1
3
2
4
Self-appreciation/
Self-care
Reframing
Self-compassion
Appreciation and
gratitude

## Slide 23
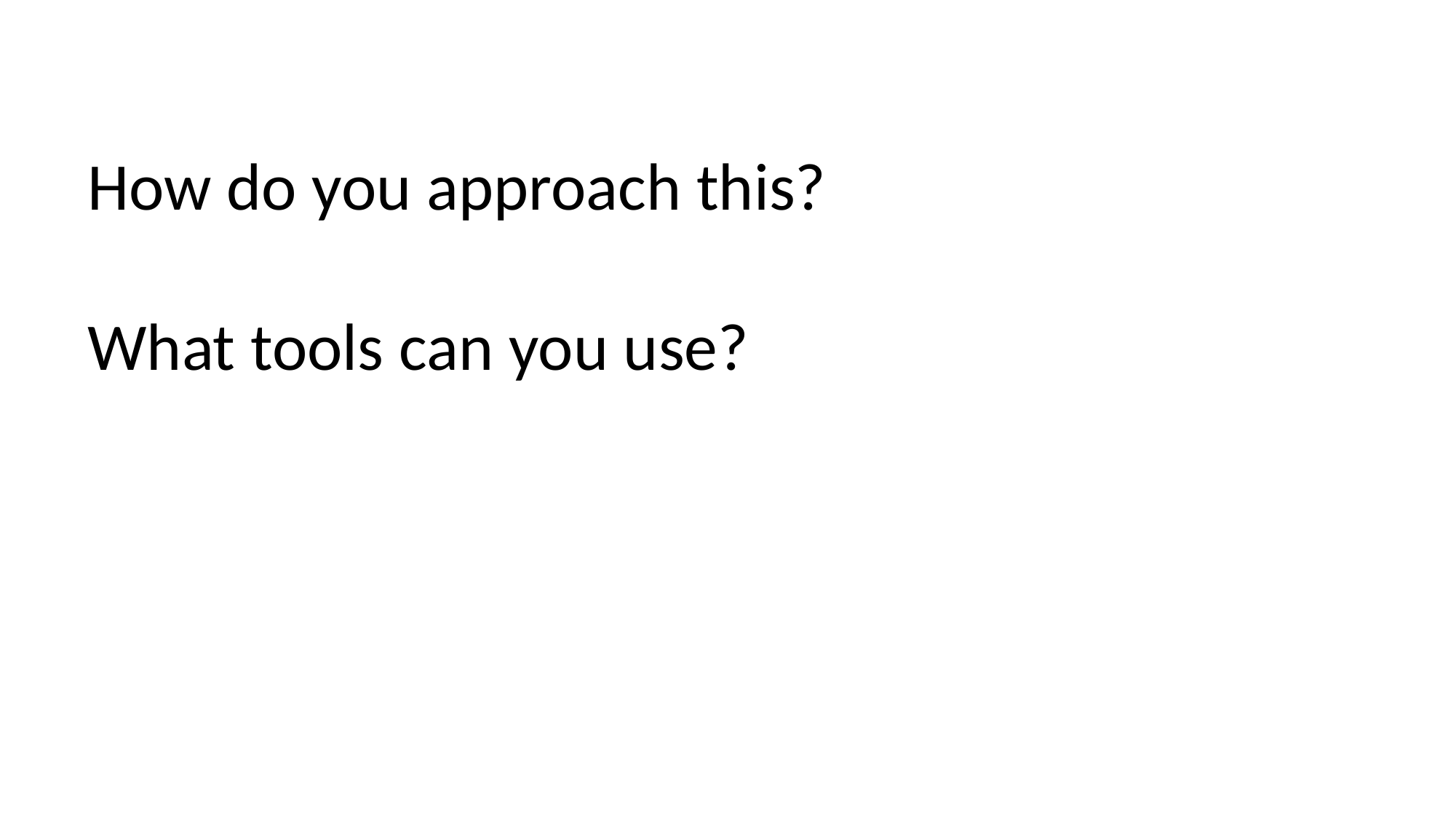

How do you approach this?
What tools can you use?

## Slide 24
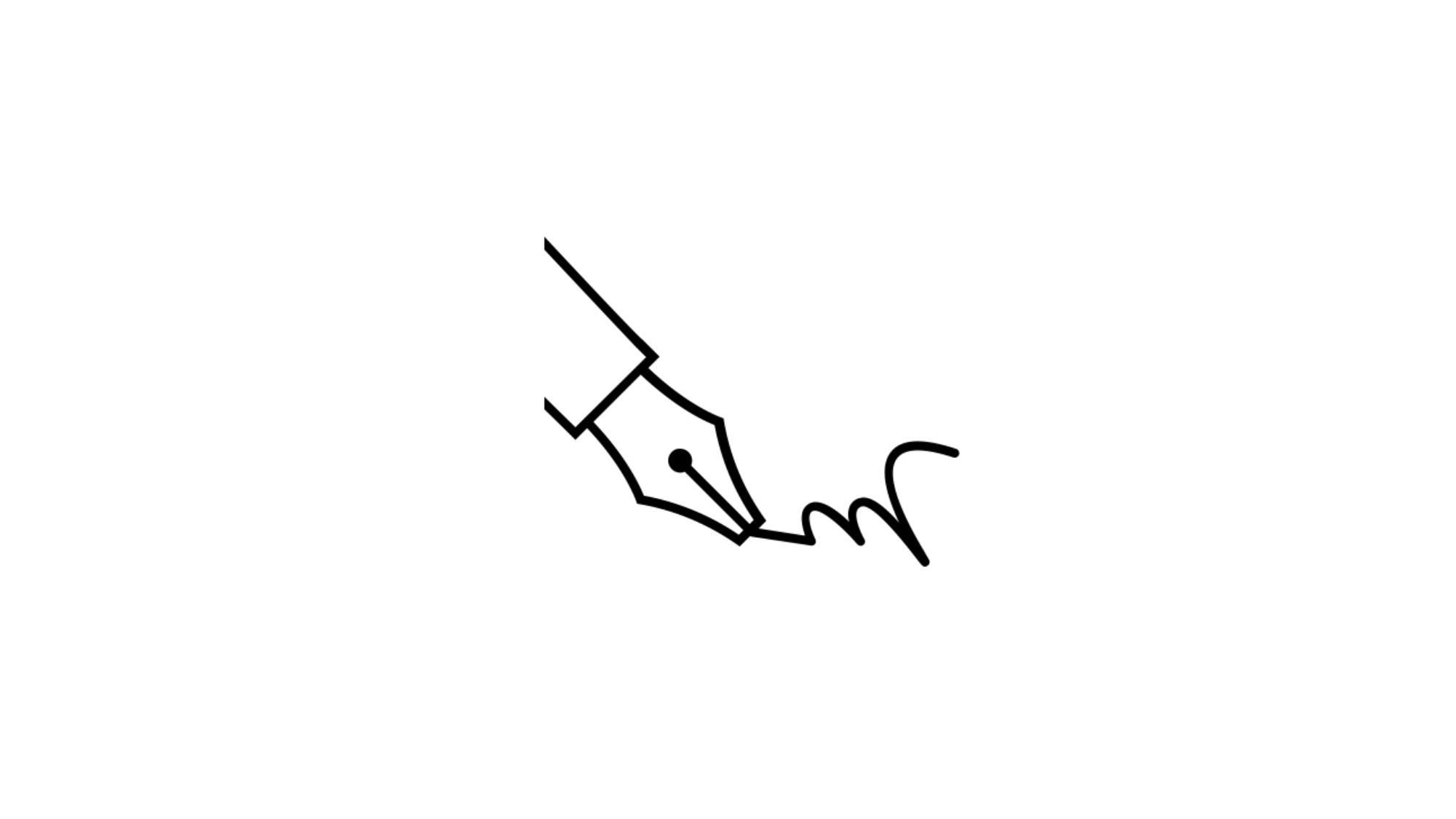

## Slide 25
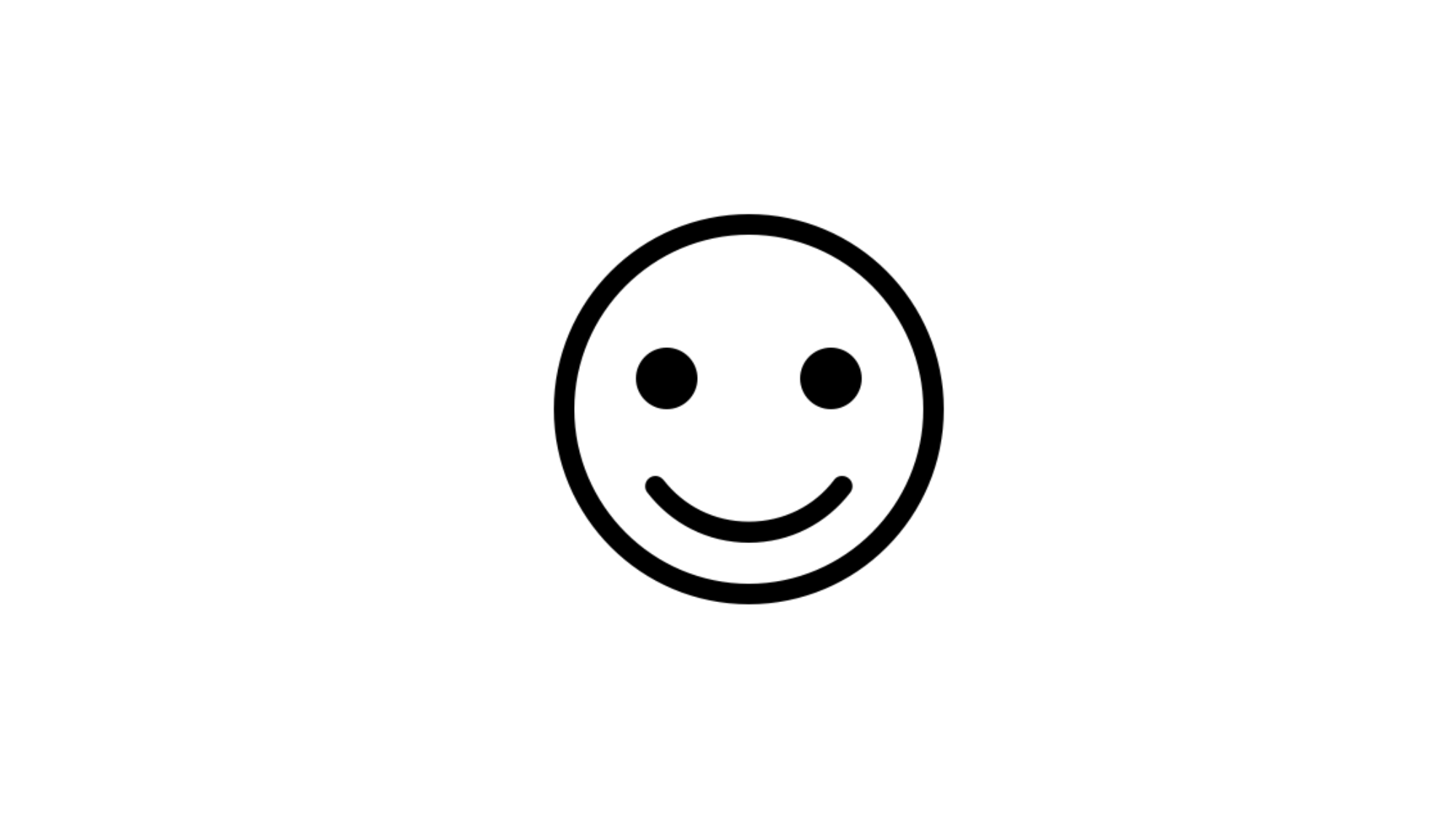

## Slide 26
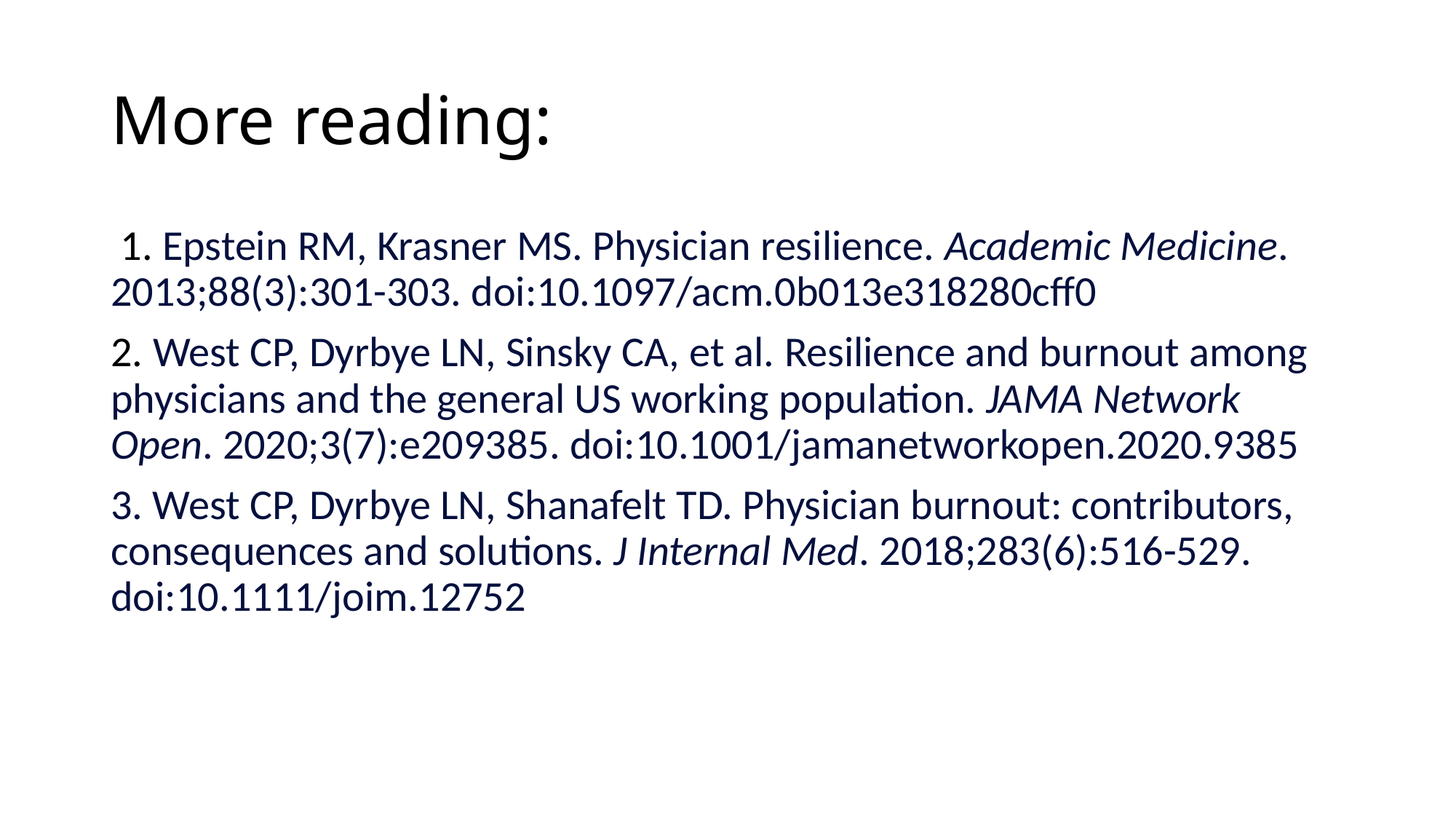

# More reading:
 1. Epstein RM, Krasner MS. Physician resilience. Academic Medicine. 2013;88(3):301-303. doi:10.1097/acm.0b013e318280cff0
2. West CP, Dyrbye LN, Sinsky CA, et al. Resilience and burnout among physicians and the general US working population. JAMA Network Open. 2020;3(7):e209385. doi:10.1001/jamanetworkopen.2020.9385
3. West CP, Dyrbye LN, Shanafelt TD. Physician burnout: contributors, consequences and solutions. J Internal Med. 2018;283(6):516-529. doi:10.1111/joim.12752

## Slide 27
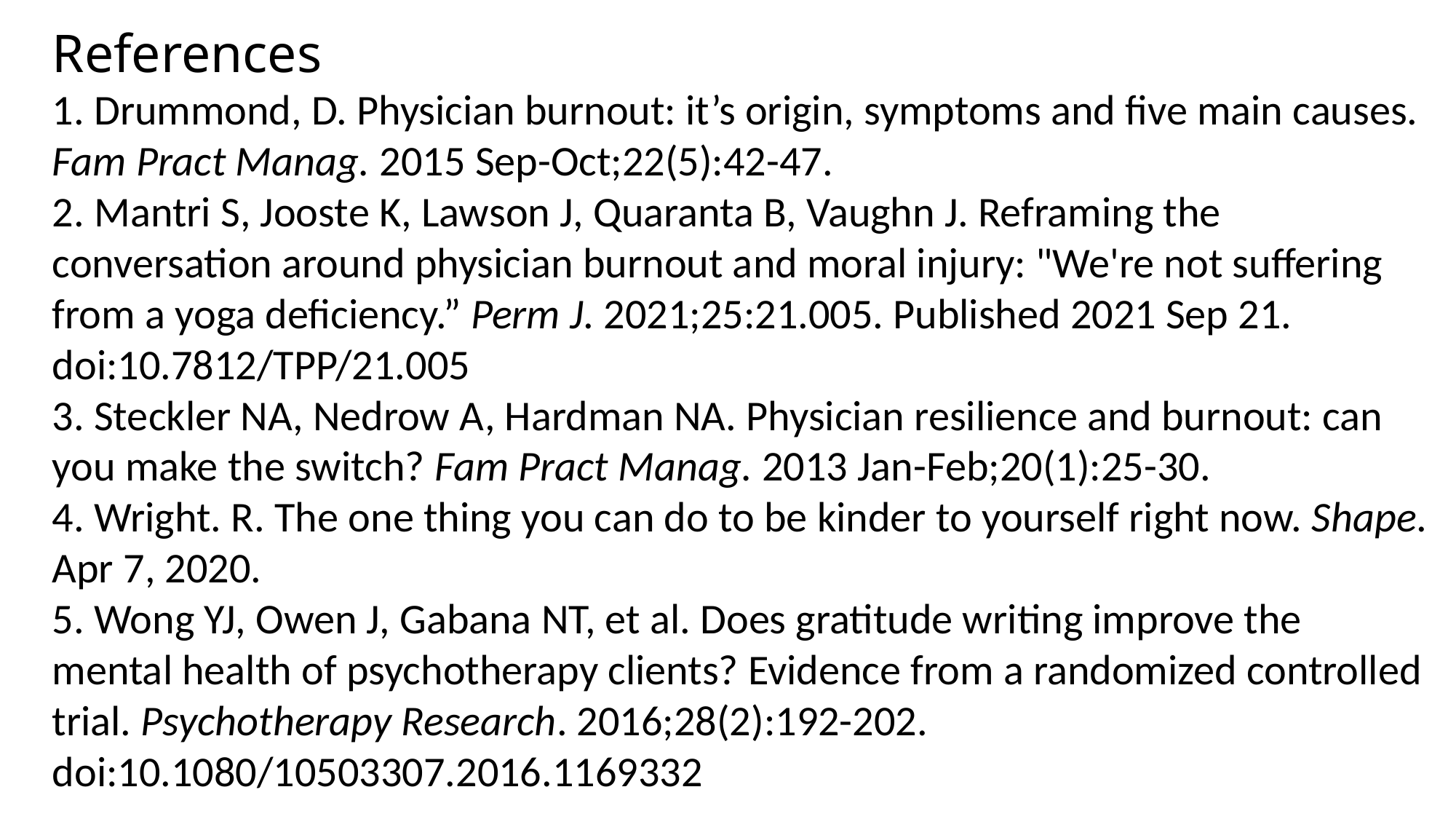

# References
1. Drummond, D. Physician burnout: it’s origin, symptoms and five main causes. Fam Pract Manag. 2015 Sep-Oct;22(5):42-47.
2. Mantri S, Jooste K, Lawson J, Quaranta B, Vaughn J. Reframing the conversation around physician burnout and moral injury: "We're not suffering from a yoga deficiency.” Perm J. 2021;25:21.005. Published 2021 Sep 21. doi:10.7812/TPP/21.005
3. Steckler NA, Nedrow A, Hardman NA. Physician resilience and burnout: can you make the switch? Fam Pract Manag. 2013 Jan-Feb;20(1):25-30.
4. Wright. R. The one thing you can do to be kinder to yourself right now. Shape. Apr 7, 2020.
5. Wong YJ, Owen J, Gabana NT, et al. Does gratitude writing improve the mental health of psychotherapy clients? Evidence from a randomized controlled trial. Psychotherapy Research. 2016;28(2):192-202. doi:10.1080/10503307.2016.1169332
