# Supplementary material for: What You Didn’t Learn in Residency: A Collective Curriculum for New Academic EM Faculty and Fellows
Source: J Educ Teach Emerg Med. 2024 Jan 31;9(1):C16–40. doi: 10.21980/J8WP9Z (PMC10854884; doi:10.21980/J8WP9Z)
Supplement: Supplementary file 3 — Please see associated Power Point [file jetem-9-1-C16-AppendixG.pptx]

## Slide 1
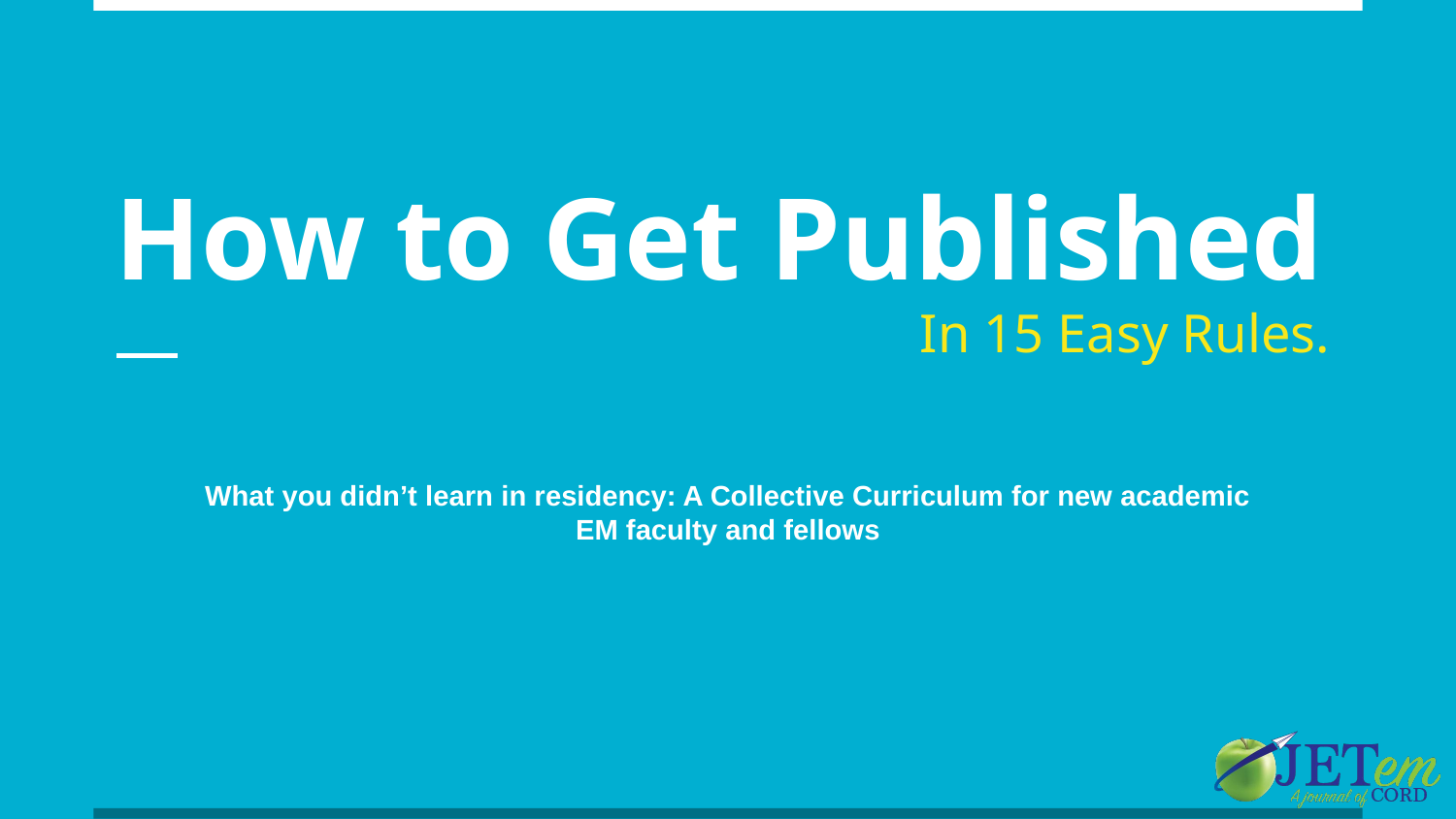

# How to Get Published
In 15 Easy Rules.
What you didn’t learn in residency: A Collective Curriculum for new academic EM faculty and fellows

## Slide 2
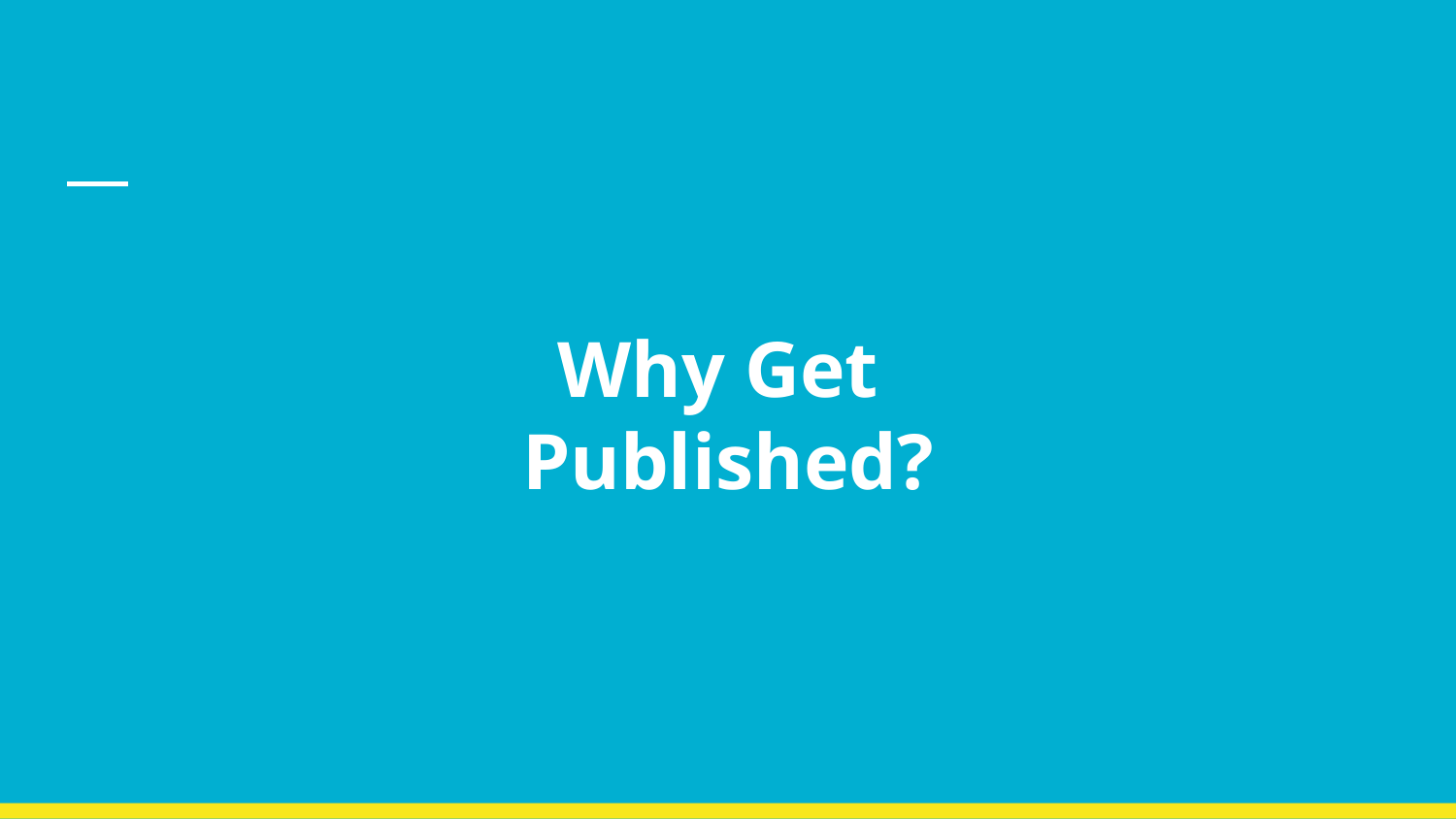

# Why Get
Published?

## Slide 3
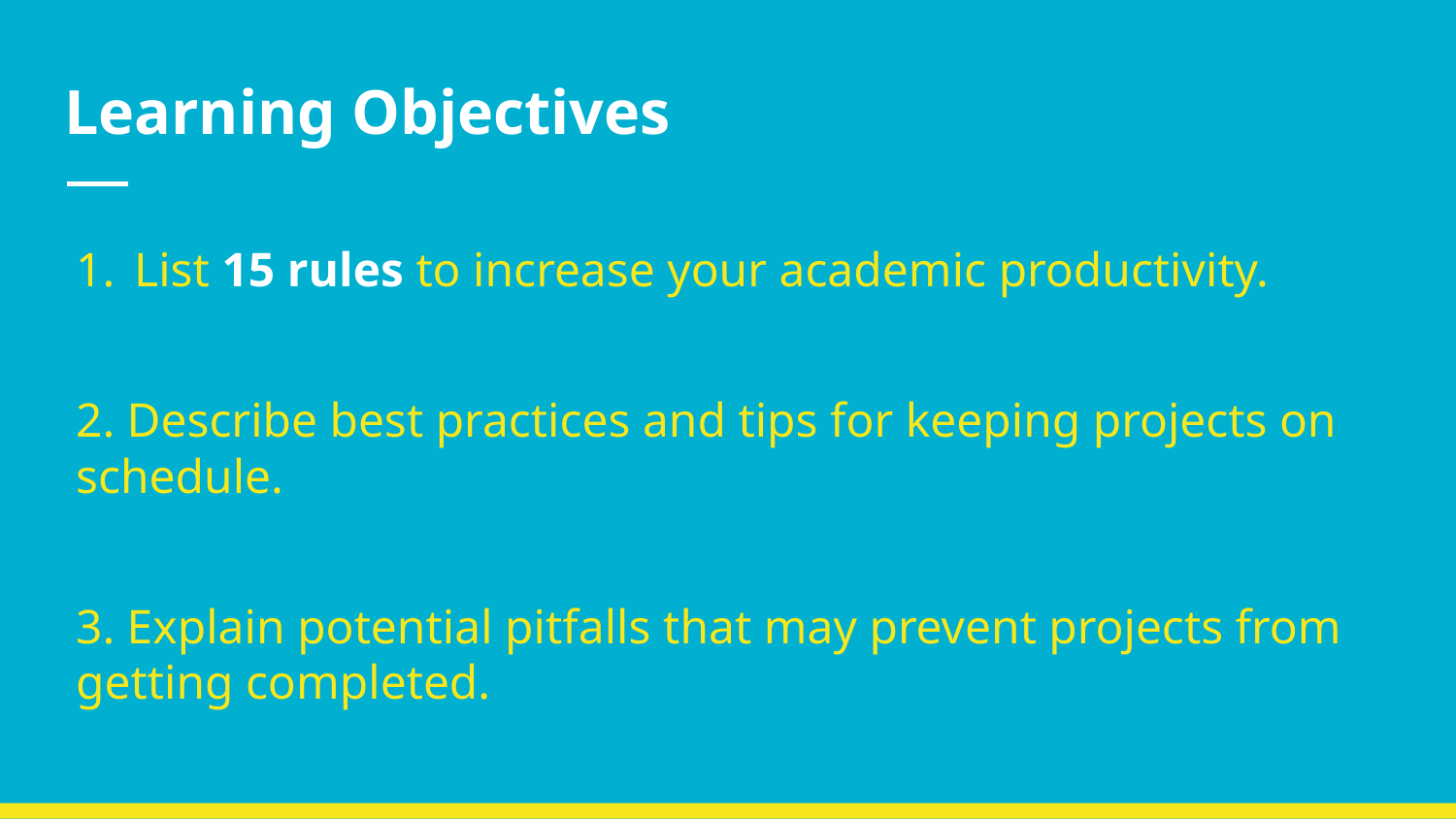

# Learning Objectives
List 15 rules to increase your academic productivity.
2. Describe best practices and tips for keeping projects on schedule.
3. Explain potential pitfalls that may prevent projects from getting completed.

## Slide 4
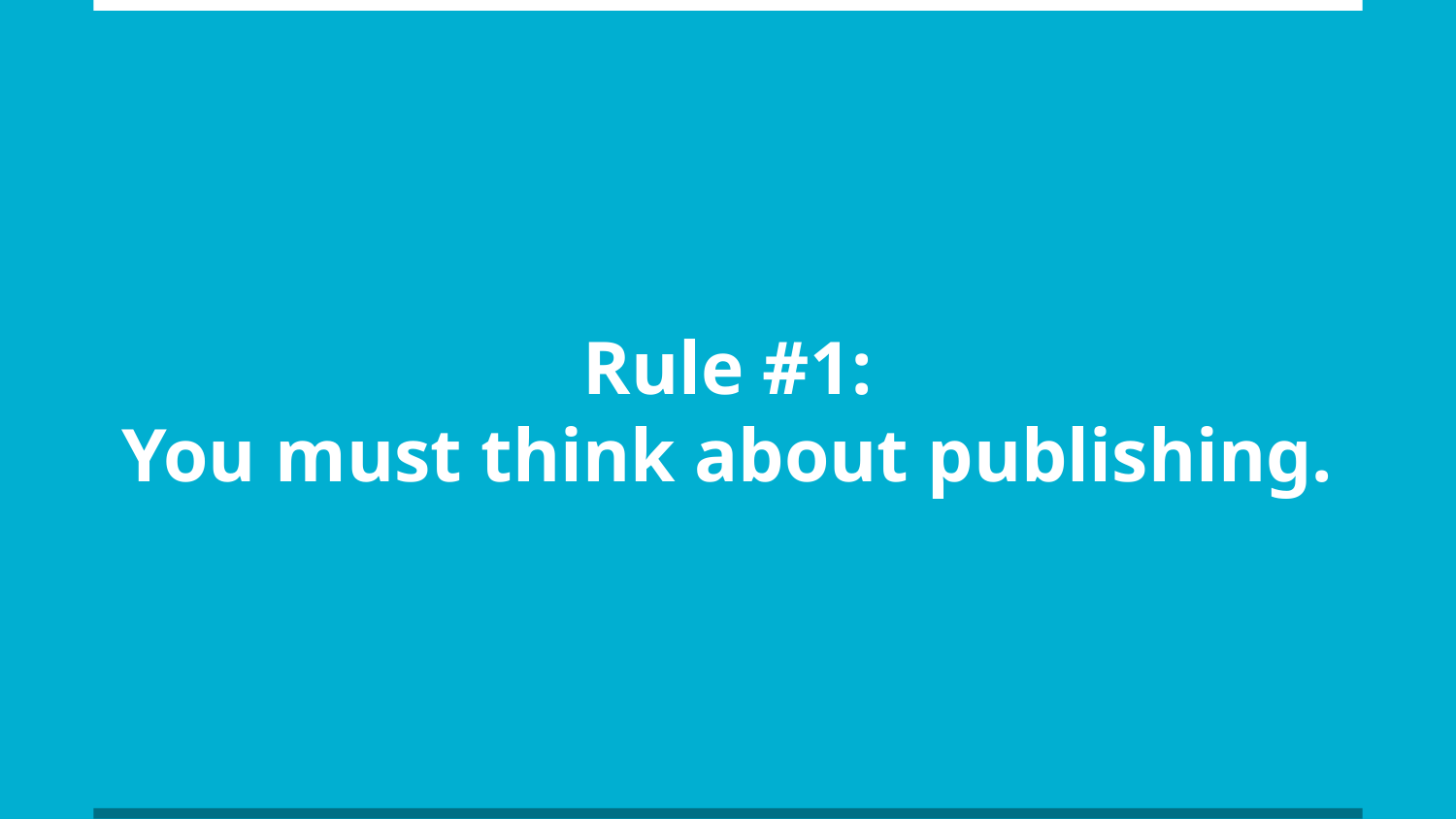

# Rule #1:
You must think about publishing.

## Slide 5
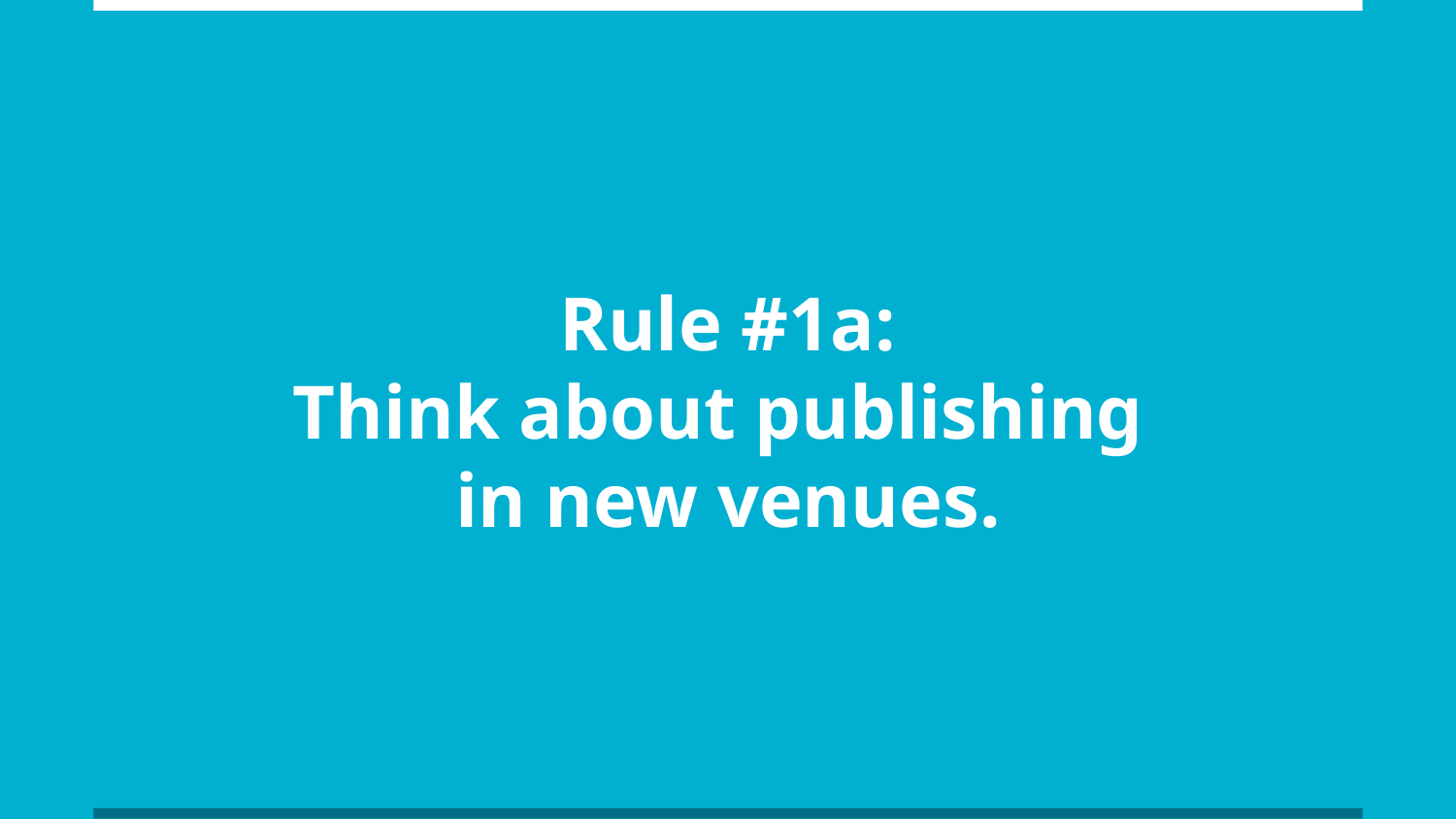

# Rule #1a:
Think about publishing
in new venues.

## Slide 6
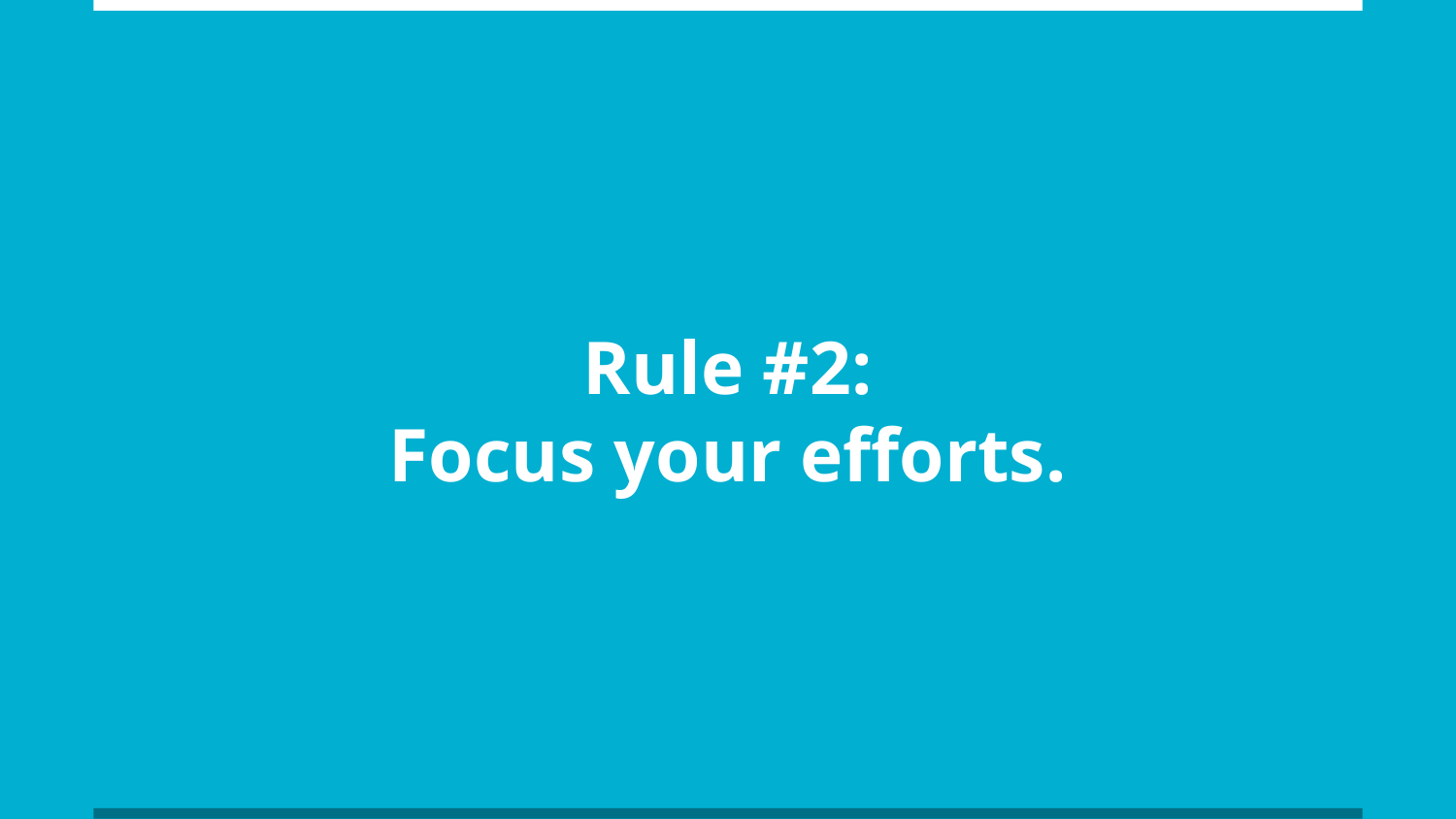

# Rule #2:
Focus your efforts.

## Slide 7
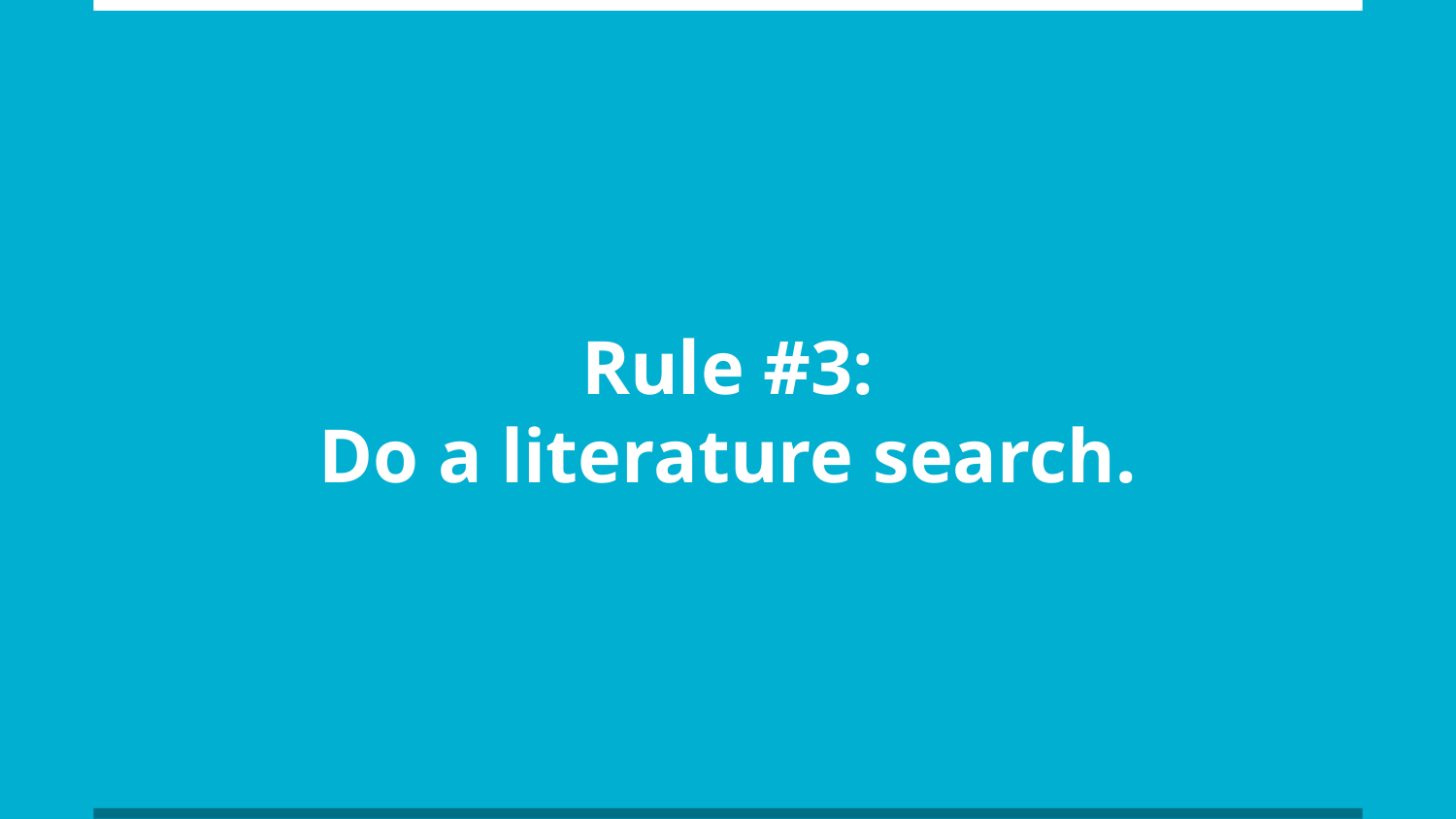

# Rule #3:
Do a literature search.

## Slide 8
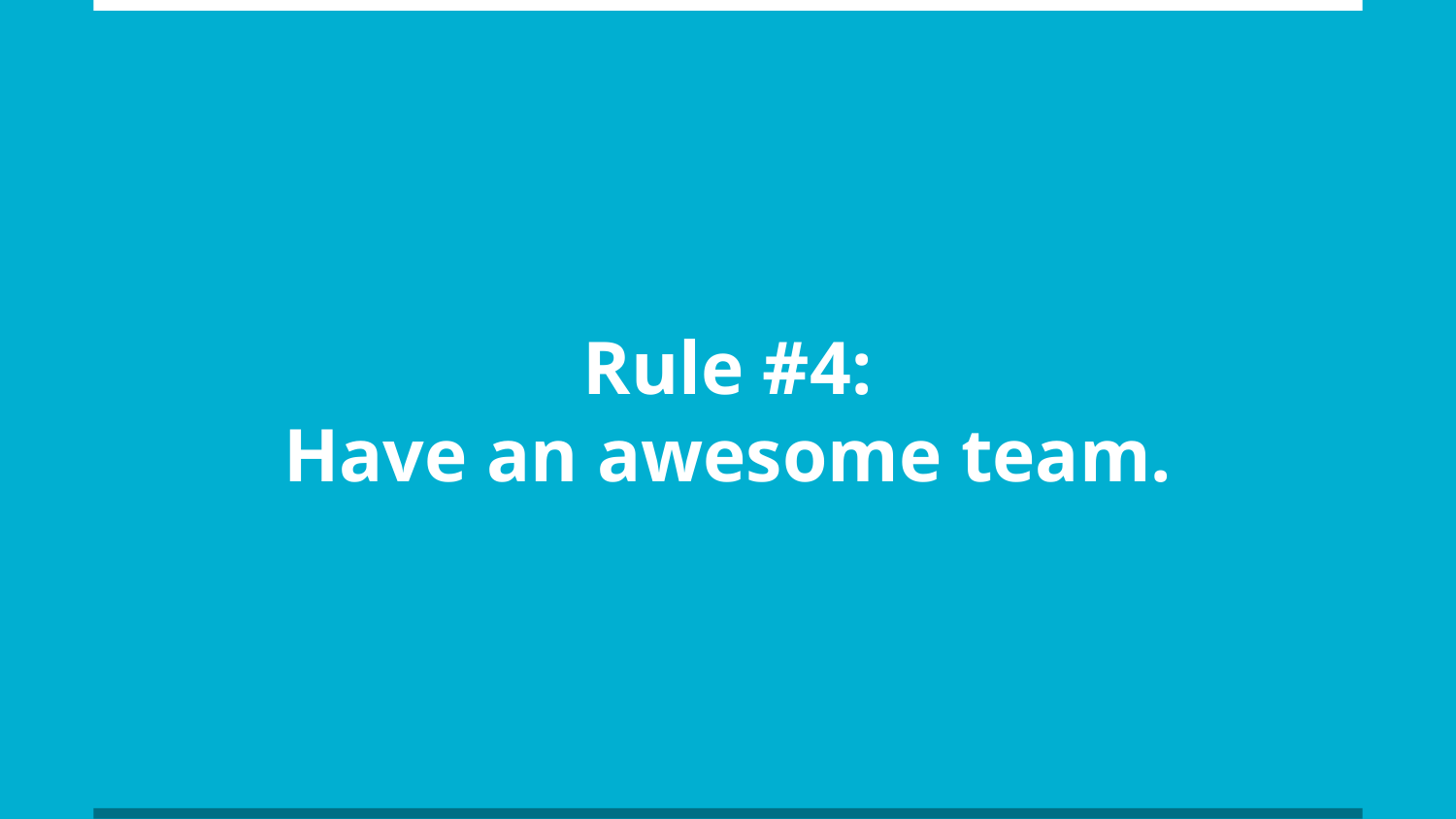

# Rule #4:
Have an awesome team.

## Slide 9
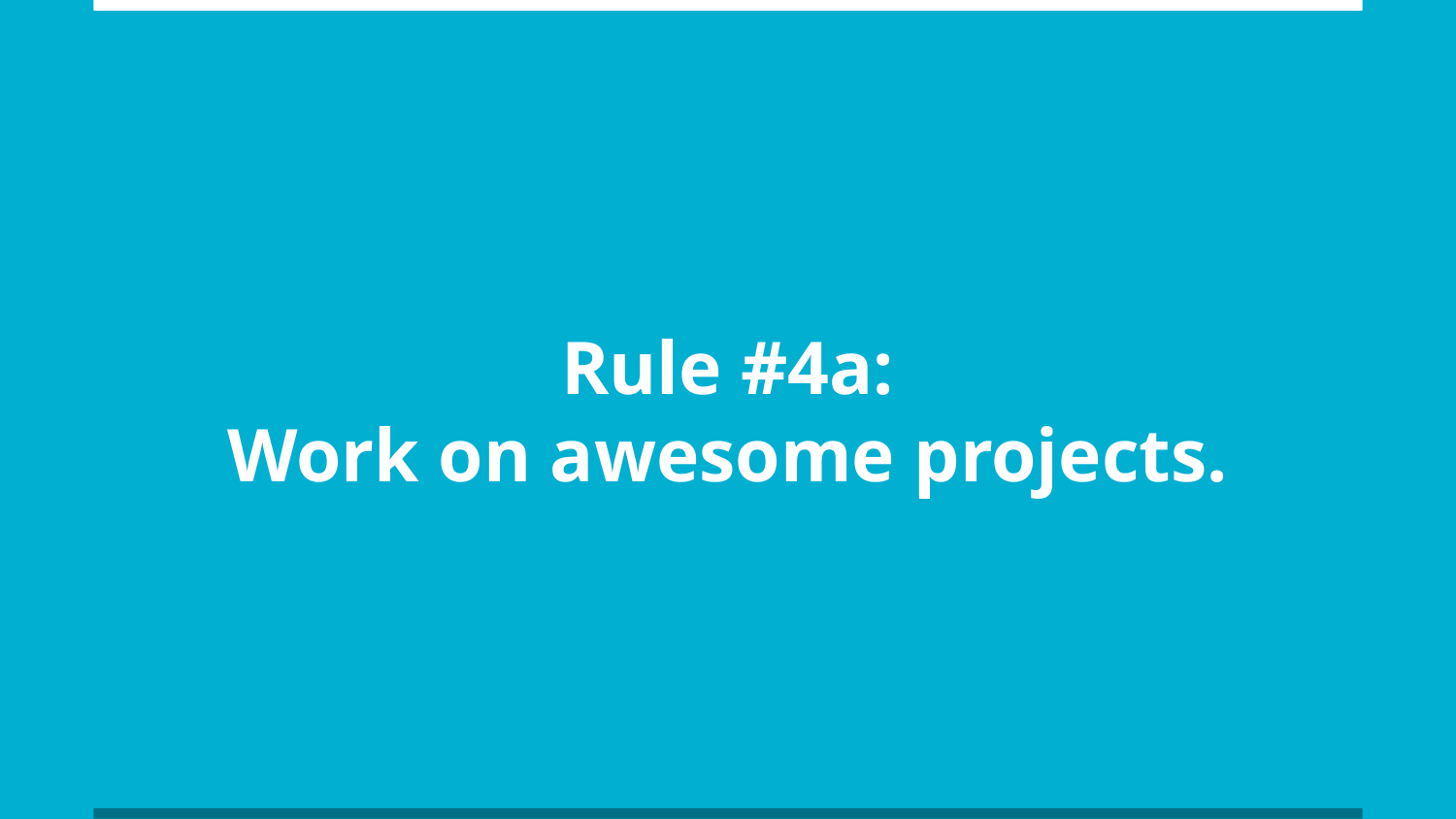

# Rule #4a:
Work on awesome projects.

## Slide 10
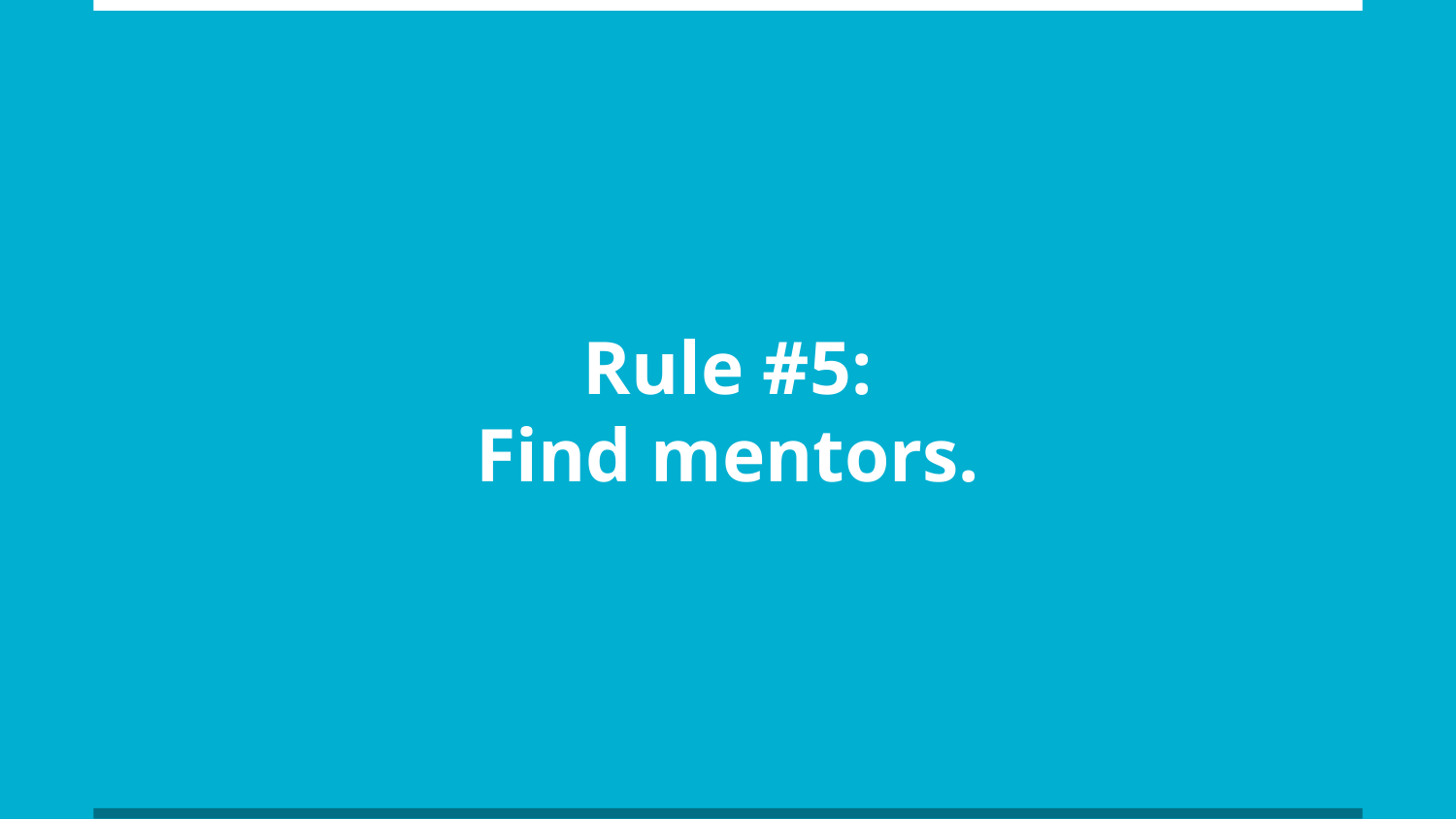

# Rule #5:
Find mentors.

## Slide 11
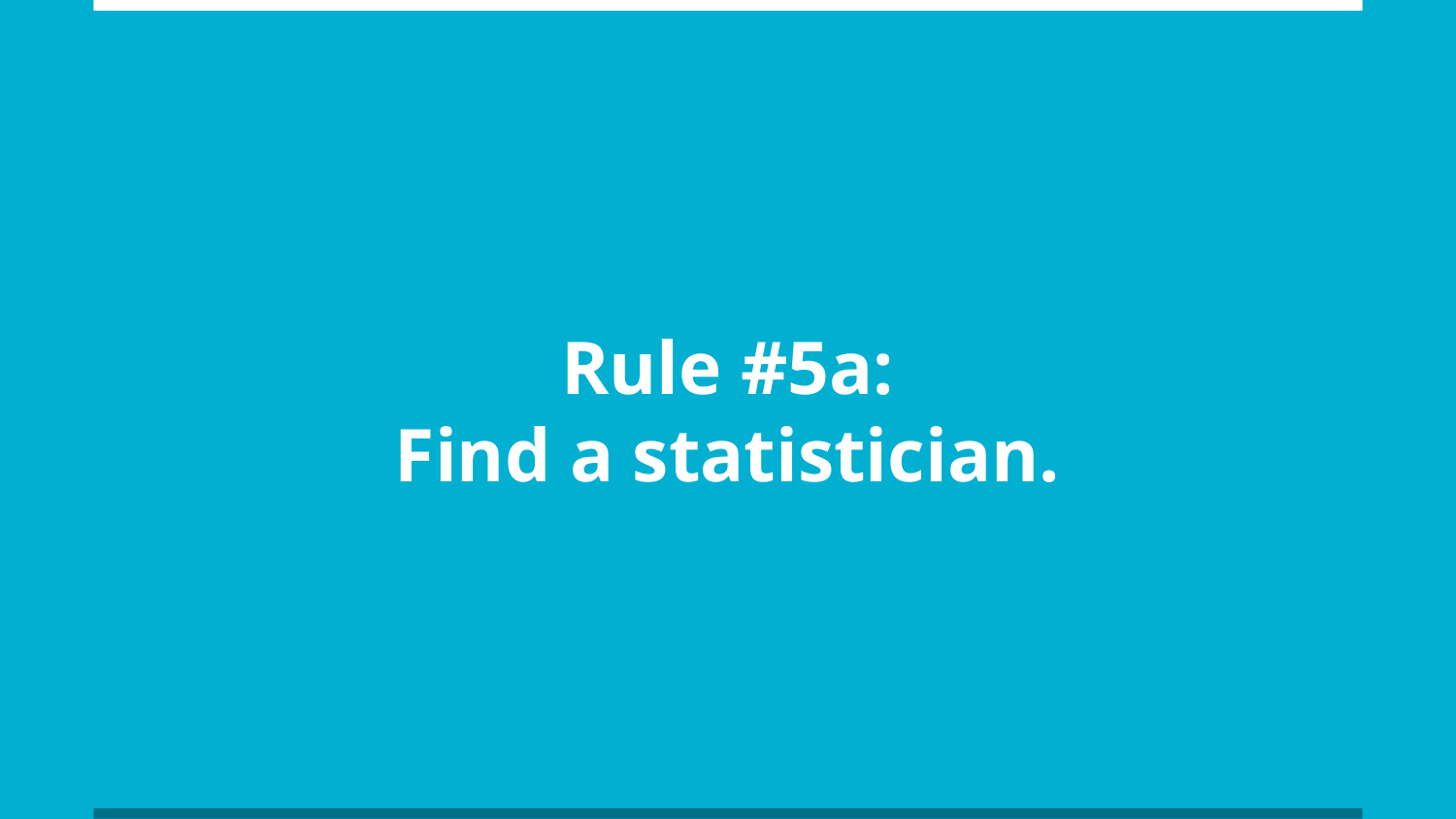

# Rule #5a:
Find a statistician.

## Slide 12
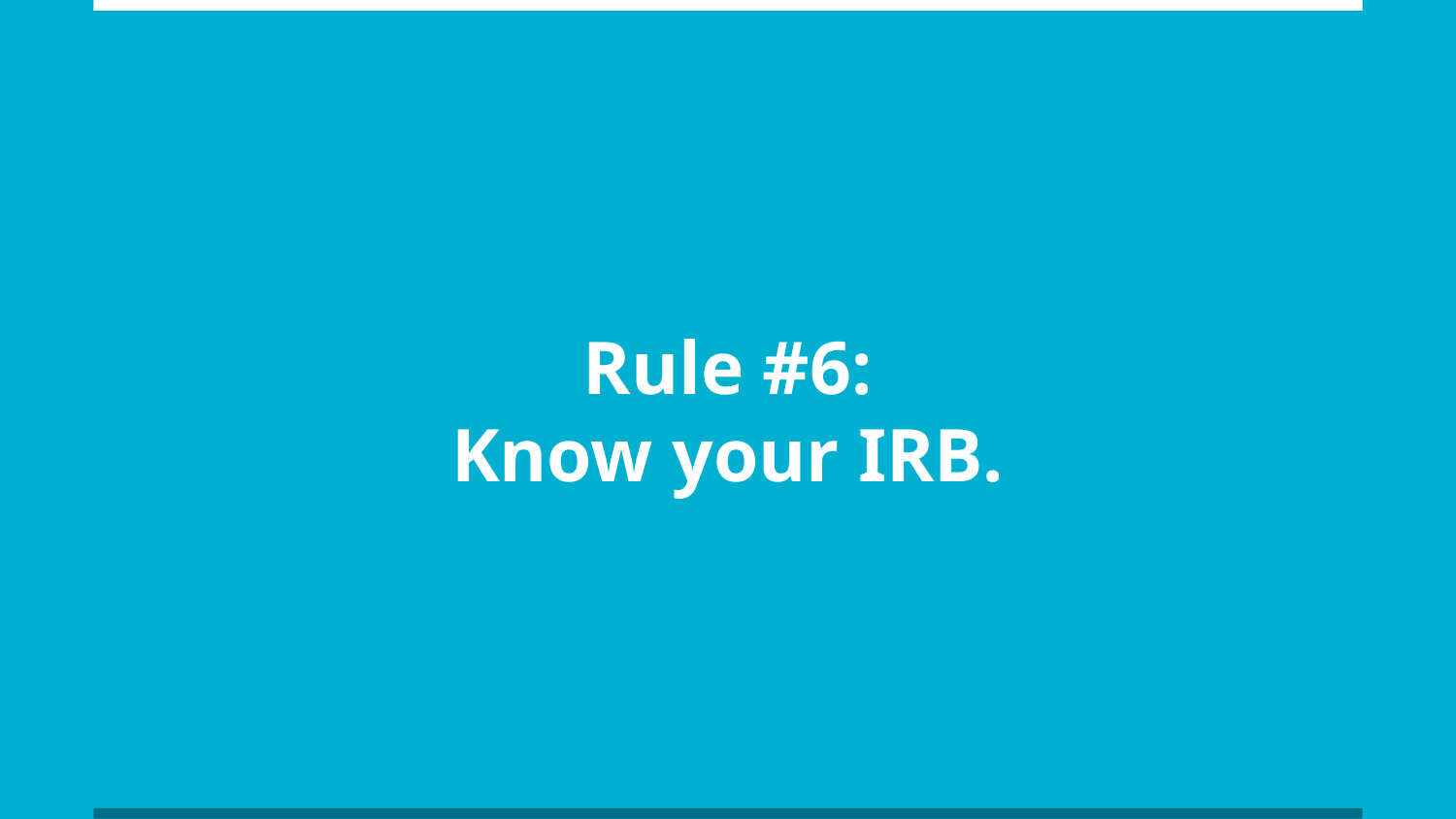

# Rule #6:
Know your IRB.

## Slide 13
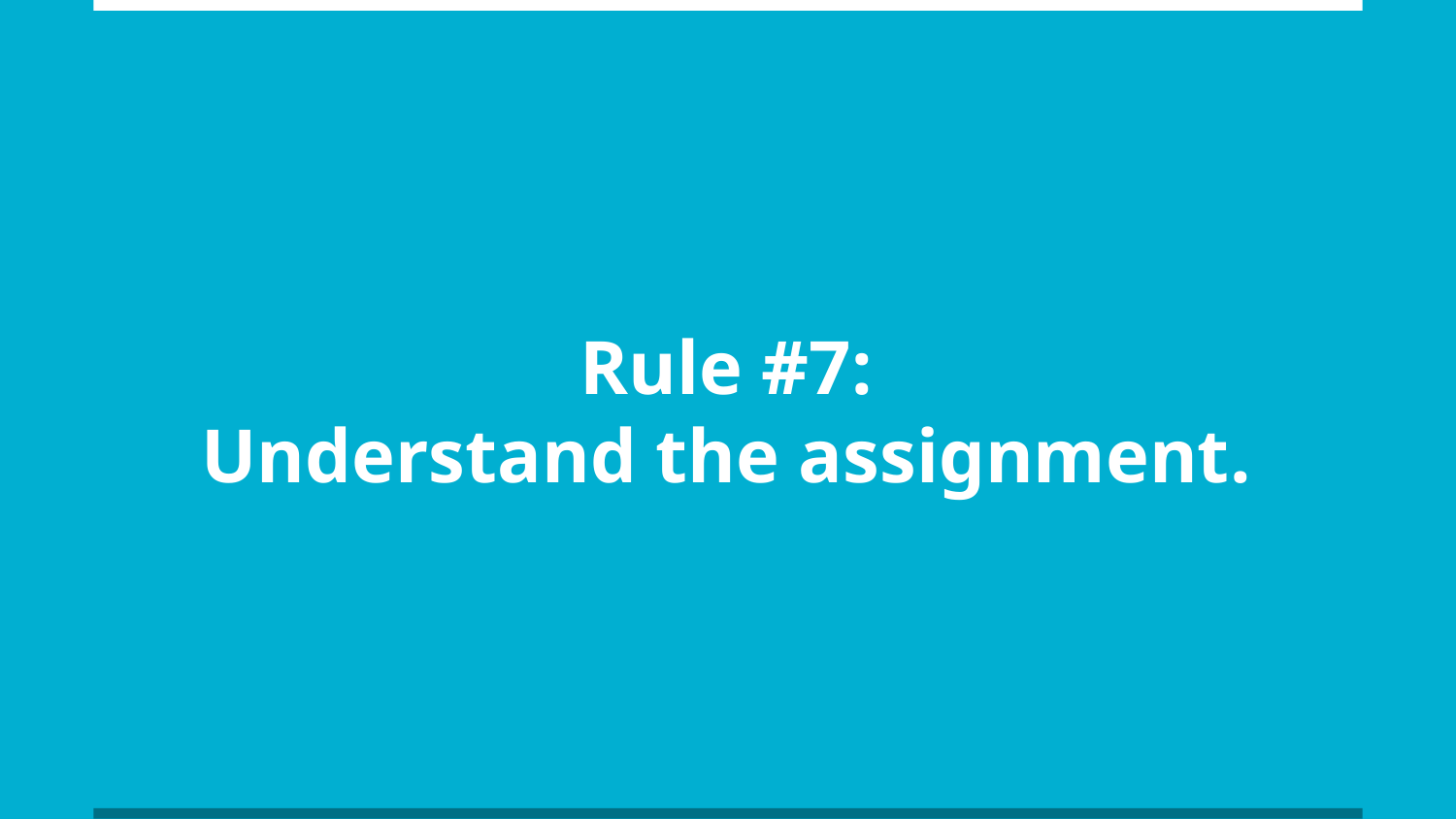

# Rule #7:
Understand the assignment.

## Slide 14
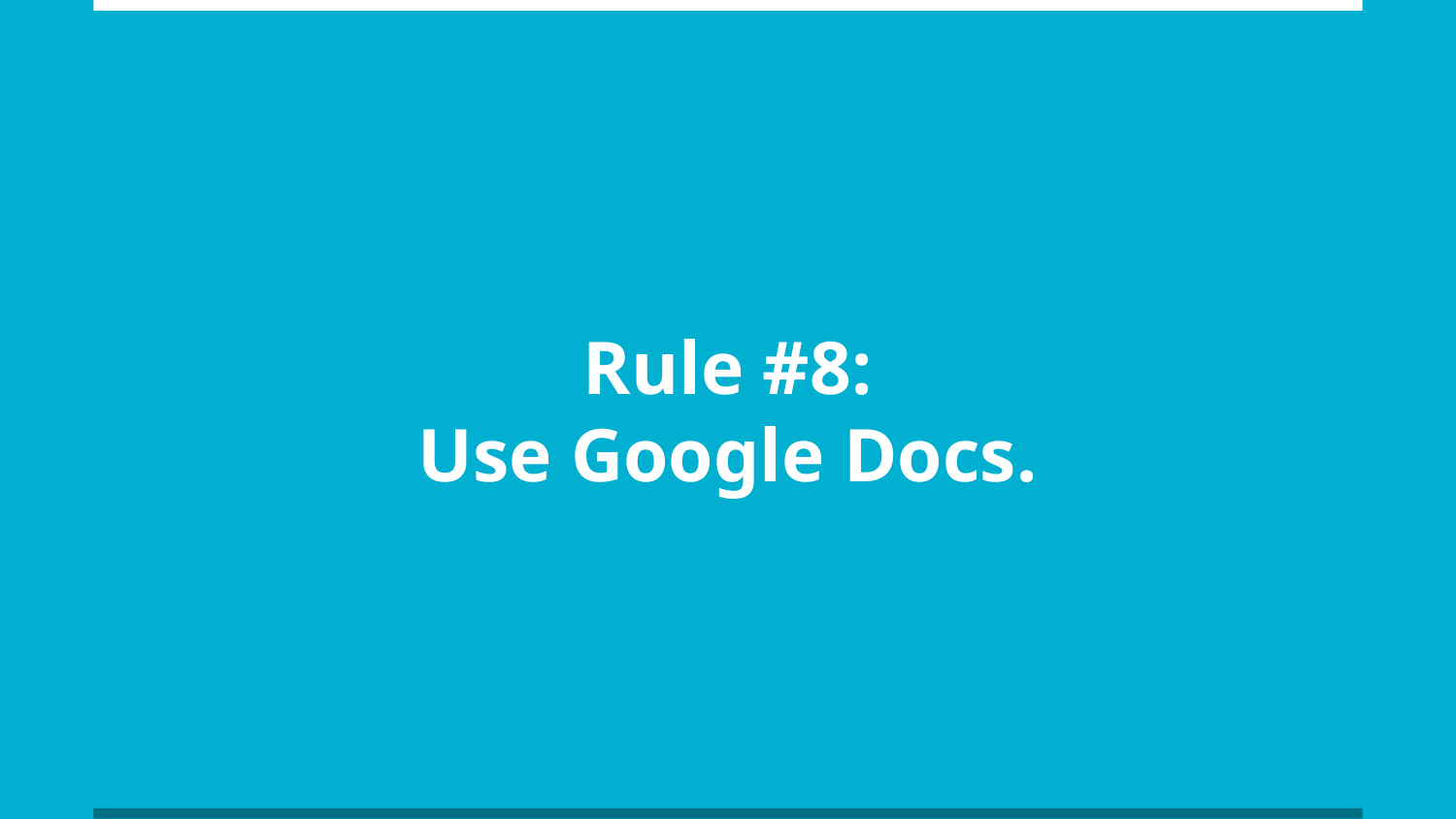

# Rule #8:
Use Google Docs.

## Slide 15
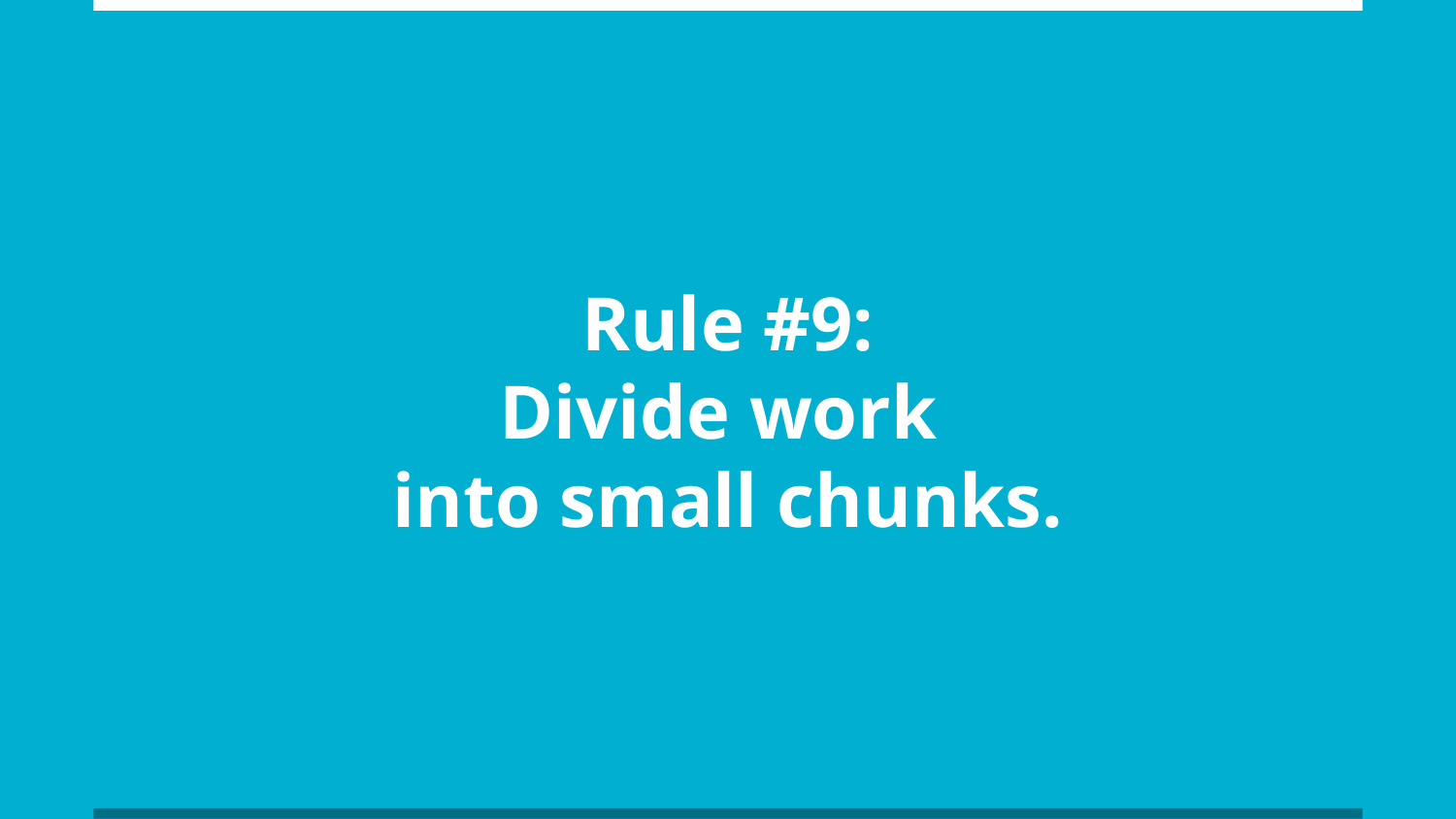

# Rule #9:
Divide work
into small chunks.

## Slide 16
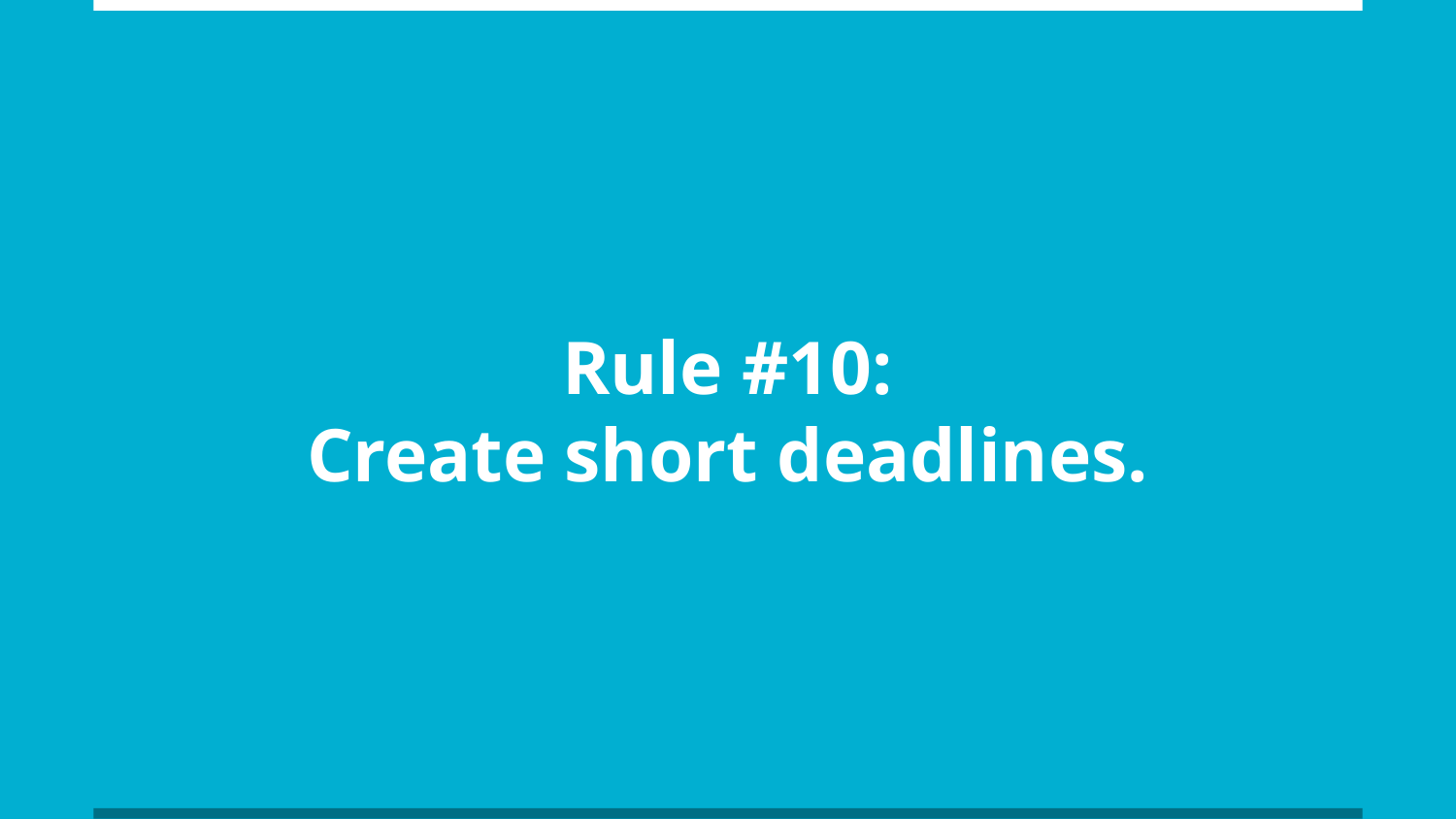

# Rule #10:
Create short deadlines.

## Slide 17
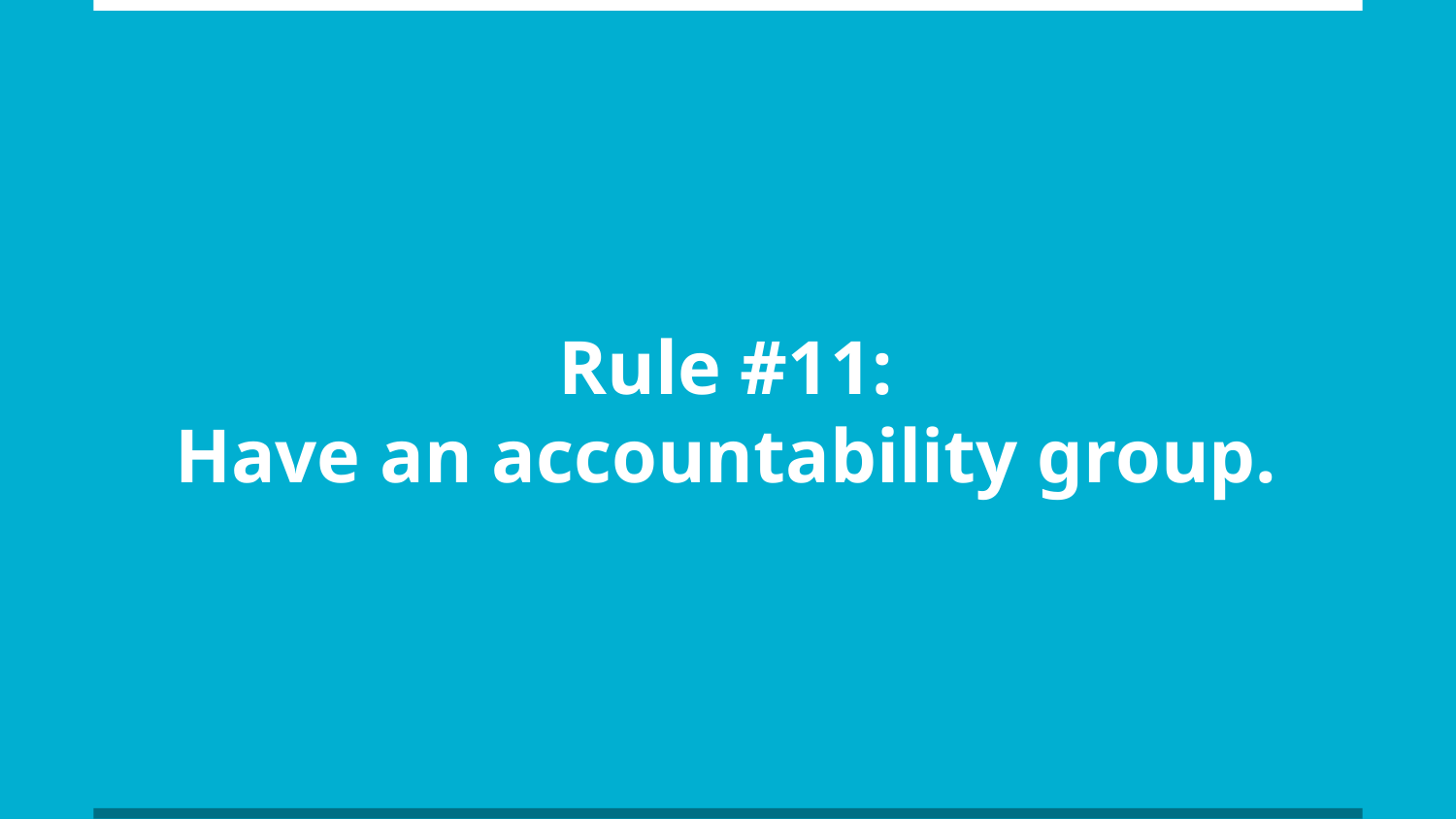

# Rule #11:
Have an accountability group.

## Slide 18
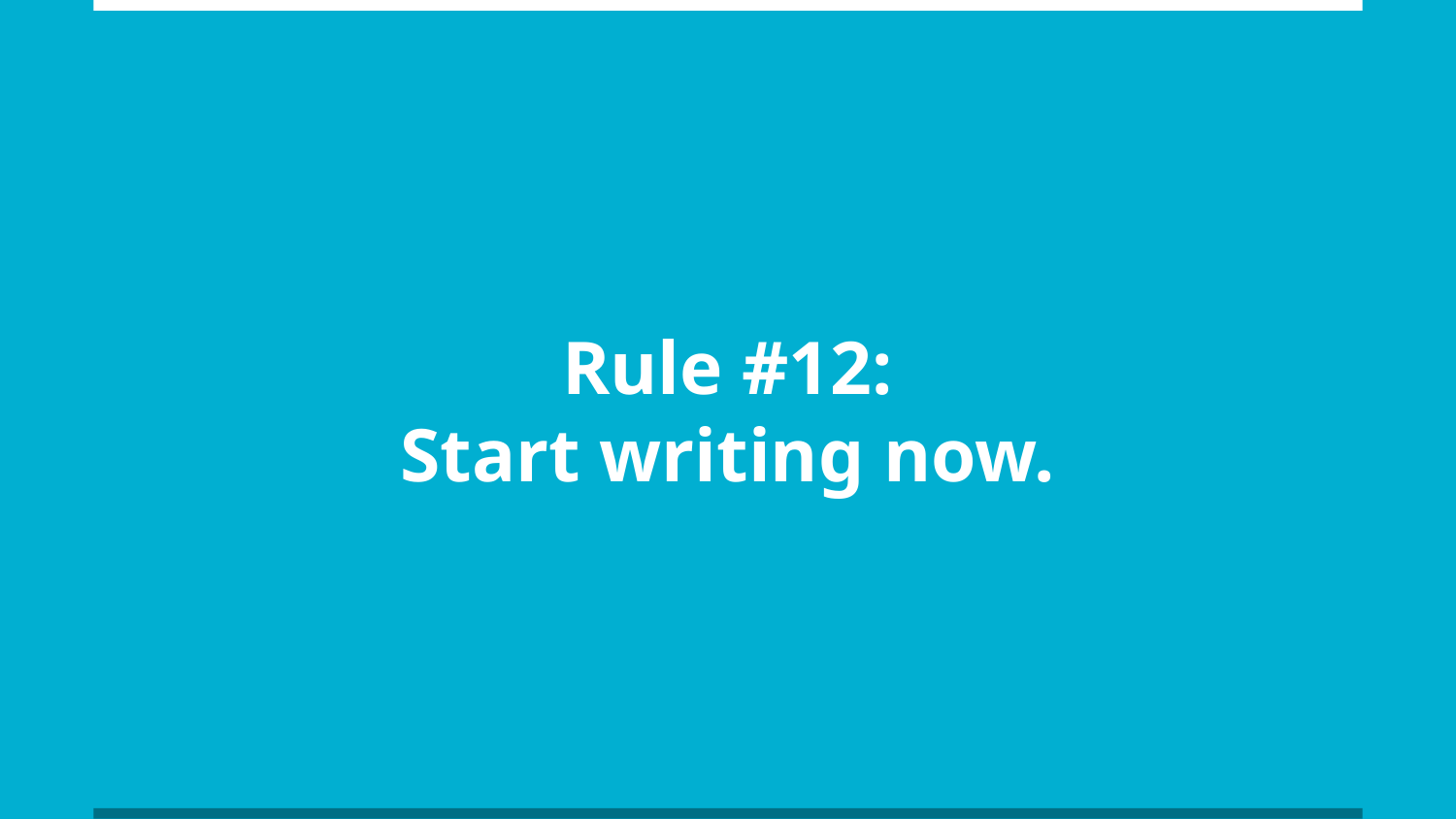

# Rule #12:
Start writing now.

## Slide 19
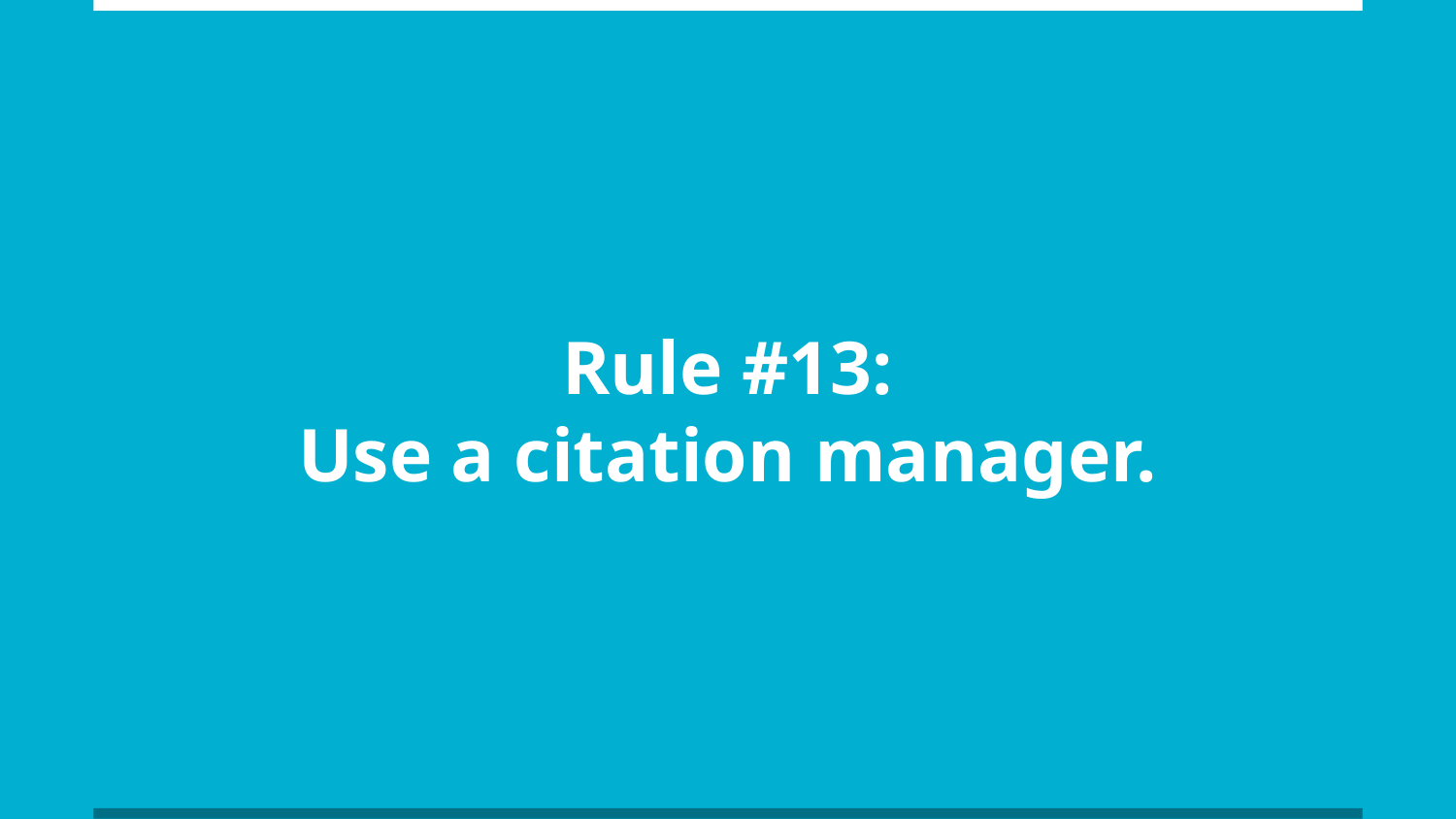

# Rule #13:
Use a citation manager.

## Slide 20
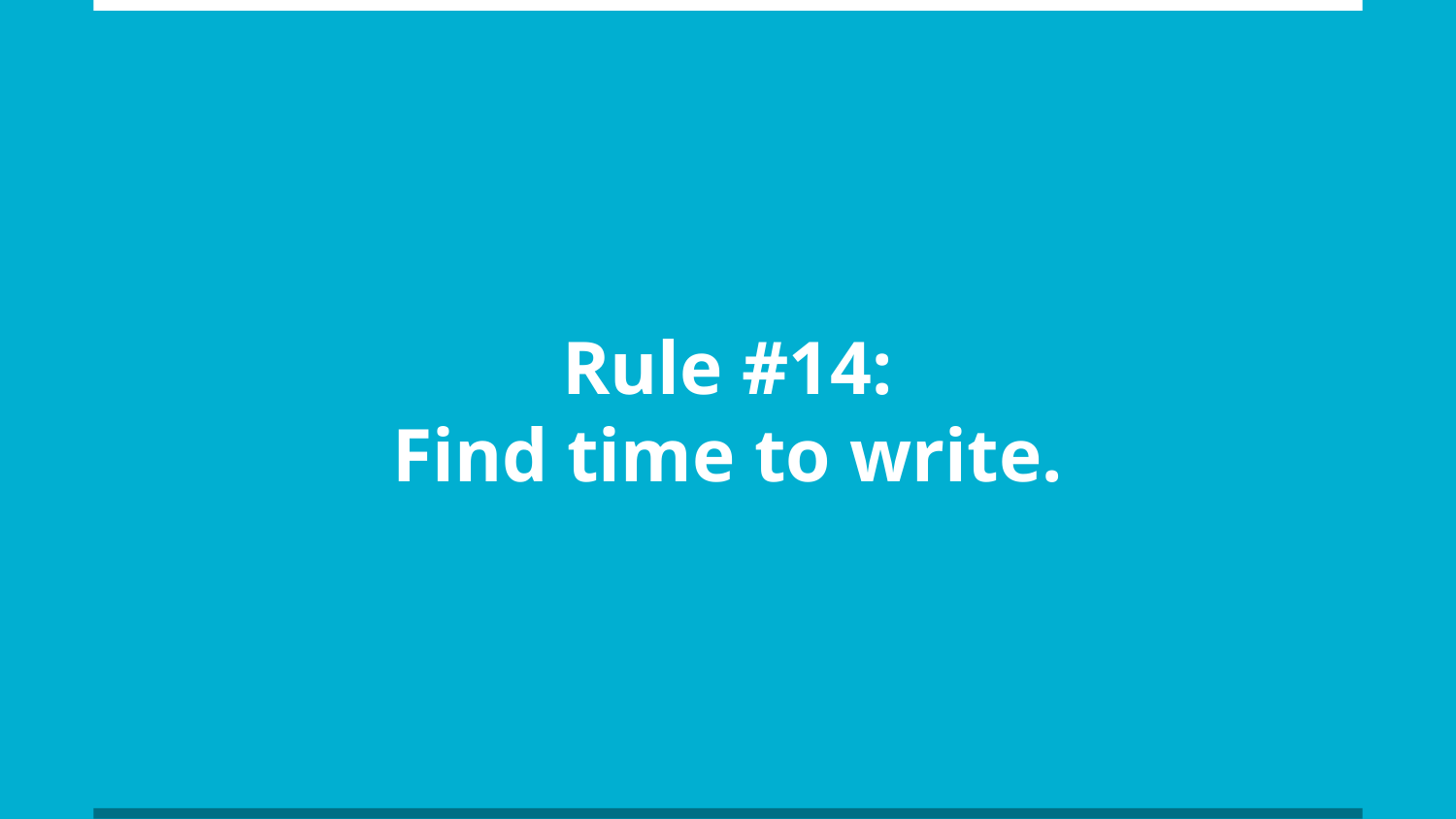

# Rule #14:
Find time to write.

## Slide 21
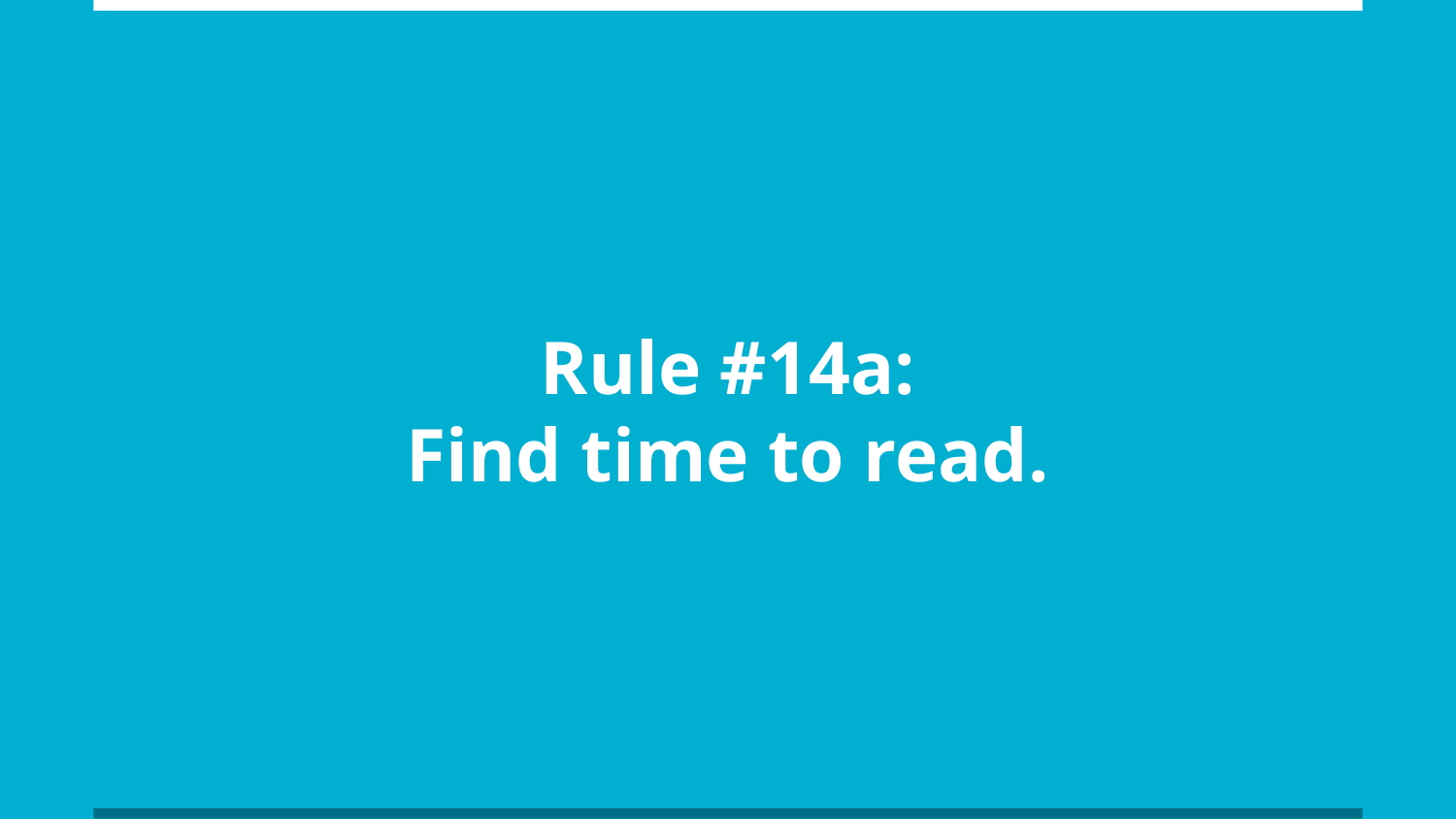

# Rule #14a:
Find time to read.

## Slide 22
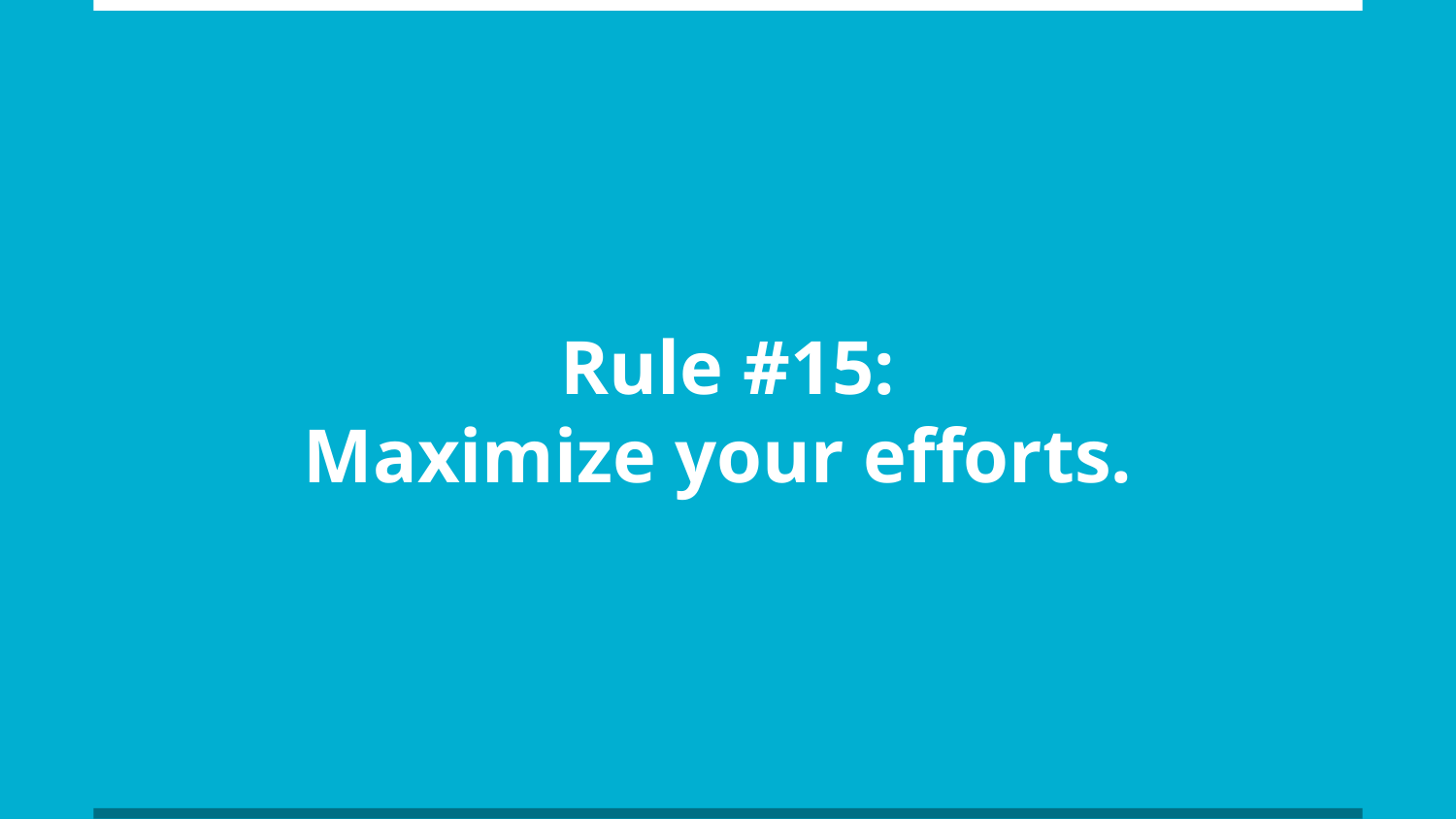

# Rule #15:
Maximize your efforts.

## Slide 23
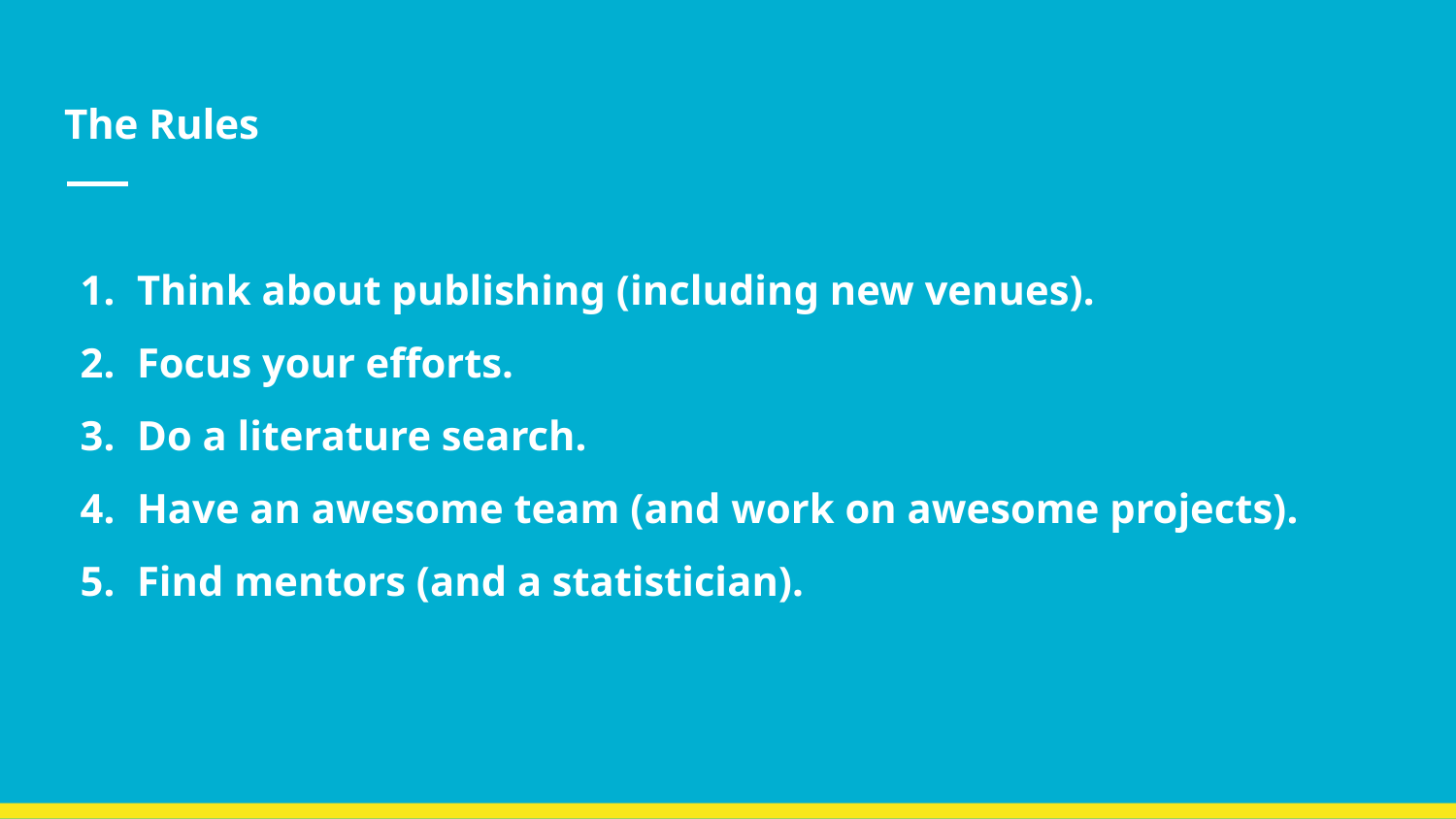

# The Rules
Think about publishing (including new venues).
Focus your efforts.
Do a literature search.
Have an awesome team (and work on awesome projects).
Find mentors (and a statistician).

## Slide 24
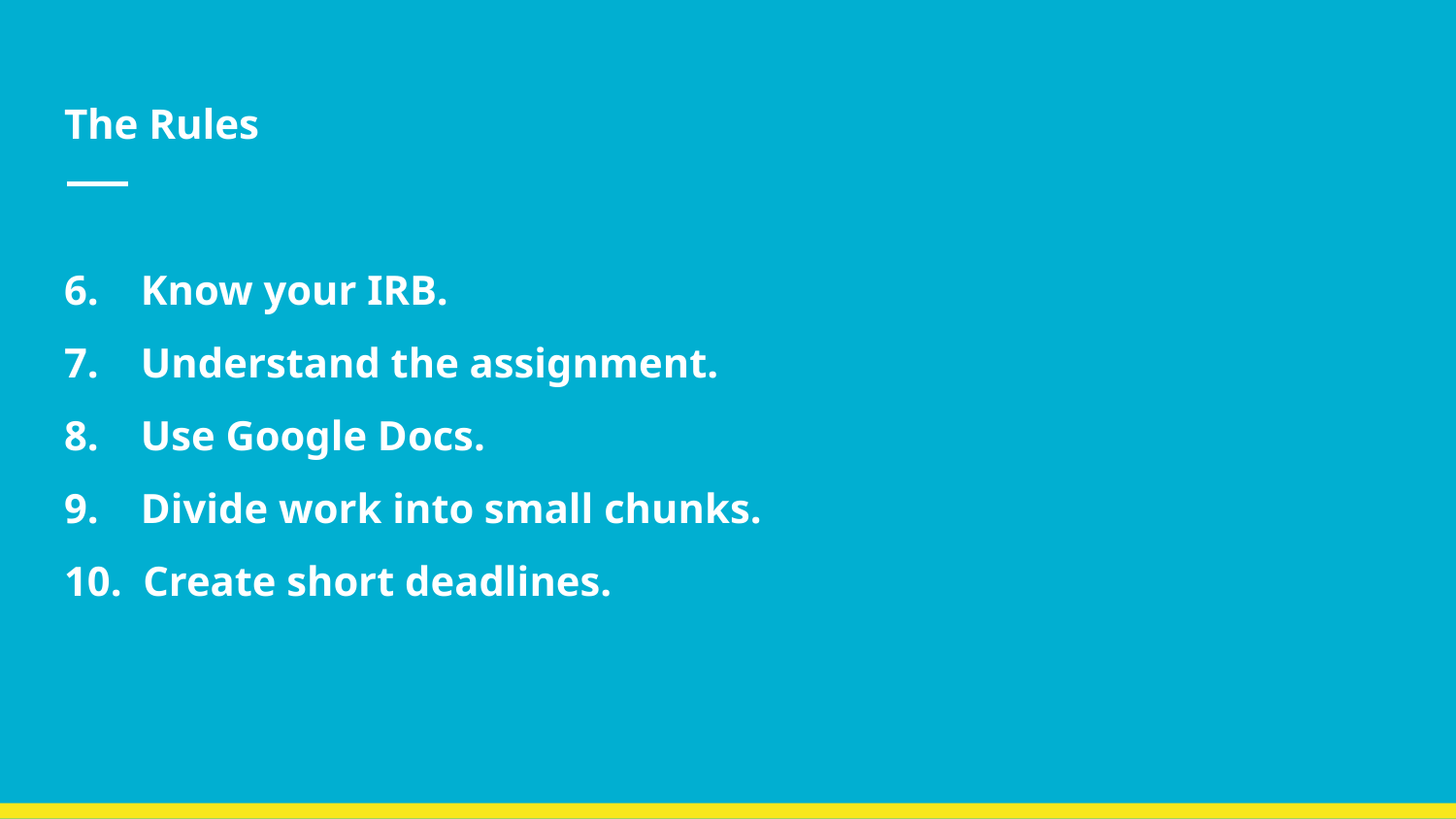

# The Rules
6. Know your IRB.
7. Understand the assignment.
8. Use Google Docs.
9. Divide work into small chunks.
10. Create short deadlines.

## Slide 25
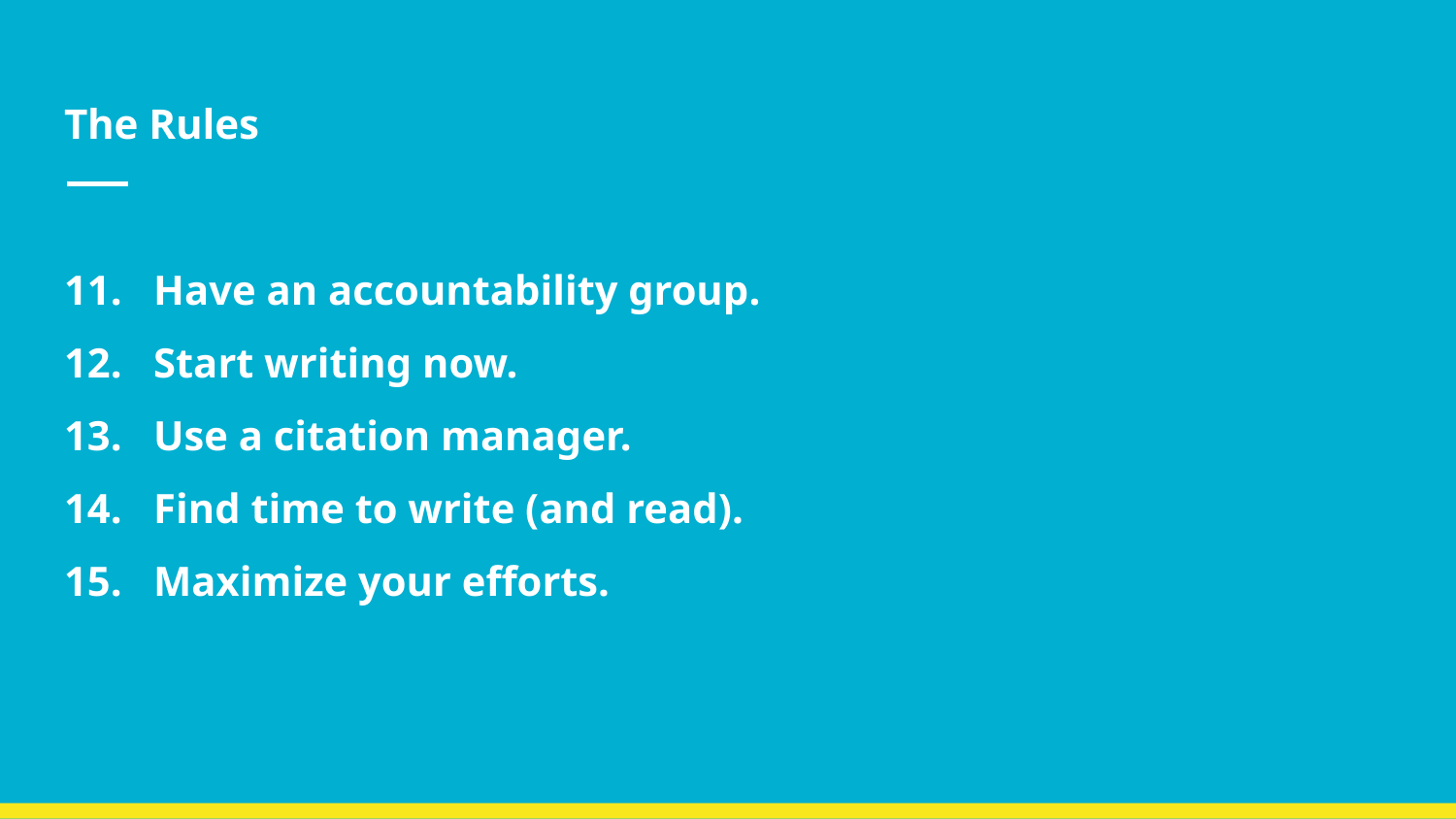

# The Rules
11. Have an accountability group.
12. Start writing now.
13. Use a citation manager.
14. Find time to write (and read).
15. Maximize your efforts.

## Slide 26
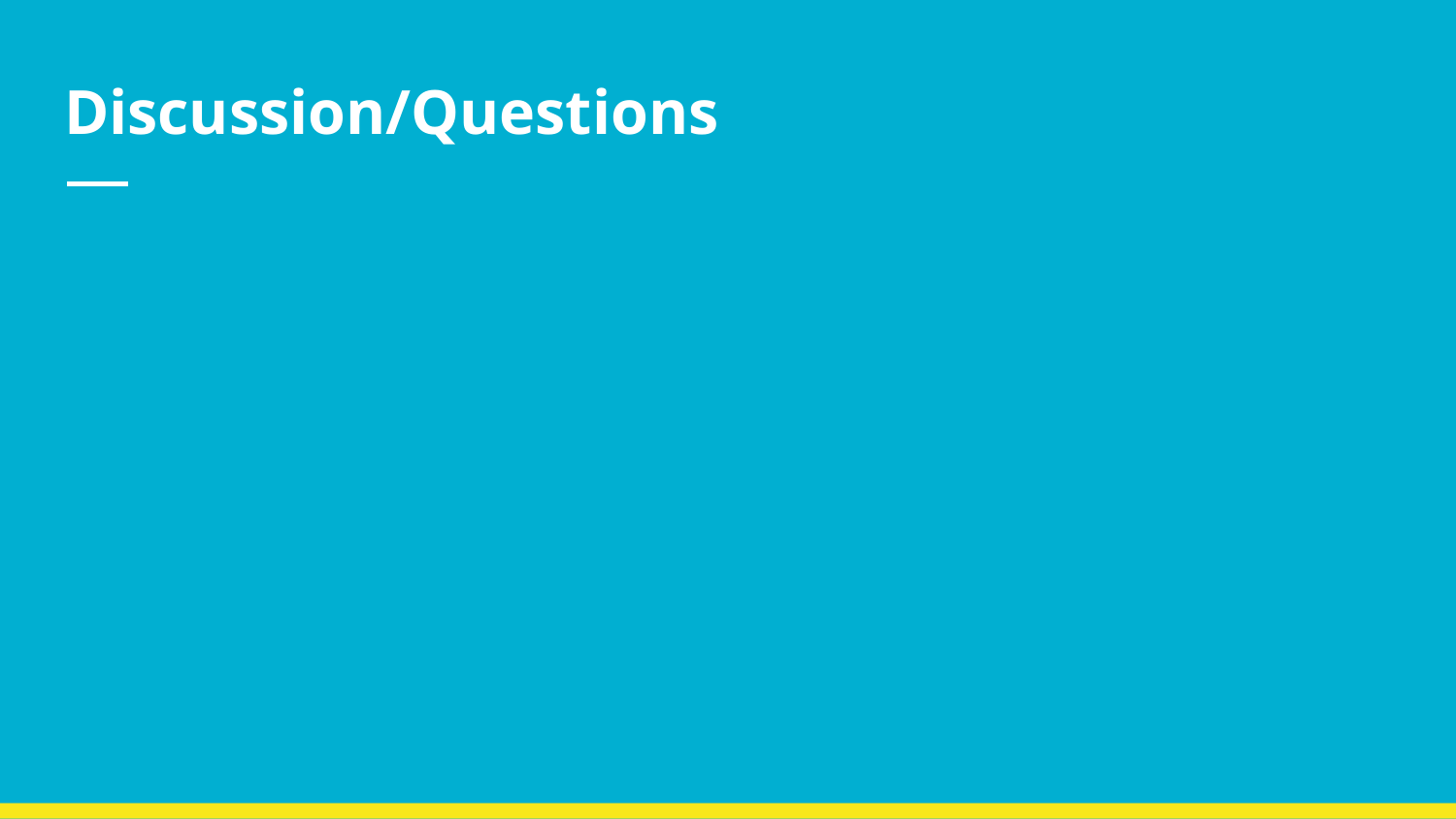

# Discussion/Questions
