# Supplementary material for: What You Didn’t Learn in Residency: A Collective Curriculum for New Academic EM Faculty and Fellows
Source: J Educ Teach Emerg Med. 2024 Jan 31;9(1):C16–40. doi: 10.21980/J8WP9Z (PMC10854884; doi:10.21980/J8WP9Z)
Supplement: Supplementary file 4 — Please see associated Power Point [file jetem-9-1-C16-AppendixH.pptx]

## Slide 1
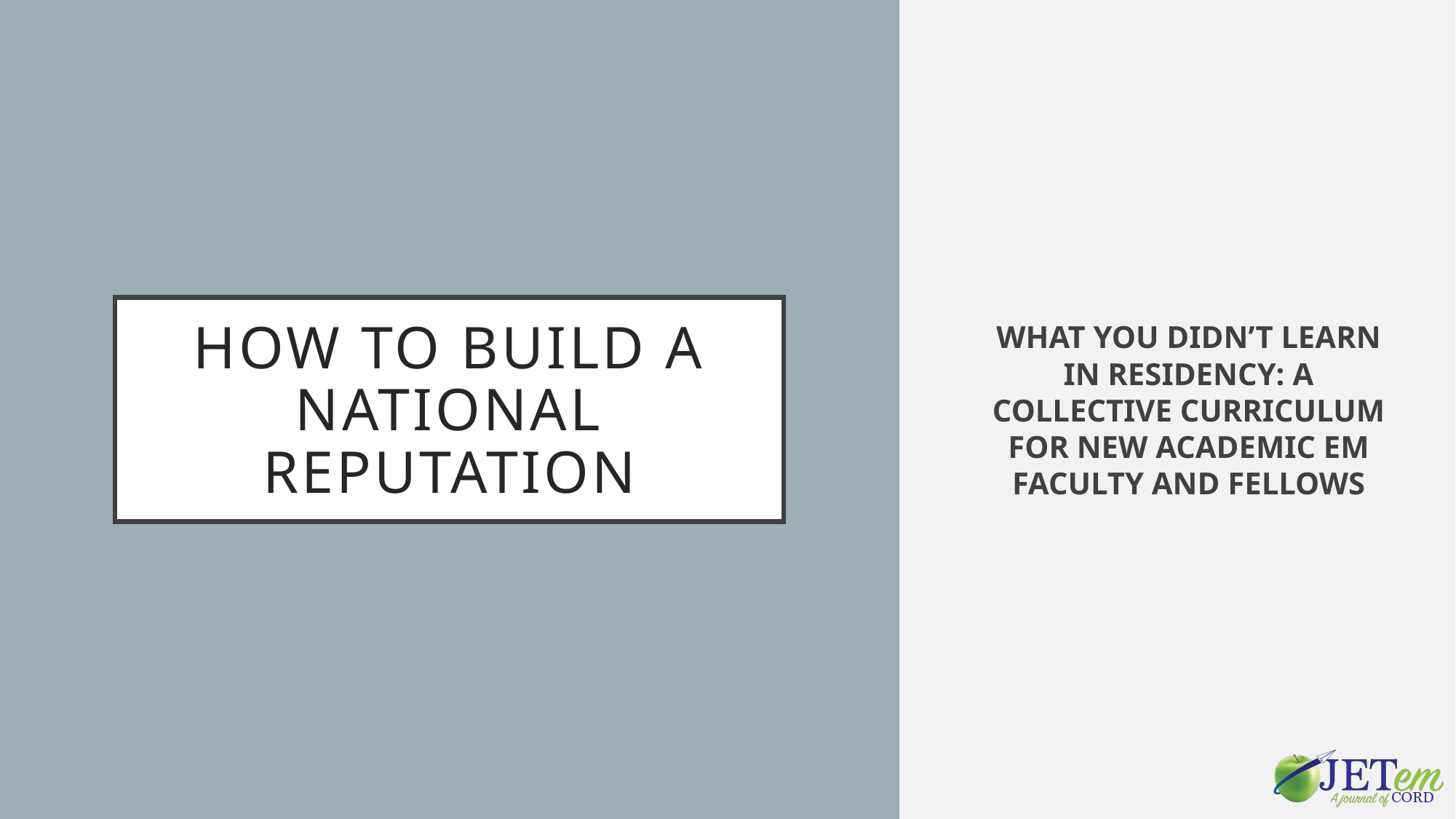

WHAT YOU DIDN’T LEARN IN RESIDENCY: A COLLECTIVE CURRICULUM FOR NEW ACADEMIC EM FACULTY AND FELLOWS
# HOW TO BUILD A NATIONAL REPUTATION

## Slide 2
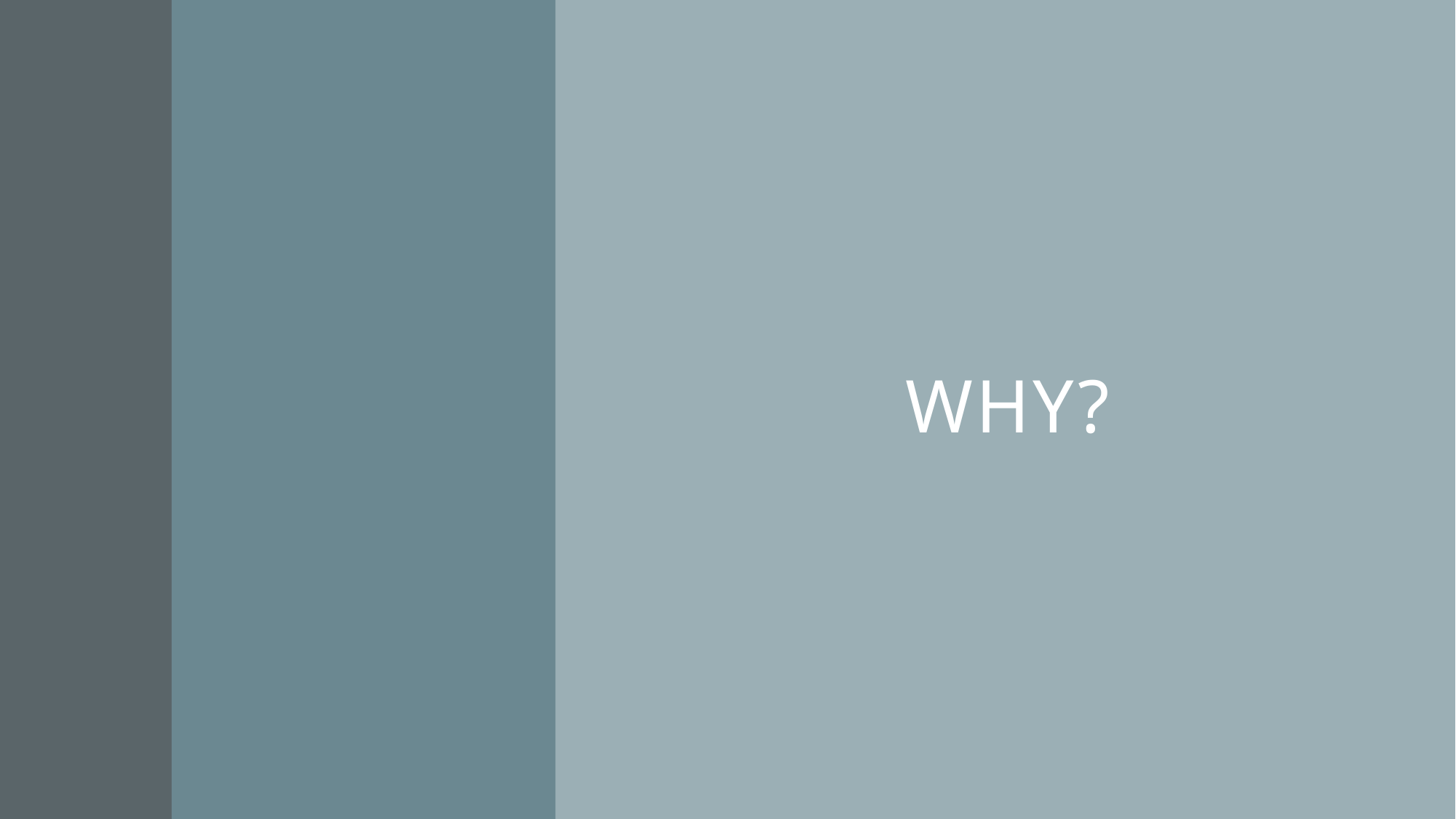

Why?

## Slide 3
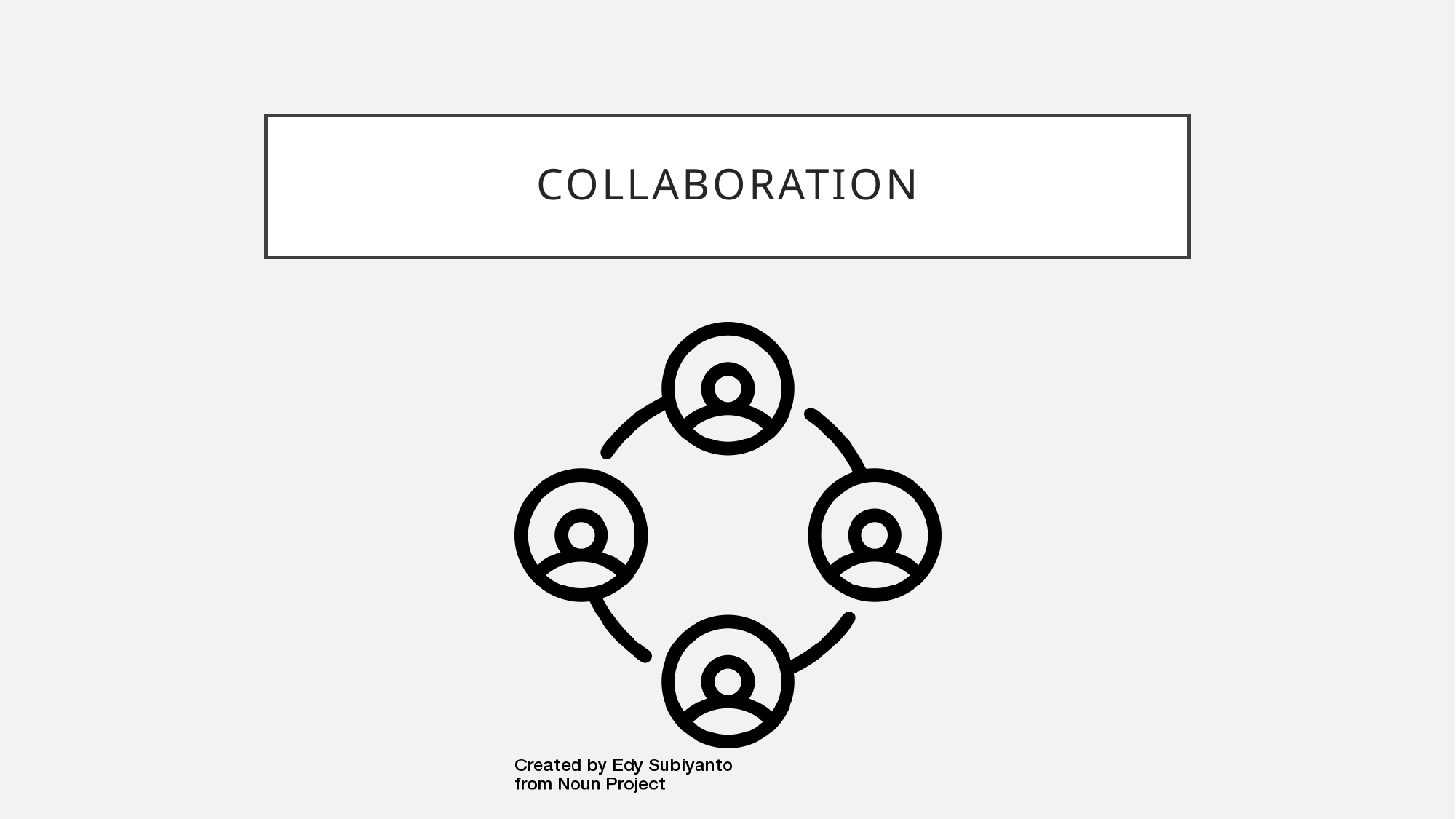

# Collaboration

## Slide 4
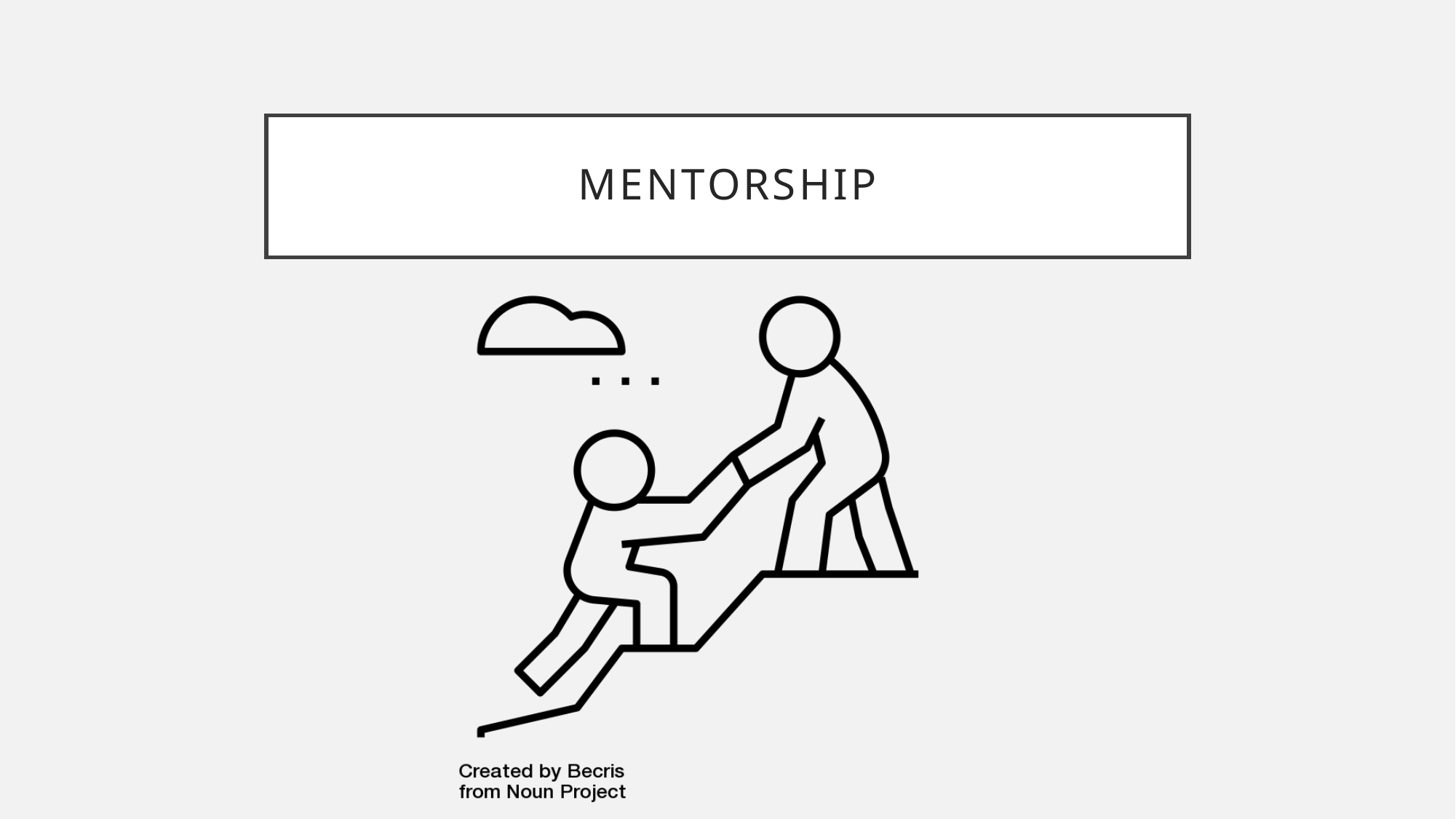

# Mentorship

## Slide 5
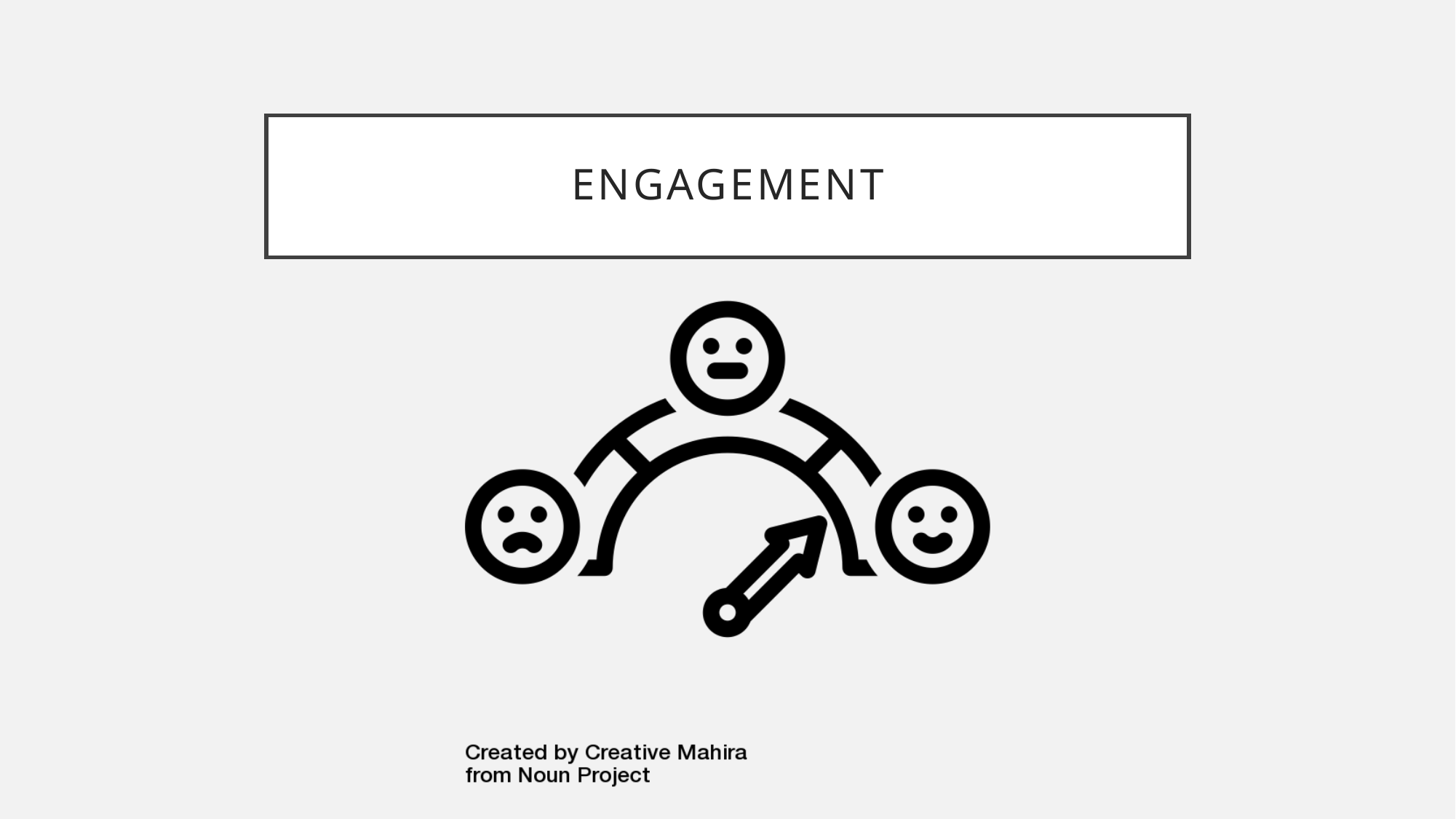

# Engagement

## Slide 6
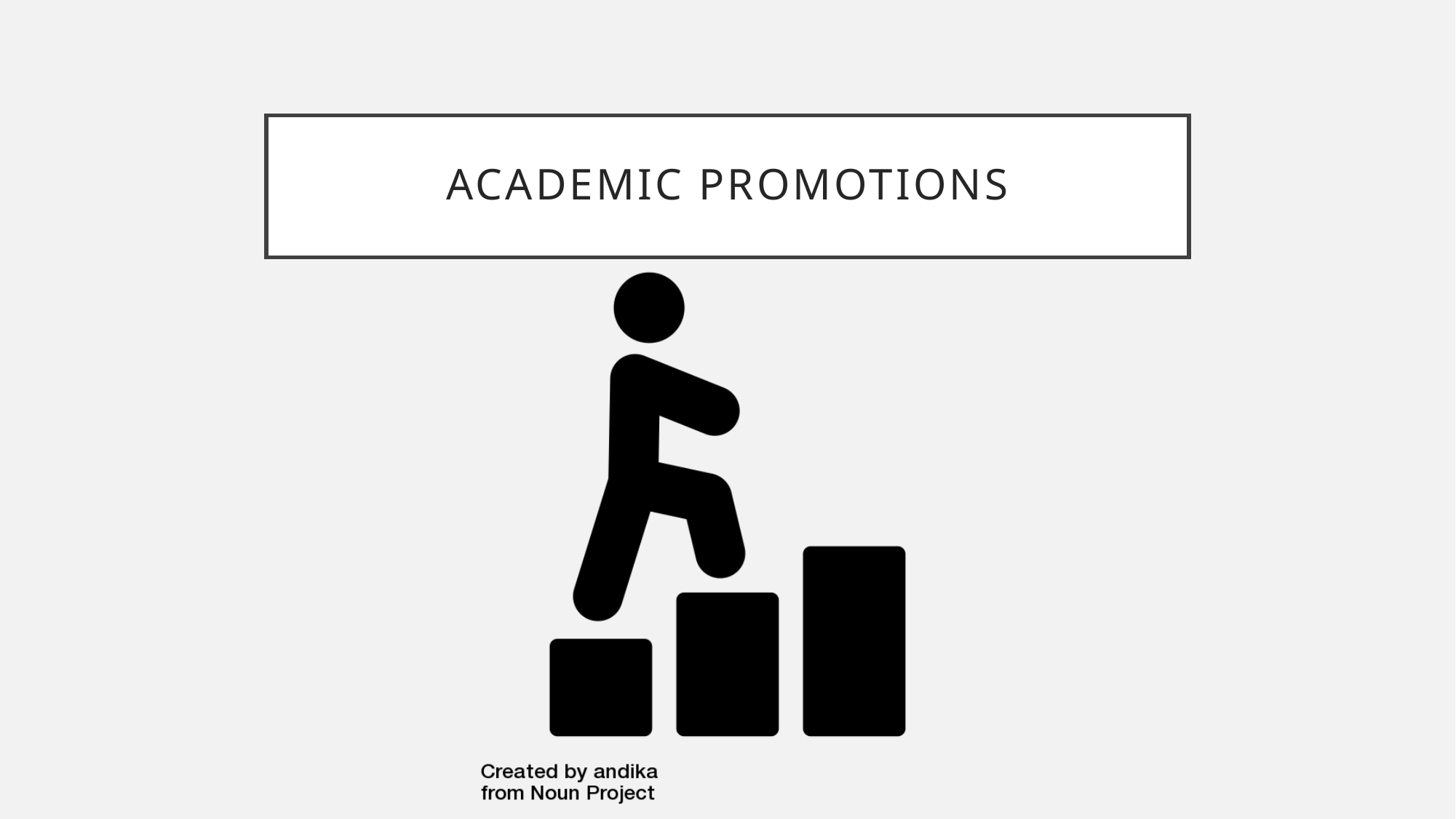

# Academic Promotions

## Slide 7
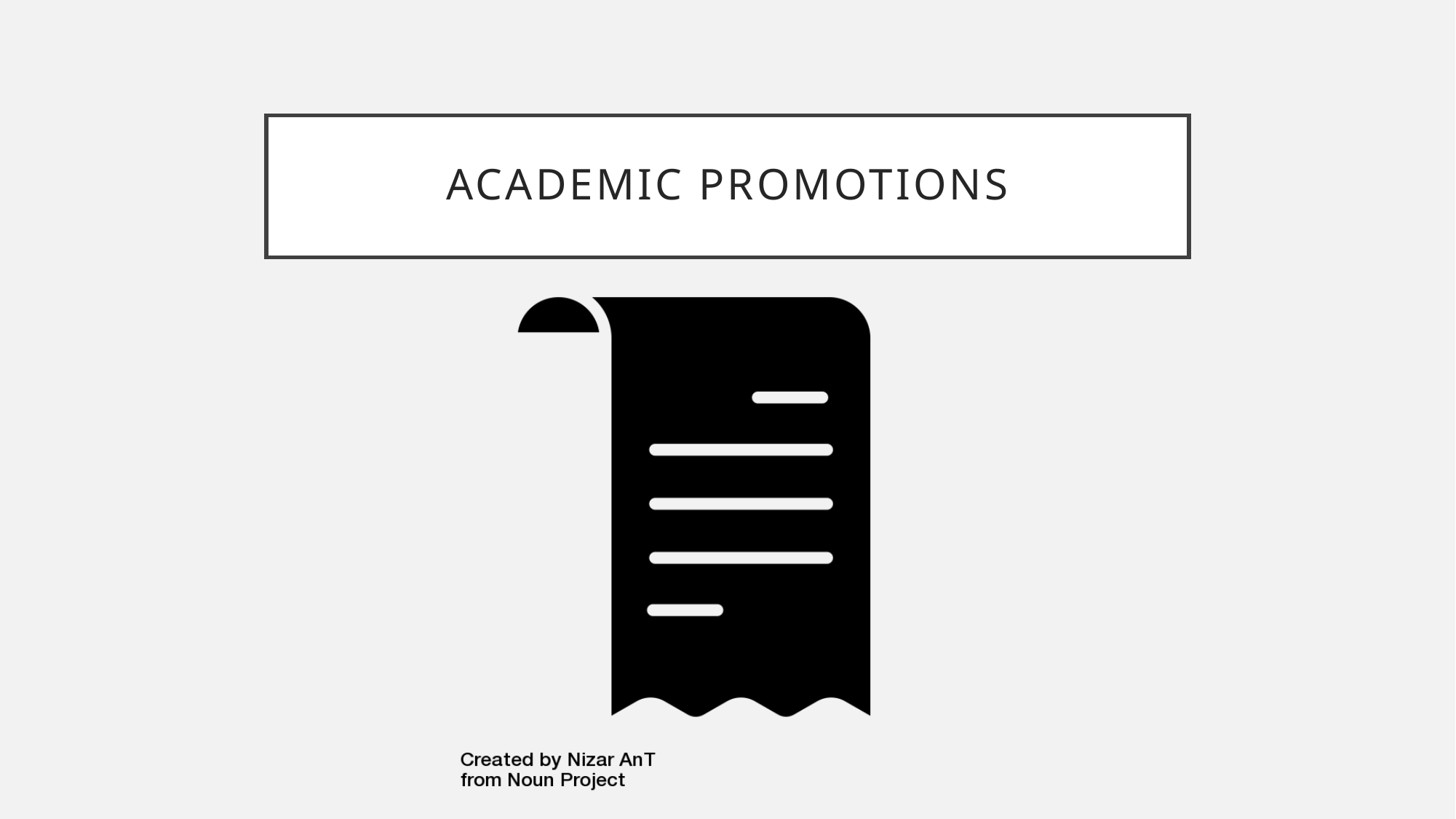

# Academic Promotions

## Slide 8
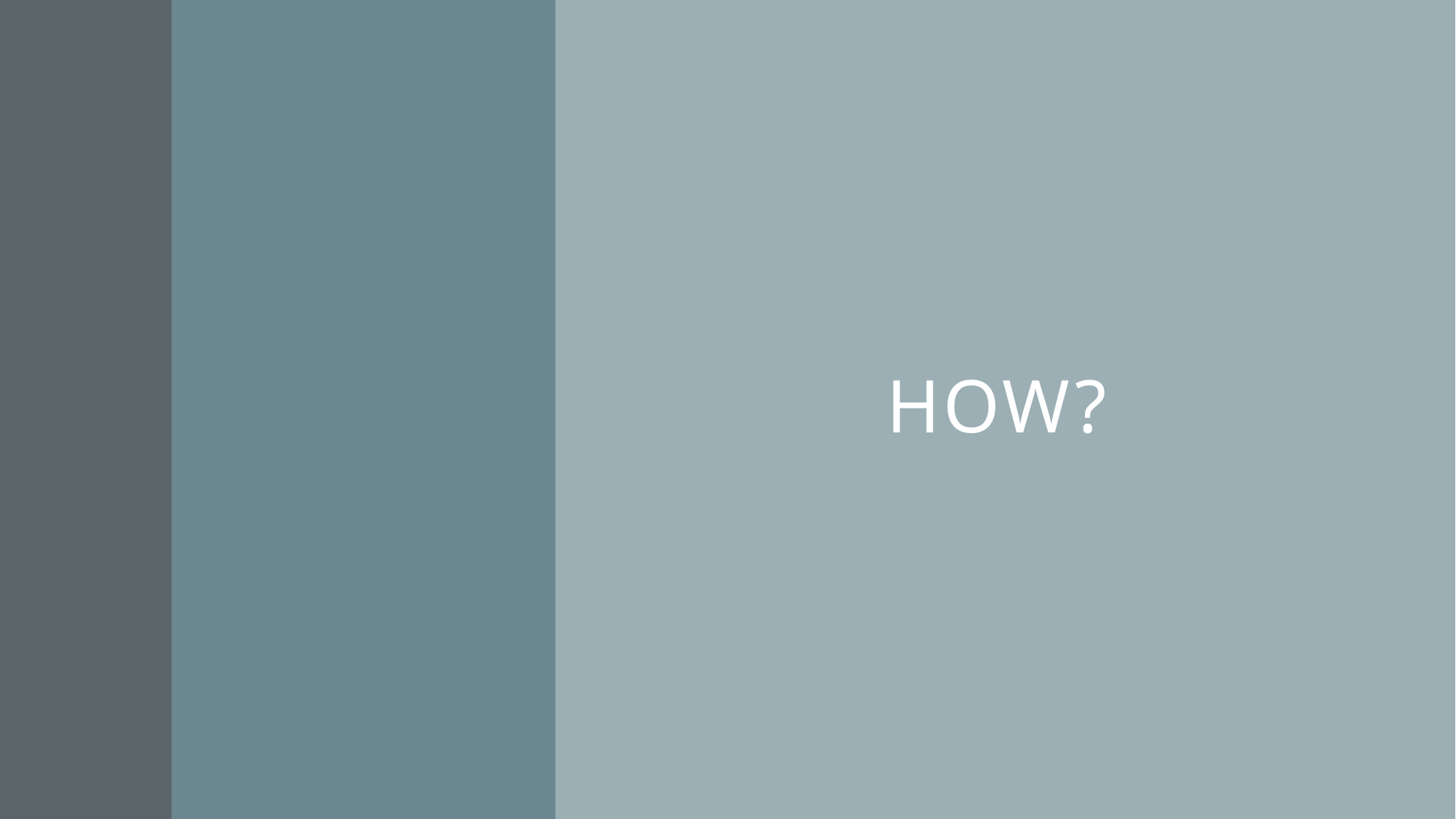

# How?

## Slide 9
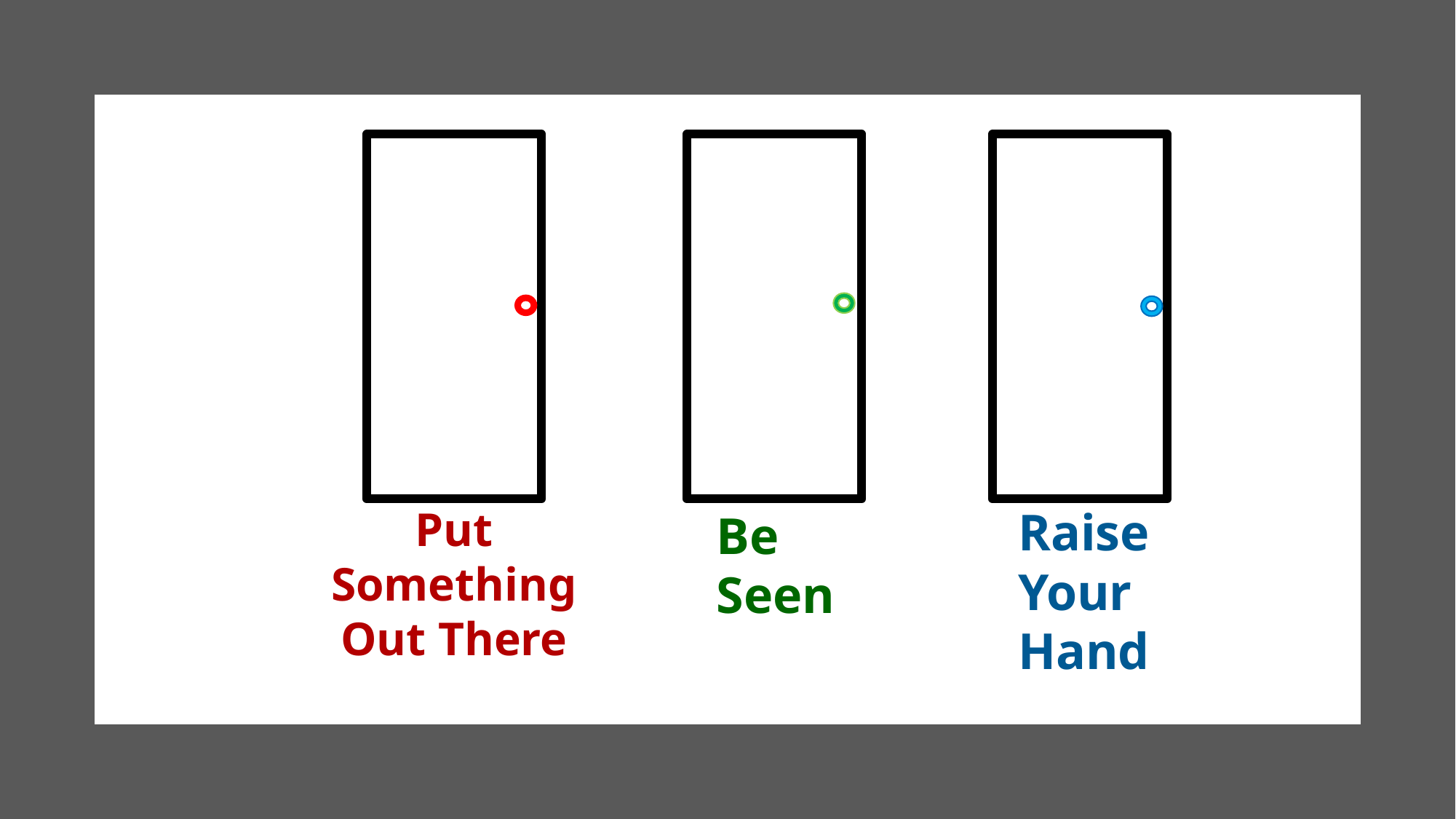

Put Something Out There
Raise Your Hand
Be Seen

## Slide 10
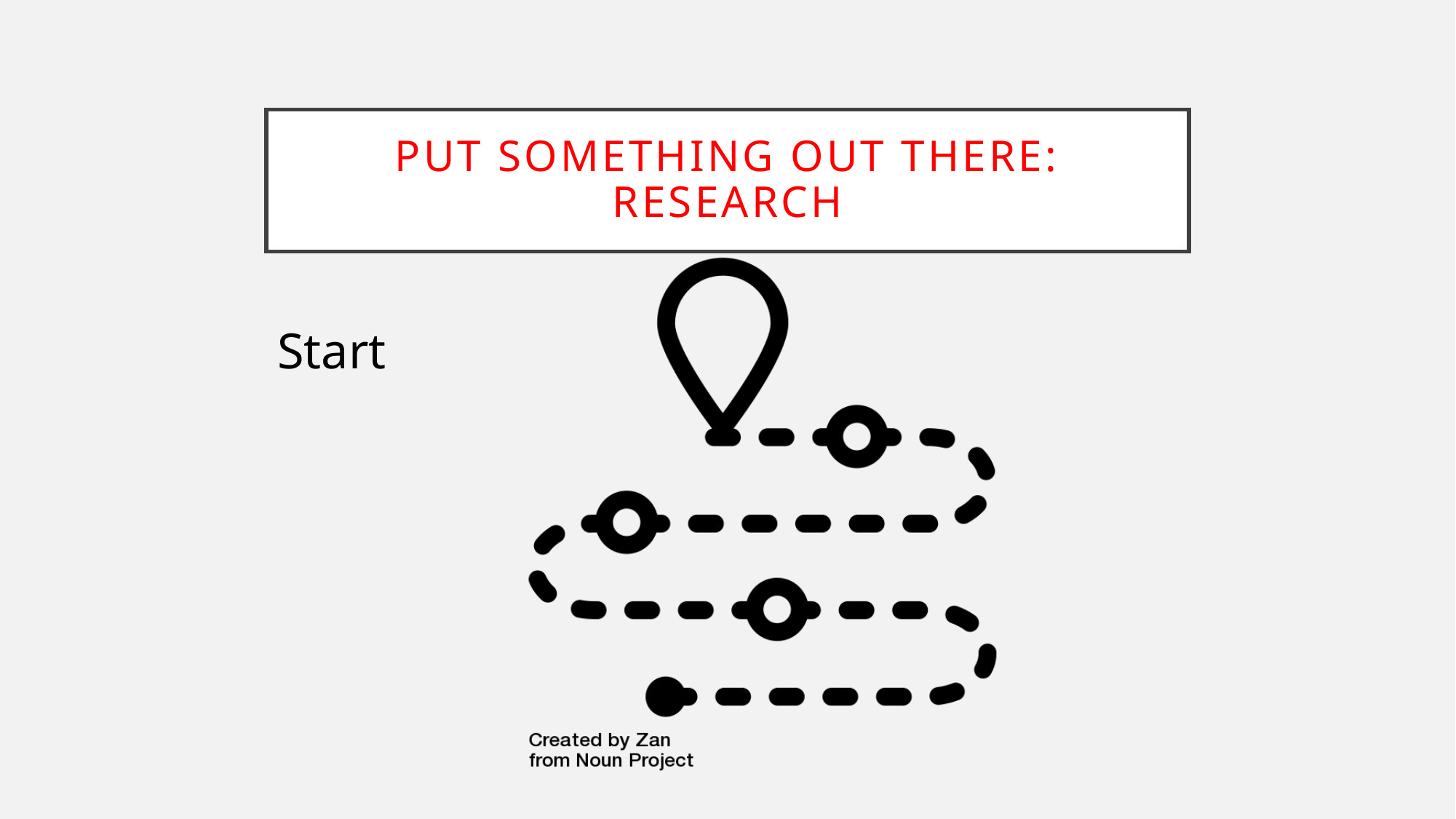

Put Something out There: Research
Start

## Slide 11
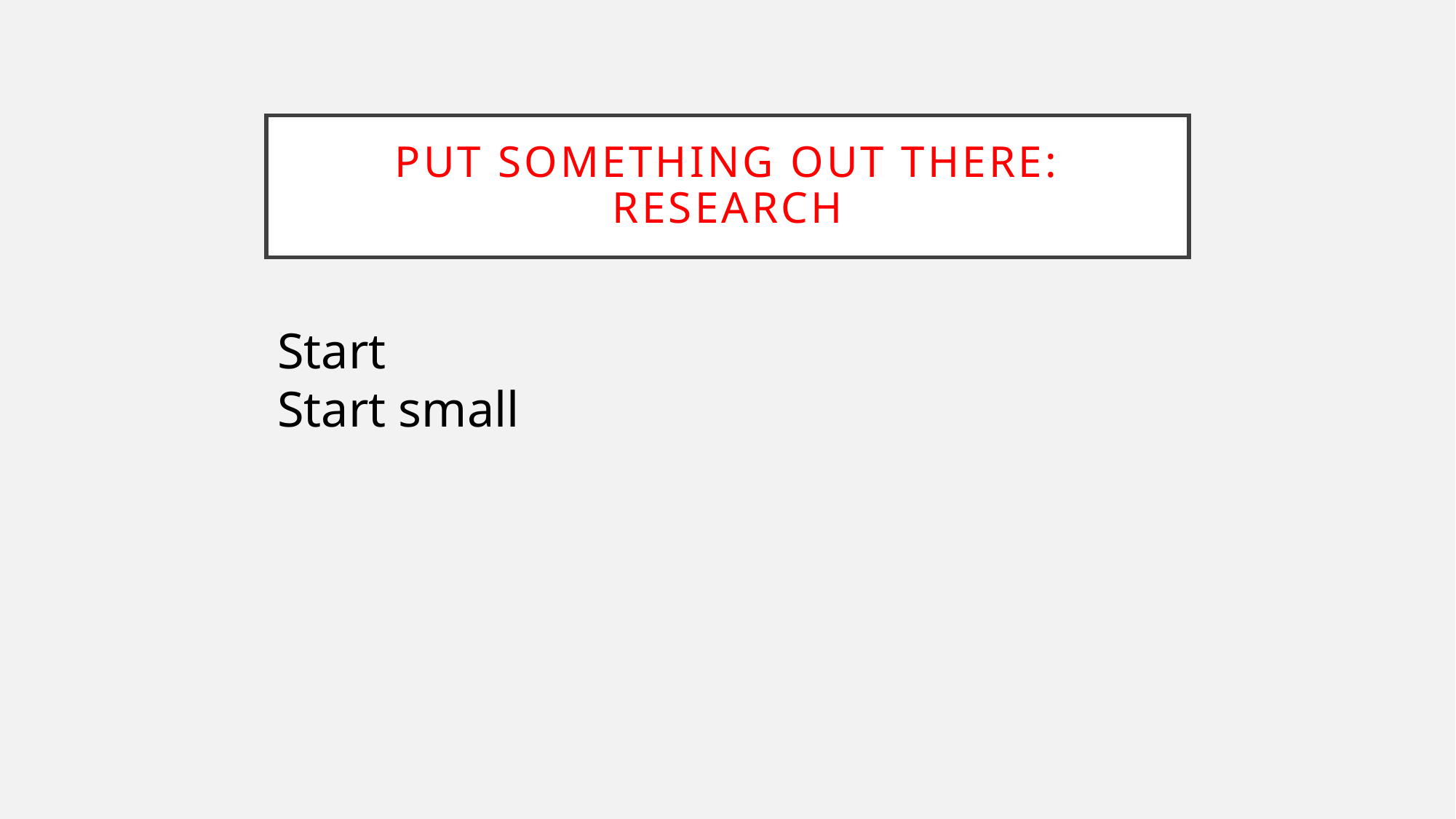

# Put Something out there: Research
Start
Start small

## Slide 12
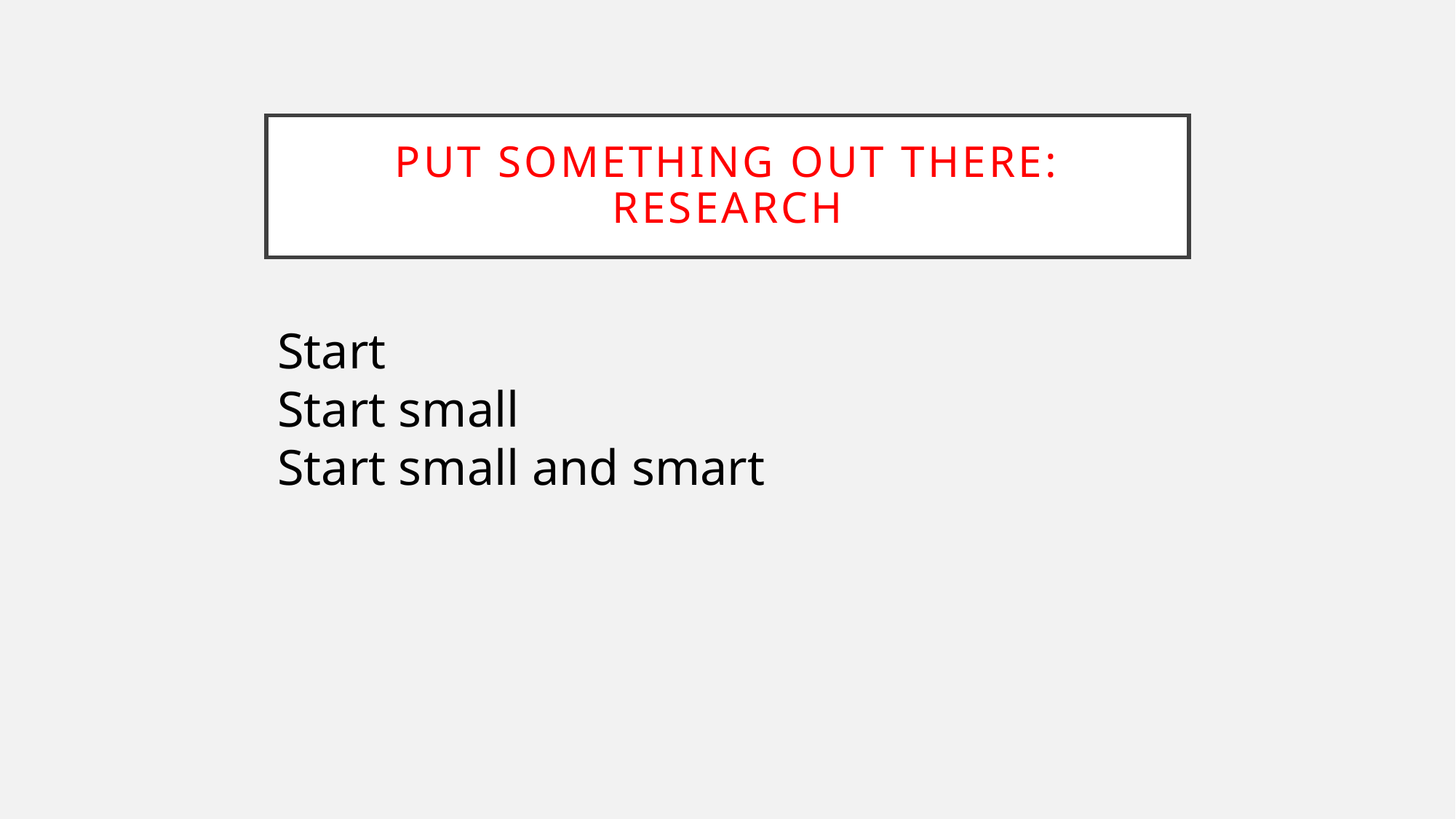

# Put something Out There: Research
Start
Start small
Start small and smart

## Slide 13
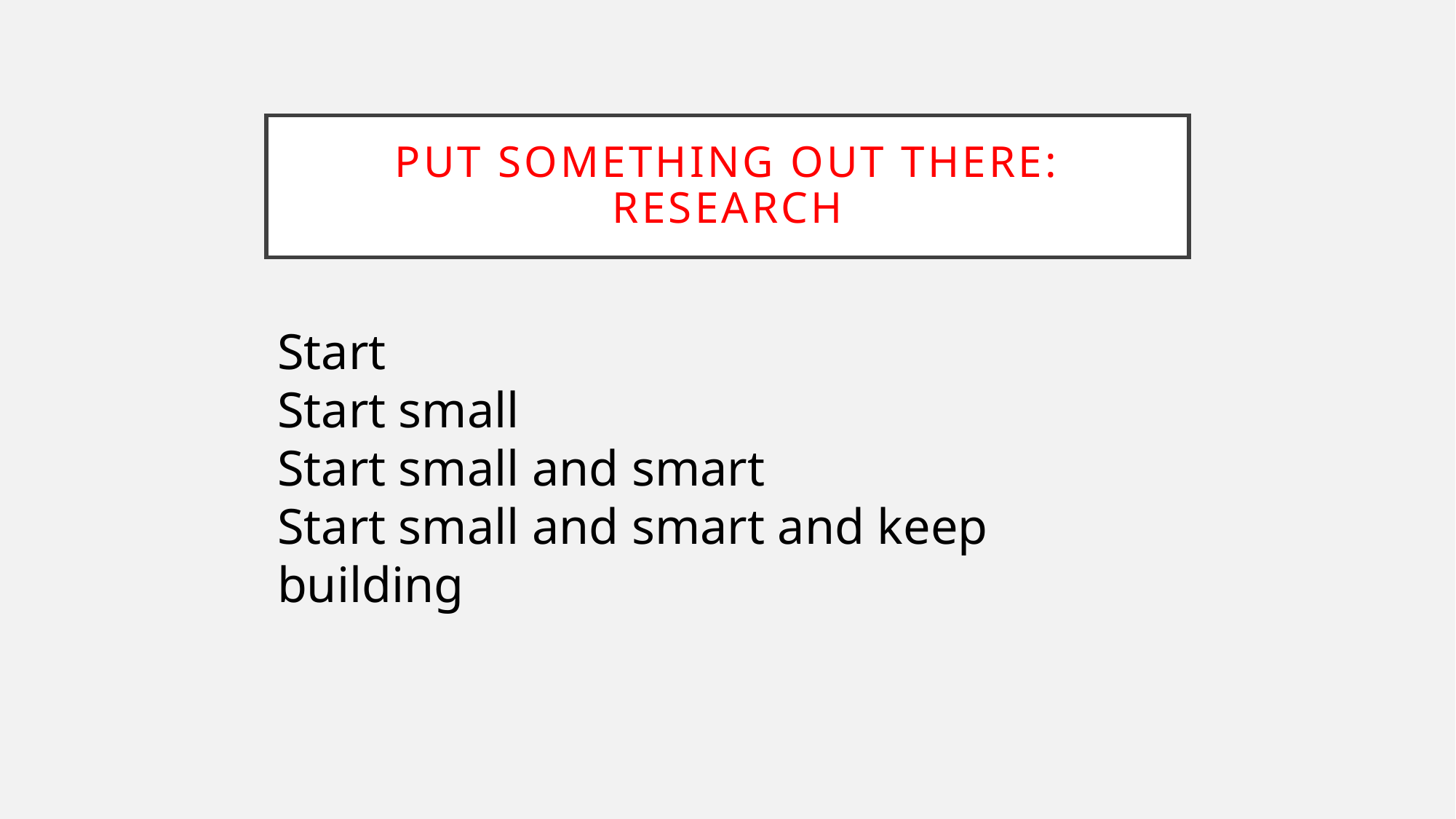

# Put something out there: Research
Start
Start small
Start small and smart
Start small and smart and keep building

## Slide 14
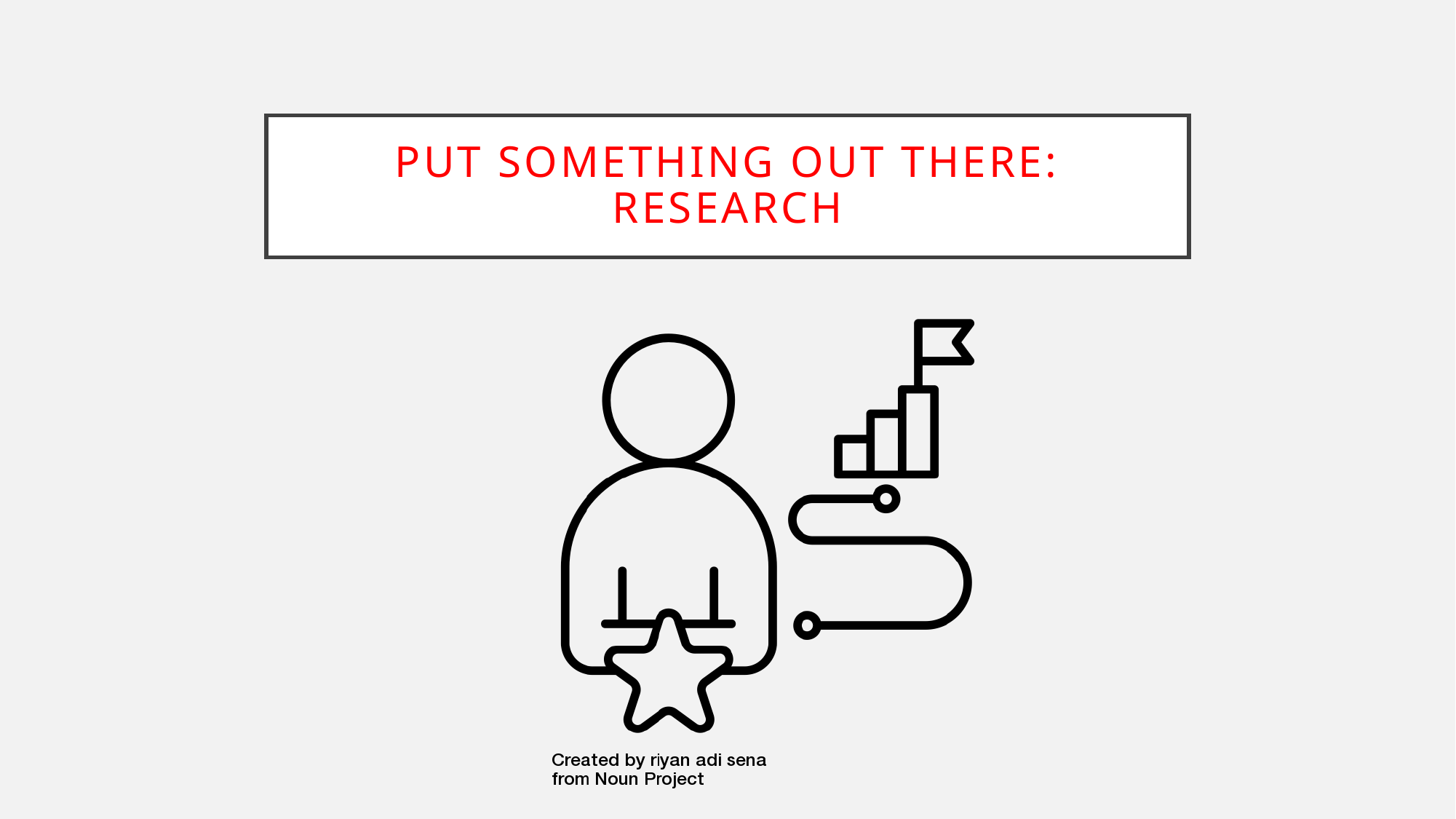

# Put something out There: Research

## Slide 15
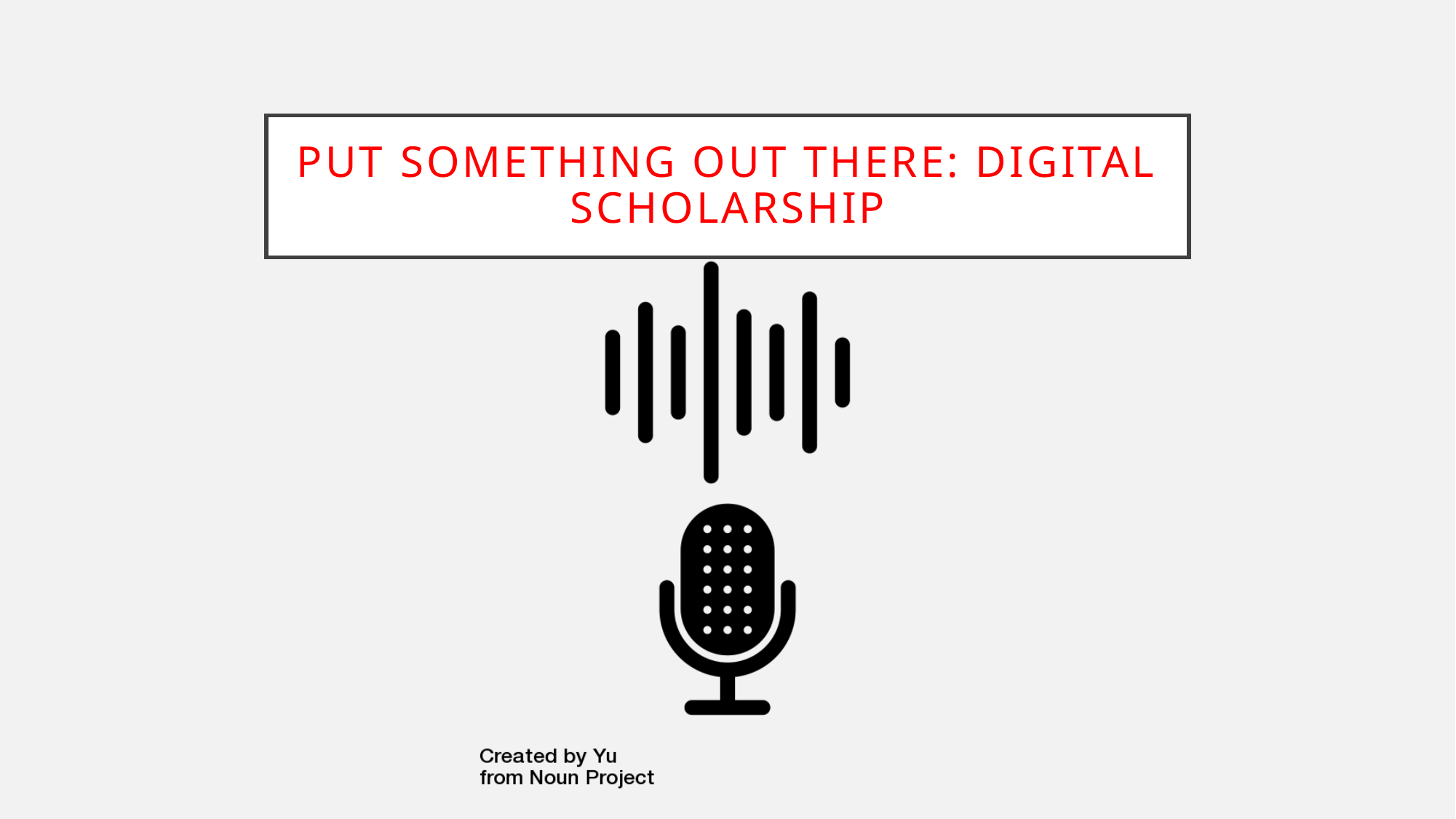

# Put Something out There: Digital Scholarship

## Slide 16
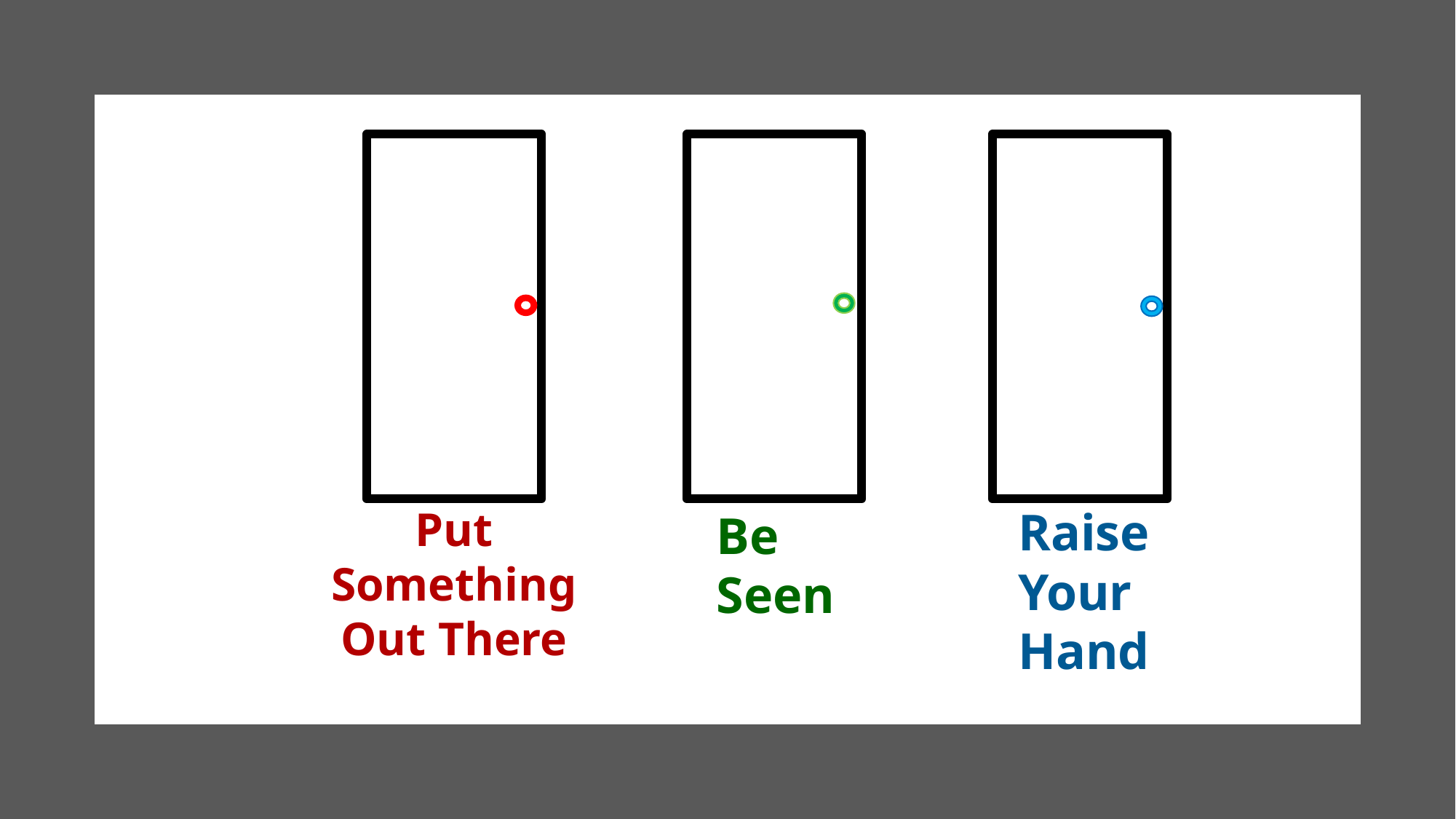

Put Something Out There
Raise Your Hand
Be Seen

## Slide 17
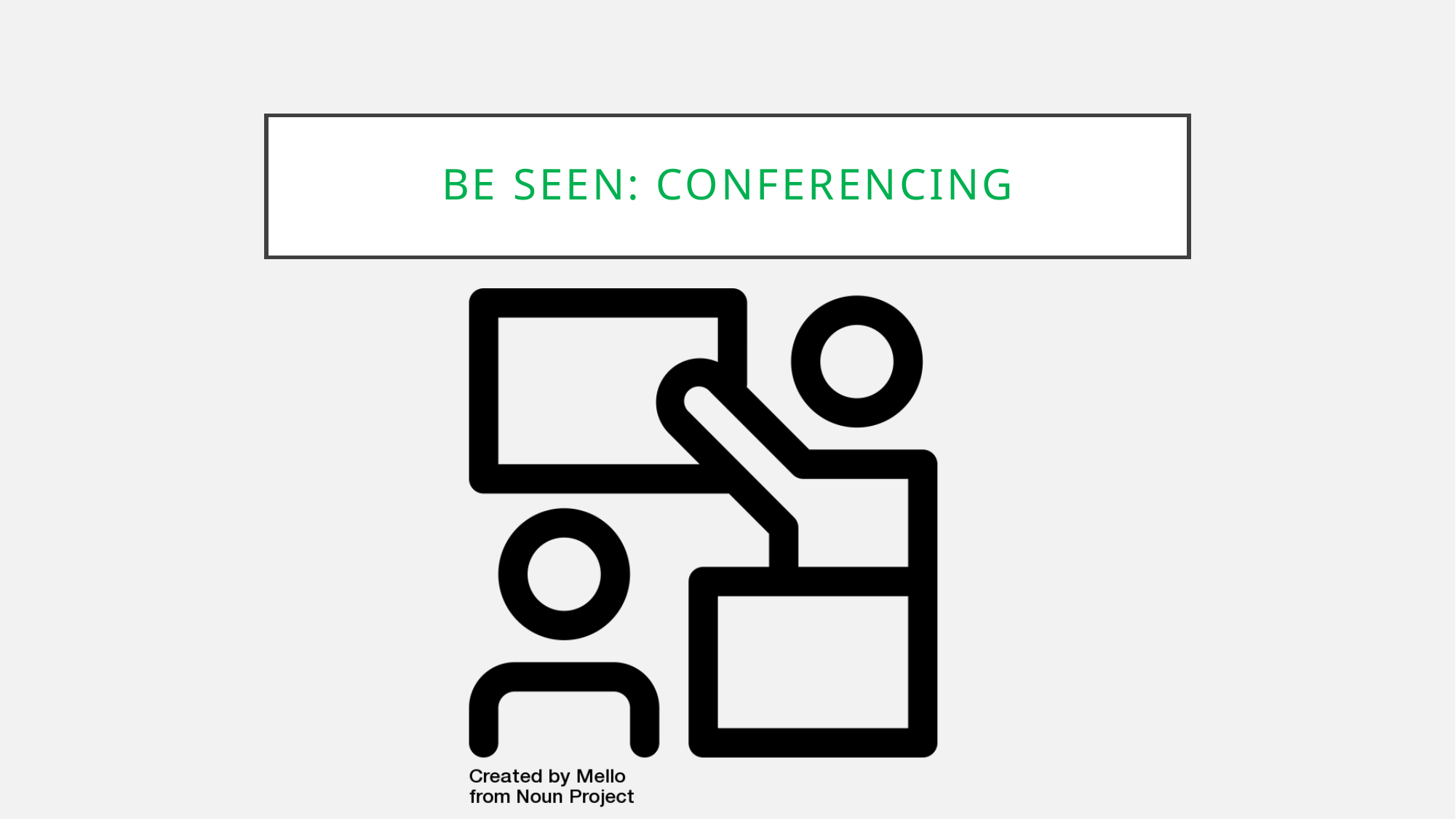

# Be Seen: Conferencing

## Slide 18
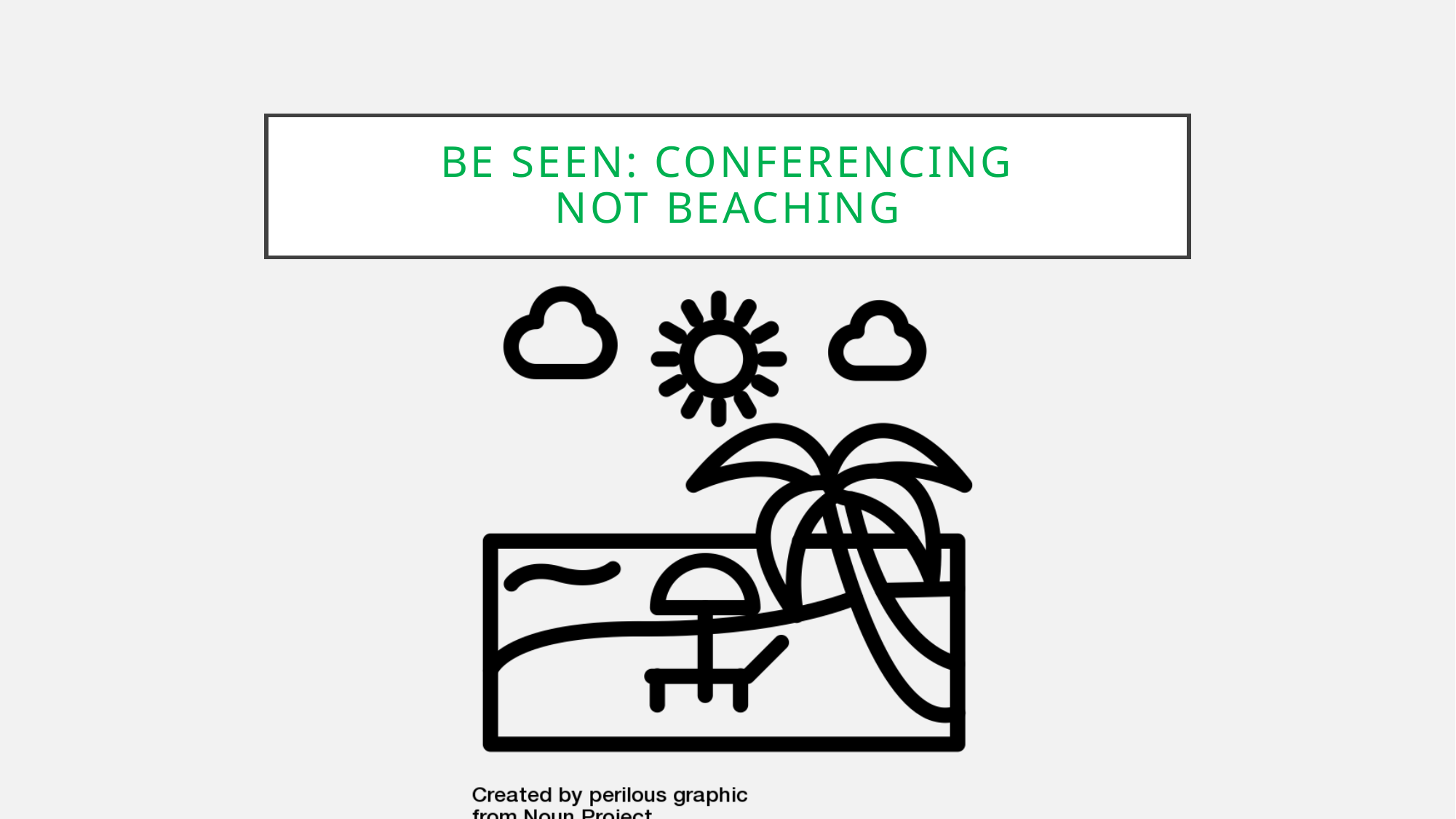

# Be Seen: Conferencing Not Beaching

## Slide 19
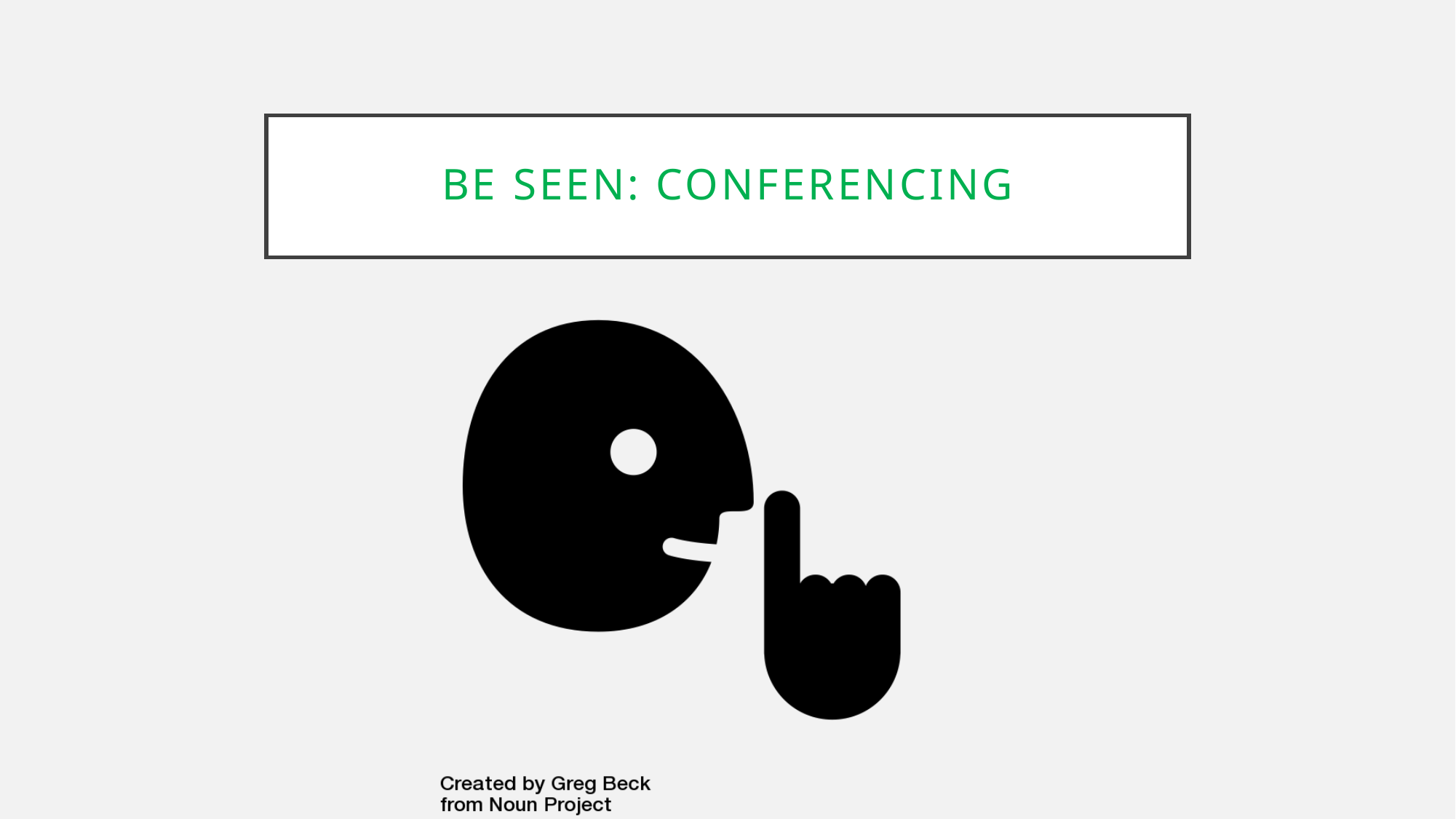

# Be SEEN: Conferencing

## Slide 20
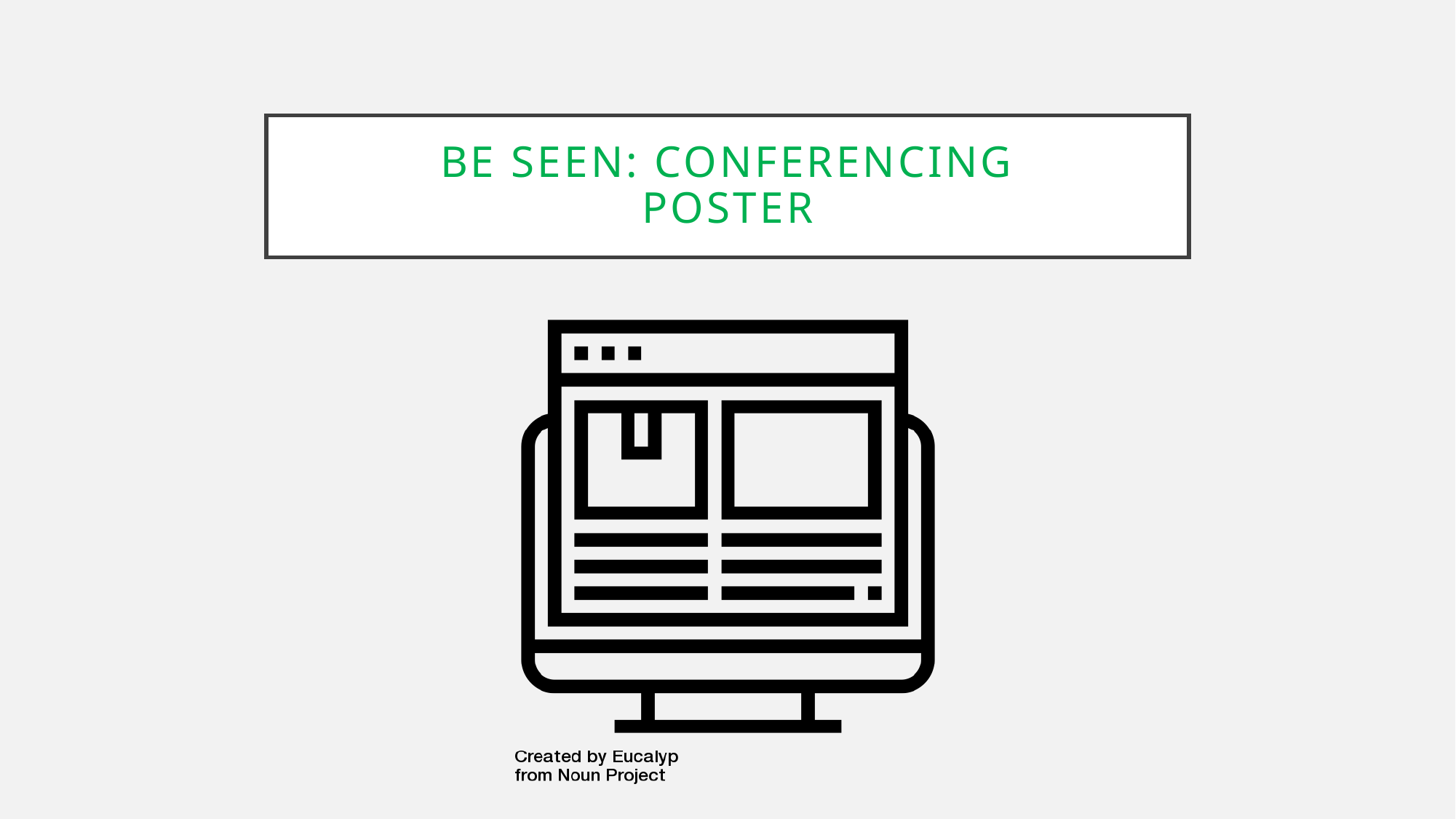

# Be Seen: Conferencing poster

## Slide 21
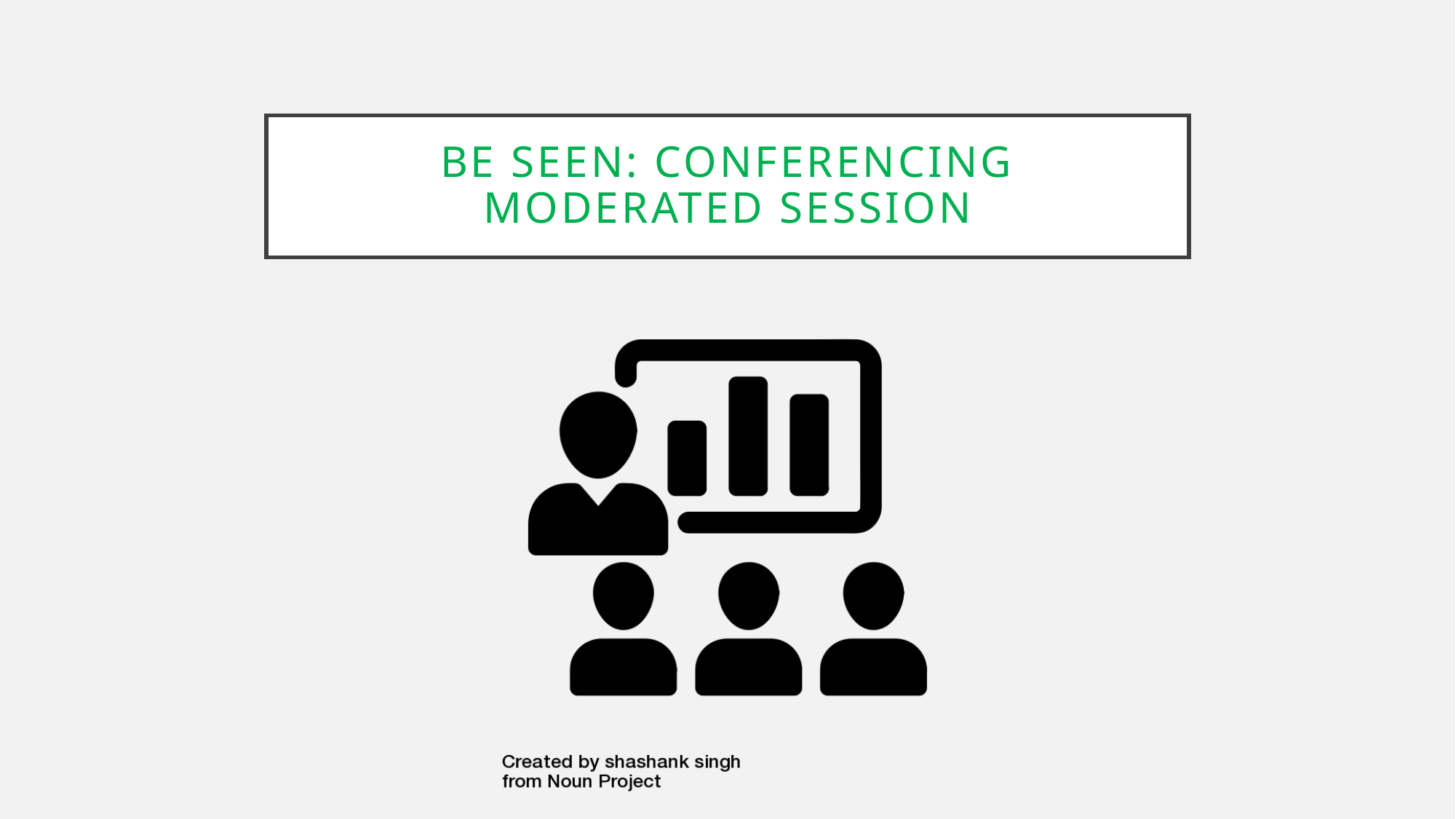

# BE SEEN: CONFERENCING Moderated Session

## Slide 22
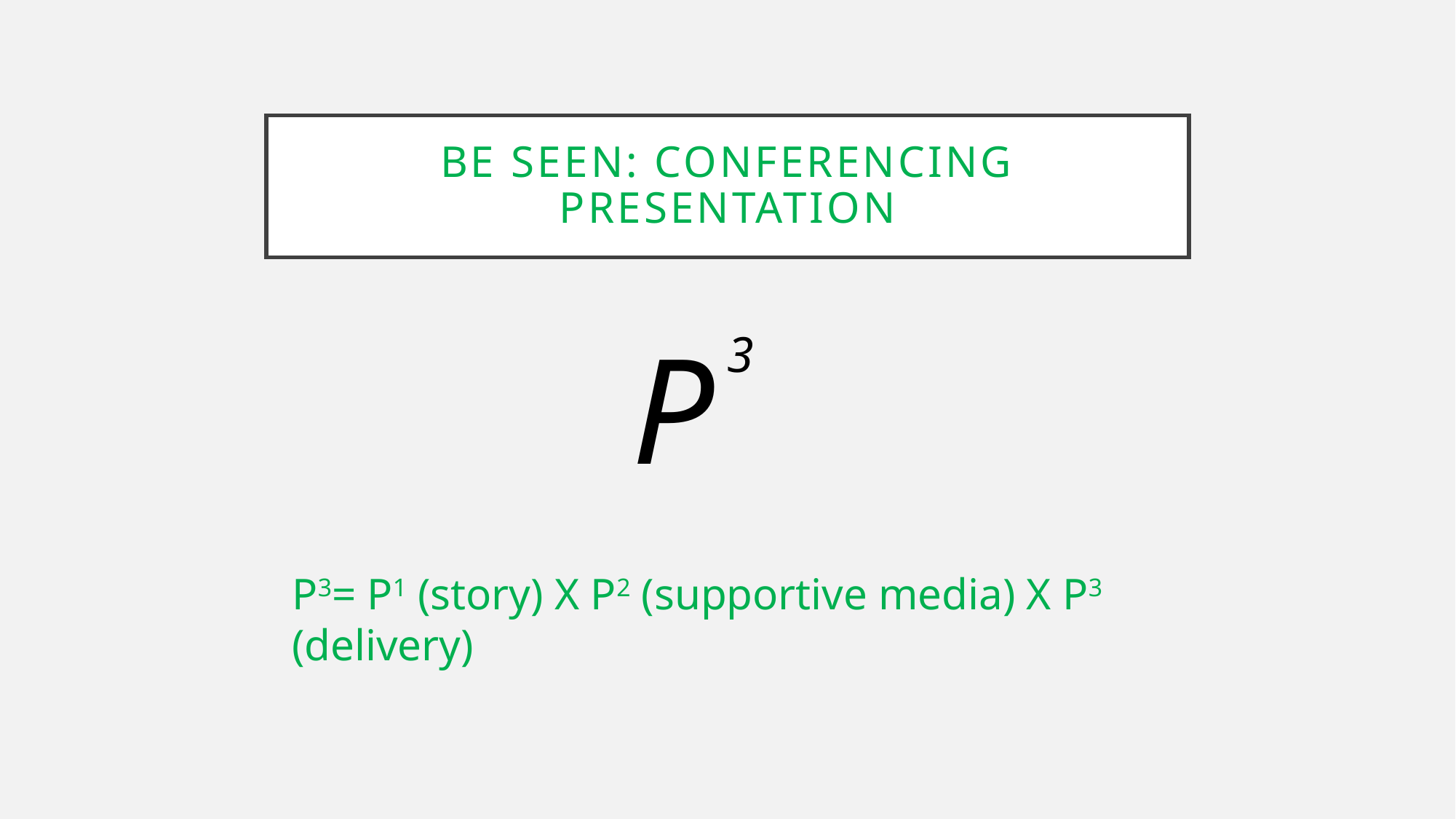

# Be SEEN: Conferencing Presentation
P
3
P3= P1 (story) X P2 (supportive media) X P3 (delivery)

## Slide 23
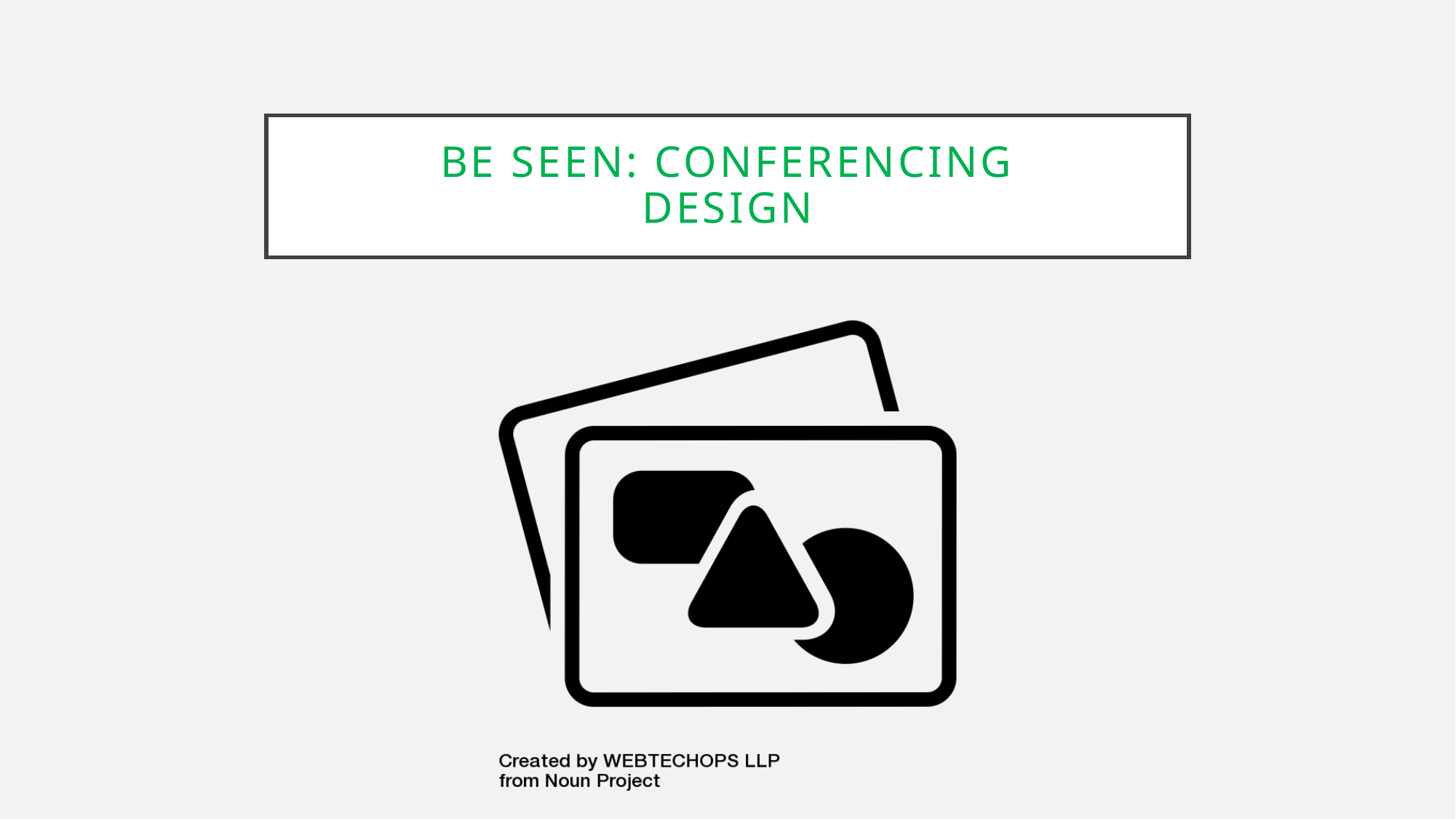

# Be Seen: Conferencing Design

## Slide 24
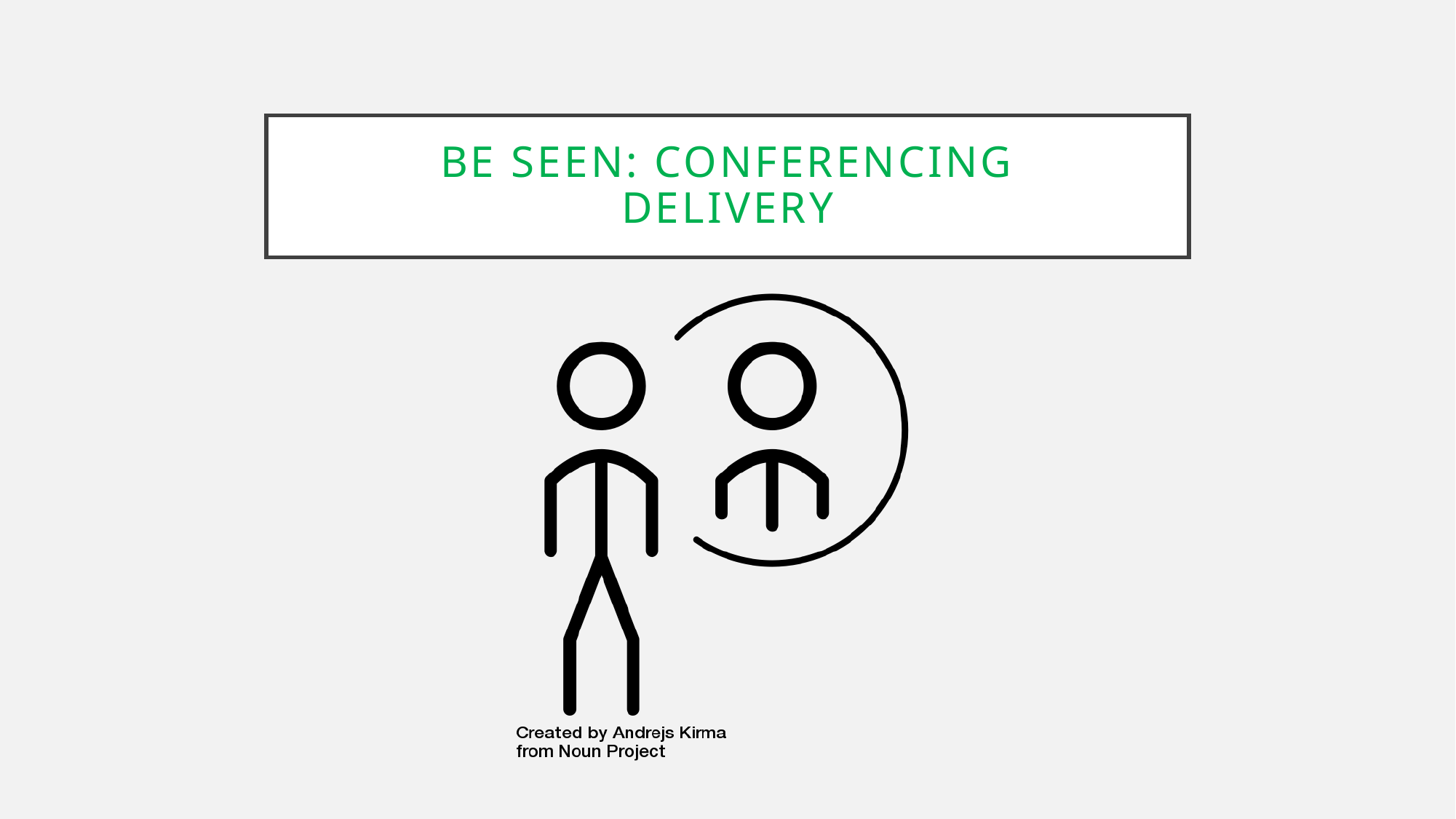

# Be Seen: Conferencing Delivery

## Slide 25
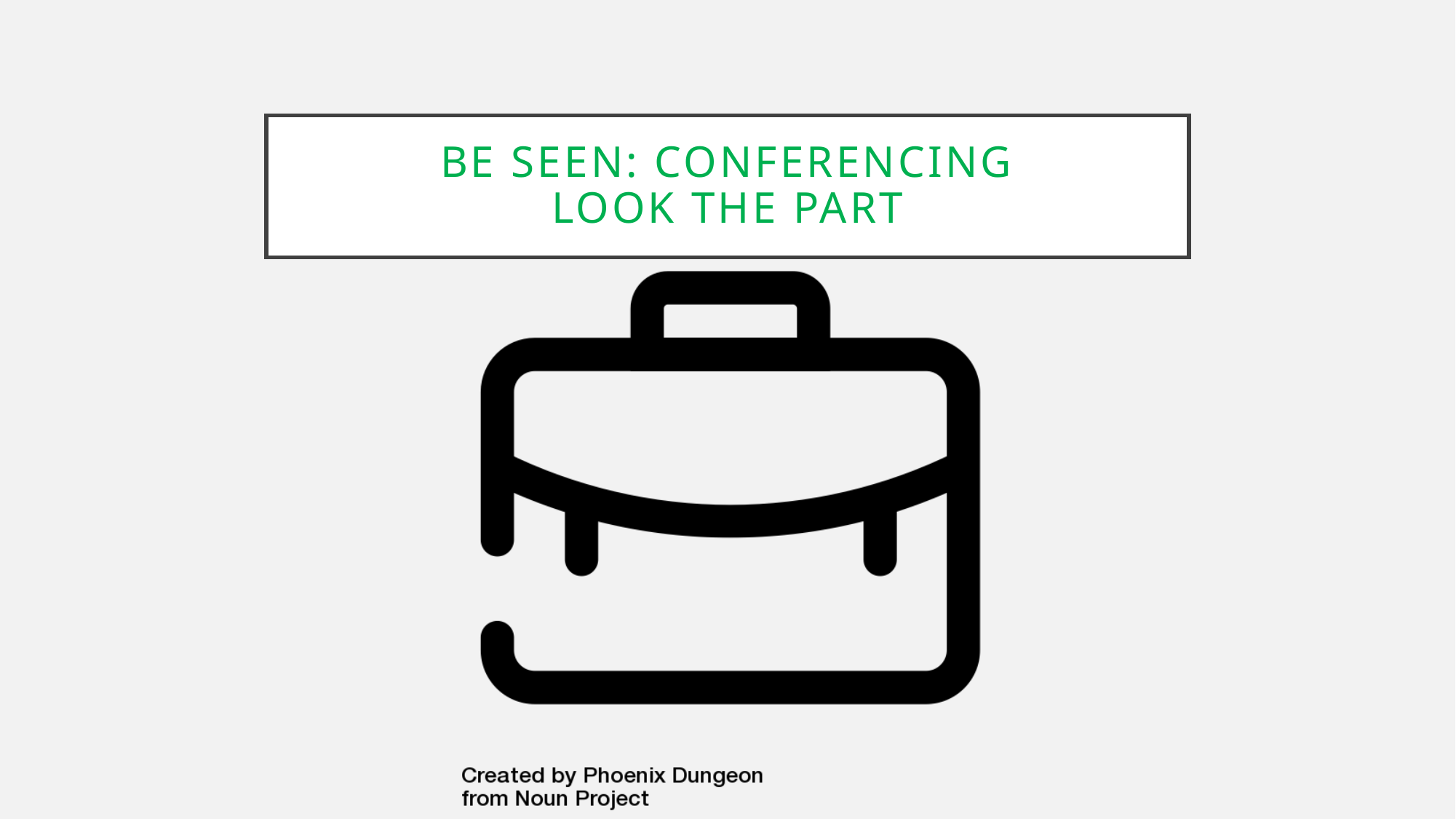

# Be SEEN: CONFERENCING look the part

## Slide 26
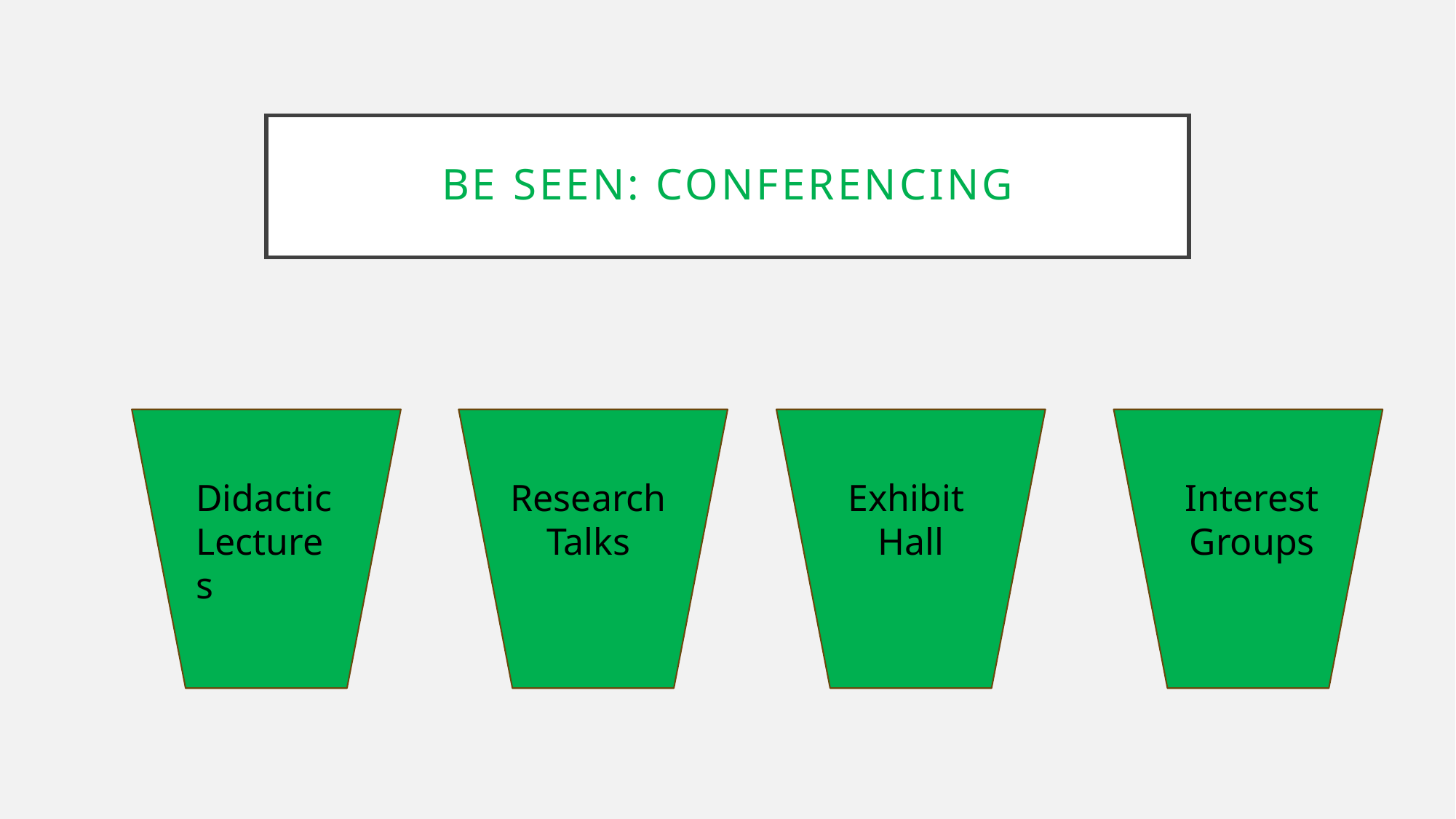

# Be SEEN: Conferencing
Research
Talks
Exhibit
Hall
Interest
Groups
Didactic Lectures

## Slide 27
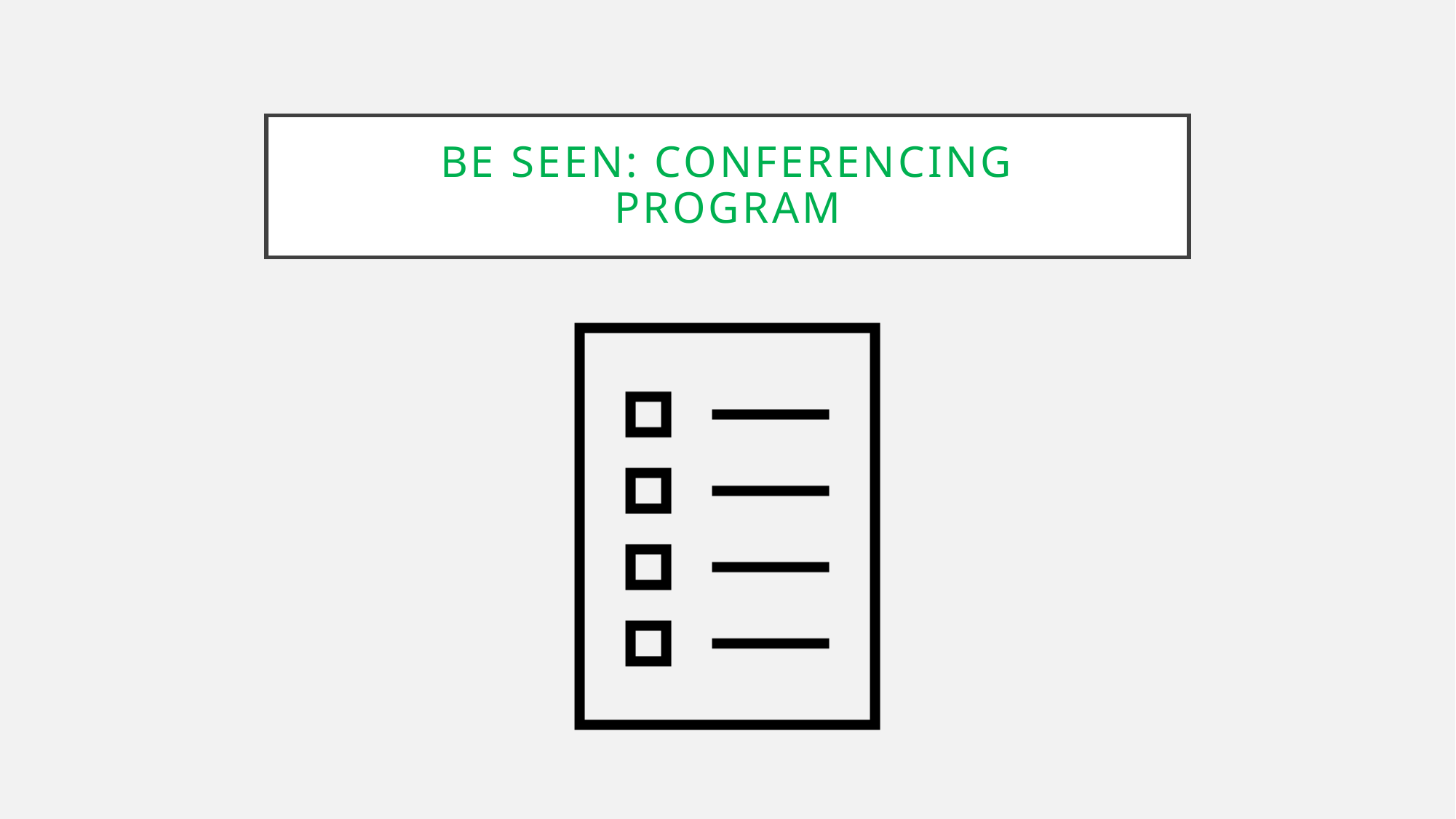

# Be Seen: CONFERENCING Program

## Slide 28
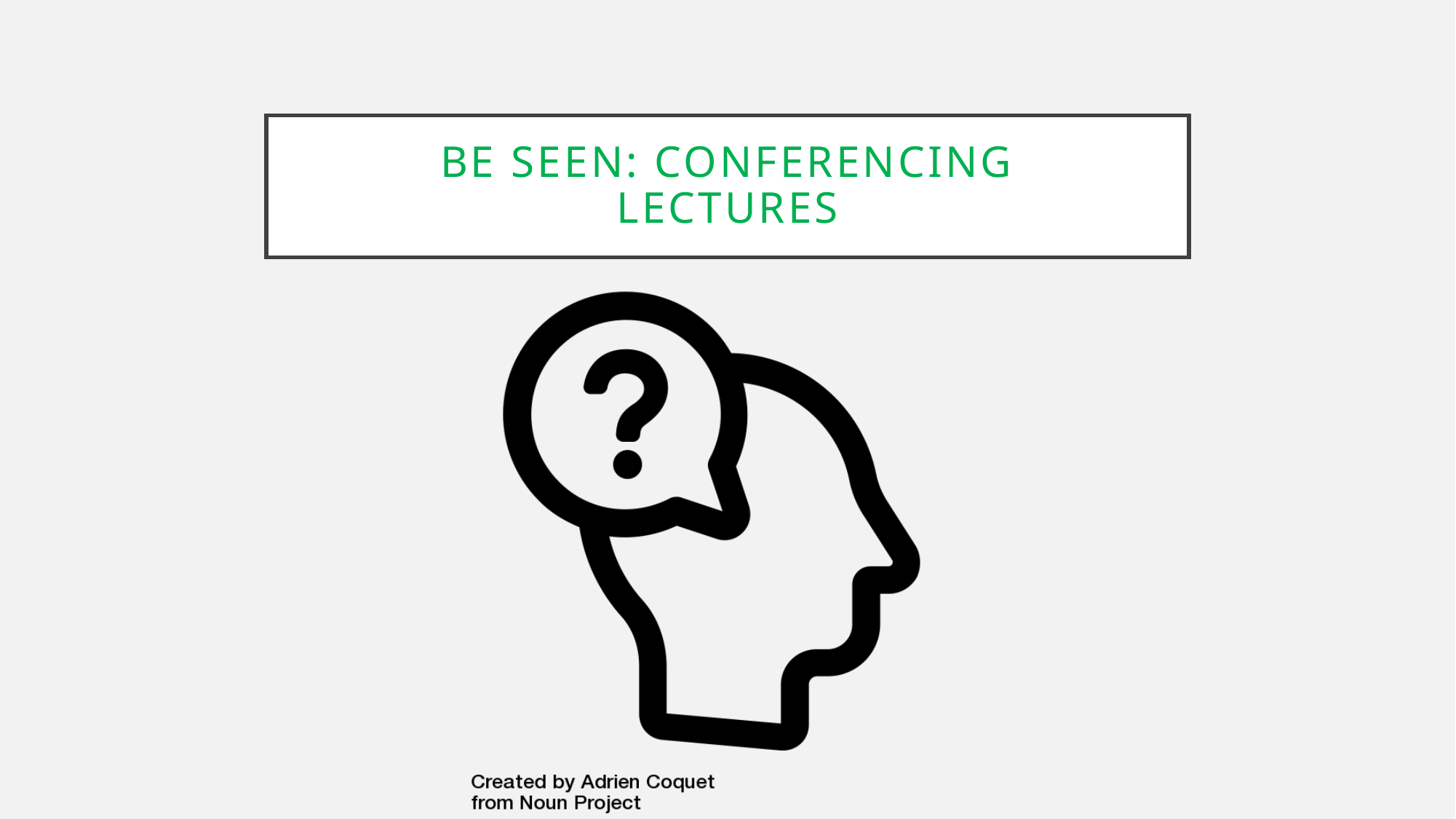

# Be SEEN: CONFERENCING Lectures

## Slide 29
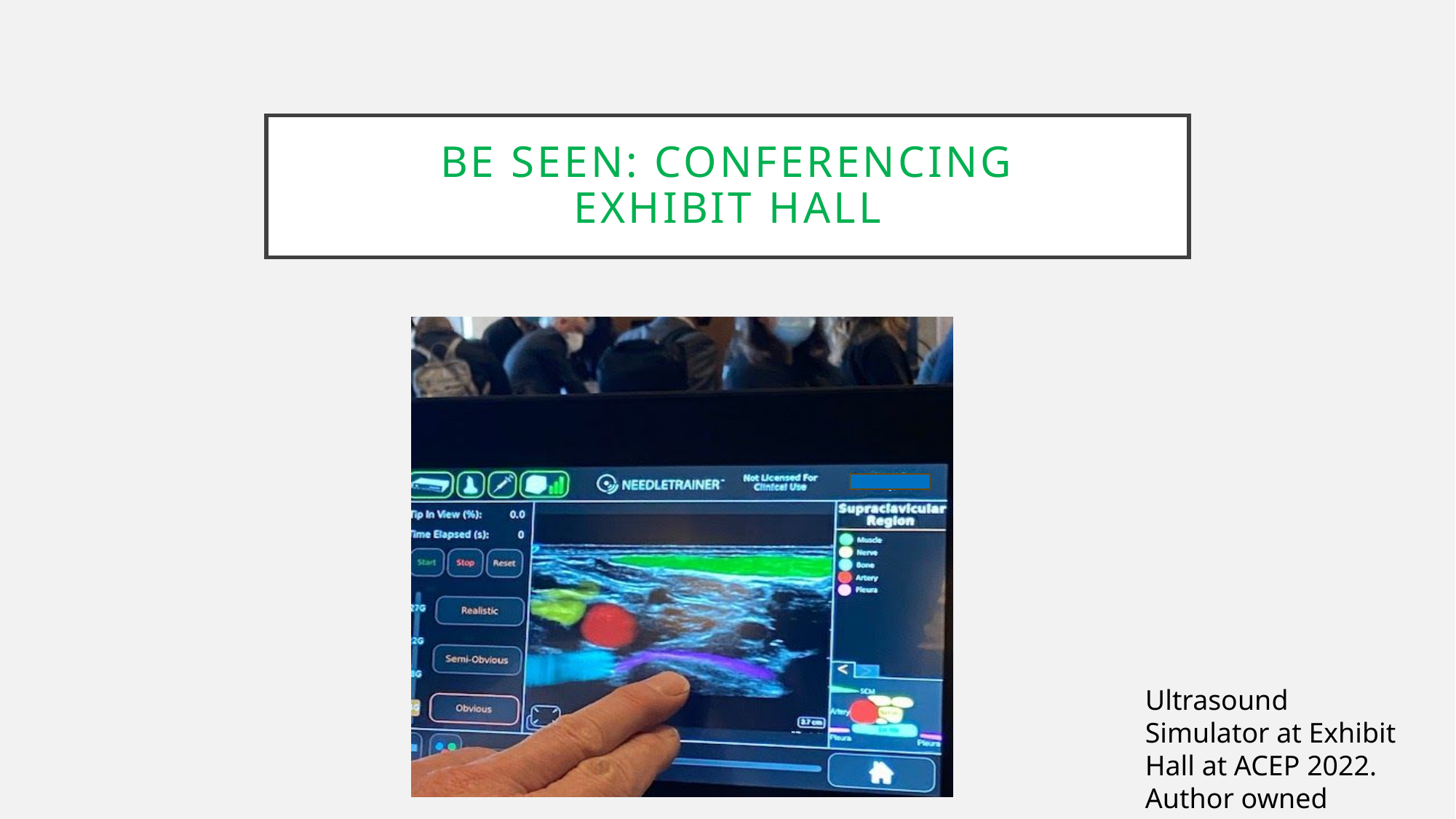

# Be SEEN: CONFERENCING Exhibit Hall
Ultrasound Simulator at Exhibit Hall at ACEP 2022. Author owned

## Slide 30
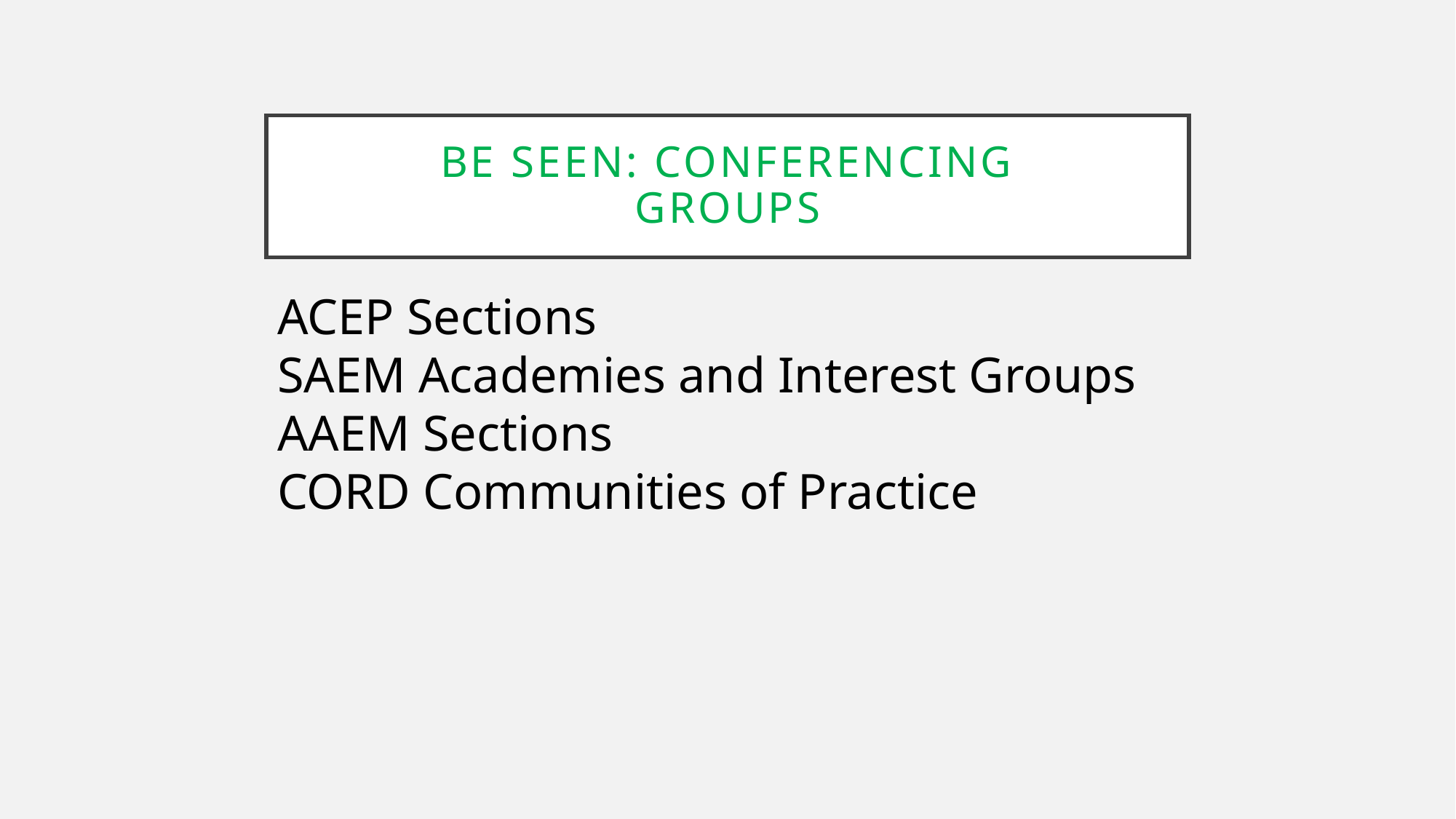

# Be SEEN: CONFERENCING Groups
ACEP Sections
SAEM Academies and Interest Groups
AAEM Sections
CORD Communities of Practice

## Slide 31
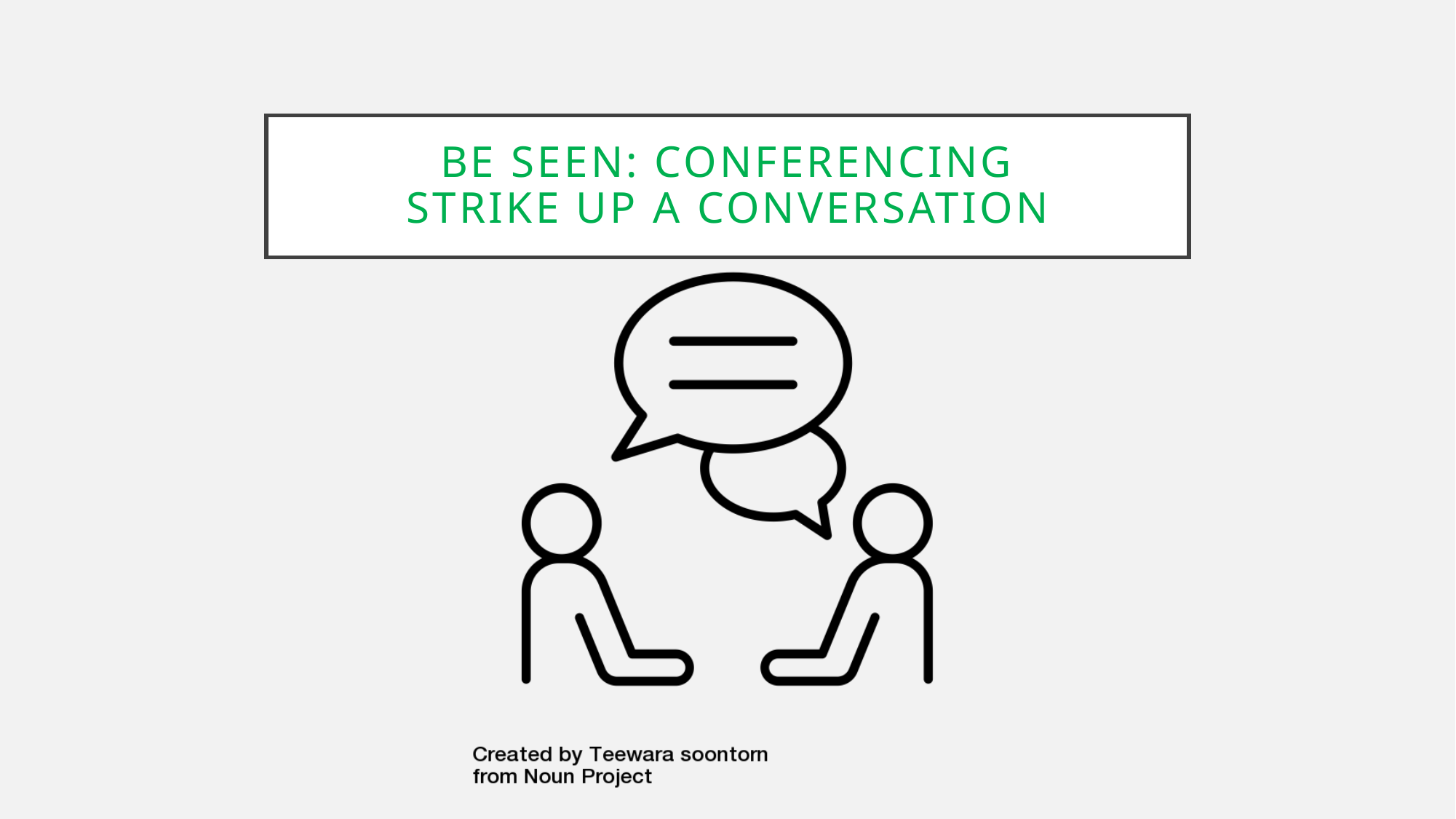

# Be Seen: CONFERENCING Strike up a conversation

## Slide 32
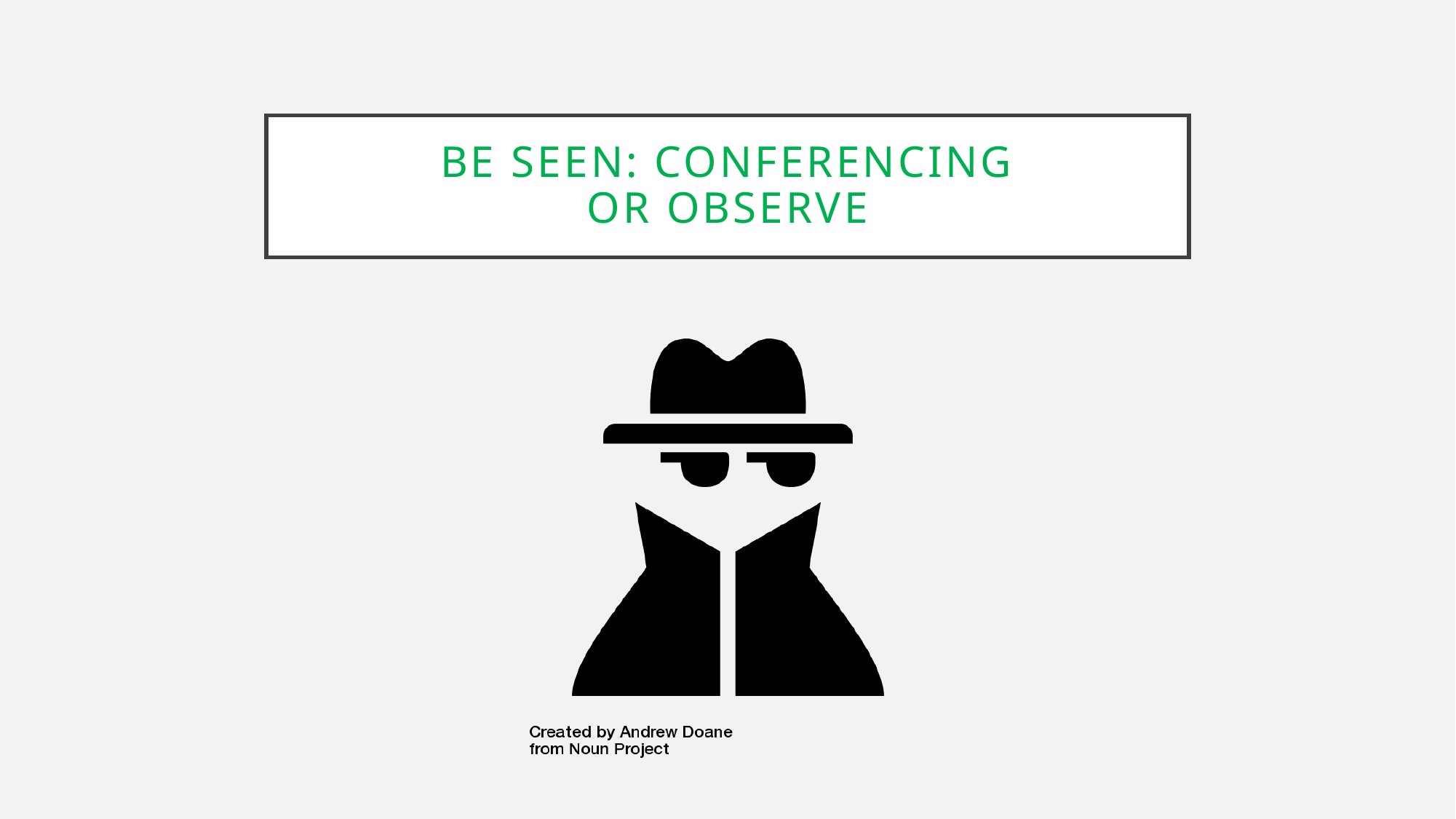

# Be Seen: CONFERENCING Or Observe

## Slide 33
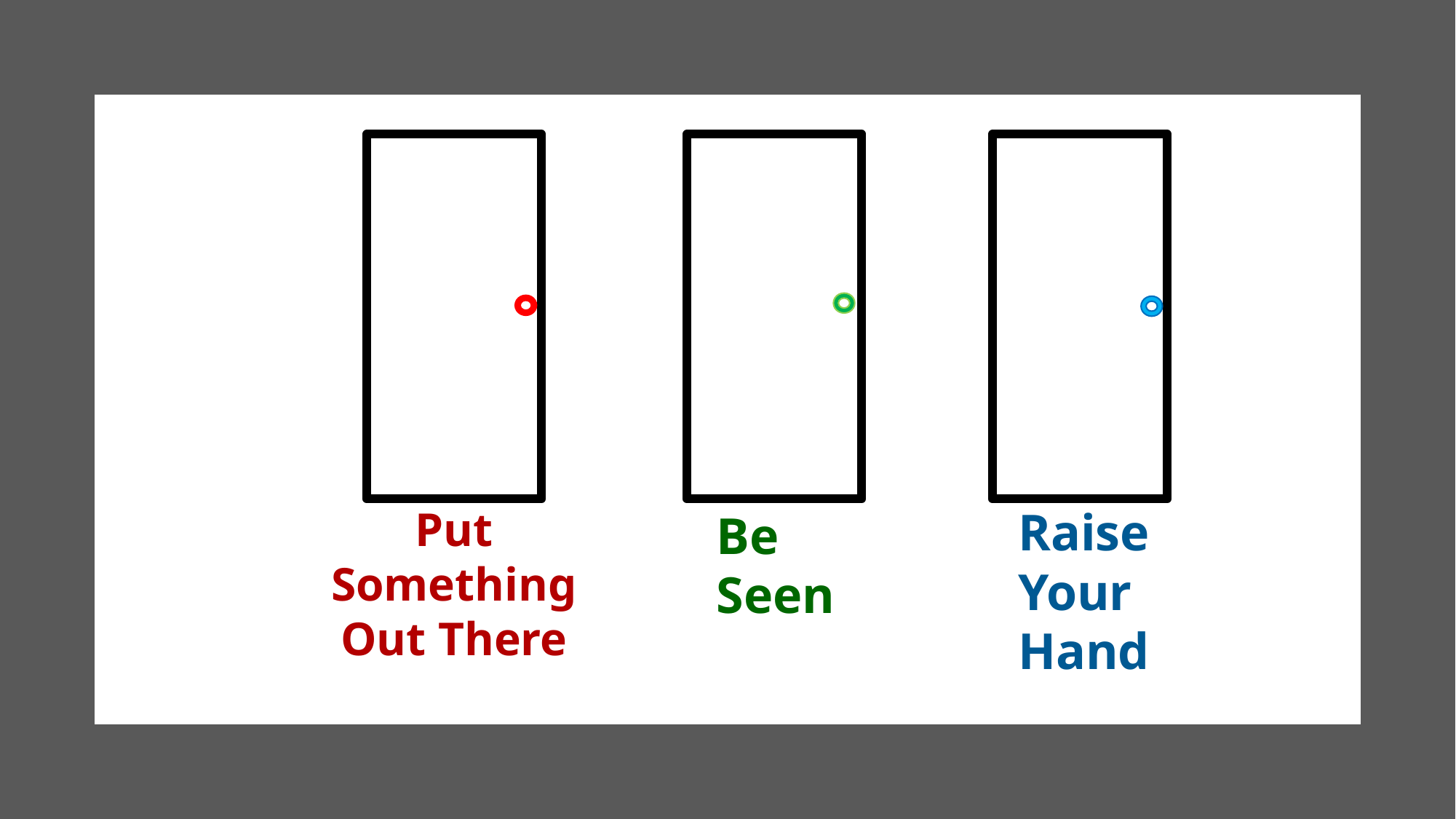

Put Something Out There
Raise Your Hand
Be Seen

## Slide 34
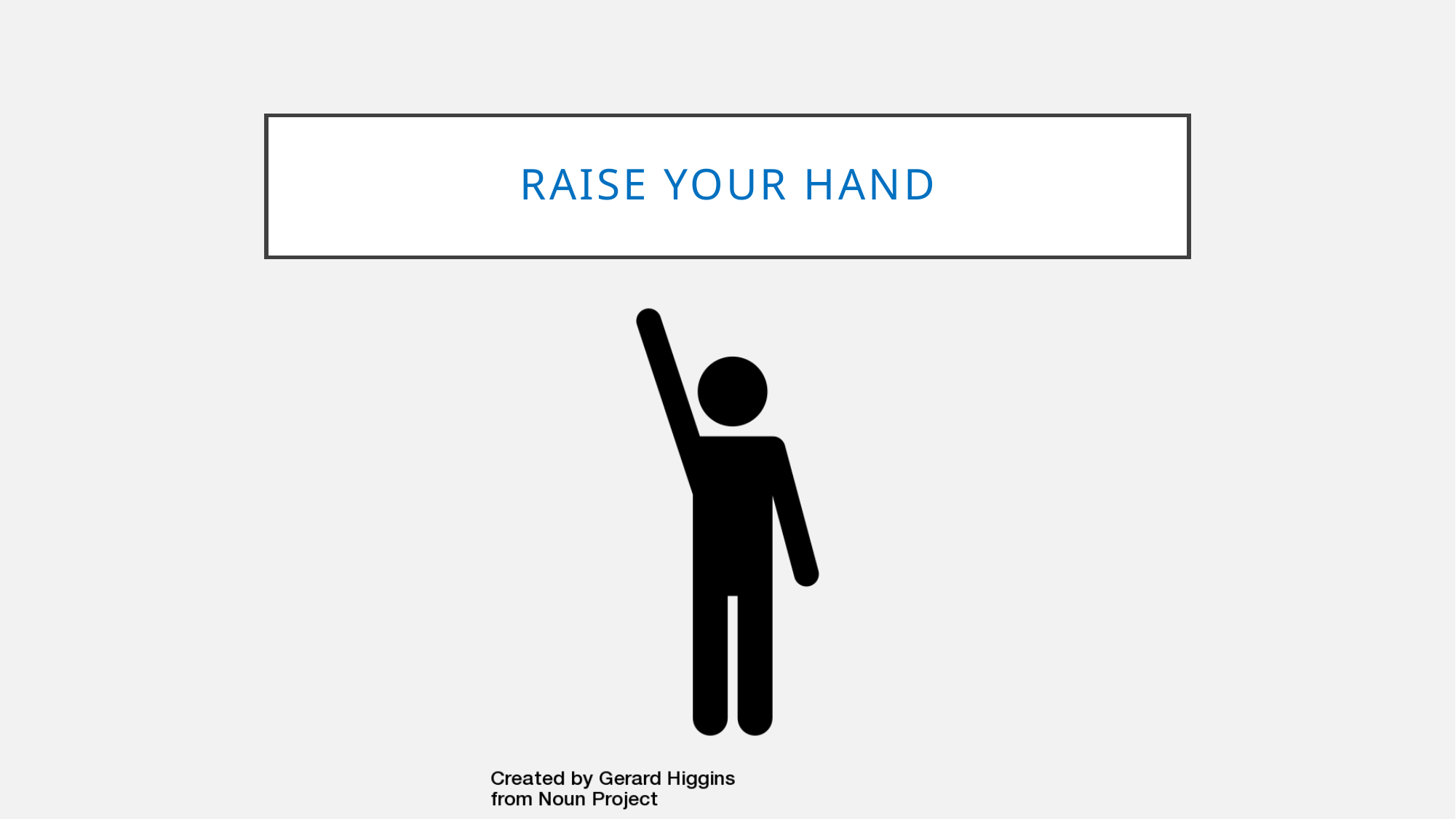

# Raise Your Hand

## Slide 35
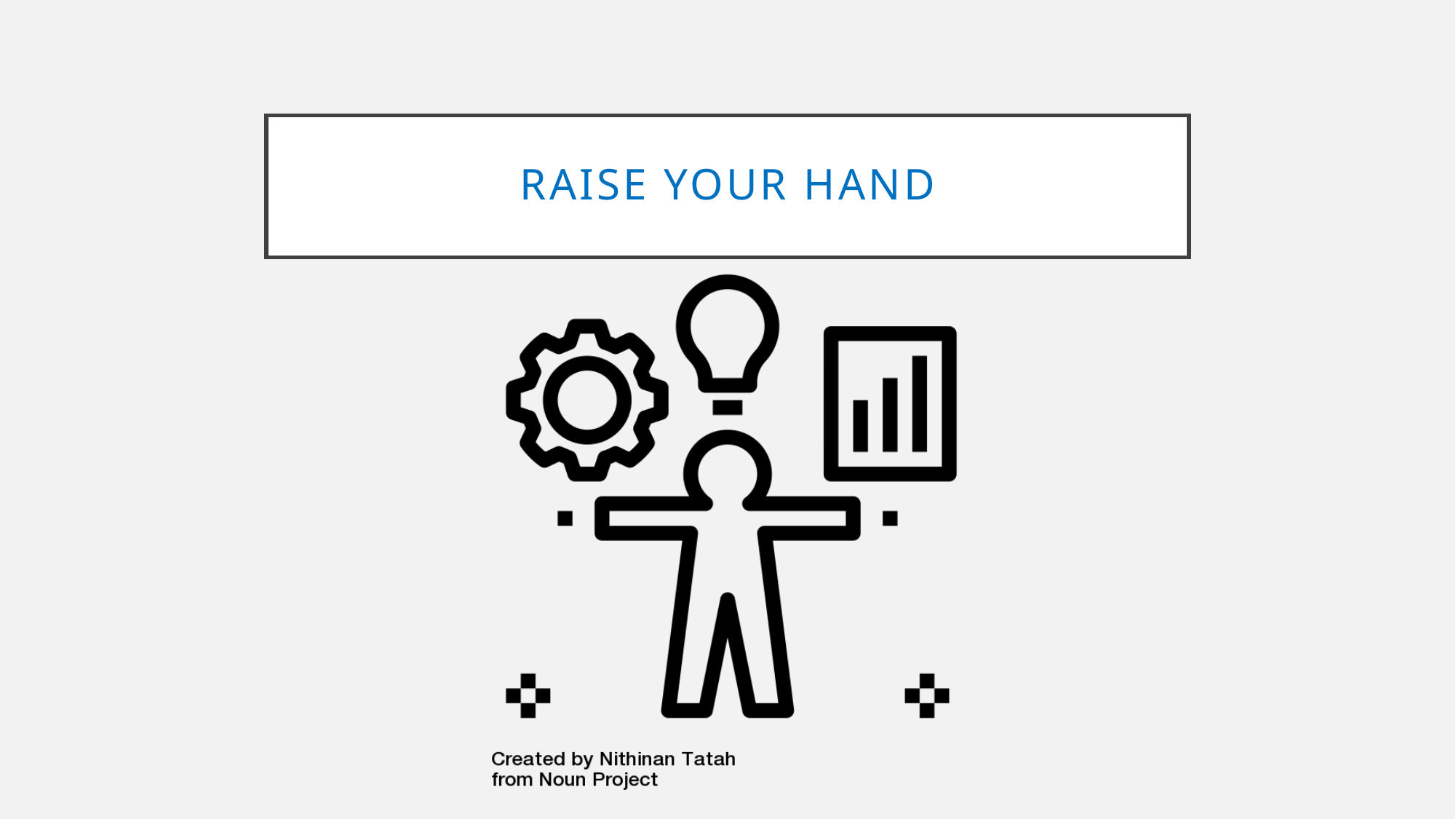

# Raise Your Hand

## Slide 36
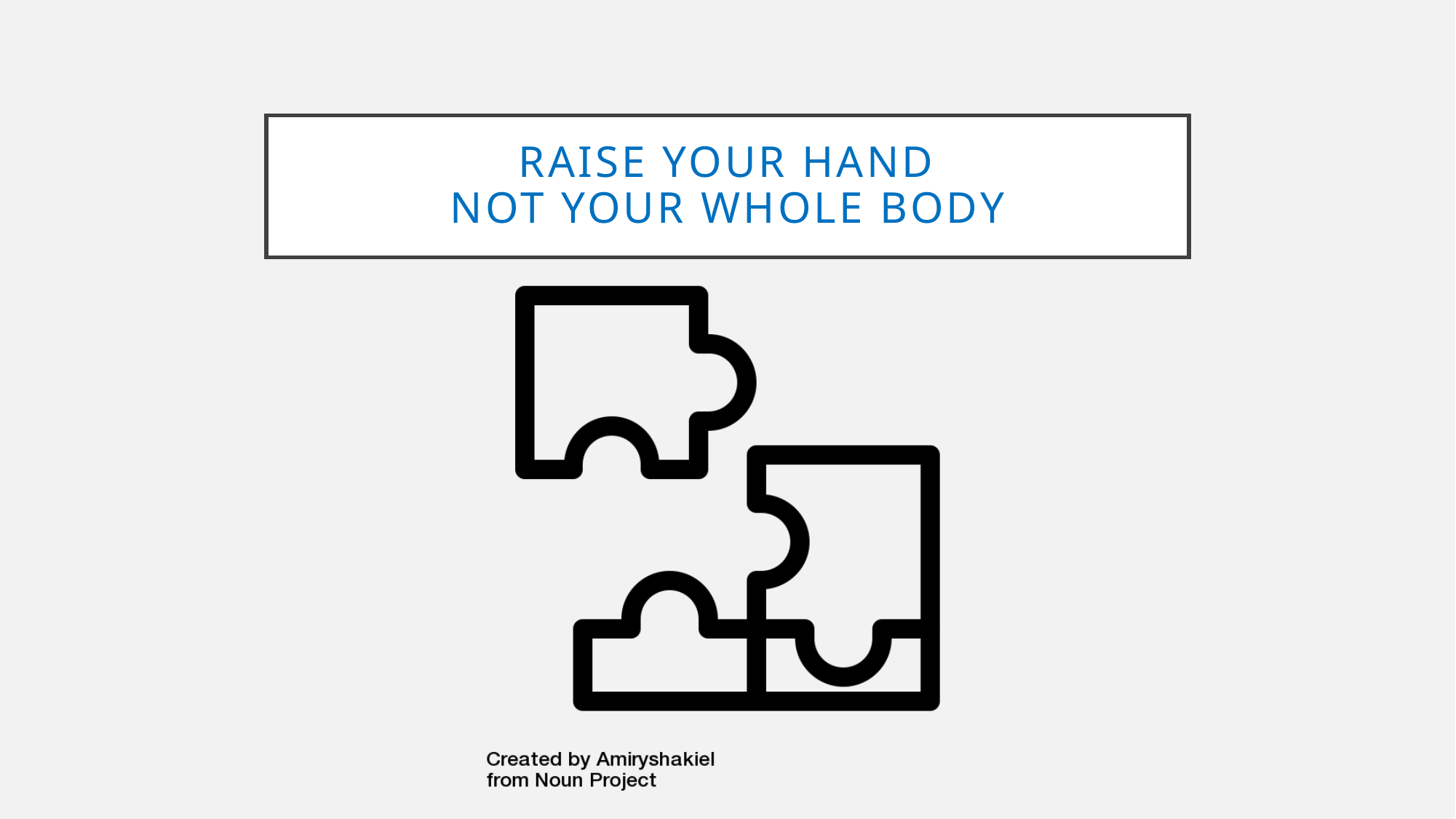

# Raise your hand Not your whole body

## Slide 37
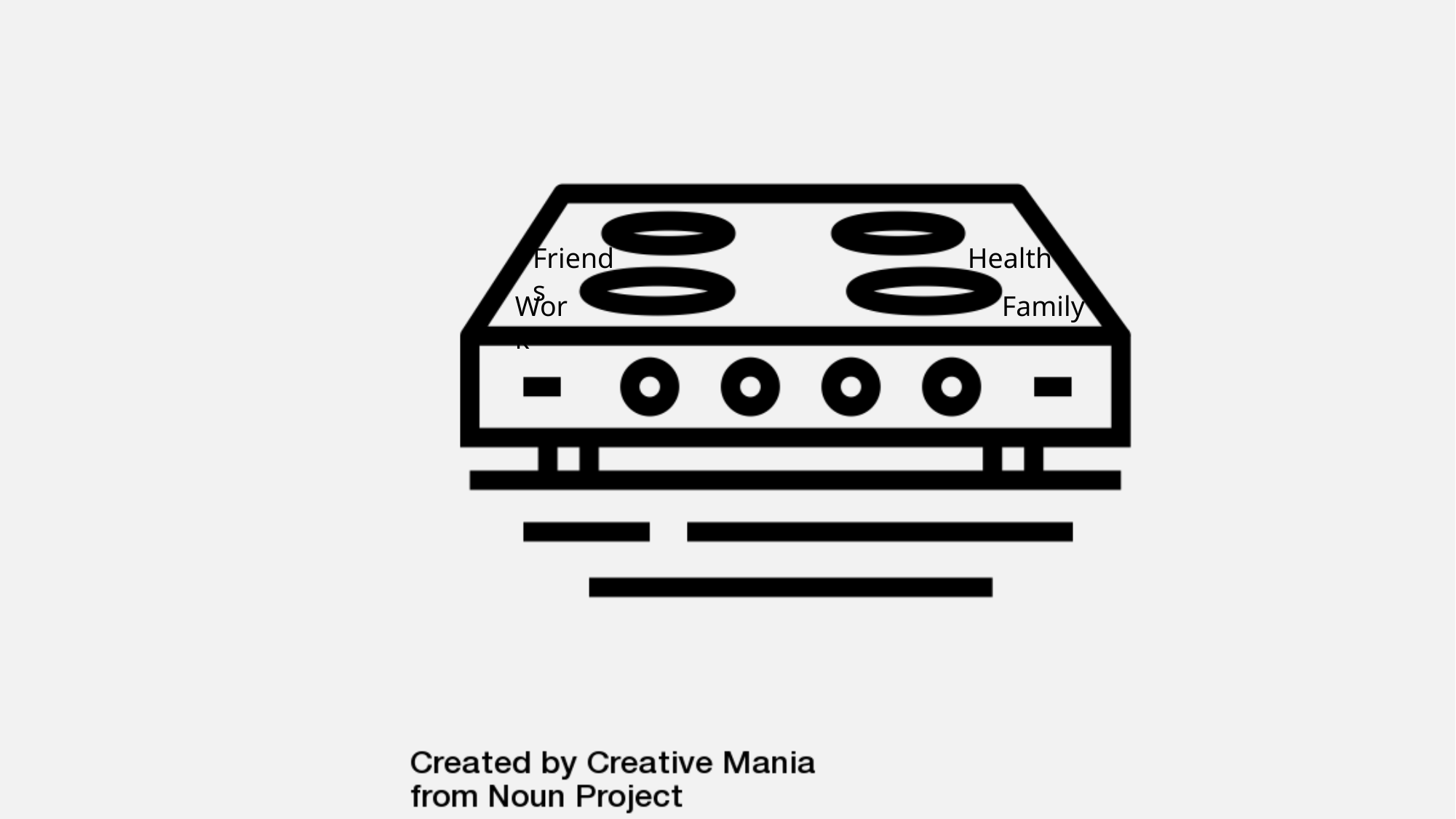

Friends
Health
Work
Family

## Slide 38
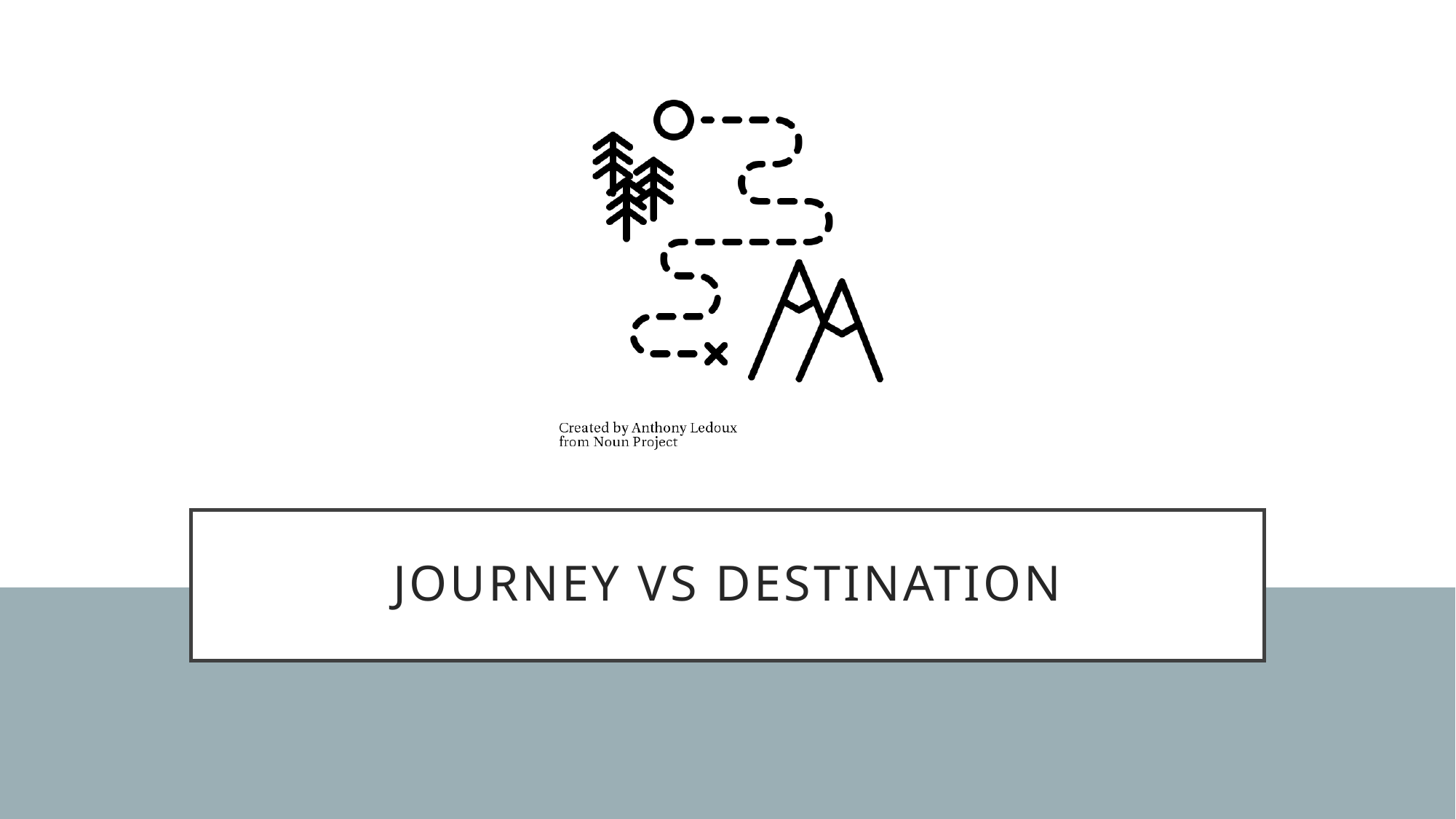

# Journey vs Destination

## Slide 39
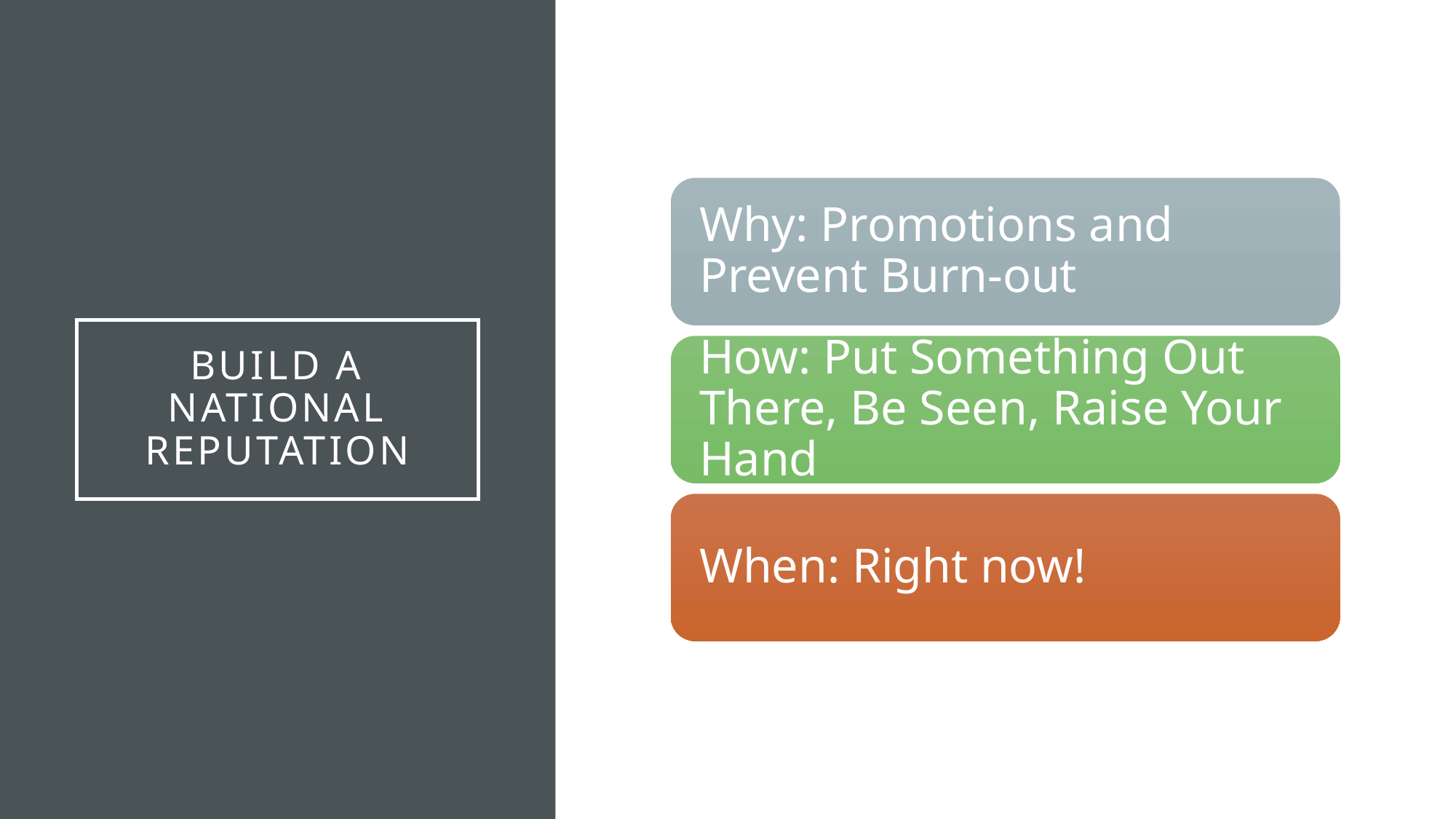

# Build a National Reputation

## Slide 40
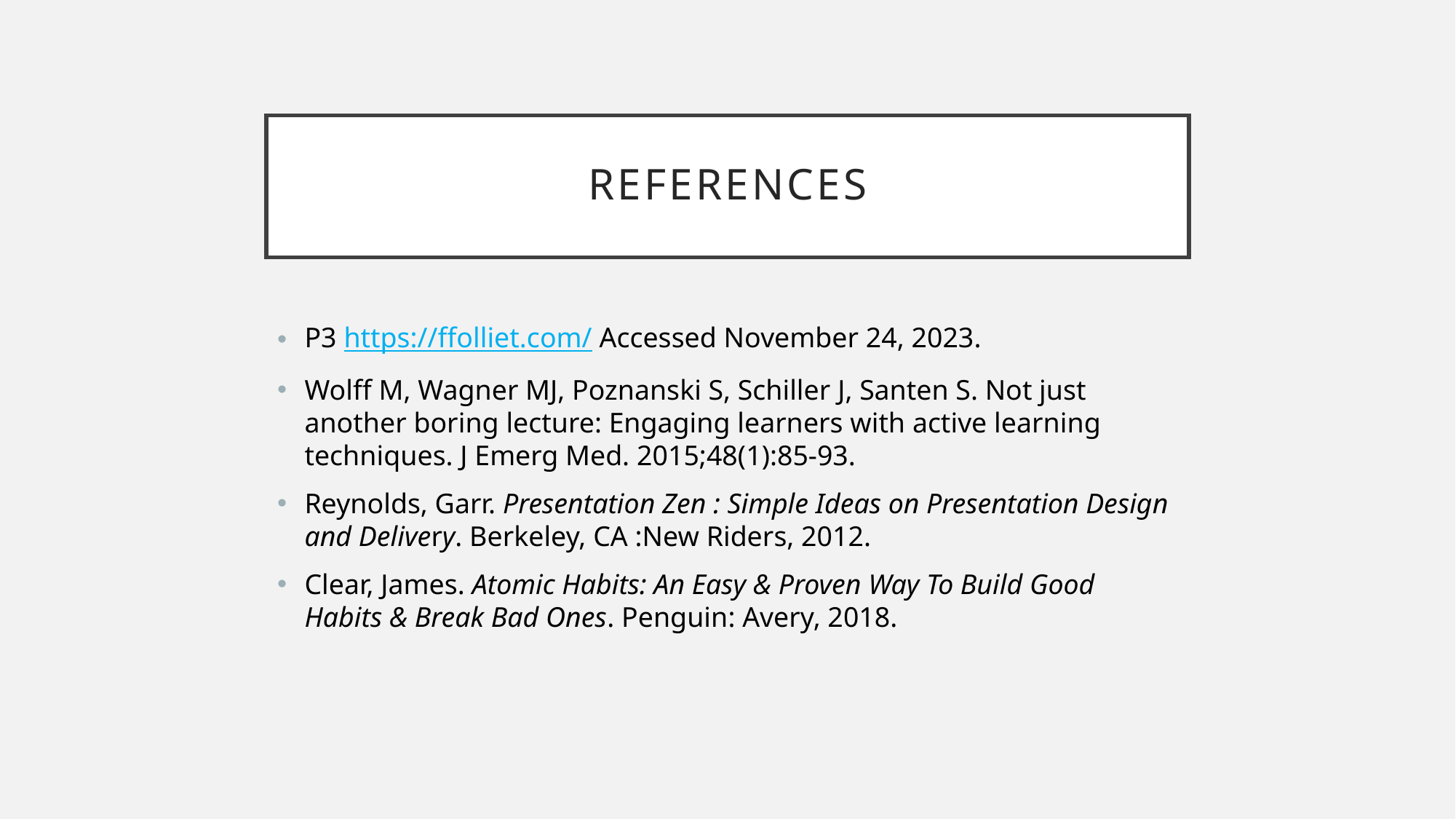

# references
P3 https://ffolliet.com/ Accessed November 24, 2023.
Wolff M, Wagner MJ, Poznanski S, Schiller J, Santen S. Not just another boring lecture: Engaging learners with active learning techniques. J Emerg Med. 2015;48(1):85-93.
Reynolds, Garr. Presentation Zen : Simple Ideas on Presentation Design and Delivery. Berkeley, CA :New Riders, 2012.
Clear, James. Atomic Habits: An Easy & Proven Way To Build Good Habits & Break Bad Ones. Penguin: Avery, 2018.
